# Simple agarose micro-confinement array and machine-learning-based classification for analyzing the patterned differentiation of mesenchymal stem cells

**DOI:** 10.1371/journal.pone.0173647

**Published:** 2017-04-05

**Authors:** Nobuyuki Tanaka, Tadahiro Yamashita, Asako Sato, Viola Vogel, Yo Tanaka

**Affiliations:** 1 Quantitative Biology Center (QBiC), RIKEN, Suita, Osaka, Japan; 2 Laboratory of Applied Mechanobiology, Department of Health Sciences and Technology, ETH Zurich, Zurich, Switzerland; 3 Graduate School of Frontier Biosciences, Osaka University, Suita, Osaka, Japan; Osaka Shiritsu Daigaku, JAPAN

## Abstract

The geometrical confinement of small cell colonies gives differential cues to cells sitting at the periphery versus the core. To utilize this effect, for example to create spatially graded differentiation patterns of human mesenchymal stem cells (hMSCs) *in vitro* or to investigate underpinning mechanisms, the confinement needs to be robust for extended time periods. To create highly repeatable micro-fabricated structures for cellular patterning and high-throughput data mining, we employed here a simple casting method to fabricate more than 800 adhesive patches confined by agarose micro-walls. In addition, a machine learning based image processing software was developed (open code) to detect the differentiation patterns of the population of hMSCs automatically. Utilizing the agarose walls, the circular patterns of hMSCs were successfully maintained throughout 15 days of cell culture. After staining lipid droplets and alkaline phosphatase as the markers of adipogenic and osteogenic differentiation, respectively, the mega-pixels of RGB color images of hMSCs were processed by the software on a laptop PC within several minutes. The image analysis successfully showed that hMSCs sitting on the more central versus peripheral sections of the adhesive circles showed adipogenic versus osteogenic differentiation as reported previously, indicating the compatibility of patterned agarose walls to conventional microcontact printing. In addition, we found a considerable fraction of undifferentiated cells which are preferentially located at the peripheral part of the adhesive circles, even in differentiation-inducing culture media. In this study, we thus successfully demonstrated a simple framework for analyzing the patterned differentiation of hMSCs in confined microenvironments, which has a range of applications in biology, including stem cell biology.

## Introduction

Learning how spatial confinement orchestrate the differentiation processes of cells is essential for the investigation of mechanisms that regulate morphogenesis of multicellular system and tissue regeneration processes [1–3]. While numerous studies have shown the importance of spatial gradients of soluble factors during development [1,4], the importance of spatial patterning [5–11] and of the mechanical environment such as stiffness or surface tethering of the material emerged as additional key factors that regulate cell fate, including that of stem cells [12–19]. Moreover, gradients of mechanical forces can guide the spatially differentiation pattern of stem cell populations [6,20]. The mechanosensory inputs from the environment are converted into cellular signals by various mechanisms, including the stretching of molecules within the force-bearing protein networks by which the extracellular environment is coupled via the cytoskeleton to the cell nucleus and the resulting mechanotransduction processes take an essential role in regulating cell differentiation [21–28]. While many of the mechanisms have been delineated from single cell studies, investigations of differentiation processes of multicellular systems under micro-confined conditions are required to finally close the gap of our understanding how single cell studies might relate to the tissue level.

While numerous patterning methods are currently utilized to investigate how spatial confinement regulates cell functions [29,30], many of them cannot confine cell populations for extended time periods. For example, cells can scrape off microprinted adhesion molecules which often limits the durability of the pattern to several days in some cases [31,32]. In the past, the combination of micro contact printing of cell adhesive molecules such as fibronectin and adsorption of blocking agents such as polyethylene glycol was commonly used for cell patterning [33]. Because of the weak physisorption of adhesive and blocking agent onto the substrate surface, those patterns can be removed by cells in long-term cell culture. This is a particularly pressing problem for the study of stem cell differentiation processes. To improve on the long term-stability of micro patterns, agarose micro structures were recently developed for cell patterning based on repellency [34]. Utilizing the extremely inert property of agarose with respect to cellular adhesion [35] and biomolecular absorption [36], it was shown to successfully contain cellular patterns for more than 10 days [37]. This method does not limit the adhesive types such as poly-L-lysine (PLL) or proteins, therefore, it is suitable for patterning of a variety of surface chemistries, proteins, and cells. Since agarose is commonly available in biological laboratories for various uses [38–40], this patterning method is simple and generic and can easily be applied in a wide context of biomedical research. Here we show that it is well suited for stem cell differentiation experiments that run over extended time periods. The simple and high-throughput agarose micro-patterning technique is combined here with high-throughput machine learning-based image processing, since such experiments involve numerous microphotographs of hMSCs. Early studies [6] have classified the differentiation of hMSCs by superposition of single color stains, for example, Oil Red O staining and Fast Blue staining for adipogenic and osteogenic differentiation, respectively, using color CCD images. While simple thresholding is intuitive, there is room for improvement for high-throughput image analysis with better accuracy and reproducibility. Supported by increasing computational speed, machine learning analysis is getting increasingly popular in biology [41]. Especially, supervised learning approach with support vector machine (SVM) available in open source libraries is quite effective for discriminating data into known classes [42]. SVM algorithm, which is much simpler than the cutting-edge machine learning methods such as deep learning [43], is applied here to classify spatially differentiated hMSCs.

## Materials and methods

### Whole procedure of hMSC culture and analysis

**Fig 1:**
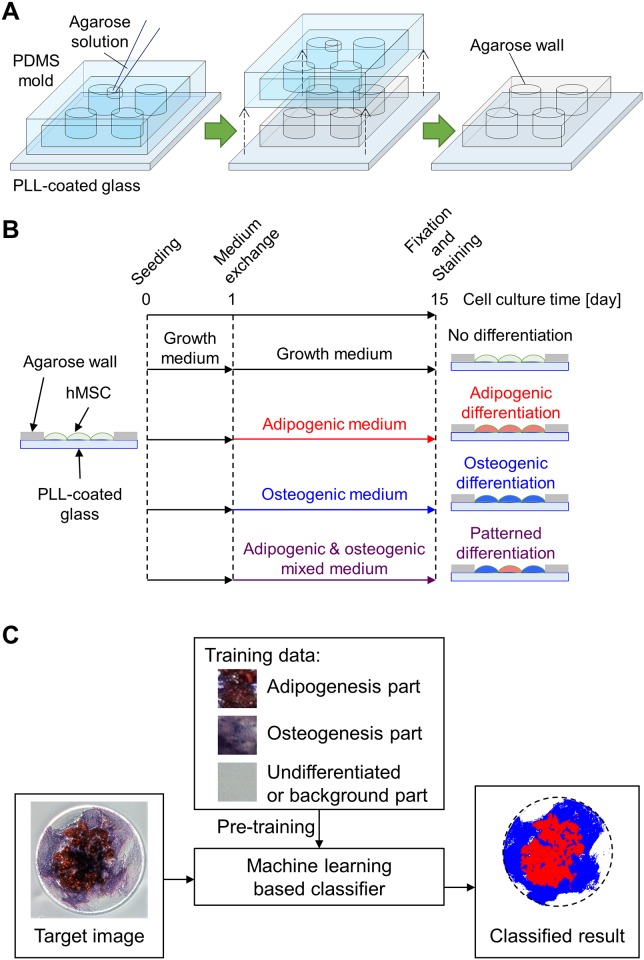
Experimental procedures. (A) Fabrication of agarose micro-wall arrays: Agarose solution was introduced into degassed polydimethylsiloxane (PDMS) mold on a poly-L-lysine (PLL) coated glass culture surface. After gelation and dehydration of agarose solution, agarose micro-wall array was recovered on the glass surface by removal of the PDMS mold. (B) Cultivation and differentiation of human mesenchymal stem cells (hMSCs): hMSCs were seeded on the agarose micro-wall array. After 1-d cultivation in growth medium, the medium was replaced into four different types of culture media; (1) growth, (2) adipogenic, (3) osteogenic, and (4) adipogenic-osteogenic mixture media and hMSCs were cultivated for more 14 days. After the further cultivation, cells were fixed and stained for adipogenic and osteogenic markers and then observed by microscope. (C) Machine learning based classification of hMSC patterned differentiation: The classifier with support vector machine was trained with supervised data composed of small regions of microphotographs selected by a human operator. The trained classifier automatically labeled each pixel based on the classified result.

**Fig 2:**
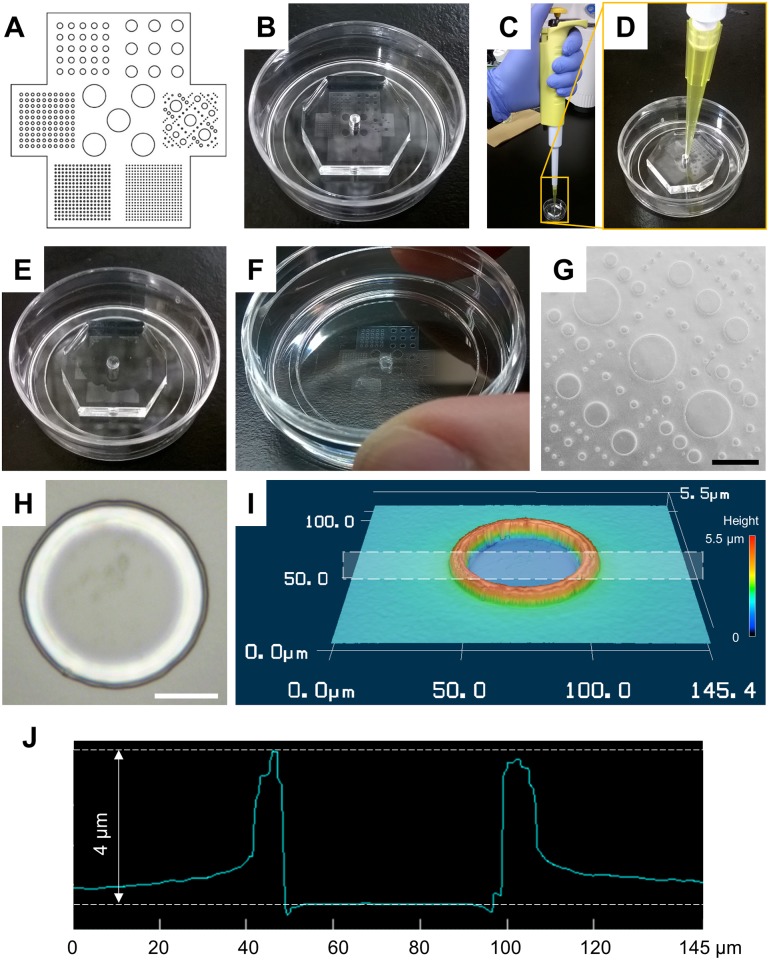
Preparation of agarose micro-wall array. (A) Pattern design. (B) Polydimethylsiloxane (PDMS) mold with the designed pattern was placed on a poly-L-lysine coated glass bottom dish. (C) Application of agarose solution to degassed PDMS mold. (D) Few-hundred-μL-agarose solution was applied from the center hole of degassed PDMS mold. (E) The internal space of PDMS mold was gradually filled with agarose solution without any external pumping. (F) The agarose micro-wall array recovered on the glass surface. (G) Microphotograph of a part of agarose micro-wall array with variety features. Black bar indicates 800 μm. (H, I, and J) Microphotograph, three-dimensional view, and cross-sectional profile of the smallest feature of agarose micro wall. White rectangle in (I) indicates the area of cross-sectional profile (J). White bar indicates 20 μm.

**Fig 3:**
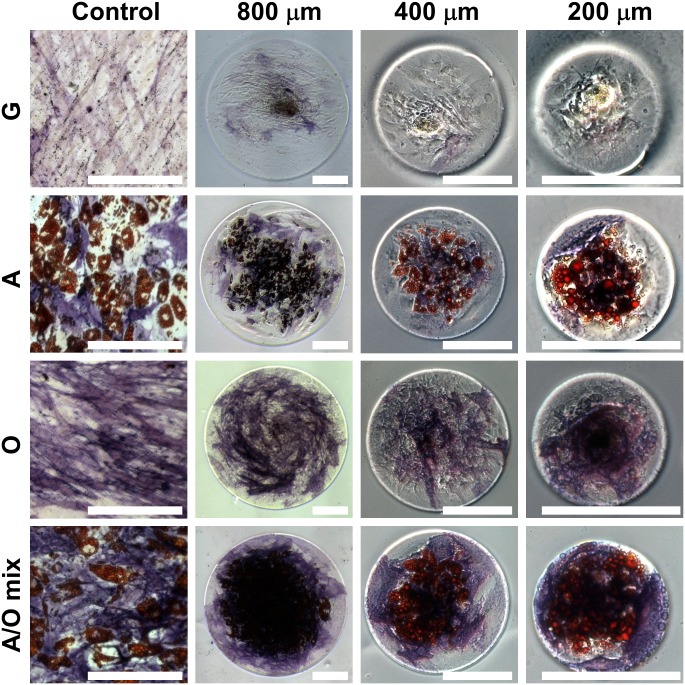
Differentiation of human Mesenchymal Stem Cells (hMSCs) after 15 days in culture. Micrographs from the left to right rows show hMSCs cultivated in the control, 800, 400, and 200 mm-diameter confinements, respectively. Micrographs from upper to lower lines show hMSCs cultivated in growth, adipogenic, osteogenic, and adipogenic-osteogenic mixture media, respectively. White bar indicates 200 μm. G: Growth, A: Adipogenic, O: Osteogenic, and A/O mix: Adipogenic-osteogenic mixed media.

In this study, we designed an experimental framework to investigate how geometrical constraints influence the differentiation of hMSC populations, using materials and technologies that are widely available in biology laboratories. The procedure consists of three steps: (1) fabrication of agarose micro-wall arrays (Fig 1A), (2) cultivation and differentiation of hMSCs (Fig 1B), and (3) machine learning based classification with support vector machine for the microphotograph of differentiation-marker-stained hMSCs (Fig 1C).

### Preparation of micro-molds

PDMS micro-molds were prepared by a photolithographic method [44]. Briefly, negative photoresist (SU-8 2035) (Nippon Kayaku, Tokyo) was coated on a 2-inch-diameter Si-wafer (p-type, mirror-finished <100> surface) (SEMITEC, Chiba) with a spin-coater (1H-D7) (Mikasa, Tokyo) spinning first at 500 rpm for 10 s and then at 4,500 rpm for 30 s more. The photoresist covering the Si-wafer was baked at 95°C for 10 min. The photoresist was exposed to UV-light at an intensity of 9.9×10^3^ mW/cm^2^ for 12 s with a mask aligner (MA-10) (Mikasa) through a photomask having a desired pattern. The photoresist after UV-exposure was baked at 95°C for 3 min and treated with a developer, 2-methocy-1-methylethyl acetate (130–10505) (Wako, Osaka). The remaining photoresist on the wafer was used as a mold for the micro molds. PDMS and a curing agent for PDMS (Sylpot 184 W/C) (Dow Corning Toray, Tokyo) were mixed at a ratio of 10 to 1. The PDMS mixture was poured into the Si-wafer mold and cured at 80°C for 3 h. The cured PDMS was peeled off from the Si-wafer, trimmed, and punched out with a 2-mm-diameter through hole on the center of desired pattern as an application port for agarose solution, resulting in a micro-mold for casting agarose micro wall array.

### Fabrication of agarose micro-wall array

Agarose micro-wall arrays were fabricated on a cell culture surface as previously described [34]. Briefly, the surface of the PDMS micro-mold was cleaned with mending tape (MP-18) (Sumitomo 3M, Tokyo). After rinsed with ethanol and deionized water, the PDMS micro-mold was placed on a PLL coated glass bottom dish (D11141H) (Matsunami Glass Ind., Osaka). The mold and dish were placed in a vacuum desiccator connected to a vacuum line and degassed at a gauge pressure of -98 kPa for 1 hour. Immediately after degassing, the mold and dish were removed from the desiccator and 2w/v% agarose aqueous solution (A2576) (Sigma-Aldrich, St. Louis, MO) heated to boiling in a microwave oven was poured into the hole on the PDMS micro-mold by a micro-pipette. It was placed on a plate heater with a surface temperature of 50°C until the agarose solution was guided into the PDMS micro-mold by negative pressure and completely filled the space inside. The mold and dish were then cooled at 4°C for 30 min to induce the gelation of agarose. The gelated agarose was dehydrated in a drying oven (EO-300B) (ASONE) at 70°C for 2 d. Peeling off the PDMS micro-mold, the dehydrated agarose patterns remained on the surface of the glass bottom dish. The surface of micro-wall was monitored with a three-dimensional laser scanning confocal microscope (VK-8710) (Keyence, Osaka).

### Cell culture

Human mesenchymal stem cells (hMSC) were a kind gift from Dr. Sandra Hoffman. hMSCs were isolated from Poietics^™^ Human Bone Marrow (Lonza, Basel) [45] as previously described [46]. The passage numbers of hMSCs was kept at 6 or below. hMSCs were cultured in growth medium consisting of Dulbecco's modified Eagle's Medium (DMEM, low glucose, GlutaMAX^™^ supplement, pyruvate; 21885) (Thermo Fisher Scientific, Waltham, MA), 10% MSC qualified FBS (12662–029) (Invitrogen, Carlsbad, CA), 1% non-essential amino acids (11140–035) (Invitrogen), 1 ng/mL human recombinant basic fibroblast growth factor (13256–029) (Invitrogen) and 100 unit/mL penicillin and 100 μg/mL streptomycin (15140–122) (Thermo Fisher Scientific, Waltham, MA). To induce the differentiation of hMSCs, adipogenic induction medium (PT-3102B and PT-4135) (Lonza) and osteogenic differentiation medium were prepared. The osteogenic differentiation medium was composed of DMEM freshly supplemented with 50 μg/mL (+)-sodium L-ascorbate (A4034) (Sigma-Aldrich), 1 mM β-glycerophosphate (G9422) (Sigma-Aldrich) and 10 nM dexamethasone (D4902) (Sigma-Aldrich). For expansion, hMSCs were seeded in tissue culture flasks (T75 cm^2^) (TPP, Trasadingen) at a density of 2500 cells/cm^2^. hMSCs near confluence were trypsinized and frozen in MSC qualified FBS containing 10% DMSO (D2438) (Sigma-Aldrich) for further use. For patterned differentiation, hMSCs at passage 5 were thawed in growth medium and cultured in tissue culture flasks for a few days. The fabricated agarose micro-wall array was washed with PBS followed by sterilization by UV for 15 min. It was preincubated with hMSC growth media for more than 30 min in humidified CO2 incubator prior to seeding hMSCs. hMSCs before reaching confluence were trypsinized and then seeded on the adhesive dots surrounded by agarose micro-wall on the glass bottom dish at a density of 2×10^4^ cells/cm^2^. After 24 hours of initial culture with growth medium, the medium was replaced to either growth, adipogenic, osteogenic or mixed medium (adipogenic:osteogenic = 1:1 in volume) and cultured for 14 days. The medium was replaced every 3 days. As a control without patterns, hMSCs were cultured in 12-well plate (92012) (TPP), which was coated with 0.001% poly-L-lysine (P4707) for 1 hour prior to cell seeding (Sigma-Aldrich).

### Staining

After 14 days of cell culture with differentiation or control medium, the cells were once rinsed with PBS and fixed by 4% paraformaldehyde (PFA) (P6148) (Sigma-Aldrich) in PBS for 1 min. Subsequently, alkaline phosphatase was stained with freshly prepared and filtered staining solution containing 1 mg/mL Fast Blue RR salt (F0500) (Sigma-Aldrich) and 200 μg/mL Naphthol AS-MX phosphate (N4875) (Sigma-Aldrich) in 0.1 M Tris buffer (pH 8.5) for 30 min. The cells were then further fixed by 4% PFA in PBS for 20 min. After rinsing with PBS and 60% isopropanol, the cells were stained with 30 mg/mL lipid (Oil Red O) Staining Kit (MAK194) (Sigma-Aldrich) solution in 60% isopropanol.

### Analysis of hMSC differentiation

The stained cells were imaged with an inverted phase-contrast microscope (Axiovert 200M) (Carl-Zeiss, Oberkochen) equipped with a digital color CCD camera (AxioCam MRc) (Carl-Zeiss) with a pixel resolution of 1388×1040. The microscopic images of the cells obtained from the digital color CCD camera were analyzed by a home-made software of machine learning based classifier (available as an open source in S1 File). In this study, the lipid droplets stained by Oil Red O, the alkaline phosphatase-positive part and the area negative to both of the stains were automatically classified as (1) adipogenic, (2) osteogenic and (3) non-differentiated areas, respectively, based on the intensity balance of RGB channels of the color image. The software was coded in the programing language Python 3.5.1 with an open source platform (Anaconda 4.0.0) (Continuum Analytics, Austin, TX) including a computer vision library (OpenCV 3.1.0), a numerical computing library (NumPy 1.10.4), and machine learning library (scikit-learn 0.17.1). For machine learning, the small images mainly containing (1) adipogenic, (2) osteogenic, and (3) non-differentiated areas were first imported to the software as training data. In this step, the software learns the representative distribution of the pixel intensities of those three different areas in RGB space and thus creates a benchmark on how to classify given pixel to those three categories using a support vector machine (SVM). After the training step, color CCD images of hMSCs in dots or flat areas were processed by the software to evaluate the spatial differentiation of hMSCs to adipogenic and osteogenic lineages. Data processing to classify those hMSC differentiation was performed on a tablet style PC (Surface Pro 3) (Microsoft, Redmond, WA). Post analysis of classified images to determine the spatial distribution of differentiated area was performed by MATLAB 2013b (Mathworks, Natick, MA).

## Results and discussion

### Agarose micro-confinement array

A PDMS micro-mold with five different diameters of circle columns (800, 400, 200, 100, and 50 μm) was prepared on the PLL-coated glass bottom dish (Fig 2A and 2B). Agarose solution with a volume of 50 μL was applied from the center hole of the degassed PDMS micro-mold by simply pipetting (Fig 2C and 2D). In this experiment, ultra-low-gelling-temperature type agarose (A2576) (Sigma-Aldrich) was used to prevent the agarose solution from gelling during the poring of the solution into the micro-mold. The solution was then immediately sucked into the space between micro-mold and the surface of glass bottom dish, guided by the negative pressure caused by the degassed PDMS micro-mold (Fig 2E). After the gelation and dehydration of agarose, the micro-cast agarose was created on the glass bottom dish by peeling the micro-mold from the dish (Fig 2F).

The structure of micro-walls was clearly observed in phase-contrast micrographs (Fig 2G and 2H). The structures of PDMS micro-mold were precisely transferred to agarose patterns, thus, it resulted in the agarose wall containing circular vacant spaces with five different diameters corresponding to the original mold (Fig 2G). In the smallest case, the circular cell-adhesive area with a diameter of 50 μm surrounded by dry agarose wall with 4 μm-height was successfully fabricated on PLL-coated glass bottom dish (Fig 2H, 2I and 2J).

The previous study has already revealed that the height of the agarose micro-wall was always shrunk, resulting in round edges of agarose microstructures due to the dehydration of agarose during the fabrication [34]. On the other hand, both high accuracy and high reproducibility of size have been confirmed in both planar and height dimensions except for the edges. Although the edges of agarose micro-walls were often rounded, this hardly effect the cells in this study because the cells were unable to adhere to the agarose surface.

### Cultivation of hMSCs on a PLL surface contained by agarose micro-walls

Within a few hours after seeding, the hMSCs adhered to the PLL-coated glass bottom surfaces. The edge of the agarose micro-wall was clearly observable, indicating that the agarose surface successfully repelled the adhesion of hMSCs. The cells adhered on the circular adhesive areas with 200, 400 and 800 μm of diameters, confined by agarose walls.

After 15 days of cell culture, the hMSCs showed confluence on all of the PLL-coated surfaces either with or without confinement by patterned agarose micro-walls. Throughout the cell culture times with all types of different media, the circular patterns of hMSC were completely maintained (Fig 3). The staining revealed the spatial distribution of lipid droplets and alkaline phosphatase activity that are the markers of early adipogenic and osteogenic differentiation, respectively. Additionally, the whole bottom surface of confinement was filled with a monolayer of cells (S2 File, S1 Fig and S1 Movie), whereby we noted that the cell nuclei closer to the center were higher than those at the periphery (S1 Movie). In general, the hMSCs cultured with osteogenic differentiation factors (O or A/O mix in Fig 3) showed strong alkaline phosphatase activity, while weaker (A) or faint signals (G) were observed in the other conditions. In addition, adipogenic differentiation was observed only in the presence of adipogenic induction factors (A or A/O mix in Fig 3). The spatial distribution of adipogenic and osteogenic differentiation of hMSCs seemed random without agarose patterns.

Importantly, spatial gradients of differently differentiated cells emerged for hMSCs cultivated in a mixed medium (A/O mix in Fig 3). Confirming the observations made by Chris Chen and colleagues [6], hMSCs at the center of the adhesive surfaces preferentially differentiated into adipocytes, while those at peripheral regions underwent osteogenesis. They showed by measuring traction forces revealed gradients of stress that preceded and mirrored the patterns of differentiation, where regions of high stress resulted in osteogenesis, whereas stem cells in regions of low stress differentiated to adipocytes [6]. For hMSCs cultured on microprinted adhesive islands, it was concluded that cells at the edge of the micropattern apply higher traction forces to the substrate as they are lacking cell-cell contacts to nearest neighbors and that cell shape, cytoskeletal tension, and RhoA regulate stem cell lineage commitment [8].

What these authors, however, did not describe or analyze further, presumably due to the inherent instability of the micropatterned edges, is an additional observation made with our method: a considerable fraction of cells does not stain at all when cultured in all of these three induction media, and this fraction of undifferentiated cells is preferentially located as well in the peripheral regions of the confined adhesive islands (Fig 3).

### Machine learning based classification of patterned hMSC differentiation

**Fig 4:**
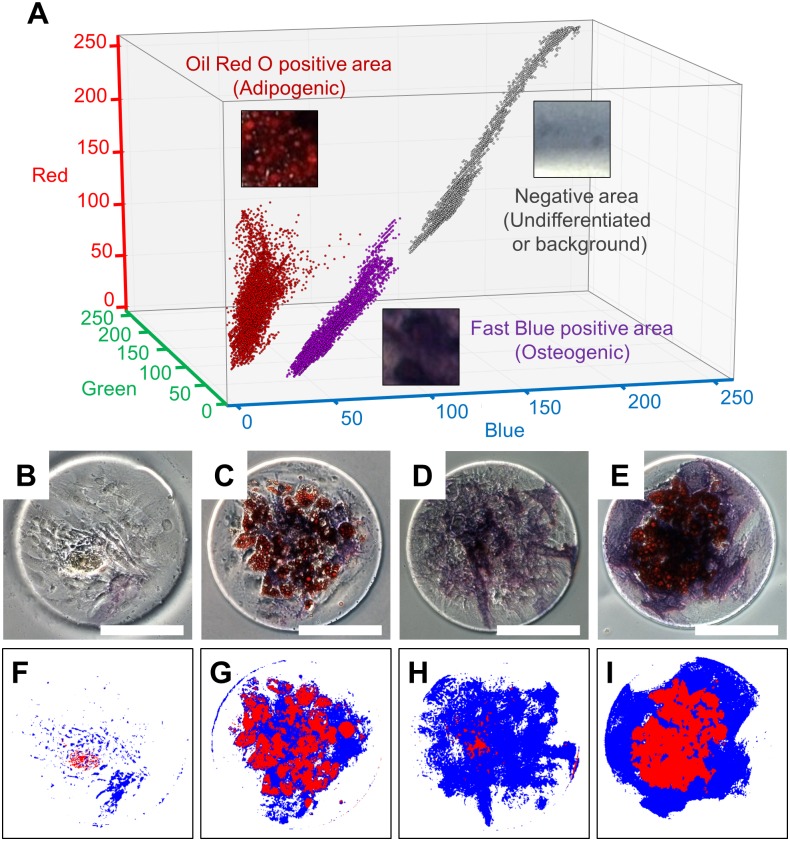
Machine learning based classification of patterned human Mesenchymal Stem Cell (hMSC) differentiation. Graph (A) shows the color distributions of pixels enclosed in (1) OilRed O positive (Adipogenic), (2) FastBlue positive (Osteogenic), and (3) negative (Undifferentiated or background) areas in a red-green-blue (RGB) three-dimensional color space. Microphotographs (B-E) show the stained surfaces of hMSCs cultured in growth, adipogenesis, osteogenesis, and adipgenesis-osteogenesis mixture media, resulting in classified images (F-I), respectively. White bar indicates 200 μm.

For further analysis of the differentiation patterns of hMSCs in confined spaces, we developed a machine learning-based image processing software (S1 File), which automatically classifies adipogenic, osteogenic, and undifferentiated areas of hMSC populations in RGB color images. In the following image analysis procedure, adipogenic and osteogenic areas were characterized by OilRed O staining of lipid droplets (red) and Fast Blue staining of alkaline phosphatase (blue), respectively. The areas negative to both of stains were regarded as resulting from undifferentiated cells. As a pre-training step, three reference groups of microphotographs mainly containing (1) adipogenic, (2) osteogenic, and (3) background were prepared (training data in S1 File and S2 Fig) as machine-learning based classifier. The size of micrographs was around 100×100 pixel square. Those training microphotographs purposely contain several dark areas, where it is hard for human visions to precisely discriminate the colors. The intensities of each pixel in RGB space were plotted in the 3D scatter graph (Fig 4A). The plots from those three reference areas formed three clusters in RGB color space. Those clusters roughly distributed around the diagonal line from the origin (R = 0, G = 0, B = 0) to the point at maximum value (R = 255, G = 255, B = 255). Based on those representative distributions of RGB pixel intensities of reference areas, the software creates the steric borders in RGB space to classify each pixel of given color image to three categories utilizing a SVM. The training time for the prepared data was 118±0.390 s (mean±SD, n = 3). Since several pixels of different reference images are locating close in RGB space, there is an inevitable error when classifying pixels to three groups. In this case, the accuracy of classification for training data was calculated 98.2% (error / total = 1,621 / 91,184 pixels). After the training step, the color images of hMSCs were processed by the pre-trained machine learning based classifier to distinguish the (1) adipogenic, (2) osteogenic, and (3) undifferentiated areas (Fig 4B–4I and S3–S6 Figs). The classification results appear to be accurate and the analyzing time per a target image with a pixel resolution of 1380×1040 was 53.5±0.146 s (mean±SD, n = 54), which is realistic scale for biological image analysis. Some pixels are detected adipogenic in the image of the hMSCs cultured with osteogenic medium (Fig 4D and 4H). The lipid droplets produced in adipogenic cells has at least several μm in size, therefore, those dispersed pixels observed in osteogenic condition are thought to be inevitable errors of classification described previously. Those errors could be corrected by further image filtering. However, we used those classification results to keep the process simple, because evaluated 98.2% of accuracy was enough for our image analysis.

The automatic classification by machine learning-based method utilized here has two merits for analyzing hMSC patterns: (1) elimination of threshold determination and (2) reduction of analysis time. Manual partitioning and classification of microscopic image are typically achieved by careful tuning of threshold values. While simple thresholding works intuitively and efficiently for intensity-based monochrome images such as fluorescent image, it gets increasingly difficult to set the proper thresholds as the dimension of information increases such as color images. This is obvious from Fig 4 that simple thresholding does not work to segment the three clusters even in three-dimensional RGB space. Even in cases where a rough partitioning of differentiation pattern is available, manual operation in single pixel level is not practical for analyzing hundreds of images in terms of reproducibility and analysis time. In our machine learning-based approach, SVM automatically finds the optimized borders of different clusters in RGB space from reference images and thus achieved more accurate and reproducible classification of differentiation patterns than manual partitioning in single pixel level. Since the staining methods of hMSCs are often limited to non-fluorescent dyes, this machine learning-based classifier could be a simple and useful tool to analyze the cells in color images.

In comparison to the deep learning approach gaining attention recently [47], deep learning based image processing enables us to realize a more complex analysis where a target image pixel was classified by the information of not only the target single pixel but also pixel information of its surrounding; however, the computation cost of deep learning is around 10-times larger than that of SVM used in this study. Therefore, the machine learning algorithm should be carefully selected to match the complexity of problem and availability of the computational resources.

Software based analysis tends to occupy human resources to develop the software. In this study, the software was composed of mainly two parts; image processing and machine learning classification. The image processing part mainly performed the transformation of image data into the digital data based on the open-source image processing library “OpenCV.” The classification part performed machine learning, which decided the threshold to divide the different classes and actual classification to the digital data of images into the classes based on the decided threshold in learning phase. This classification part is based on a classical algorithm; support vector machine, which is also available in the other open-source library “scikit-learn.” Incorporating these conventional algorithms into the original code of this study via open-source library could suppress the cost software development. Even though illustrated here using spatial gradients of hMSC patterns, our advanced machine-learning-based data analysis process, including deep learning based image processing, should find a wide range of applications in biomedical research, as we are providing the original code as an open source.

### Spatial analysis of hMSC differentiation

**Fig 5:**
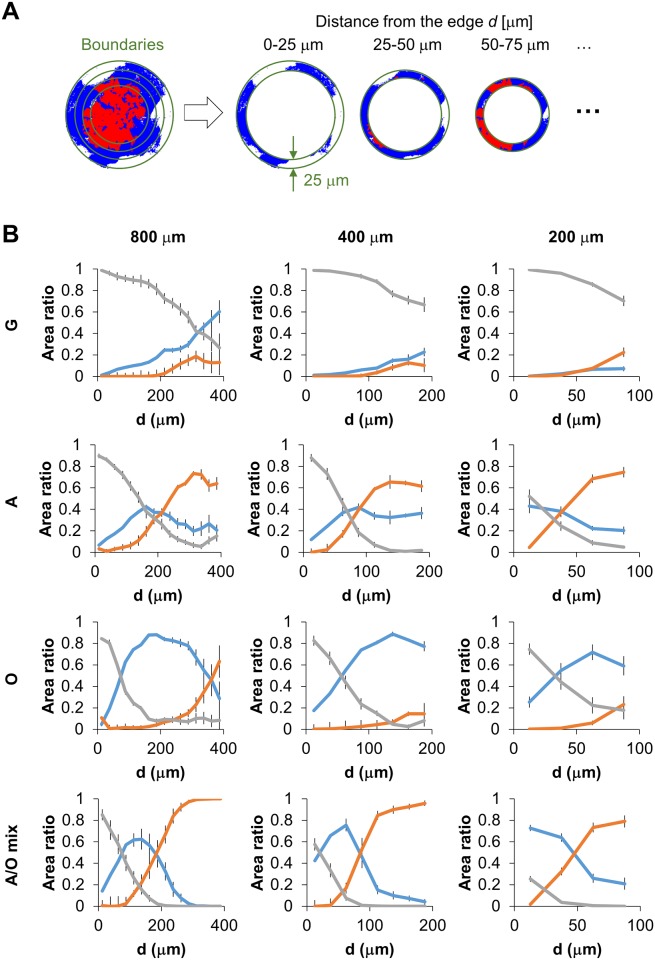
Segmental analysis of fraction of human Mesenchymal Stem Cells (hMSCs) differentiated into different linages. (A) Post-process to determine the localization of hMSC patterned differentiation. (B) Graphs from the left to right rows show the relationships between the ratio of hMSC differentiation pattern and the distance from the periphery of agarose wall with diameters of 800, 400, and 200 μm, respectively. The lines of graphs from upper to lower indicate hMSC cultivation conditions; growth, adipogenic, osteogenic, and adipogenic-osteogenic mixture media, respectively. The error bar in each graph indicates the standard deviation (Number of analyzed images showing a single confinement: N = 5, 5, and 12 for 800, 400, and 200 μm confinements, respectively).

To quantify the geometrical effect of agarose walls, the spatial distribution of hMSC differentiation was analyzed (Fig 5). The images obtained from the machine learning-based classifier were segmented into coaxial ring-shaped regions with 25 μm of thickness (Fig 5A). The fractions of adipogenic (red), osteogenic (blue) and undifferentiated (white) areas in each region were calculated. The results were plotted with respect to the distance from the center of the adhesive circles (Fig 5B). Again, we qualitatively confirm the trend that hMSCs showed preferential adipogenic versus osteogenic differentiation at central versus peripheral parts of the adhesive islands, respectively, as reported previously [6]. Our cellular patterning method by agarose micro-wall reproduced the patterned differentiation of hMSCs in confined space (Fig 3 and target data in S1 File), as previously observed with conventional micro contact printing, confirming that the emergence of patterned differentiation is driven by geometrical confinement. However, the fraction of undifferentiated cells had not been quantified before. Here we show now that the largest fraction of undifferentiated cells (cells that did not show lipid droplet production nor alkaline phosphatase activity) were preferentially found close to the confining agarose walls (A, O, and A/O mix in Figs 3 and 5B). Even in adipogeneic medium, cells located on the middle area between the vicinity of confining walls (undifferentiation), and the central part (adipogenesis) were slightly stained ALP positive (A in Figs 3 and 5B). In many images, undifferentiated cells separated the ALP positive regions from the confining agarose walls (Fig 3 and S3–S6 Figs). Future research has to provide further insights into the mechanisms of our findings.

## Conclusion

Here we introduced a new framework to analyze the patterned differentiation of hMSCs in spatial confinement and we illustrate that confinement of hMSCs by agarose micro-walls generate the same trends as previously reported when using conventional micro contact printing approaches [6]. Since this is primarily a method paper, it should be noted that this repellency-guided confinement can be exploited for a large range of biomedical applications. While we used PLL-coated glass bottom for this report, other surface chemistries or adhesive proteins such as fibronectin, collagen and laminins can be utilized as coatings as different extracellular matrices interact with different integrin subtypes and thereby trigger distinct mechanotransduction pathways [48,49]. Our method is thus a simple alternative when conventional printing approaches do not allow to fabricate sufficiently stable adhesive islands. Machine learning-based image analysis showed efficient classification of differentiated hMSCs, which allowed high-throughput analysis of our bright field images and to quantify the existence and locations of a significant fraction of undifferentiated cells which has not been described previously. This study demonstrated that this simple agarose micro-engineering and image analysis allowed stem cell phenotyping in microenvironments, which is of growing interest in basic biology. This approach can be implemented in typical biology laboratories without the requirement of special instruments, once users have access to PDMS molds.

## Supporting information

S1 Movie Scanning cross-sectional view of a representative confocal microscope image taken from the data set analyzed in S2 Fig. White dashed circle indicates the boundary of confinement. Moving yellow horizontal line indicates the position of cross-sectional view showing the lower view in the movie. White scale bars indicate 100 μm.
(MP4)

S1 File A Python program of machine learning based classifier with training and target data. (ZIP)

S2 File Materials and methods for staining and imaging of cell nuclei. (DOCX)

S1 Fig Confocal microscopy for hMSCs cultivated in 400 μm confinement in adipogenic-osteogenic mixed medium under conditions as in Fig 3. (A) Phase-contrast micrograph. (B) Fluorescent micrograph. Nuclei were stained with blue-fluorescent dye. White dashed circle indicates the boundary of confinement. (C) Merged image between (A) phase-contrast and (B) fluorescent micrographs. White scale bars indicate 100 μm.
(TIF)

S2 Fig Training data for pre-training a machine learning based classifier for human Mesenchymal Stem Cell (hMSC) differentiation. Microphotographs (A-C) show training data for OilRed O positive (Adipogenic), FastBlue positive (Osteogenic), and negative (Undifferentiated or background) areas, respectively. Black angle symbol indicates 50-pixel length in both horizontal and vertical directions.
(TIF)

S3 Fig Machine learning based classification for human Mesenchymal Stem Cells (hMSCs) cultured in the control (without any confinements). Microphotographs (A-D) show the stained surfaces of hMSCs cultured in growth, adipogenesis, osteogenesis, and adipgenesis-osteogenesis mixture media, resulting in classified images (E-H), respectively. White bar indicates 200 μm.
(TIF)

S4 Fig Machine learning based classification for human Mesenchymal Stem Cells (hMSCs) cultured in 800 μm diameter confinements. Microphotographs (A-D) show the stained surfaces of hMSCs cultured in growth, adipogenesis, osteogenesis, and adipgenesis-osteogenesis mixed media, respectively, resulting in classified images (E-H). White bar indicates 200 μm.
(TIF)

S5 Fig Machine learning based classification for human Mesenchymal Stem Cells (hMSCs) cultured in 400 μm diameter confinements. Microphotographs (A-D) show the stained surfaces of hMSCs cultured in growth, adipogenesis, osteogenesis, and adipgenesis-osteogenesis mixed media, respectively, resulting in classified images (E-H). White bar indicates 200 μm.
(TIF)

S6 Fig Machine learning based classification for human Mesenchymal Stem Cells (hMSCs) cultured in 200 μm diameter confinements. Microphotographs (A-D) show the stained surfaces of hMSCs cultured in growth, adipogenesis, osteogenesis, and adipgenesis-osteogenesis mixed media, respectively, resulting in classified images (E-H). White bar indicates 200 μm.
(TIF)
