# Supplementary figures and images for: Simple agarose micro-confinement array and machine-learning-based classification for analyzing the patterned differentiation of mesenchymal stem cells (part 2 of 2)
Source: PLoS One. 2017 Apr 5;12(4):e0173647. doi: 10.1371/journal.pone.0173647 (PMC5381775; doi:10.1371/journal.pone.0173647)

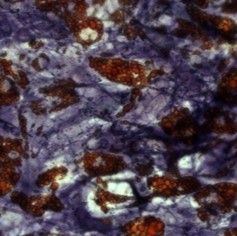

Supplement: S1 File — (ZIP) [file pone.0173647.s002.zip › S1_File/targets/MC_1.jpg]

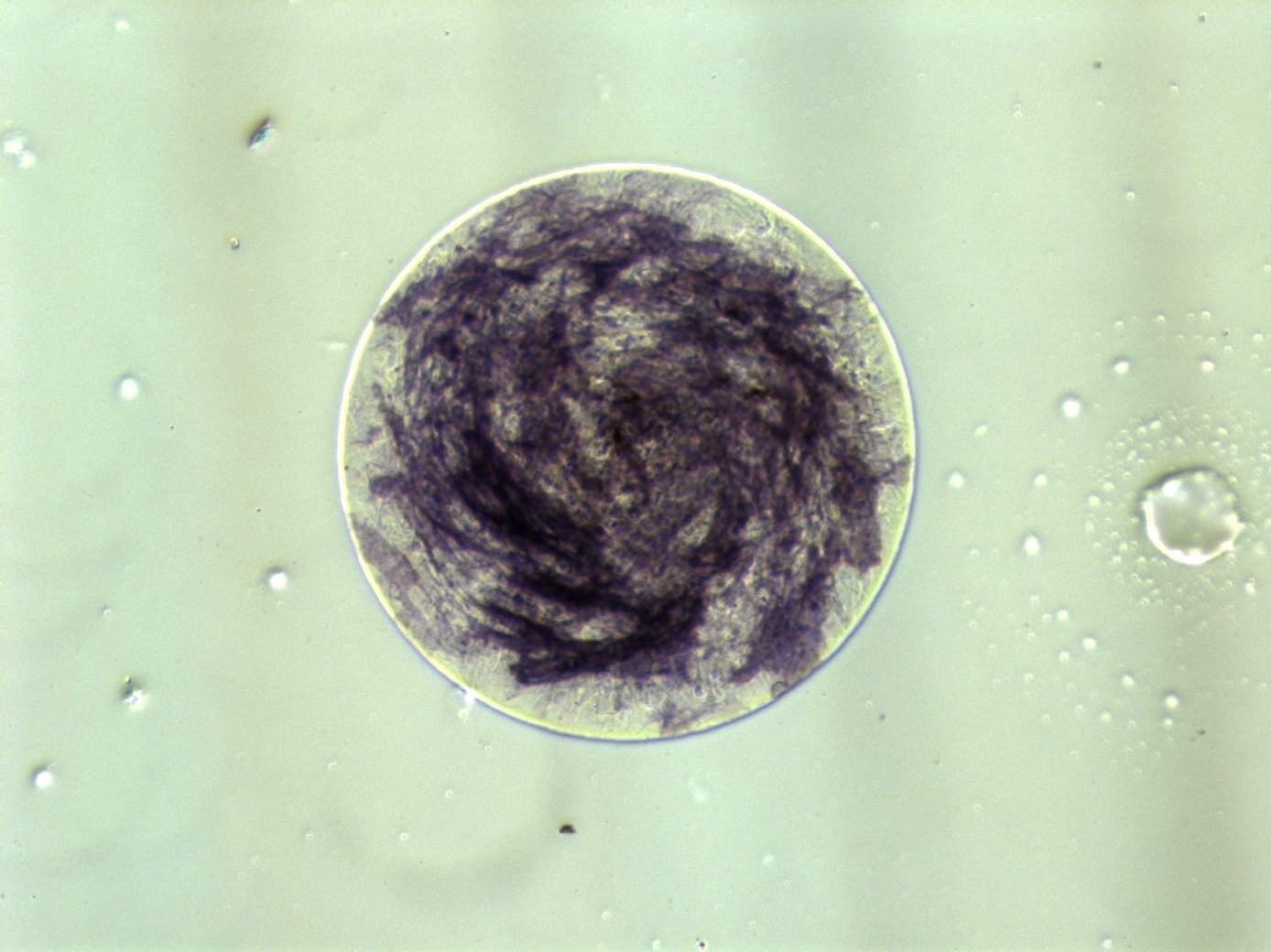

Supplement: S1 File — (ZIP) [file pone.0173647.s002.zip › S1_File/targets/O2_1.jpg]

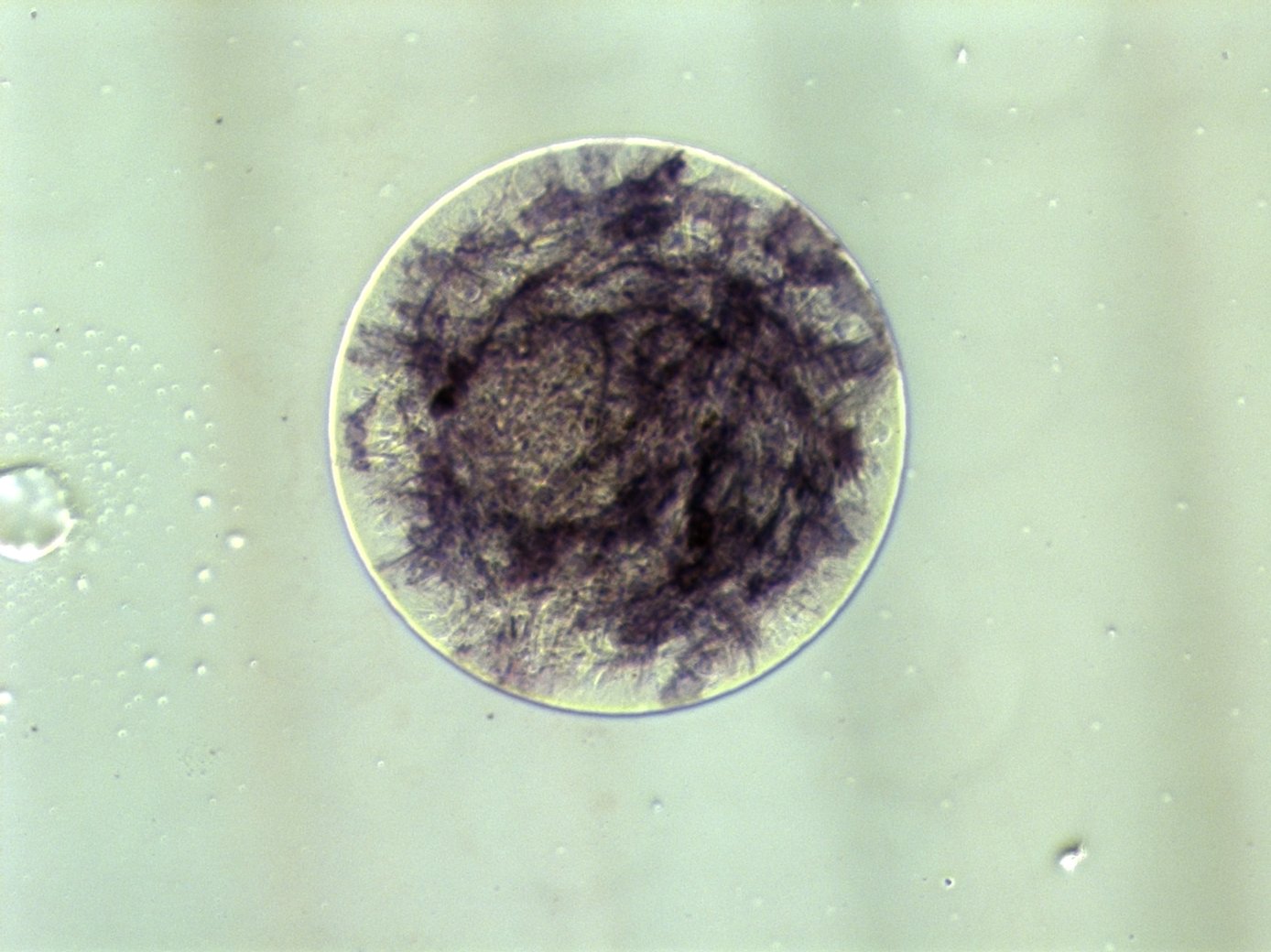

Supplement: S1 File — (ZIP) [file pone.0173647.s002.zip › S1_File/targets/O2_2.jpg]

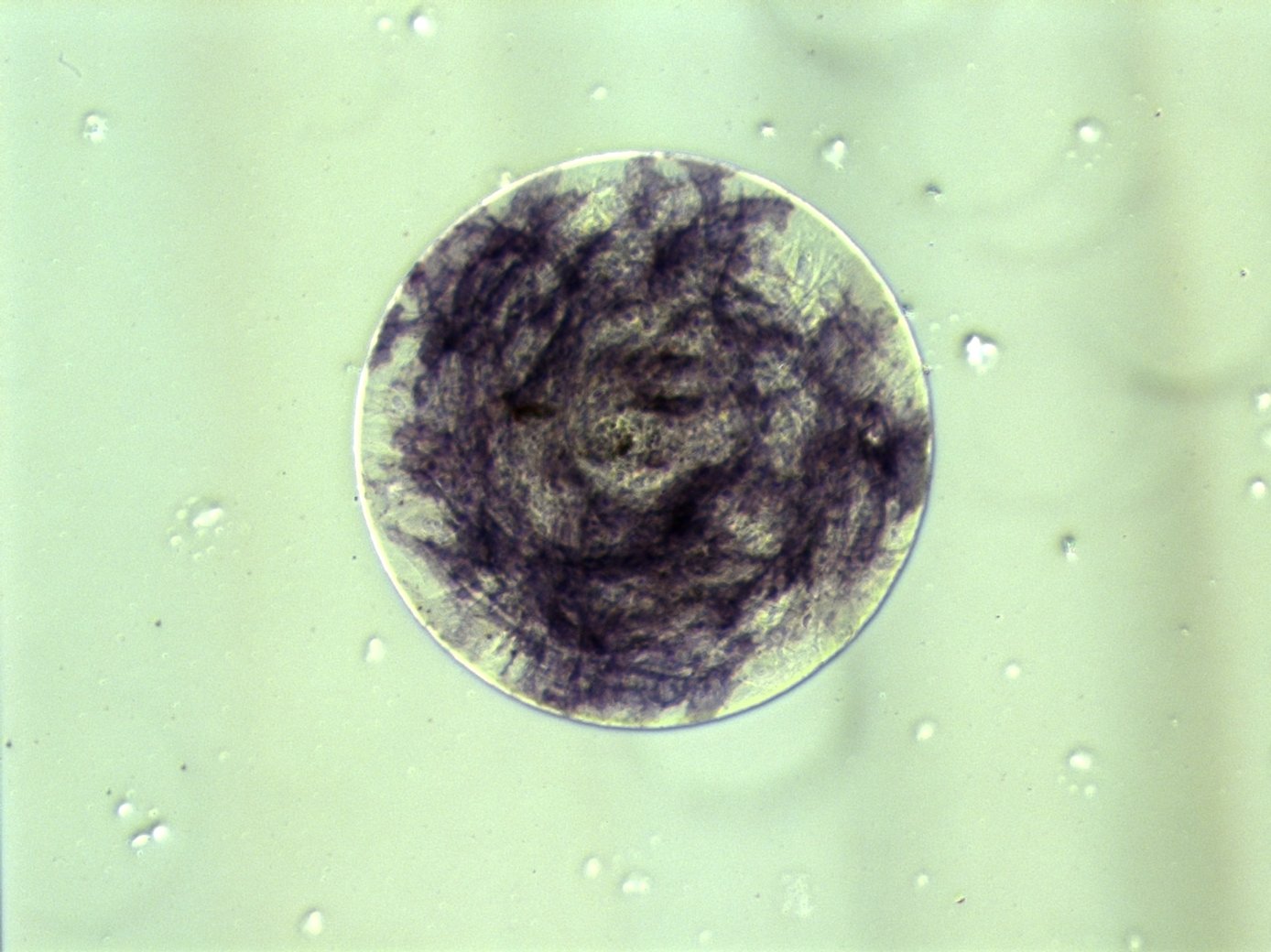

Supplement: S1 File — (ZIP) [file pone.0173647.s002.zip › S1_File/targets/O2_3.jpg]

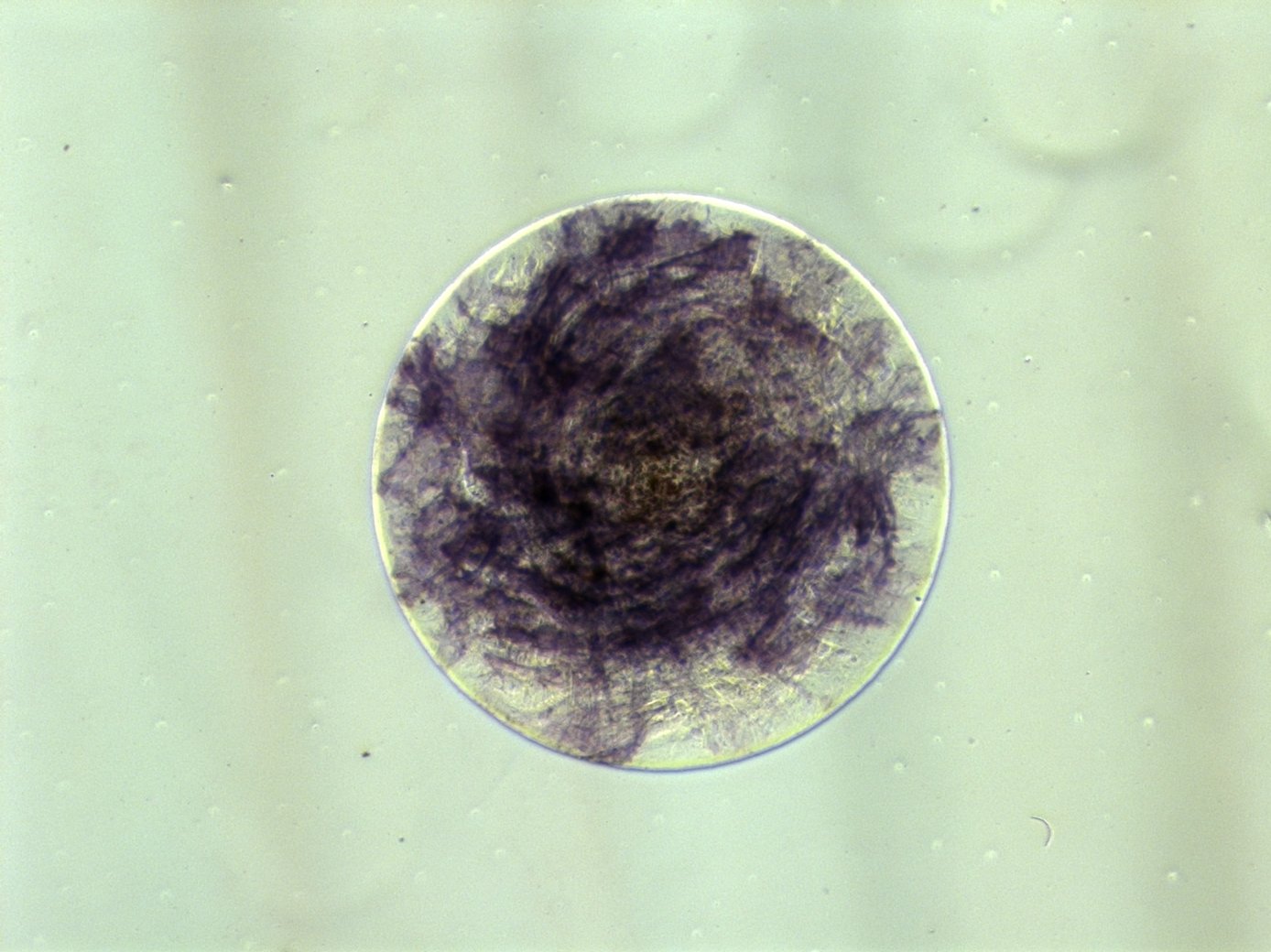

Supplement: S1 File — (ZIP) [file pone.0173647.s002.zip › S1_File/targets/O2_4.jpg]

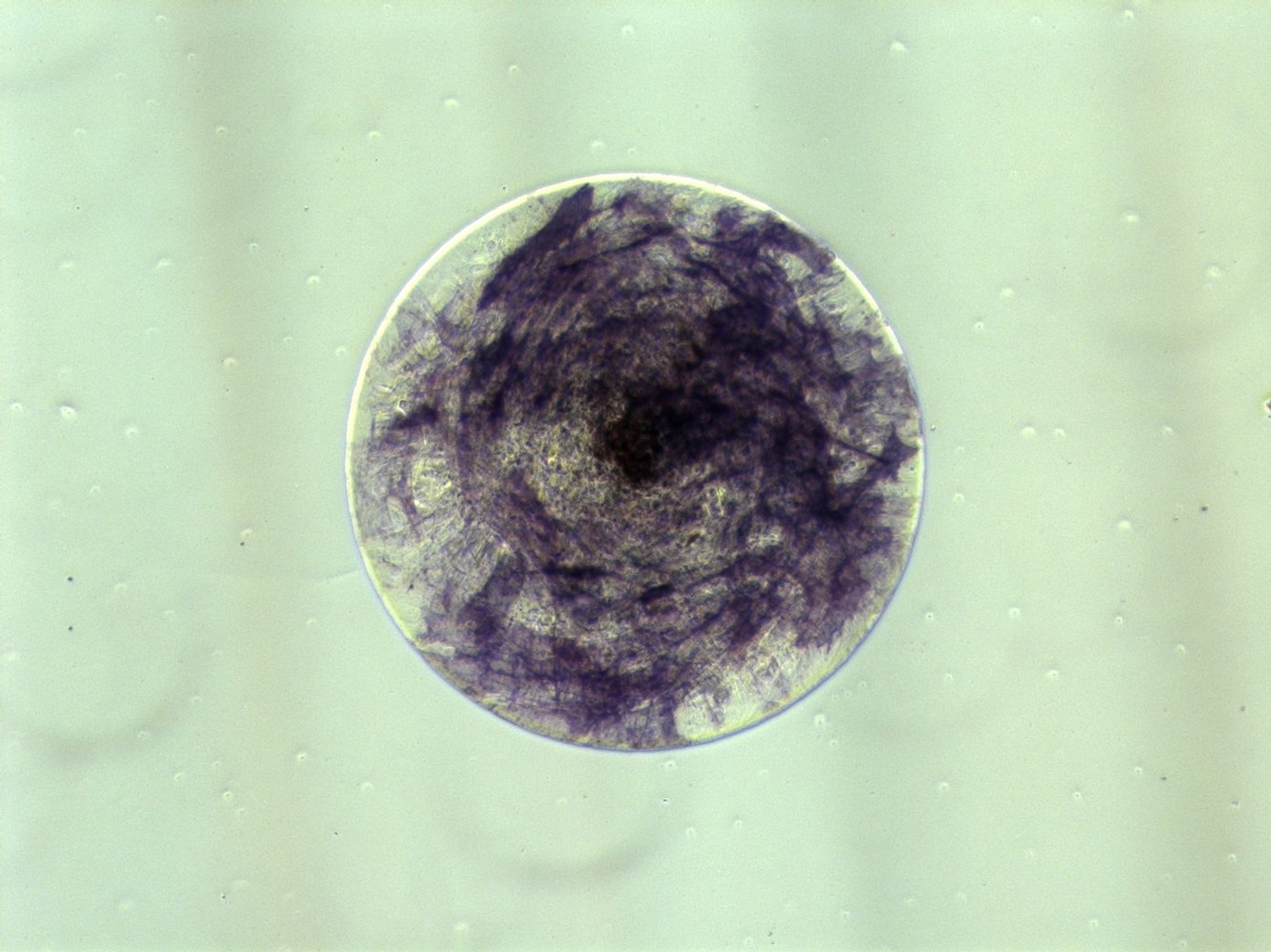

Supplement: S1 File — (ZIP) [file pone.0173647.s002.zip › S1_File/targets/O2_5.jpg]

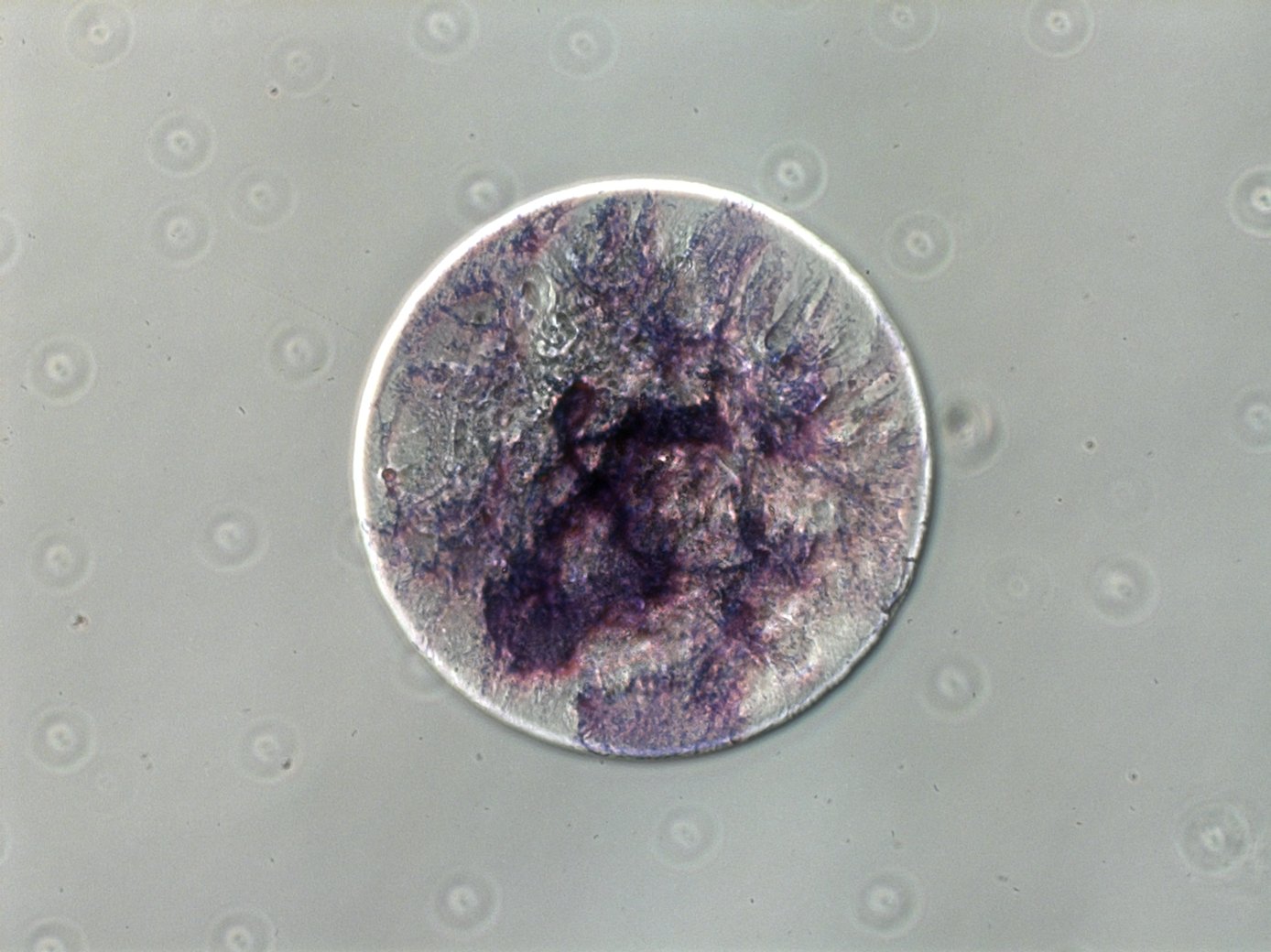

Supplement: S1 File — (ZIP) [file pone.0173647.s002.zip › S1_File/targets/O3_1.jpg]

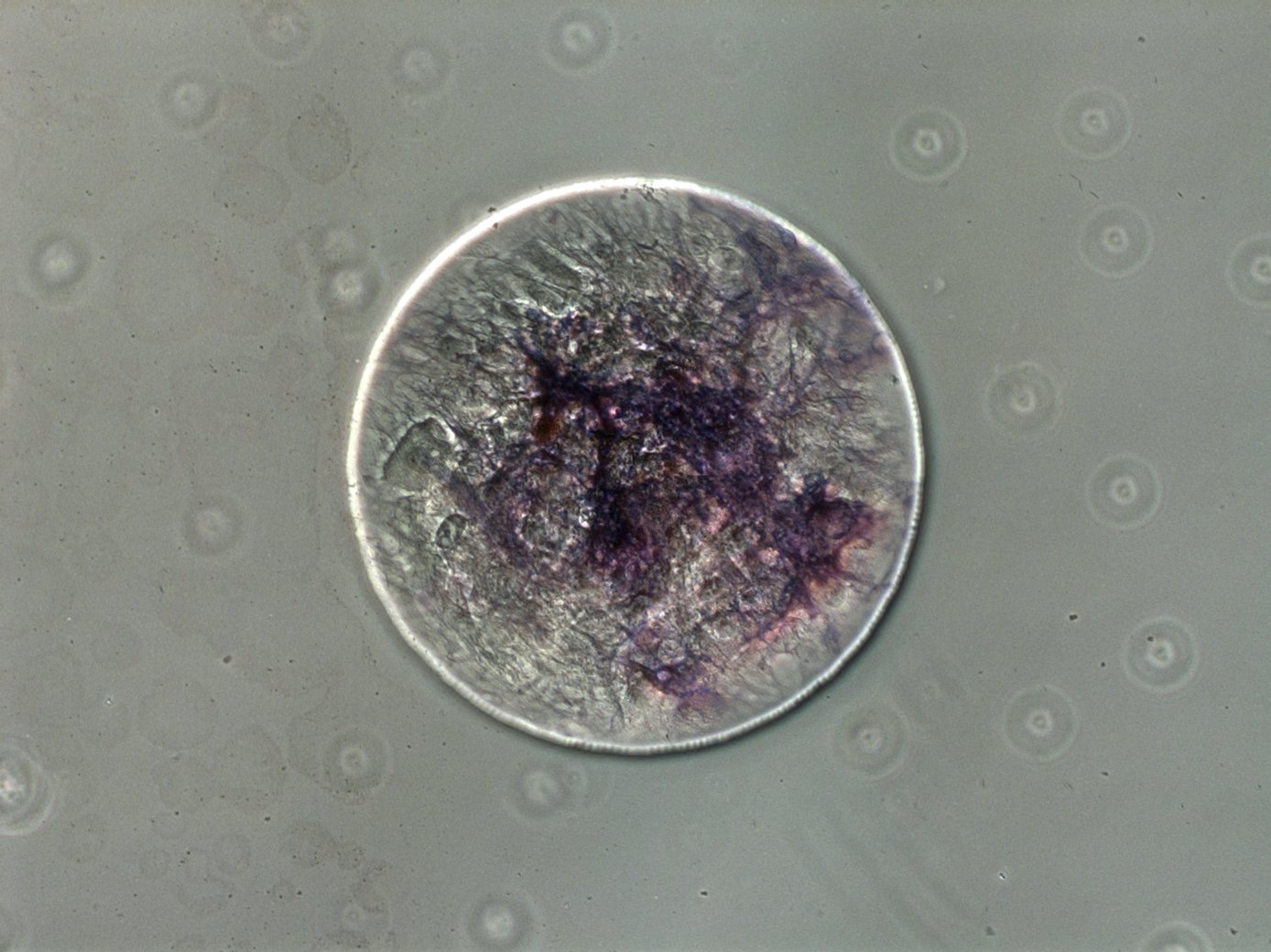

Supplement: S1 File — (ZIP) [file pone.0173647.s002.zip › S1_File/targets/O3_2.jpg]

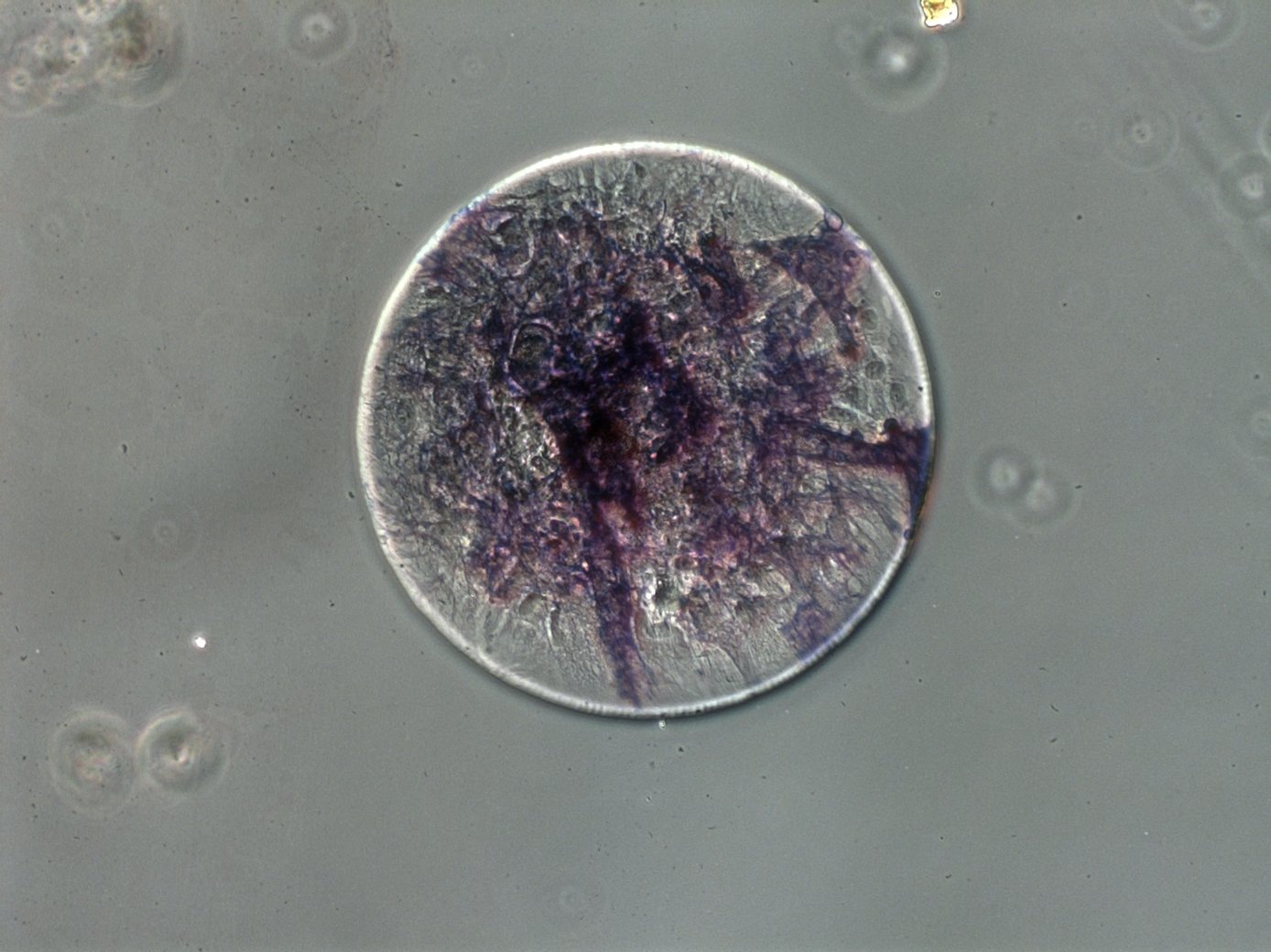

Supplement: S1 File — (ZIP) [file pone.0173647.s002.zip › S1_File/targets/O3_3.jpg]

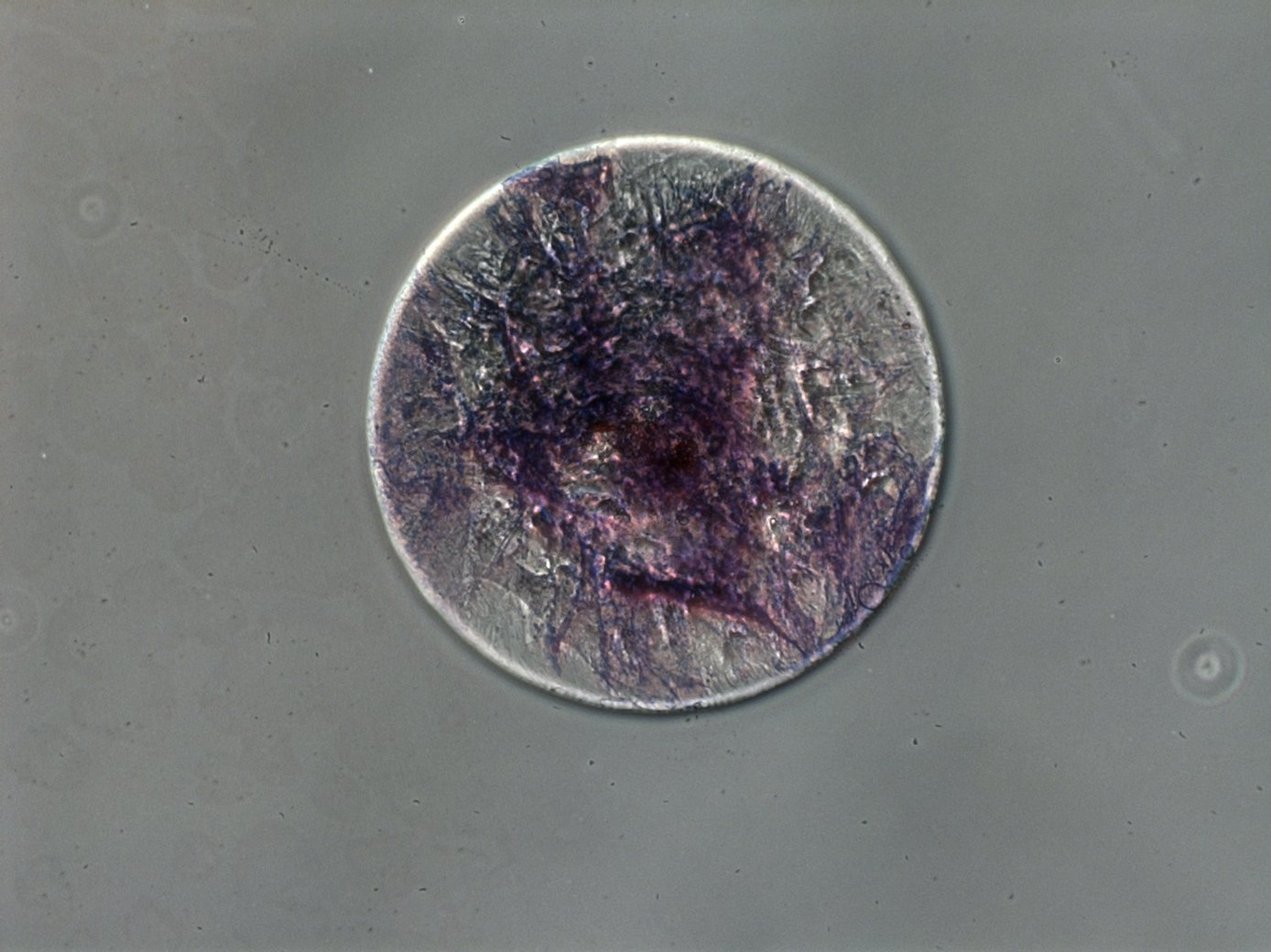

Supplement: S1 File — (ZIP) [file pone.0173647.s002.zip › S1_File/targets/O3_4.jpg]

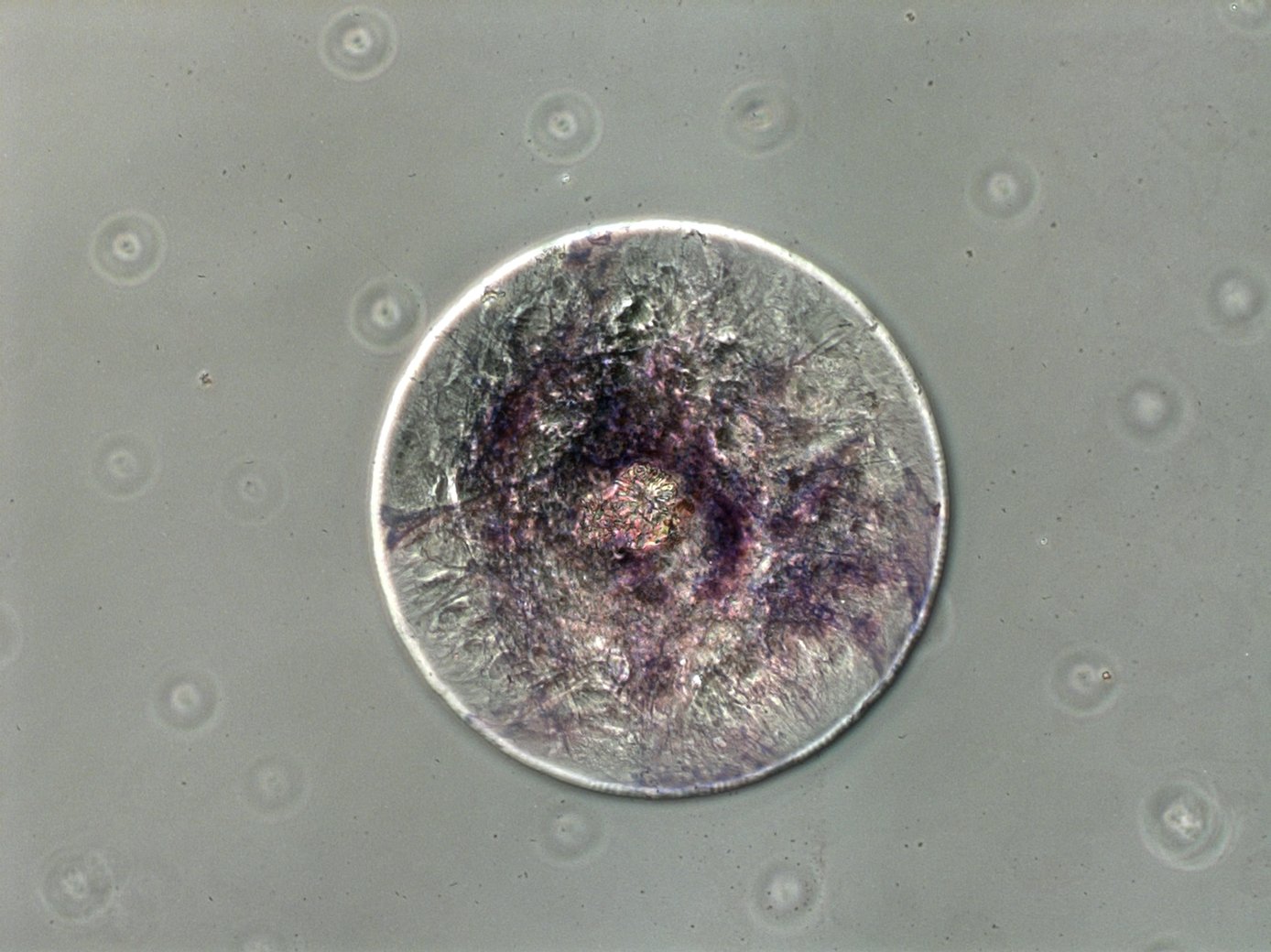

Supplement: S1 File — (ZIP) [file pone.0173647.s002.zip › S1_File/targets/O3_5.jpg]

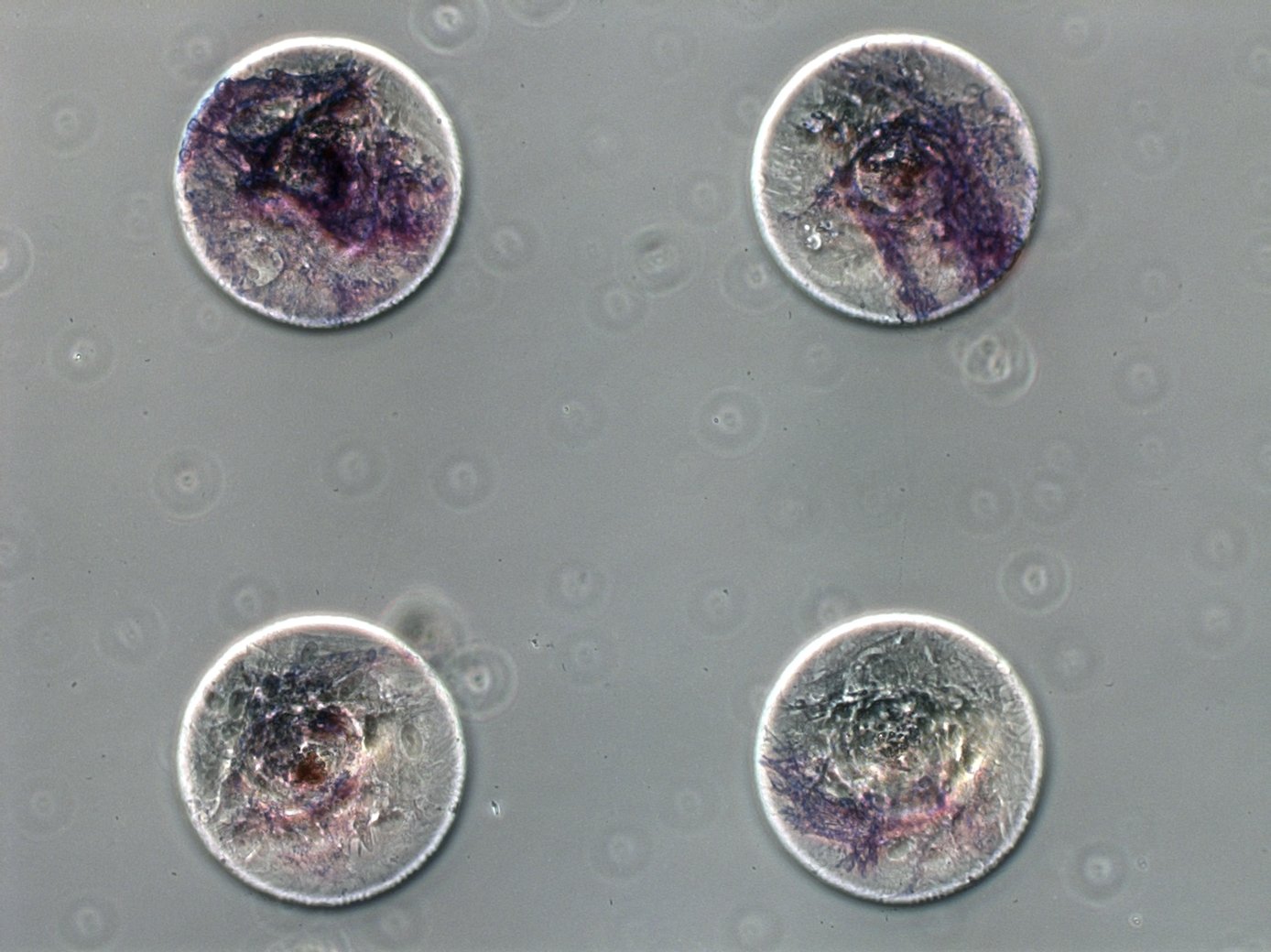

Supplement: S1 File — (ZIP) [file pone.0173647.s002.zip › S1_File/targets/O4_1.jpg]

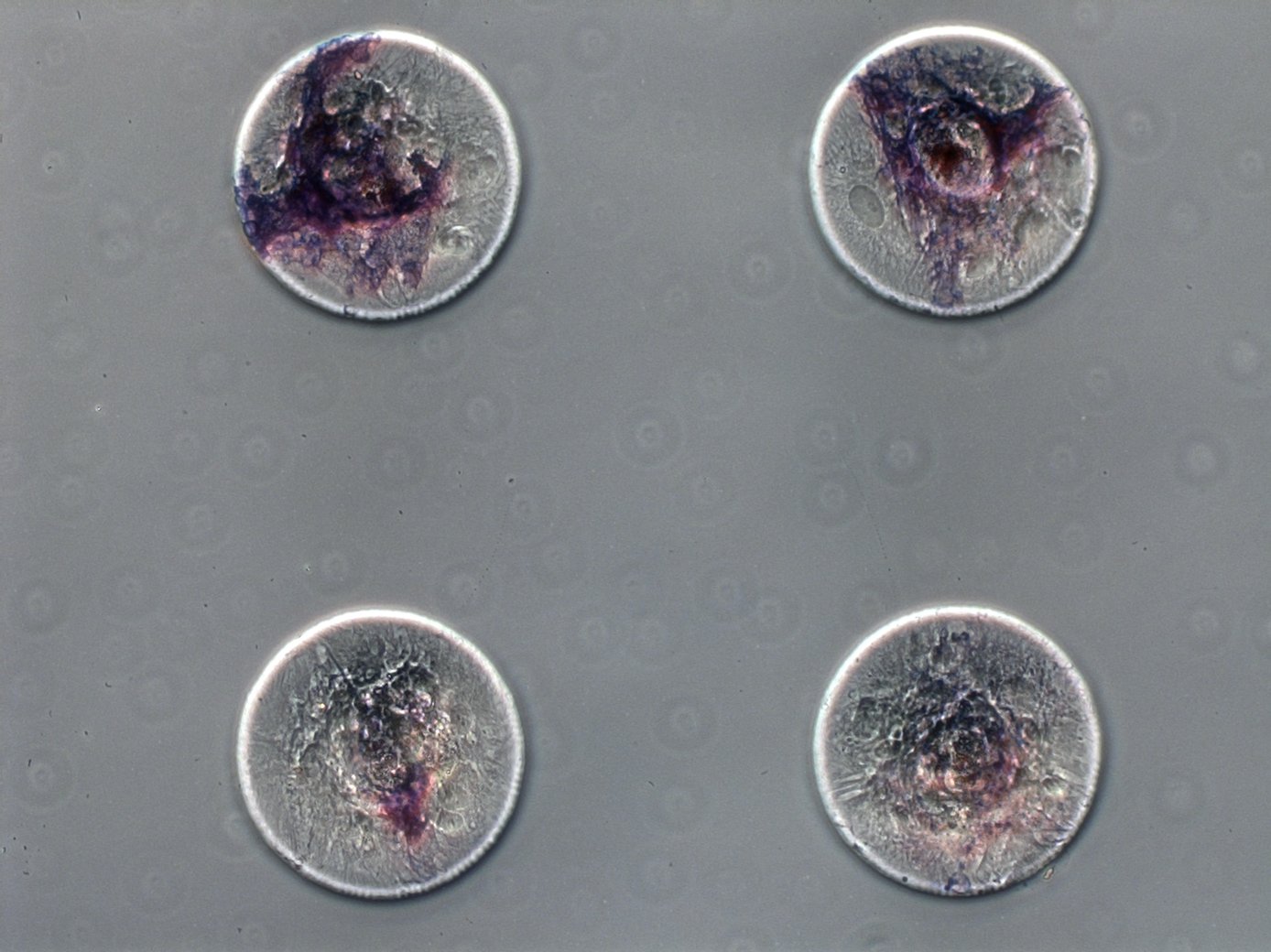

Supplement: S1 File — (ZIP) [file pone.0173647.s002.zip › S1_File/targets/O4_2.jpg]

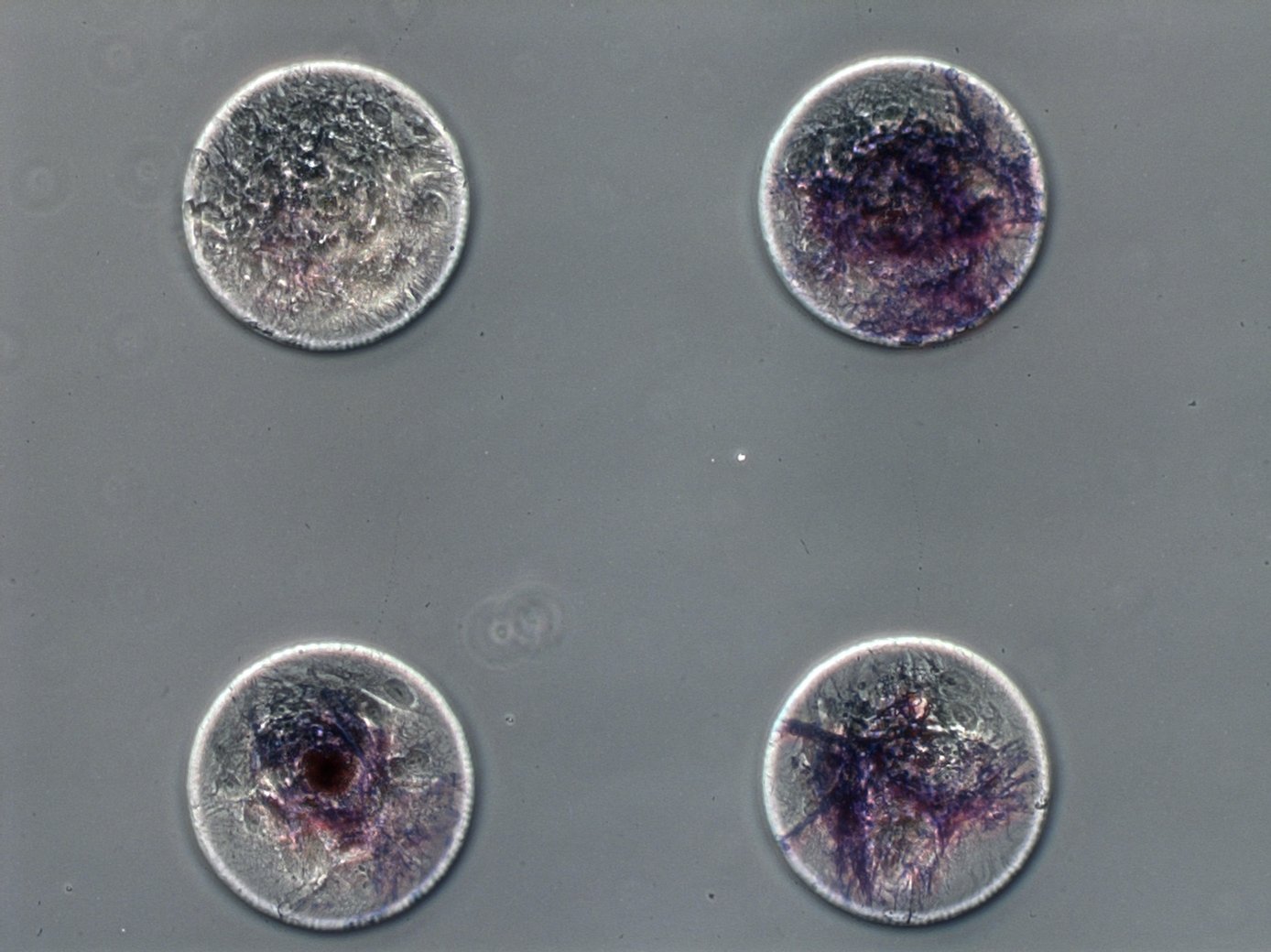

Supplement: S1 File — (ZIP) [file pone.0173647.s002.zip › S1_File/targets/O4_3.jpg]

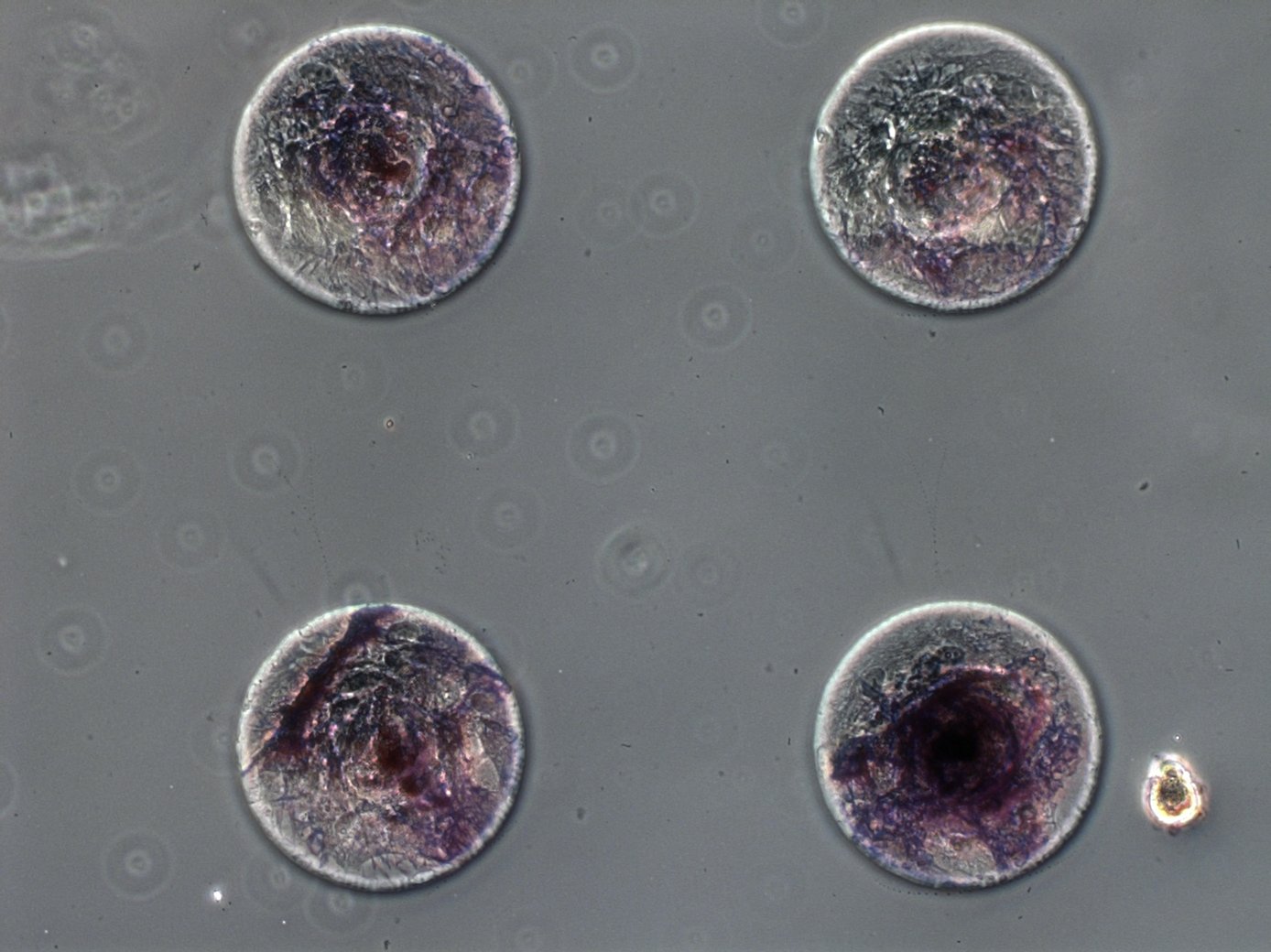

Supplement: S1 File — (ZIP) [file pone.0173647.s002.zip › S1_File/targets/O4_4.jpg]

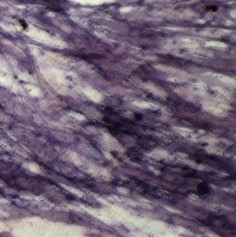

Supplement: S1 File — (ZIP) [file pone.0173647.s002.zip › S1_File/targets/OC_1.jpg]

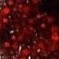

Supplement: S1 File — (ZIP) [file pone.0173647.s002.zip › S1_File/trainingData/A01.jpg]

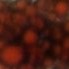

Supplement: S1 File — (ZIP) [file pone.0173647.s002.zip › S1_File/trainingData/A02.jpg]

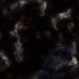

Supplement: S1 File — (ZIP) [file pone.0173647.s002.zip › S1_File/trainingData/A03.jpg]

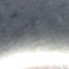

Supplement: S1 File — (ZIP) [file pone.0173647.s002.zip › S1_File/trainingData/B01.jpg]

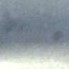

Supplement: S1 File — (ZIP) [file pone.0173647.s002.zip › S1_File/trainingData/B02.jpg]

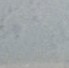

Supplement: S1 File — (ZIP) [file pone.0173647.s002.zip › S1_File/trainingData/B03.jpg]

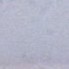

Supplement: S1 File — (ZIP) [file pone.0173647.s002.zip › S1_File/trainingData/B04.jpg]

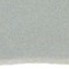

Supplement: S1 File — (ZIP) [file pone.0173647.s002.zip › S1_File/trainingData/B05.jpg]

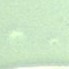

Supplement: S1 File — (ZIP) [file pone.0173647.s002.zip › S1_File/trainingData/B06.jpg]

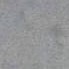

Supplement: S1 File — (ZIP) [file pone.0173647.s002.zip › S1_File/trainingData/B07.jpg]

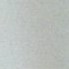

Supplement: S1 File — (ZIP) [file pone.0173647.s002.zip › S1_File/trainingData/B08.jpg]

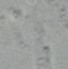

Supplement: S1 File — (ZIP) [file pone.0173647.s002.zip › S1_File/trainingData/B09.jpg]

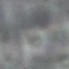

Supplement: S1 File — (ZIP) [file pone.0173647.s002.zip › S1_File/trainingData/B10.jpg]

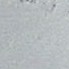

Supplement: S1 File — (ZIP) [file pone.0173647.s002.zip › S1_File/trainingData/B11.jpg]

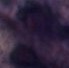

Supplement: S1 File — (ZIP) [file pone.0173647.s002.zip › S1_File/trainingData/O01.jpg]

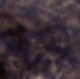

Supplement: S1 File — (ZIP) [file pone.0173647.s002.zip › S1_File/trainingData/O02.jpg]

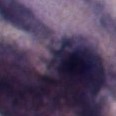

Supplement: S1 File — (ZIP) [file pone.0173647.s002.zip › S1_File/trainingData/O03.jpg]

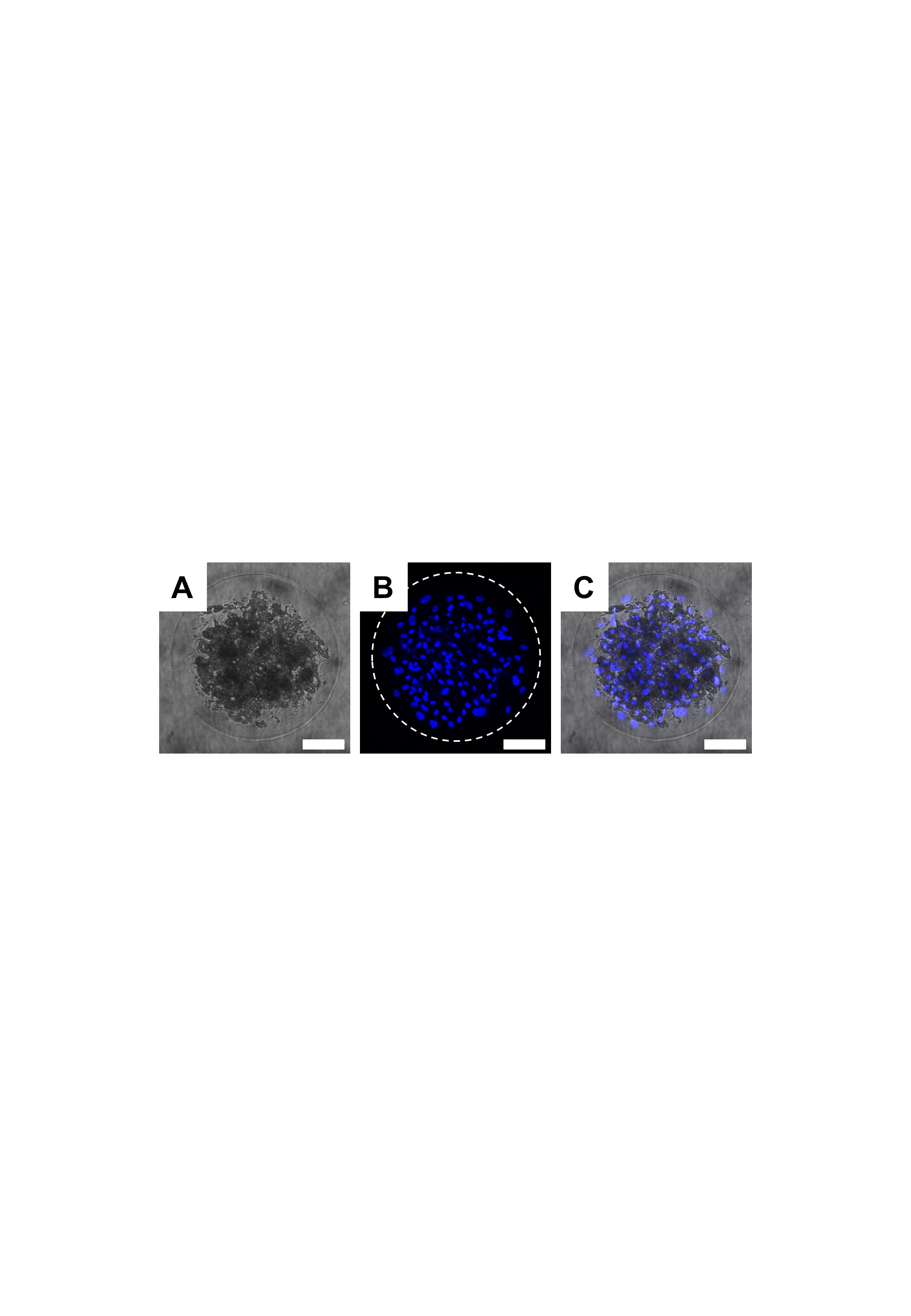

Supplement: S1 Fig — (A) Phase-contrast micrograph. (B) Fluorescent micrograph. Nuclei were stained with blue-fluorescent dye. White dashed circle indicates the boundary of confinement. (C) Merged image between (A) phase-contrast and (B) fluorescent micrographs. White scale bars indicate 100 μm. (TIF) [file pone.0173647.s004.TIF]

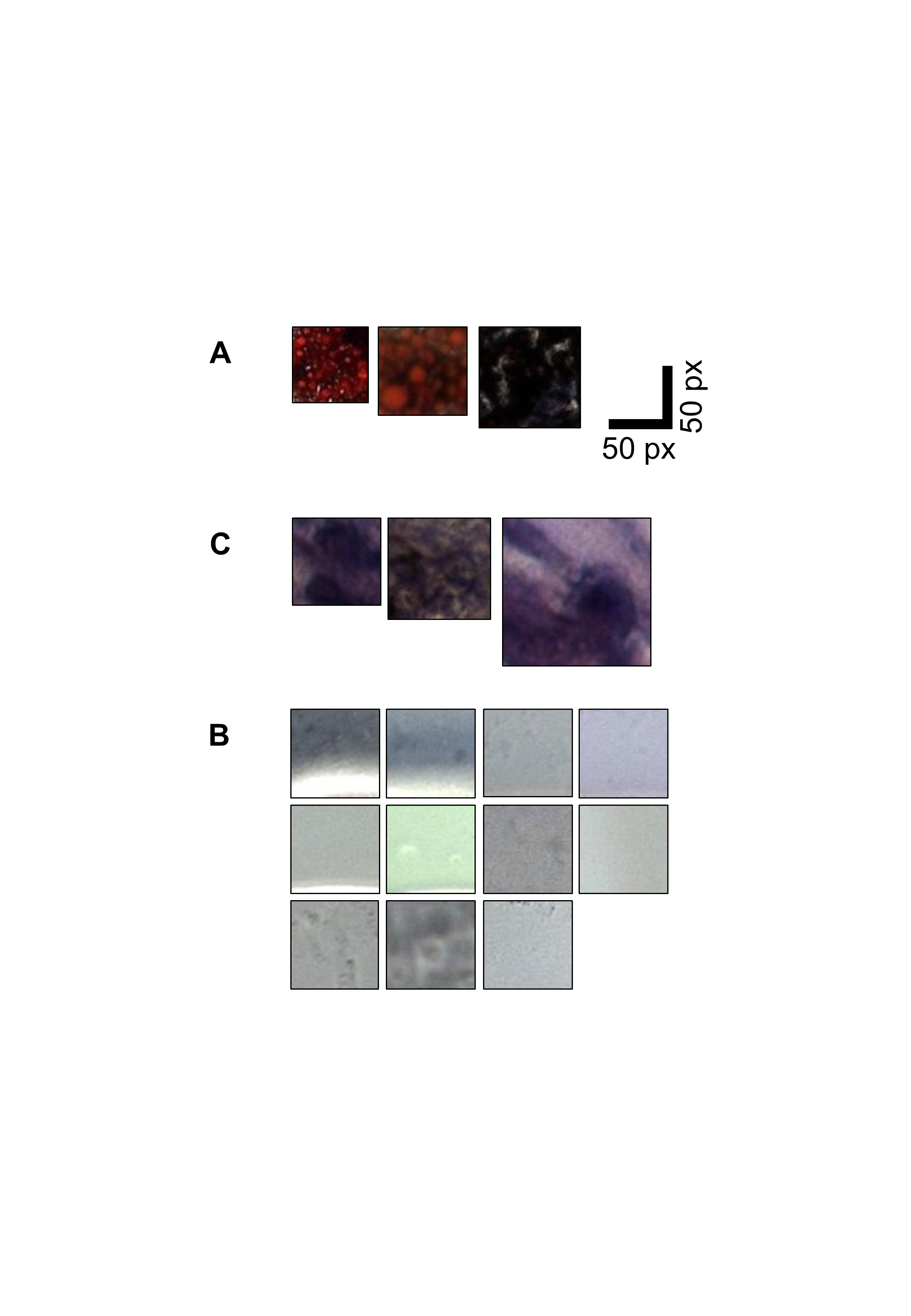

Supplement: S2 Fig — Microphotographs (A-C) show training data for OilRed O positive (Adipogenic), FastBlue positive (Osteogenic), and negative (Undifferentiated or background) areas, respectively. Black angle symbol indicates 50-pixel length in both horizontal and vertical directions. (TIF) [file pone.0173647.s005.TIF]

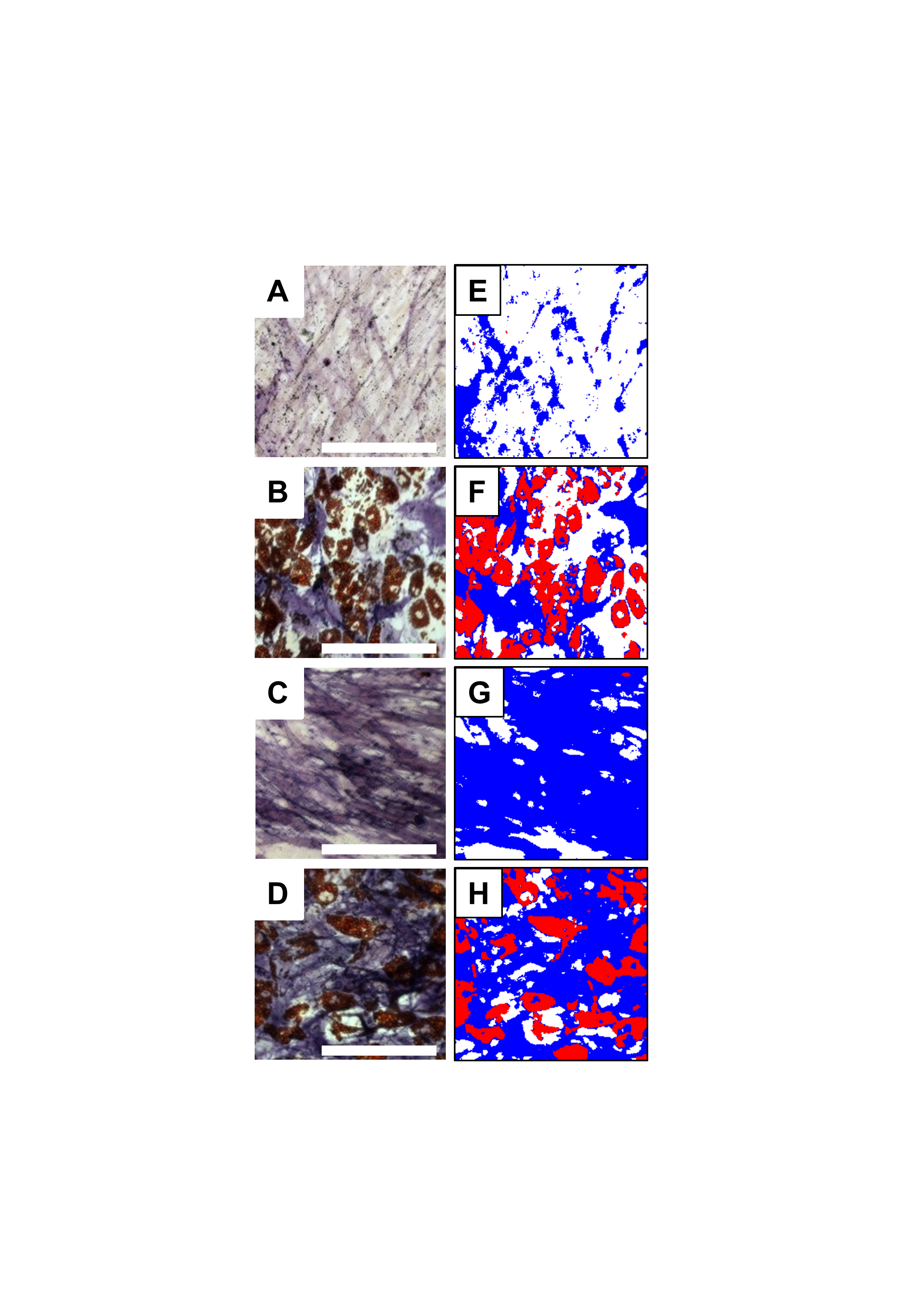

Supplement: S3 Fig — Microphotographs (A-D) show the stained surfaces of hMSCs cultured in growth, adipogenesis, osteogenesis, and adipgenesis-osteogenesis mixture media, resulting in classified images (E-H), respectively. White bar indicates 200 μm. (TIF) [file pone.0173647.s006.TIF]

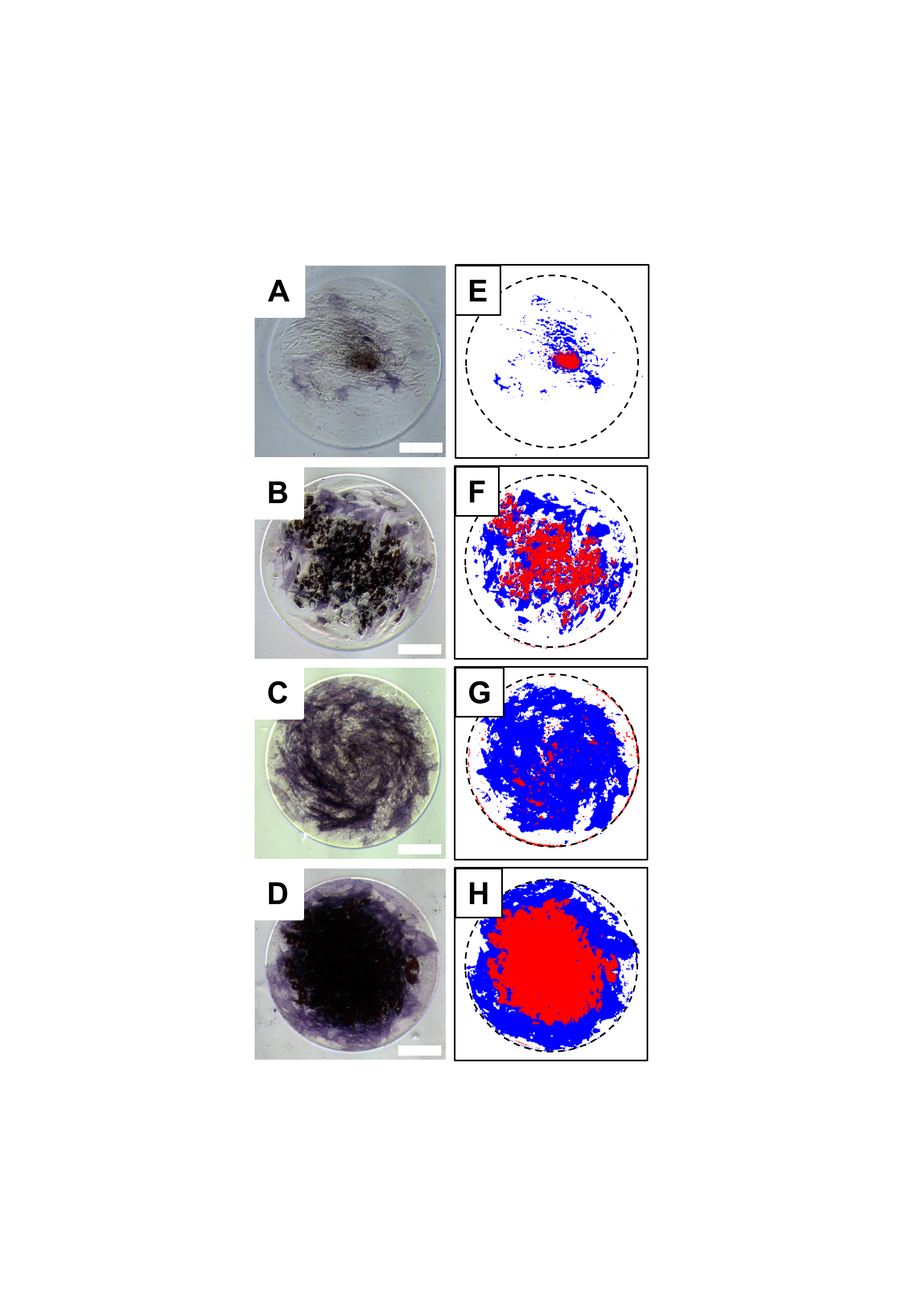

Supplement: S4 Fig — Microphotographs (A-D) show the stained surfaces of hMSCs cultured in growth, adipogenesis, osteogenesis, and adipgenesis-osteogenesis mixed media, respectively, resulting in classified images (E-H). White bar indicates 200 μm. (TIF) [file pone.0173647.s007.TIF]

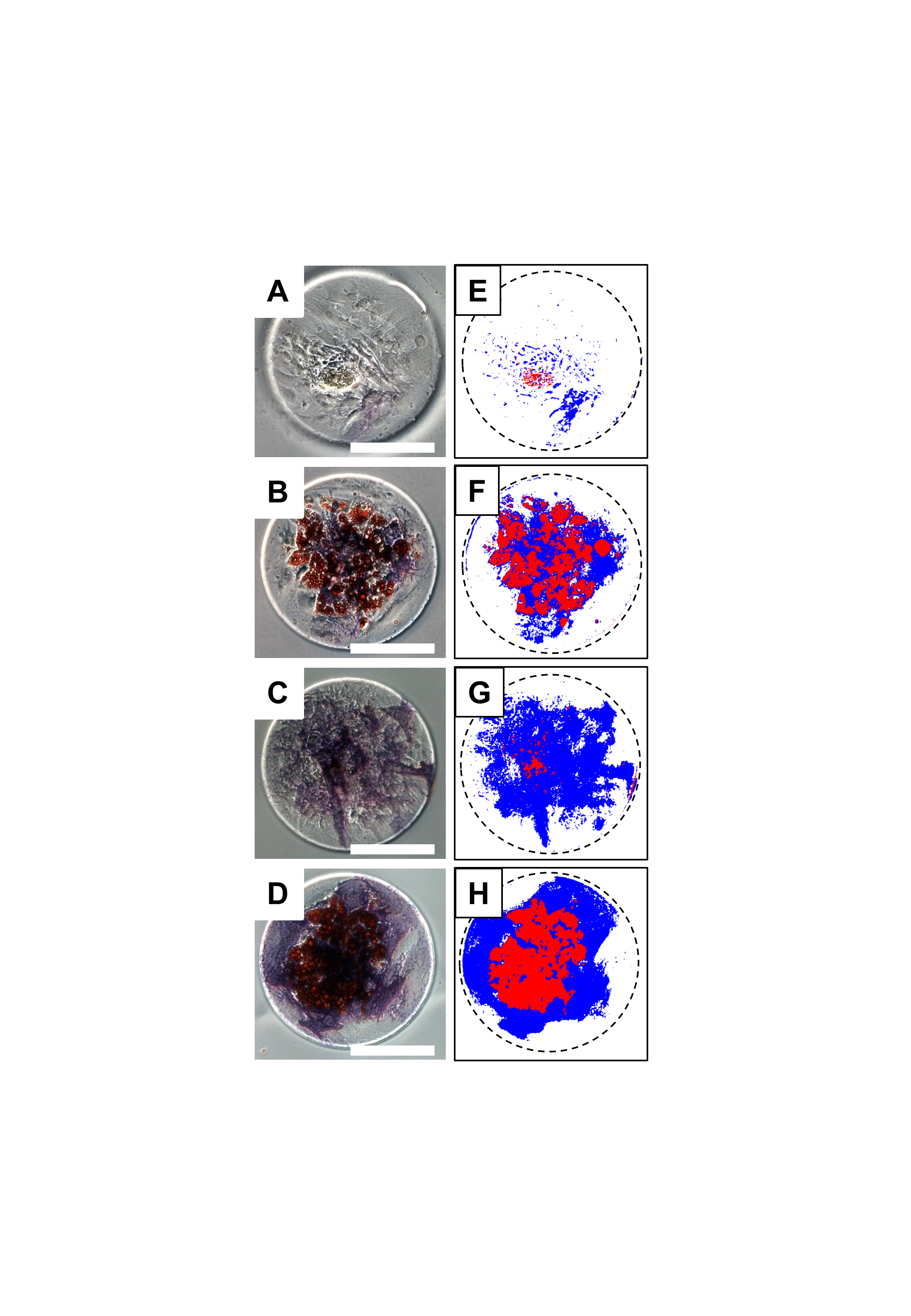

Supplement: S5 Fig — Microphotographs (A-D) show the stained surfaces of hMSCs cultured in growth, adipogenesis, osteogenesis, and adipgenesis-osteogenesis mixed media, respectively, resulting in classified images (E-H). White bar indicates 200 μm. (TIF) [file pone.0173647.s008.TIF]

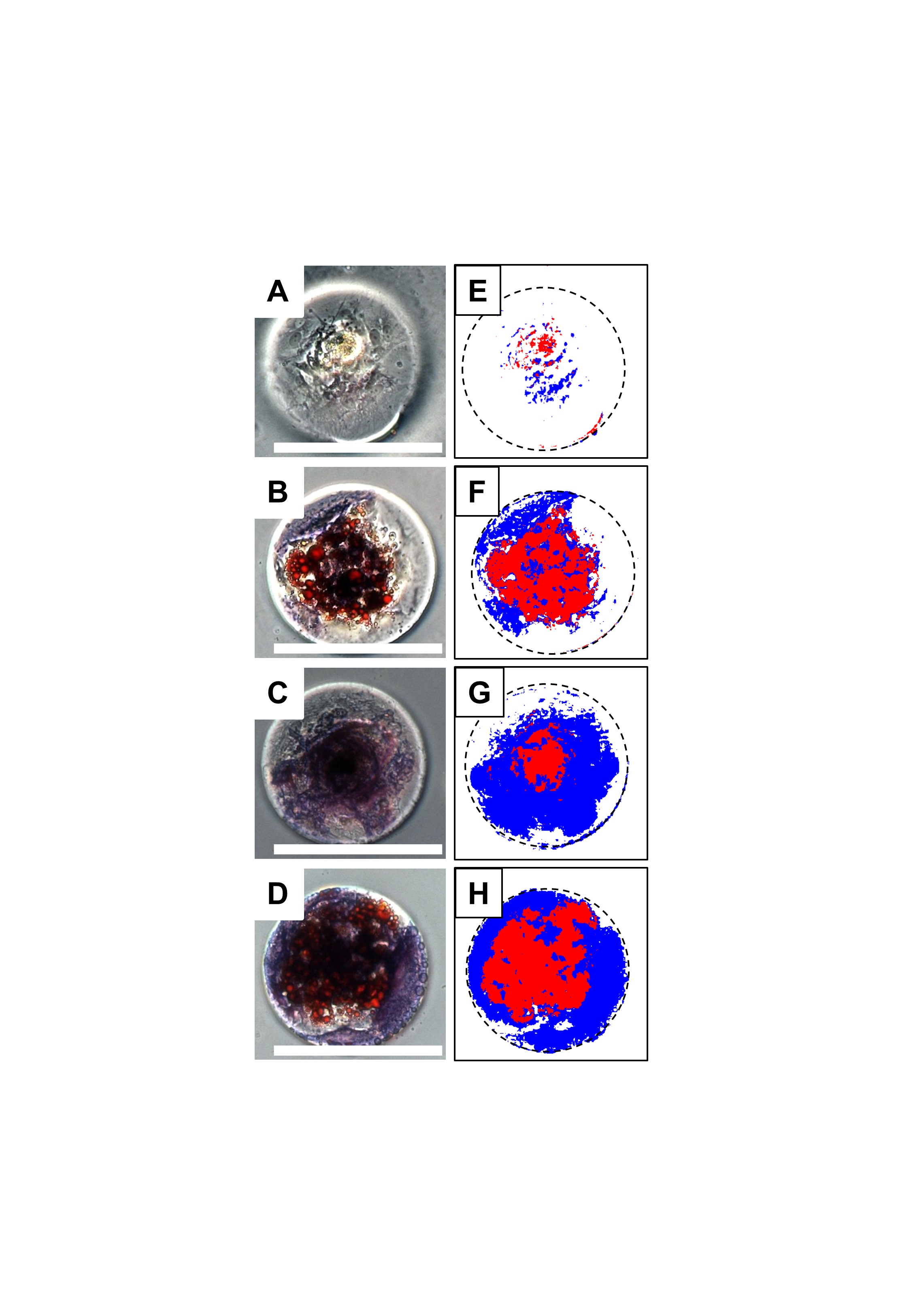

Supplement: S6 Fig — Microphotographs (A-D) show the stained surfaces of hMSCs cultured in growth, adipogenesis, osteogenesis, and adipgenesis-osteogenesis mixed media, respectively, resulting in classified images (E-H). White bar indicates 200 μm. (TIF) [file pone.0173647.s009.TIF]
