# Supplementary figures and images for: Simple agarose micro-confinement array and machine-learning-based classification for analyzing the patterned differentiation of mesenchymal stem cells (part 1 of 2)
Source: PLoS One. 2017 Apr 5;12(4):e0173647. doi: 10.1371/journal.pone.0173647 (PMC5381775; doi:10.1371/journal.pone.0173647)

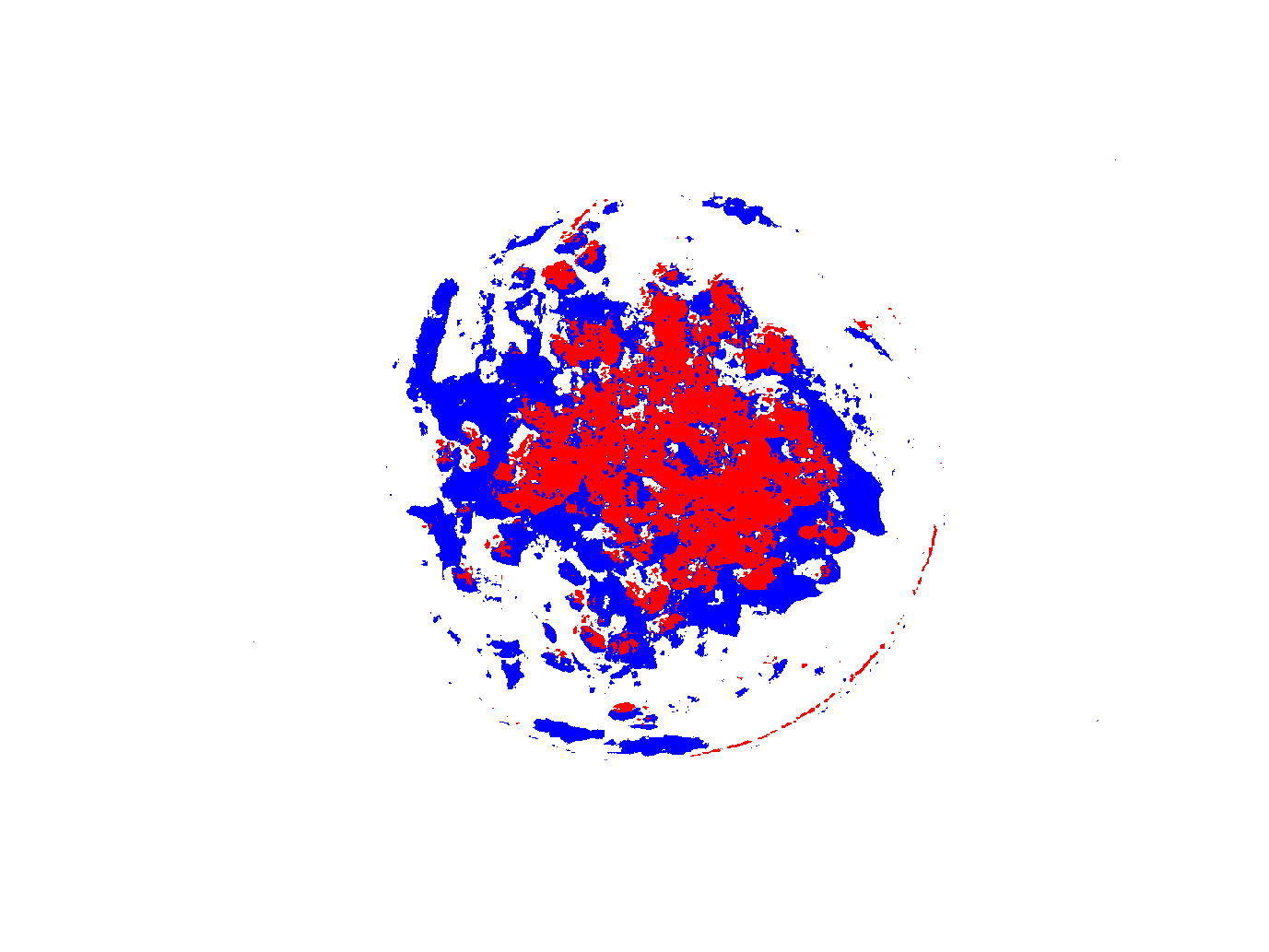

Supplement: S1 File — (ZIP) [file pone.0173647.s002.zip › S1_File/20160830_225416/A2_1.jpg.tiff]

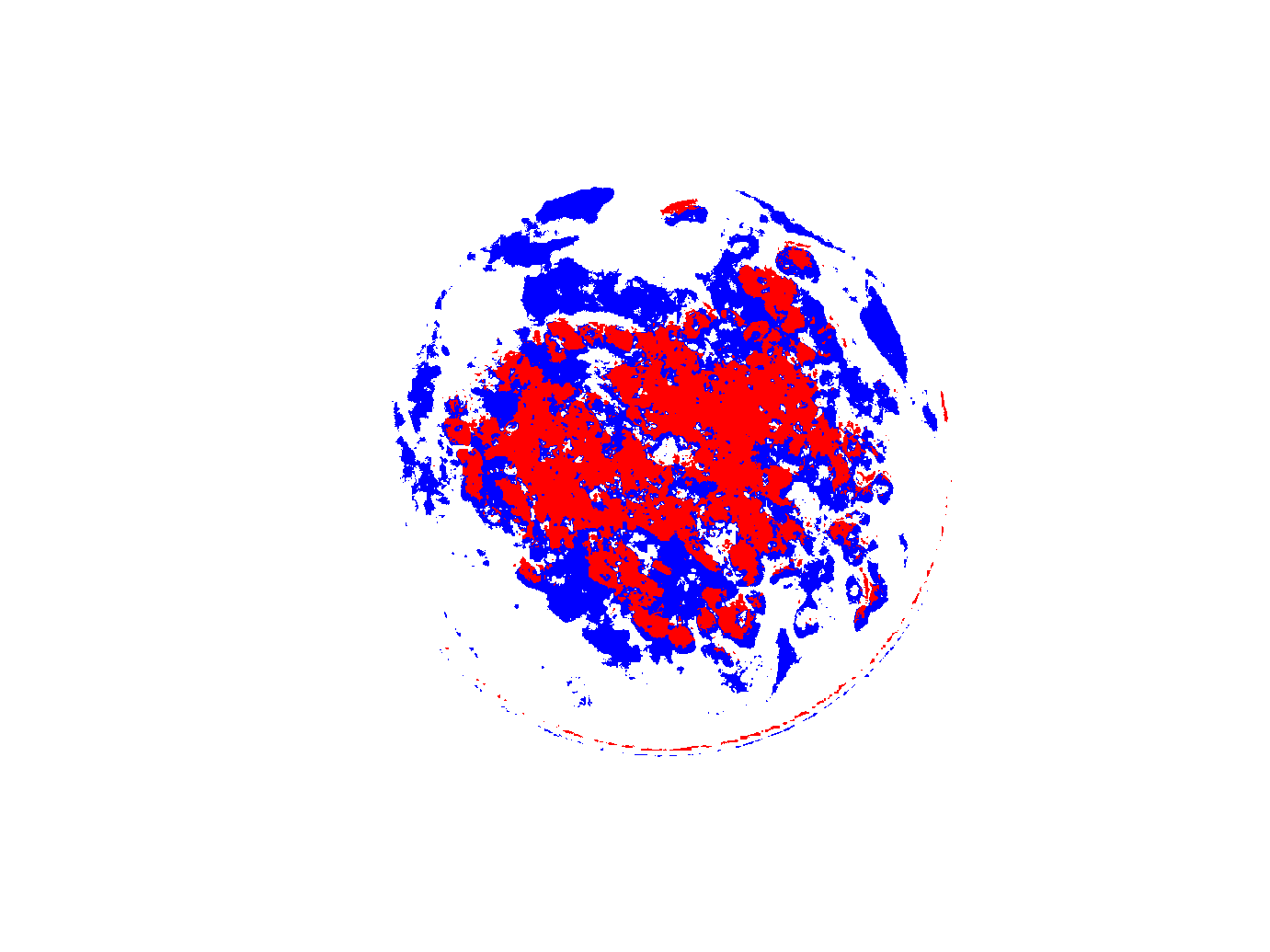

Supplement: S1 File — (ZIP) [file pone.0173647.s002.zip › S1_File/20160830_225416/A2_2.jpg.tiff]

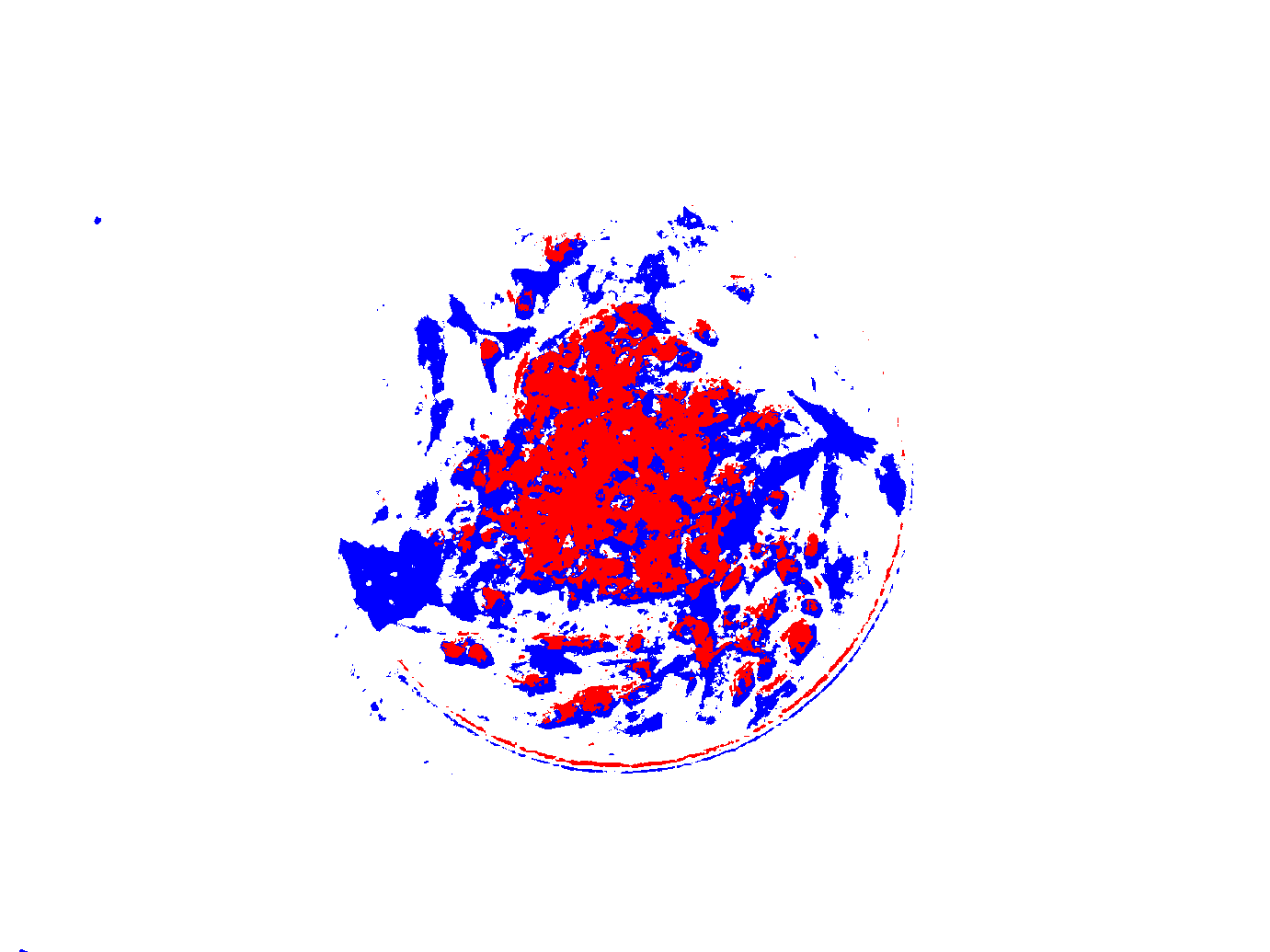

Supplement: S1 File — (ZIP) [file pone.0173647.s002.zip › S1_File/20160830_225416/A2_3.jpg.tiff]

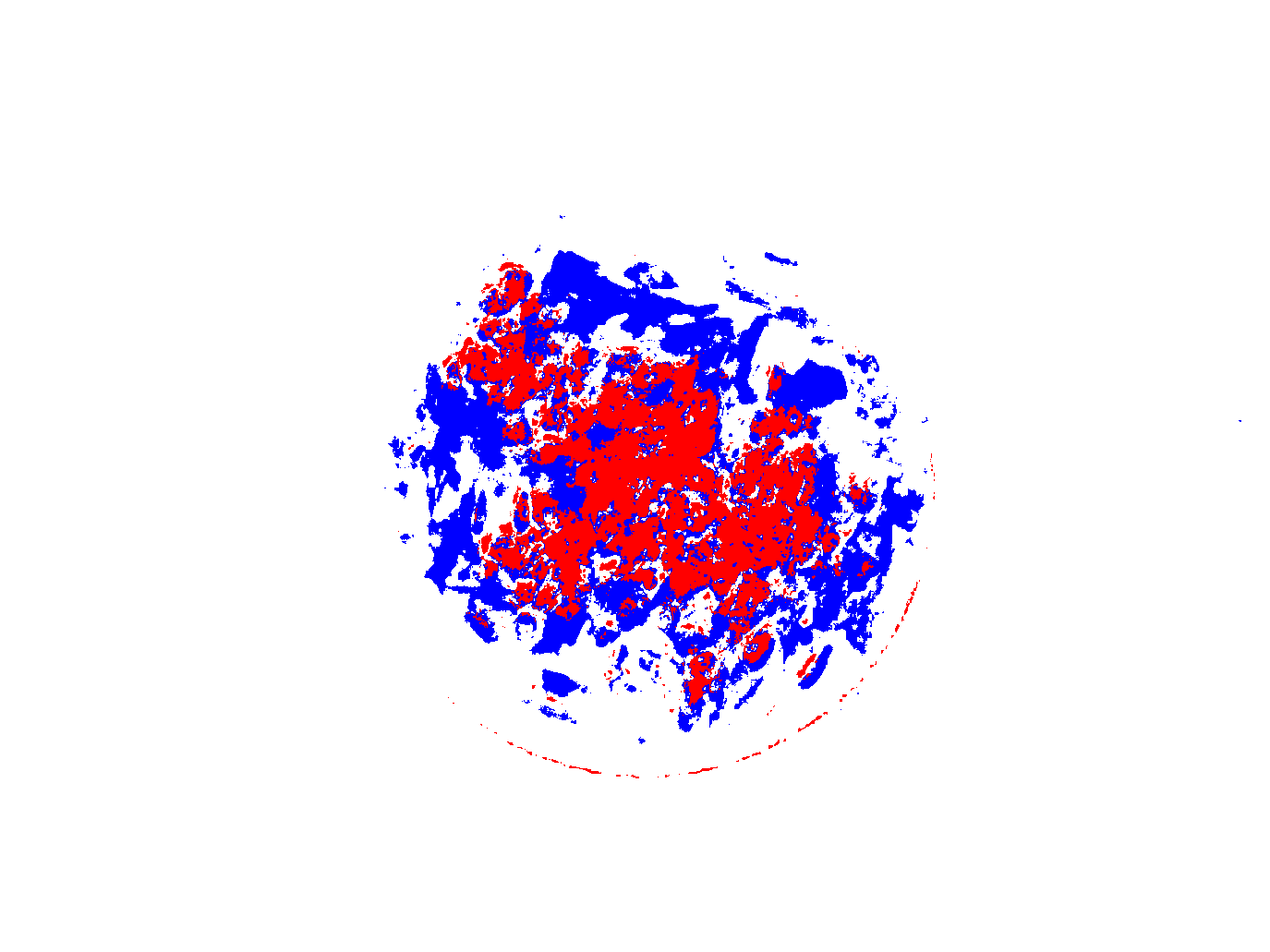

Supplement: S1 File — (ZIP) [file pone.0173647.s002.zip › S1_File/20160830_225416/A2_4.jpg.tiff]

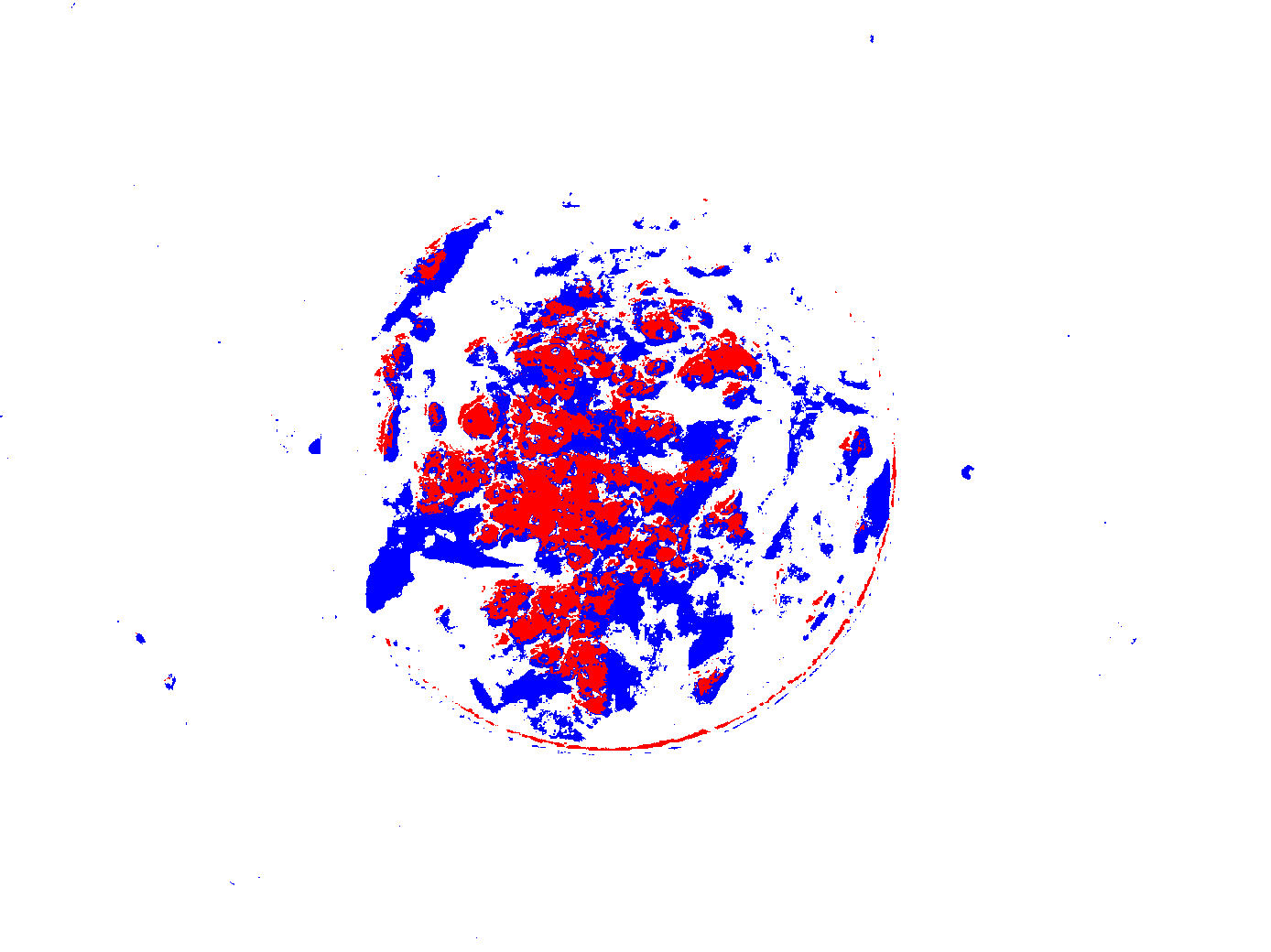

Supplement: S1 File — (ZIP) [file pone.0173647.s002.zip › S1_File/20160830_225416/A2_5.jpg.tiff]

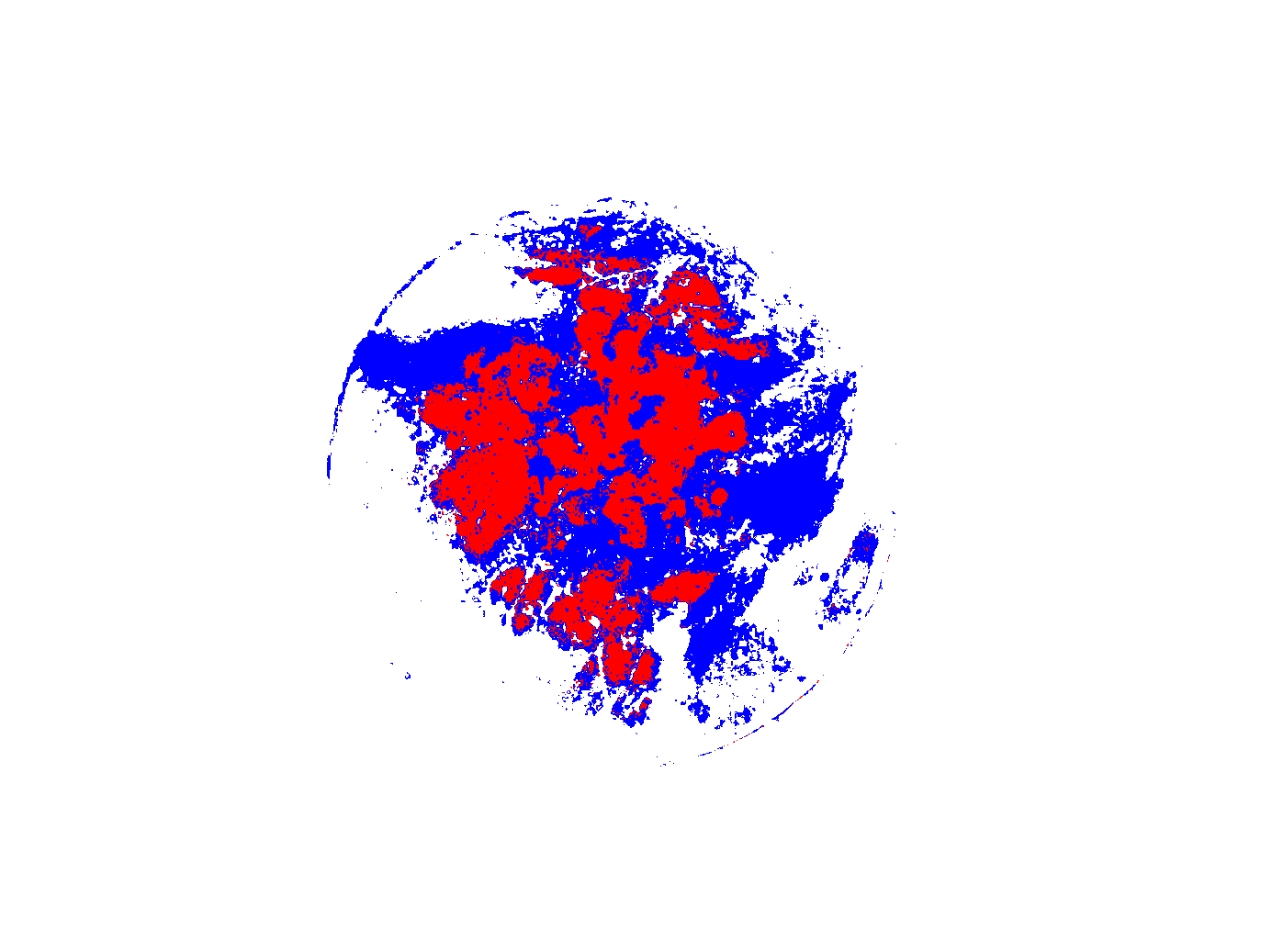

Supplement: S1 File — (ZIP) [file pone.0173647.s002.zip › S1_File/20160830_225416/A3_1.jpg.tiff]

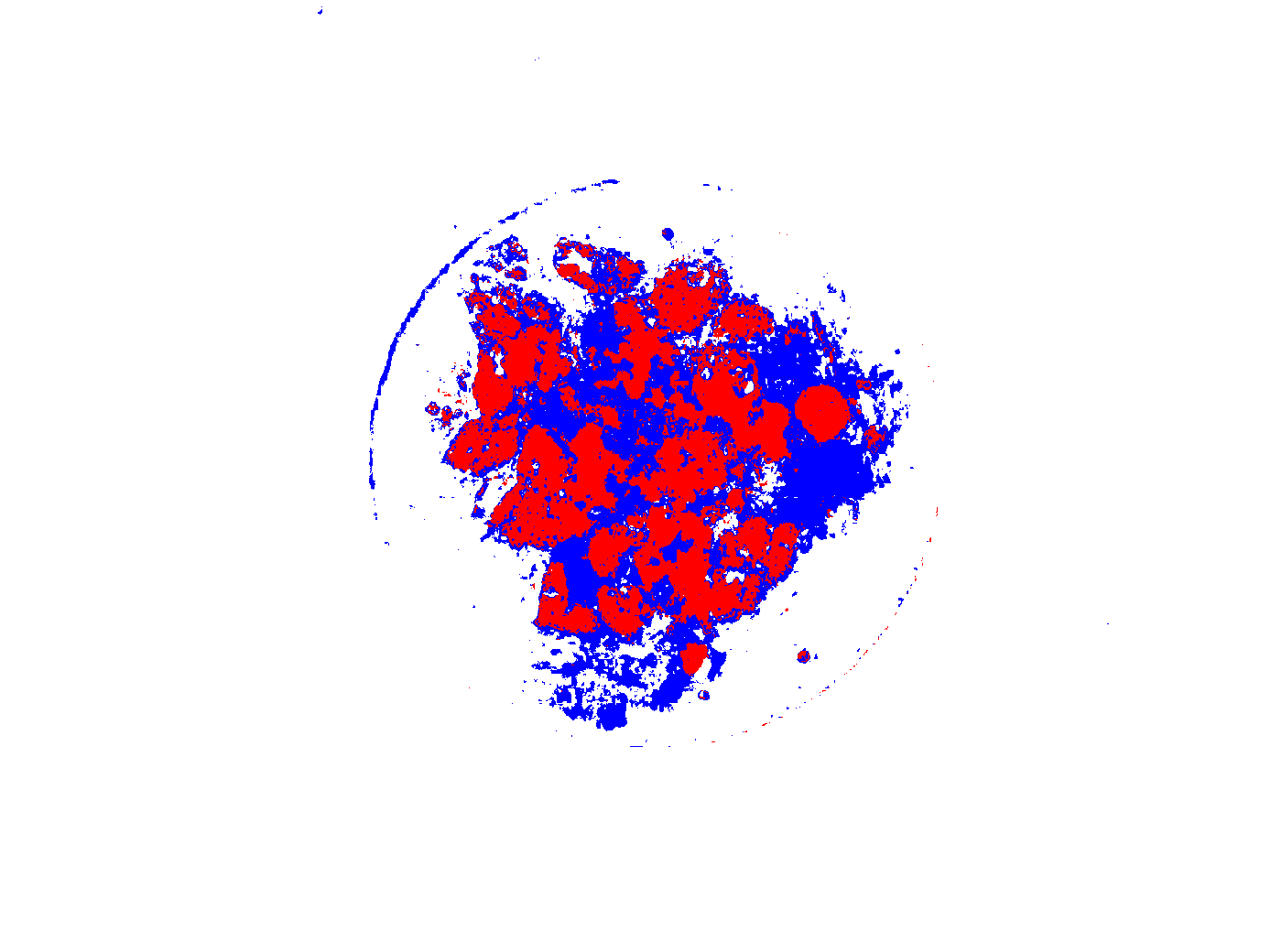

Supplement: S1 File — (ZIP) [file pone.0173647.s002.zip › S1_File/20160830_225416/A3_2.jpg.tiff]

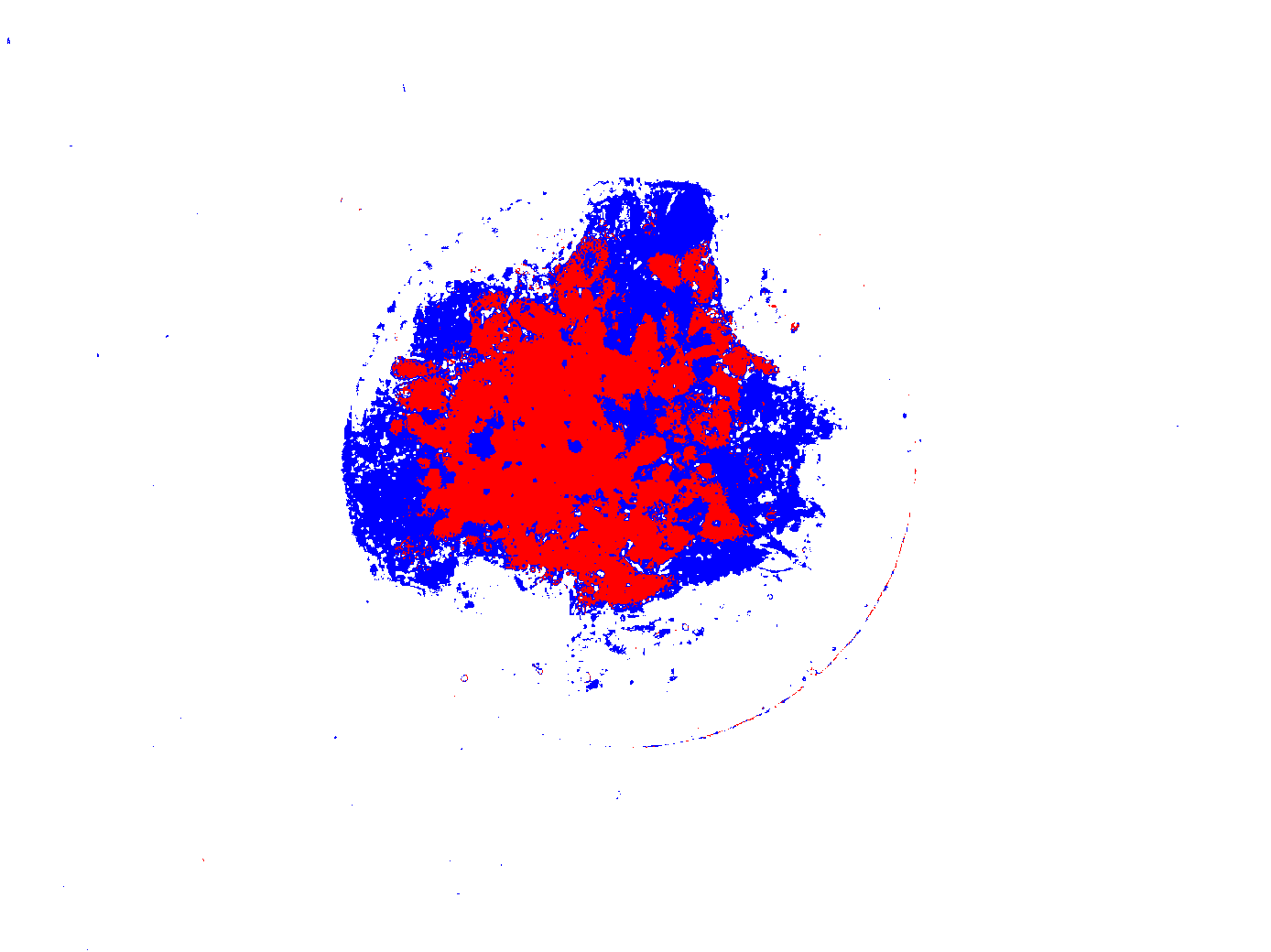

Supplement: S1 File — (ZIP) [file pone.0173647.s002.zip › S1_File/20160830_225416/A3_3.jpg.tiff]

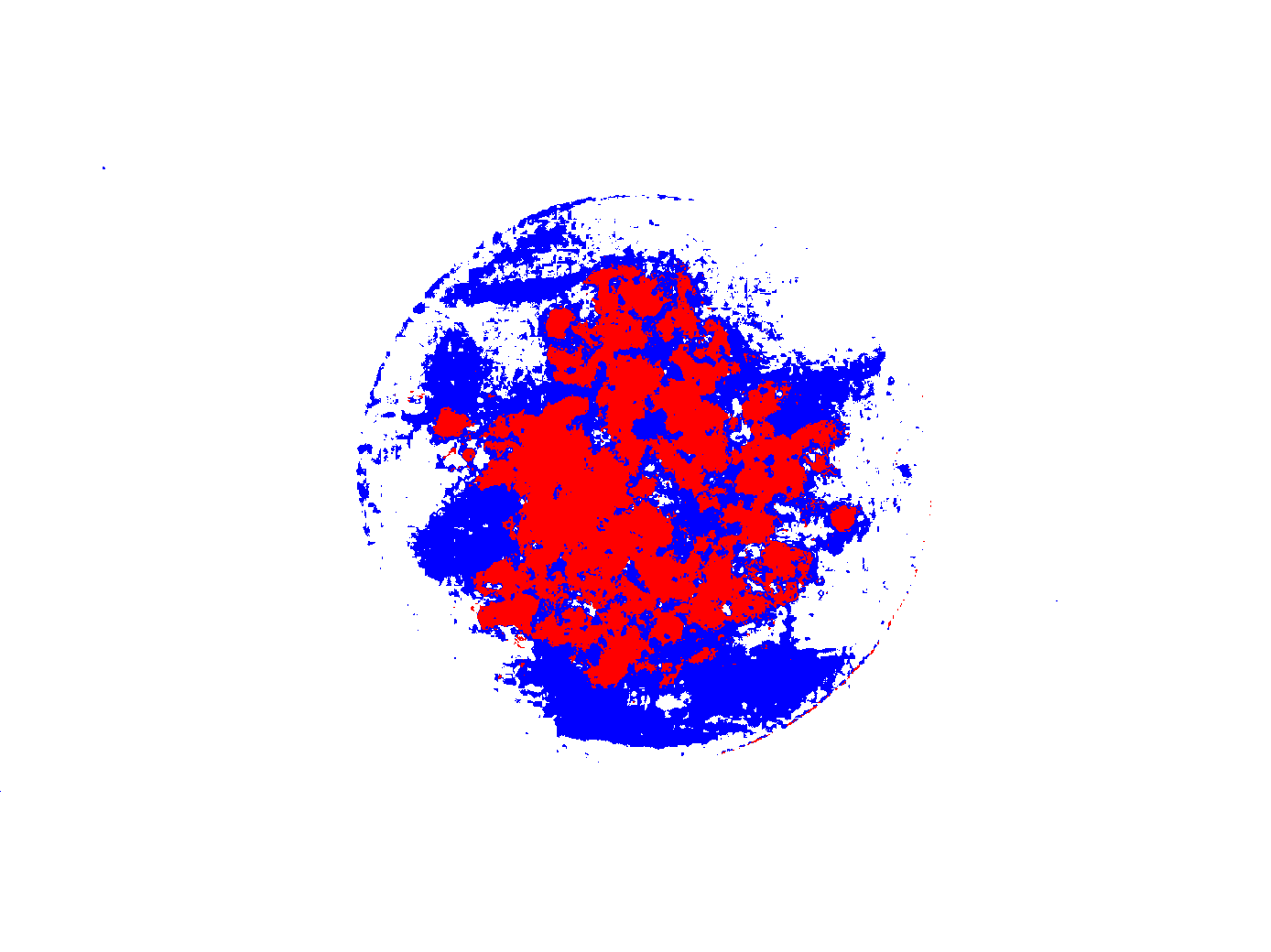

Supplement: S1 File — (ZIP) [file pone.0173647.s002.zip › S1_File/20160830_225416/A3_4.jpg.tiff]

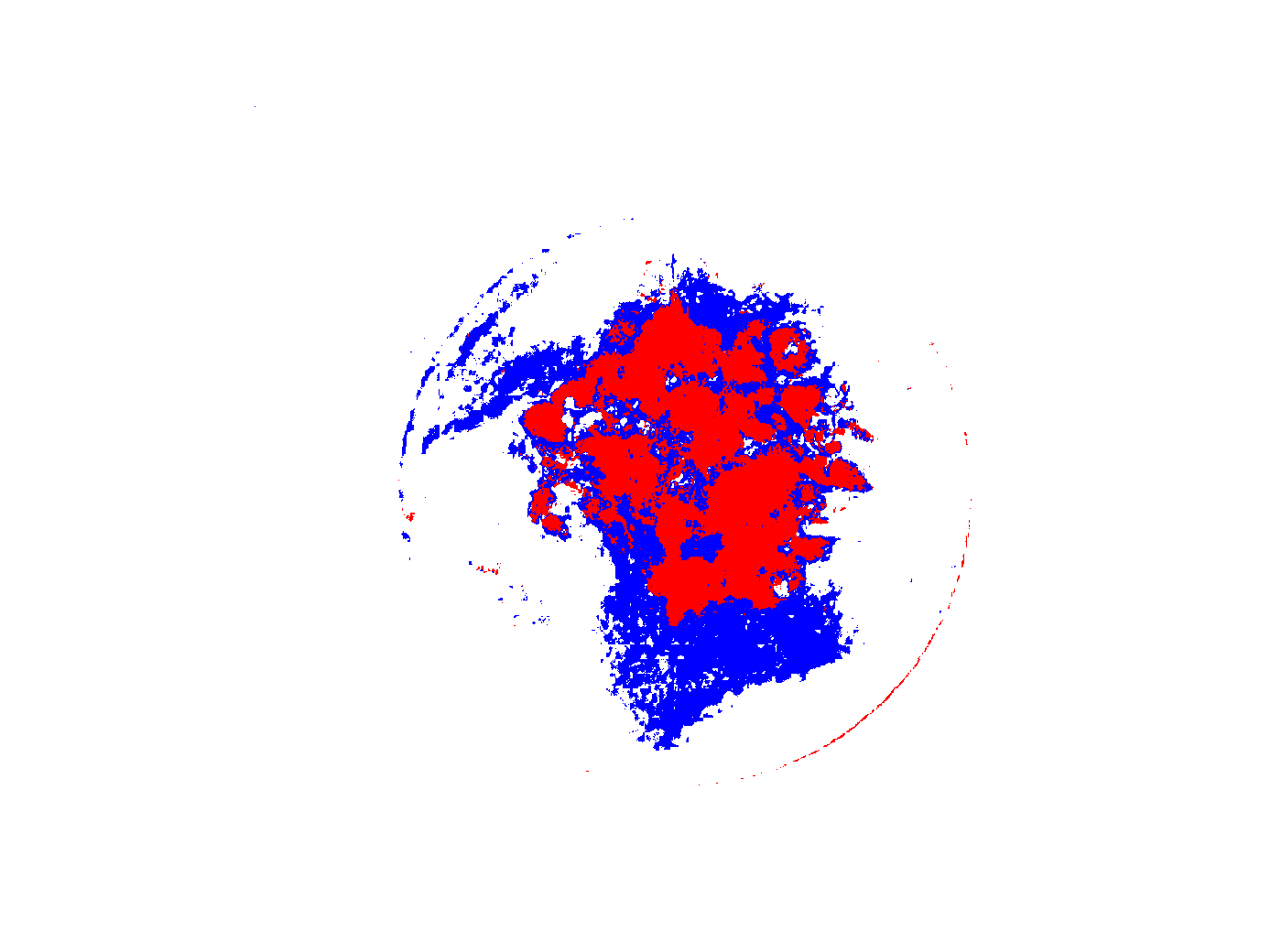

Supplement: S1 File — (ZIP) [file pone.0173647.s002.zip › S1_File/20160830_225416/A3_5.jpg.tiff]

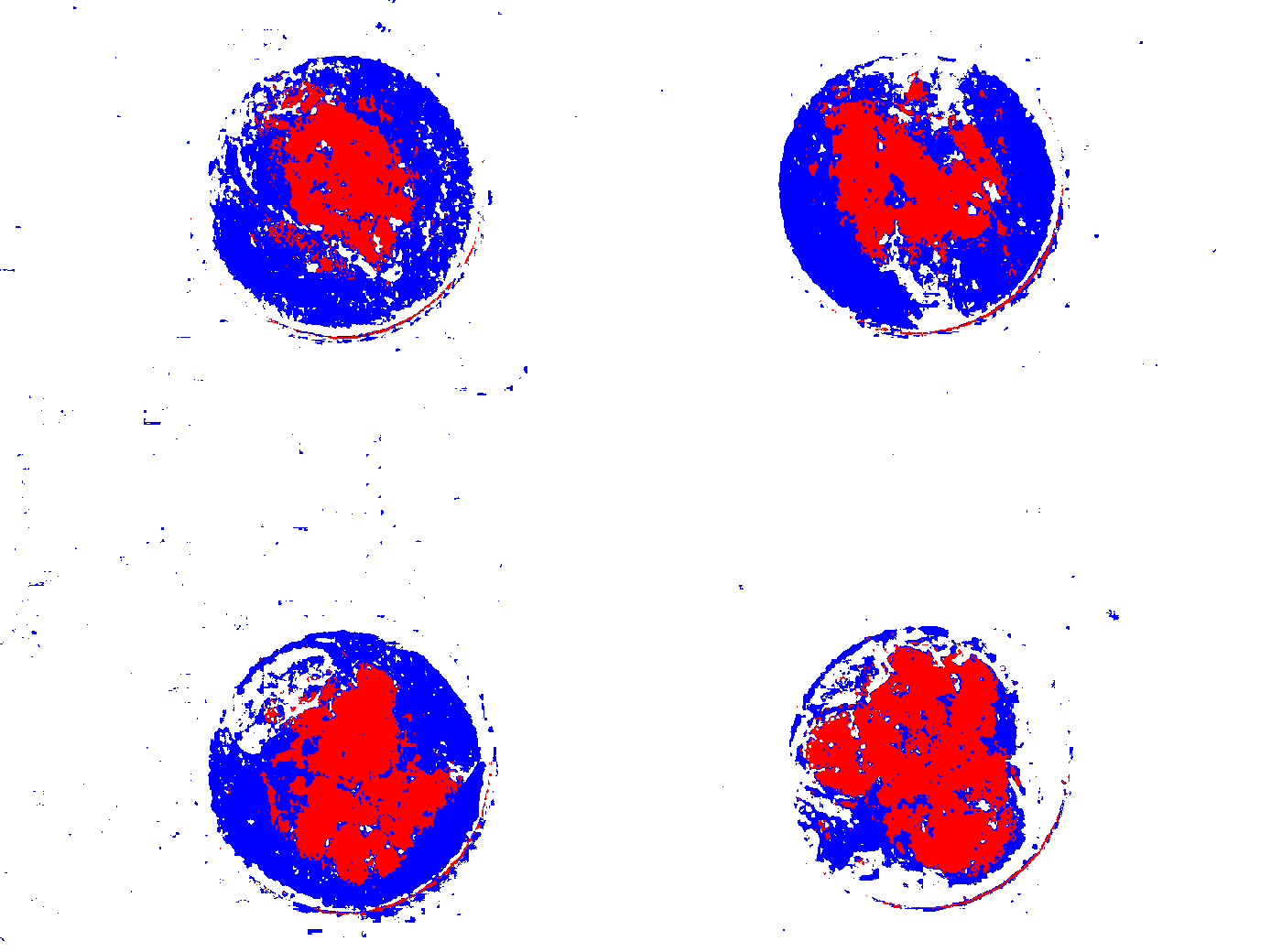

Supplement: S1 File — (ZIP) [file pone.0173647.s002.zip › S1_File/20160830_225416/A4_1.jpg.tiff]

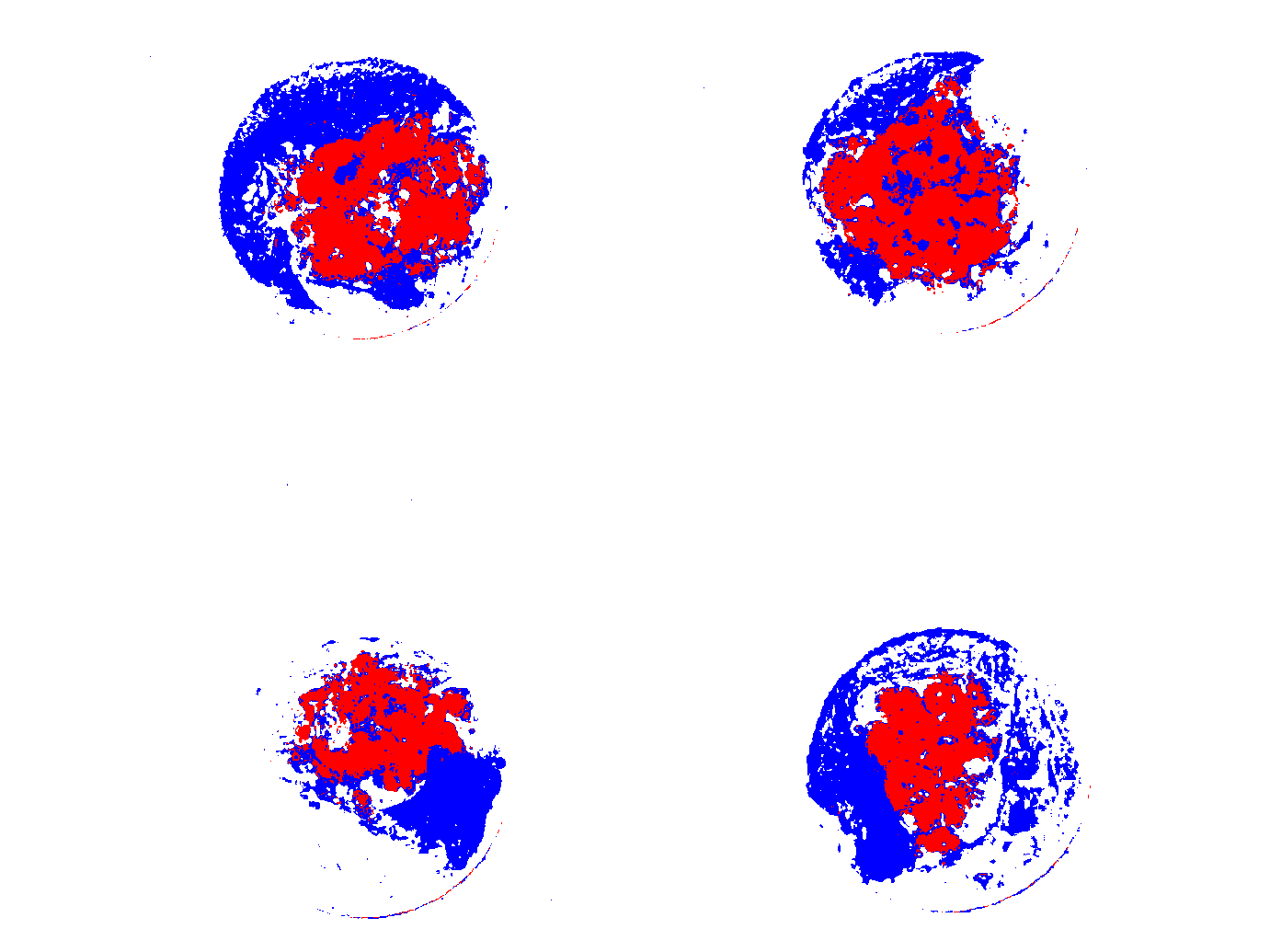

Supplement: S1 File — (ZIP) [file pone.0173647.s002.zip › S1_File/20160830_225416/A4_2.jpg.tiff]

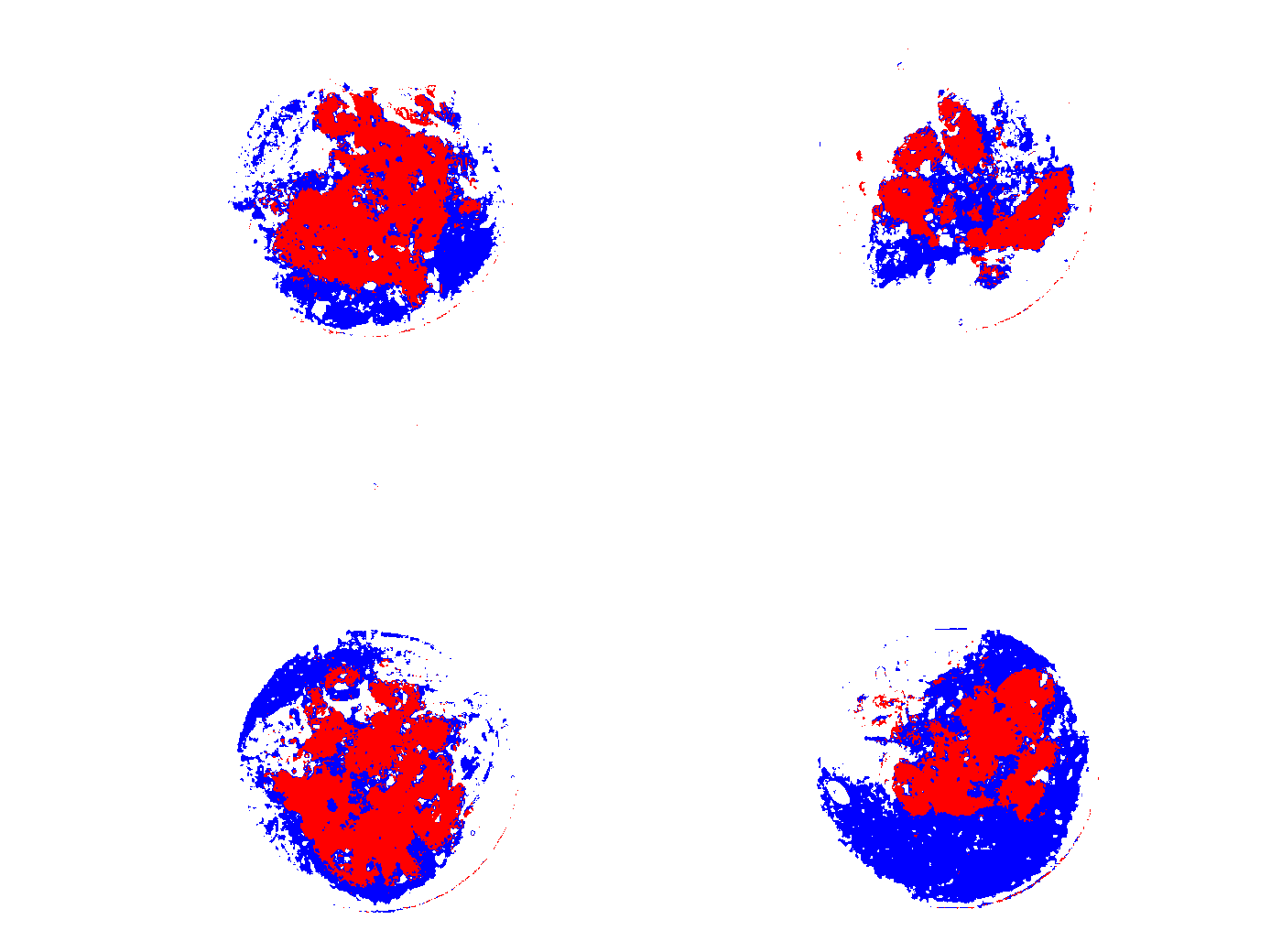

Supplement: S1 File — (ZIP) [file pone.0173647.s002.zip › S1_File/20160830_225416/A4_3.jpg.tiff]

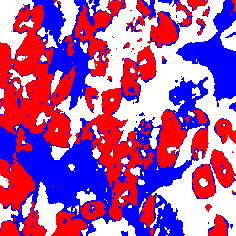

Supplement: S1 File — (ZIP) [file pone.0173647.s002.zip › S1_File/20160830_225416/AC_1.jpg.tiff]

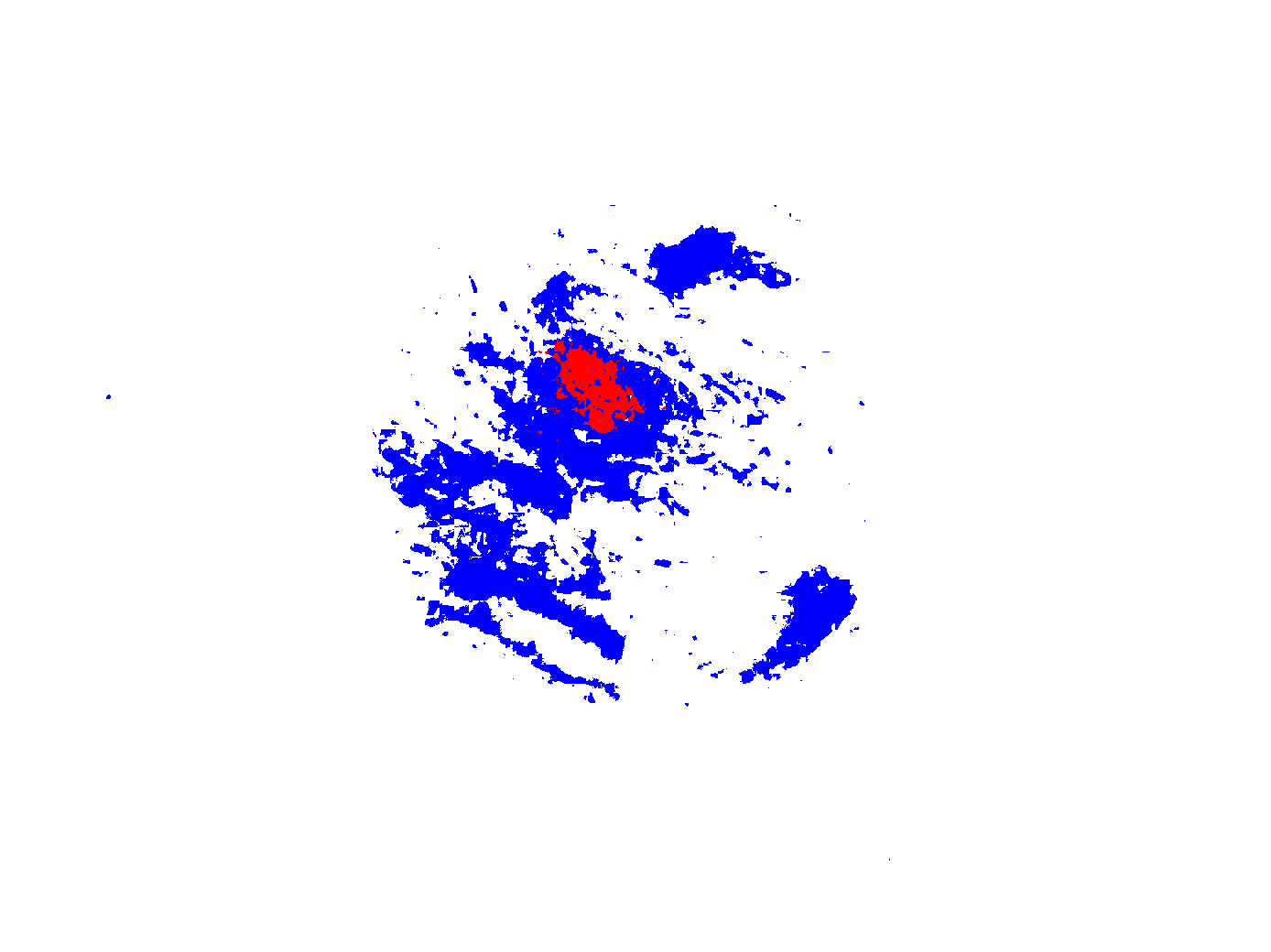

Supplement: S1 File — (ZIP) [file pone.0173647.s002.zip › S1_File/20160830_225416/G2_1.jpg.tiff]

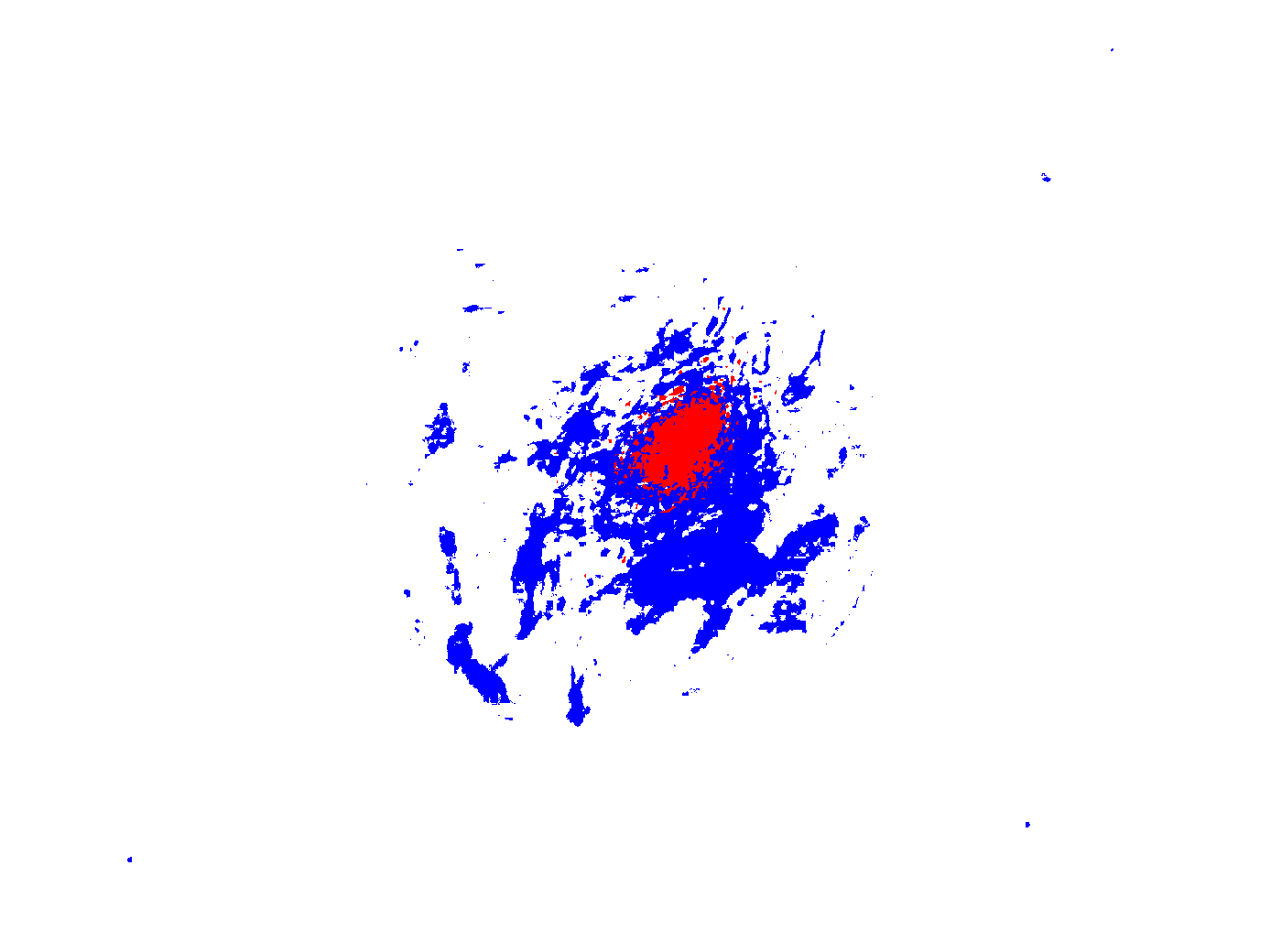

Supplement: S1 File — (ZIP) [file pone.0173647.s002.zip › S1_File/20160830_225416/G2_2.jpg.tiff]

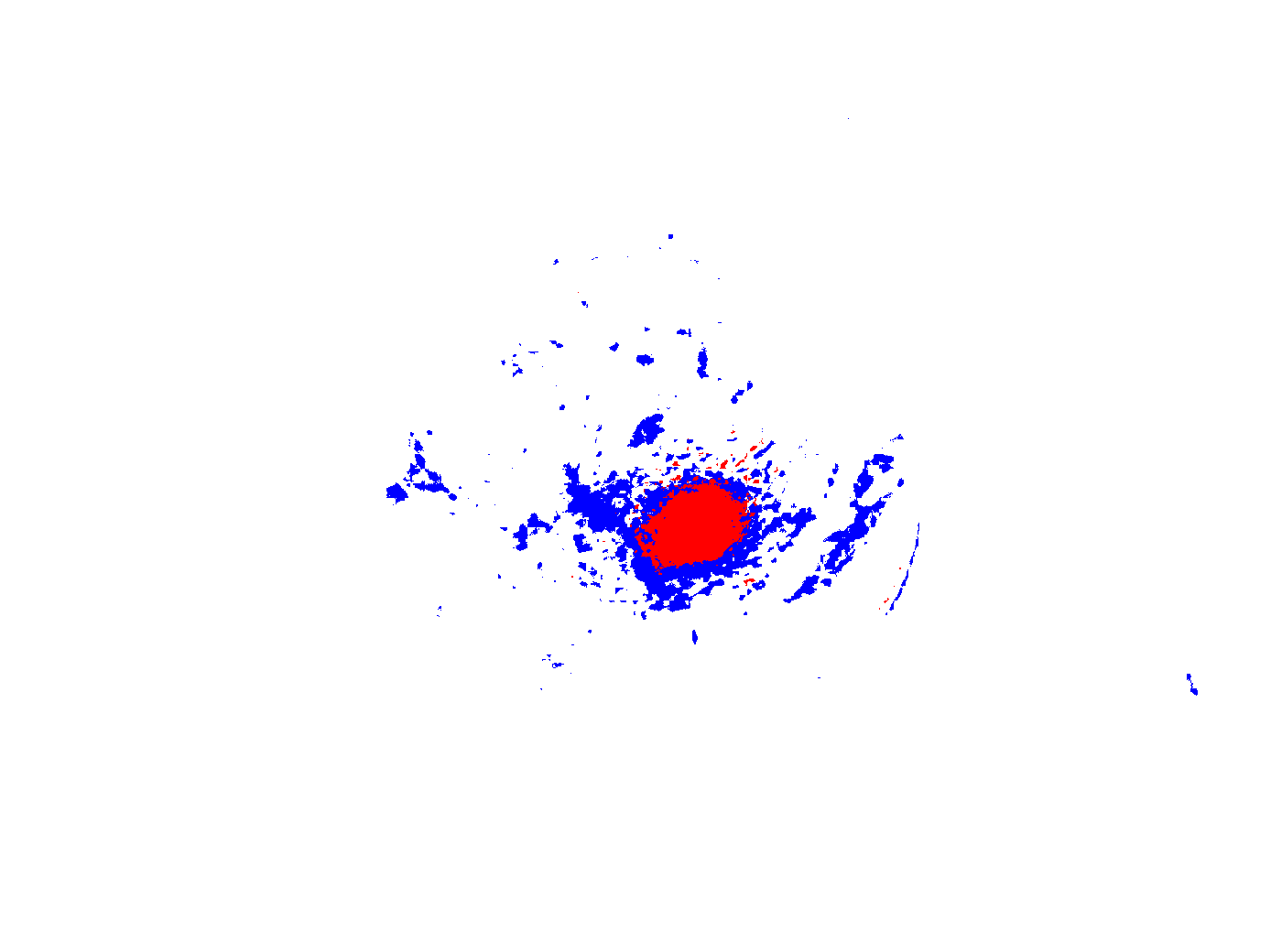

Supplement: S1 File — (ZIP) [file pone.0173647.s002.zip › S1_File/20160830_225416/G2_3.jpg.tiff]

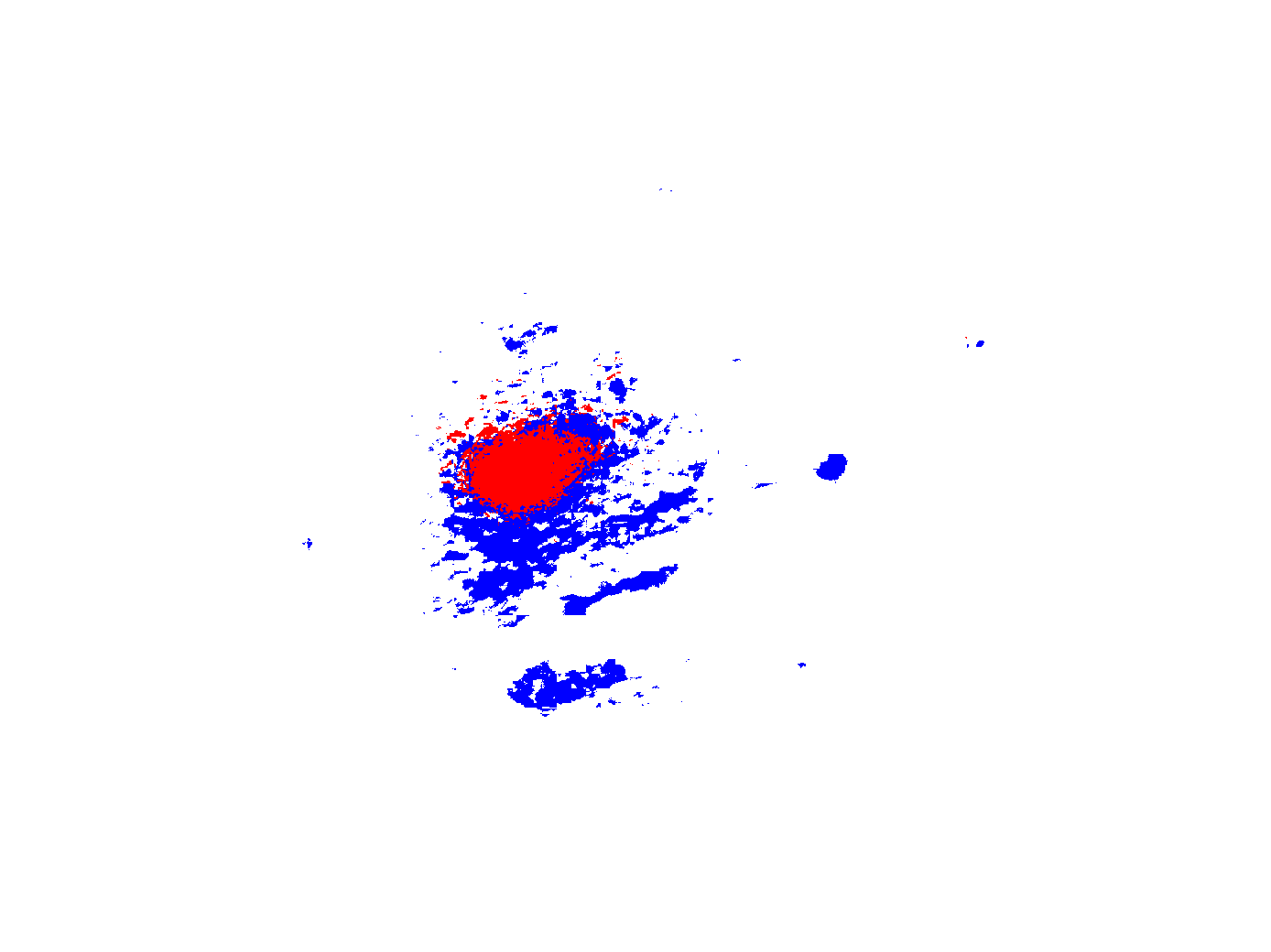

Supplement: S1 File — (ZIP) [file pone.0173647.s002.zip › S1_File/20160830_225416/G2_4.jpg.tiff]

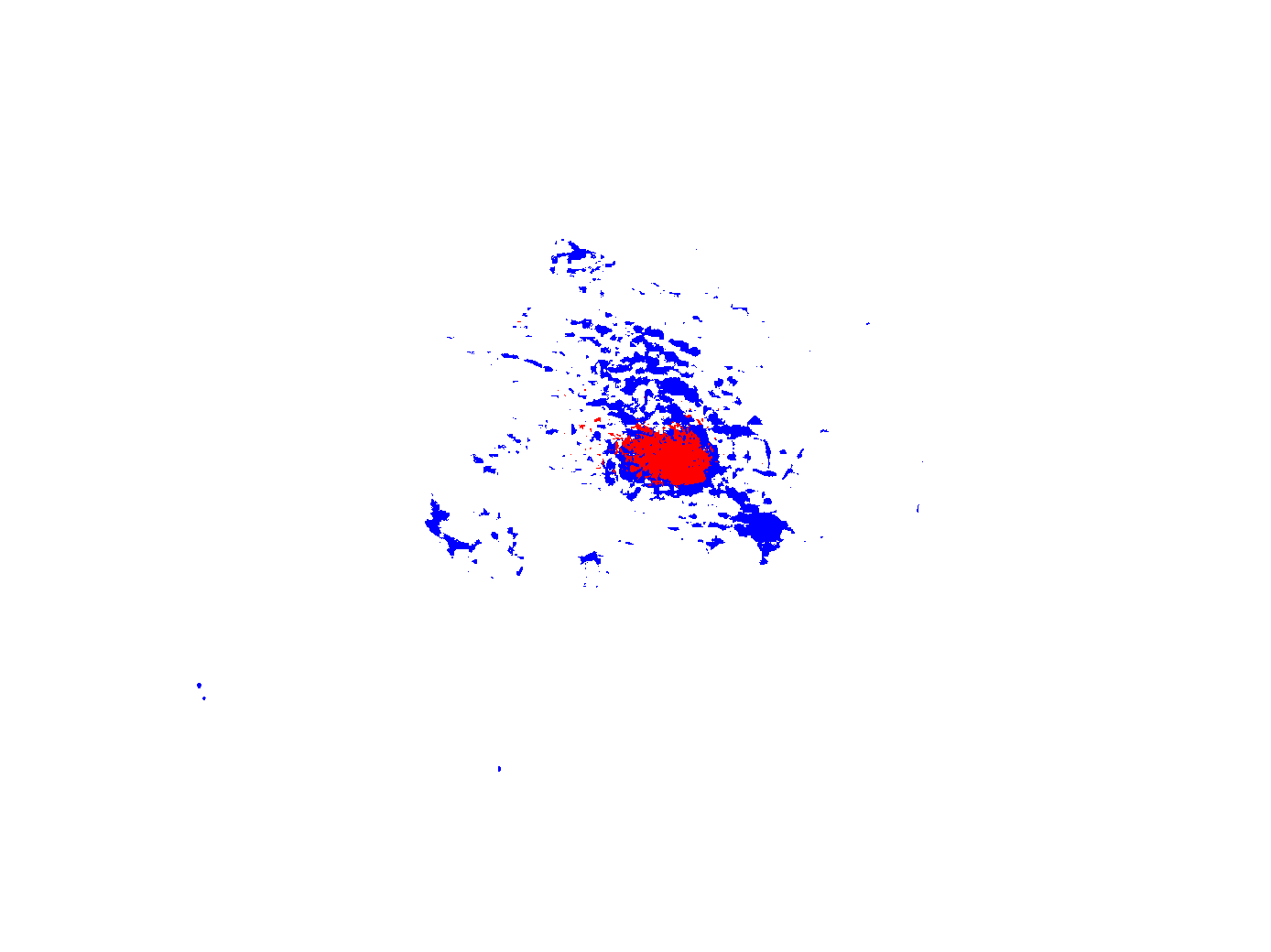

Supplement: S1 File — (ZIP) [file pone.0173647.s002.zip › S1_File/20160830_225416/G2_5.jpg.tiff]

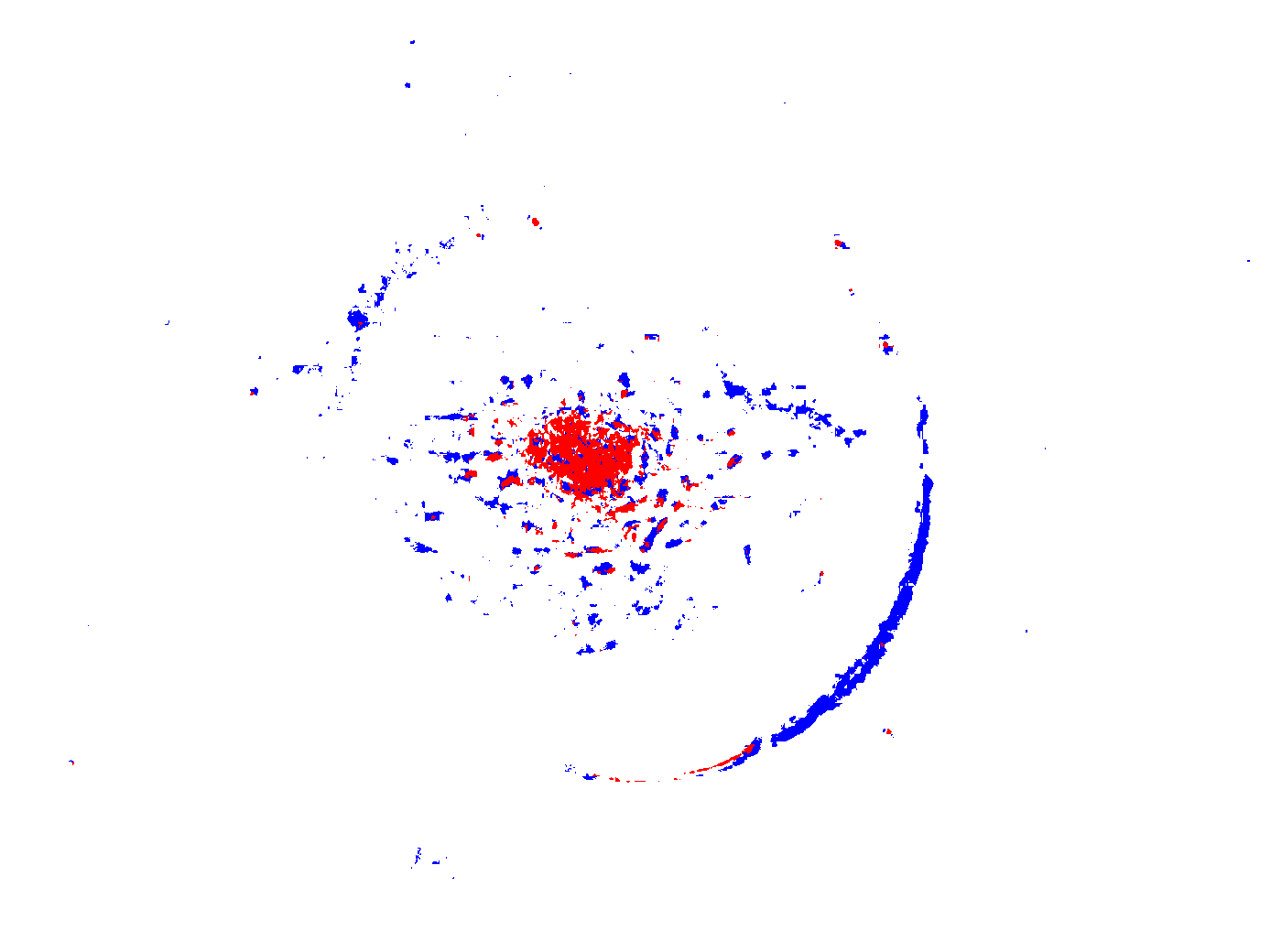

Supplement: S1 File — (ZIP) [file pone.0173647.s002.zip › S1_File/20160830_225416/G3_1.jpg.tiff]

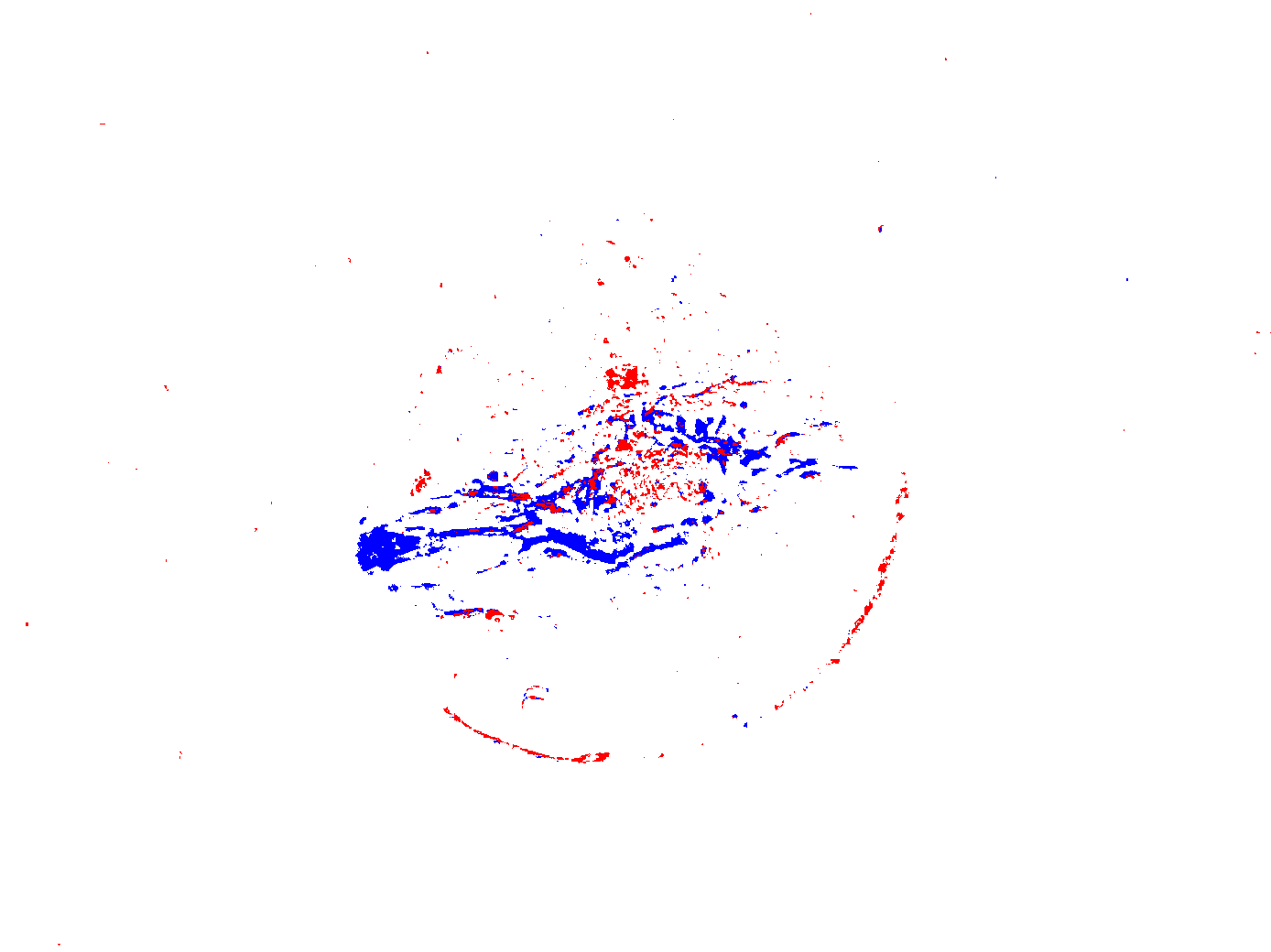

Supplement: S1 File — (ZIP) [file pone.0173647.s002.zip › S1_File/20160830_225416/G3_2.jpg.tiff]

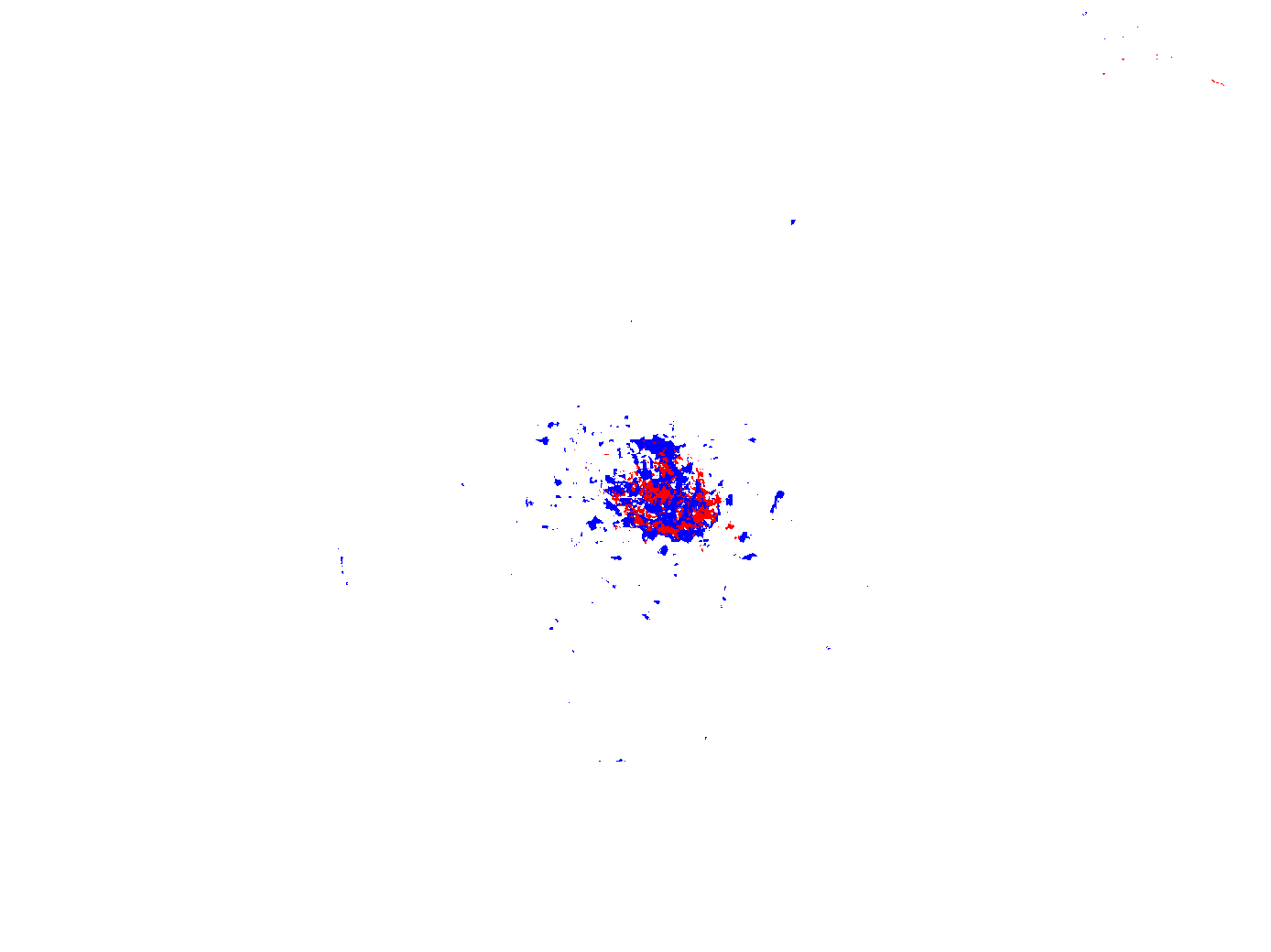

Supplement: S1 File — (ZIP) [file pone.0173647.s002.zip › S1_File/20160830_225416/G3_3.jpg.tiff]

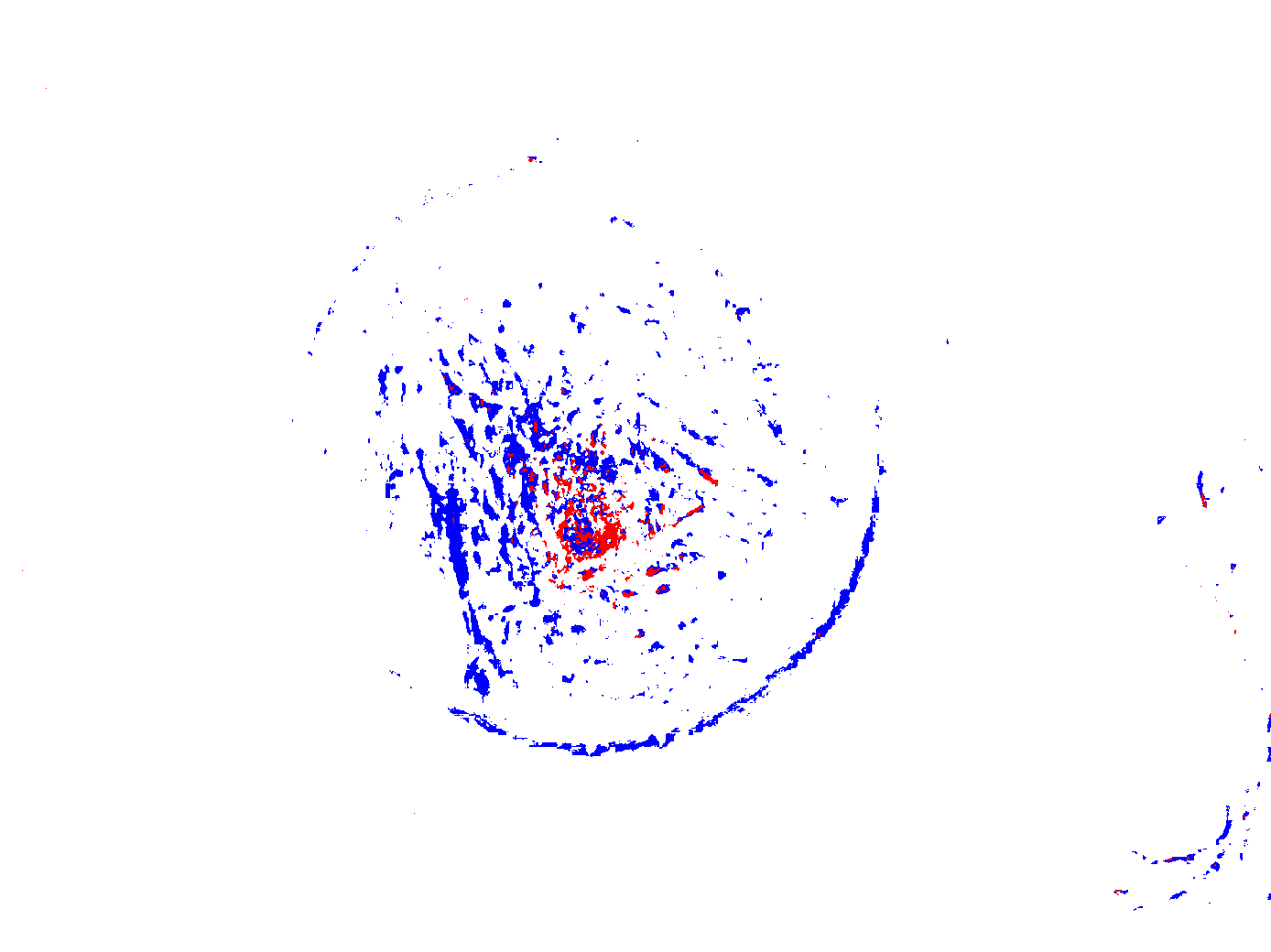

Supplement: S1 File — (ZIP) [file pone.0173647.s002.zip › S1_File/20160830_225416/G3_4.jpg.tiff]

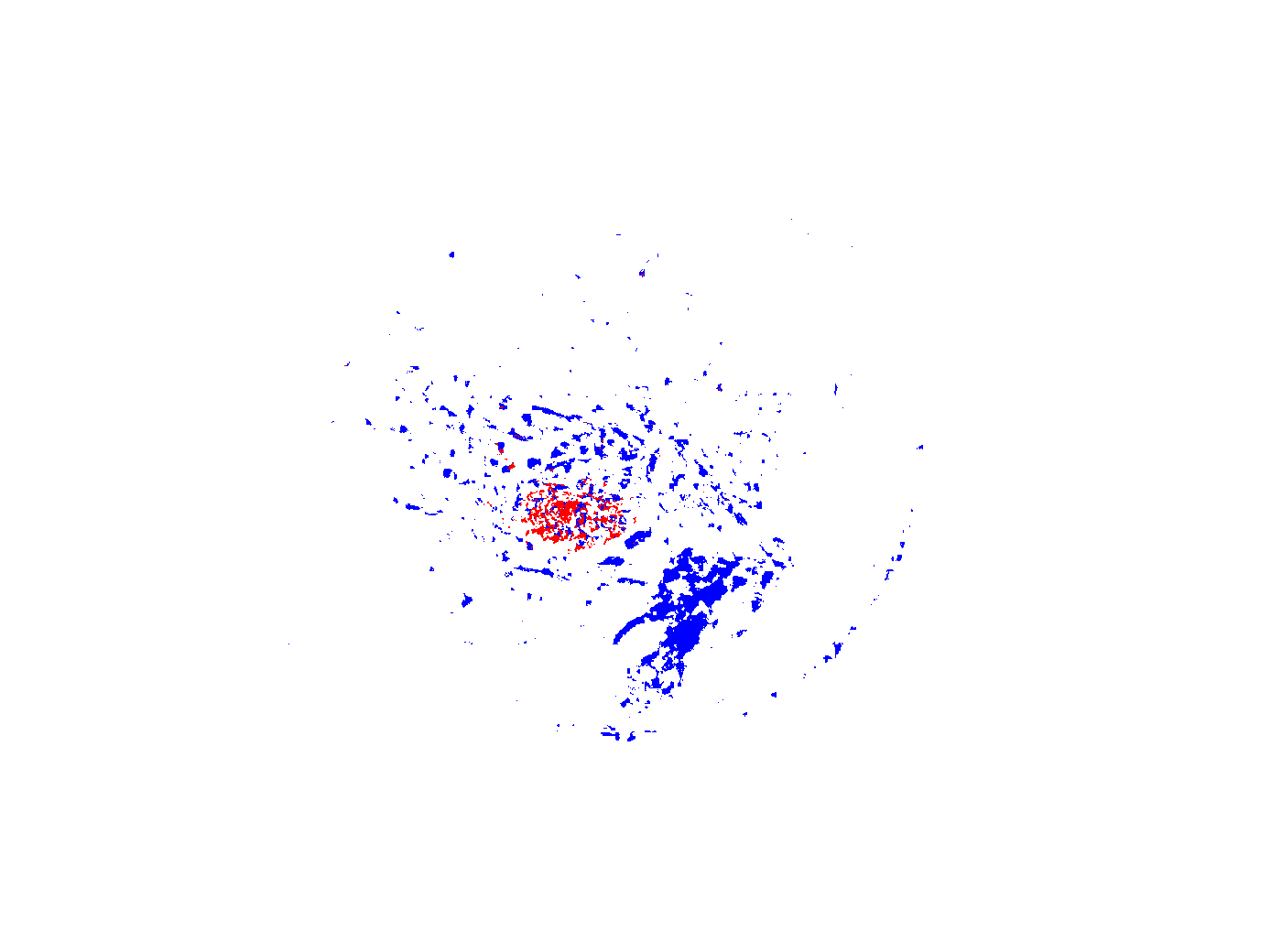

Supplement: S1 File — (ZIP) [file pone.0173647.s002.zip › S1_File/20160830_225416/G3_5.jpg.tiff]

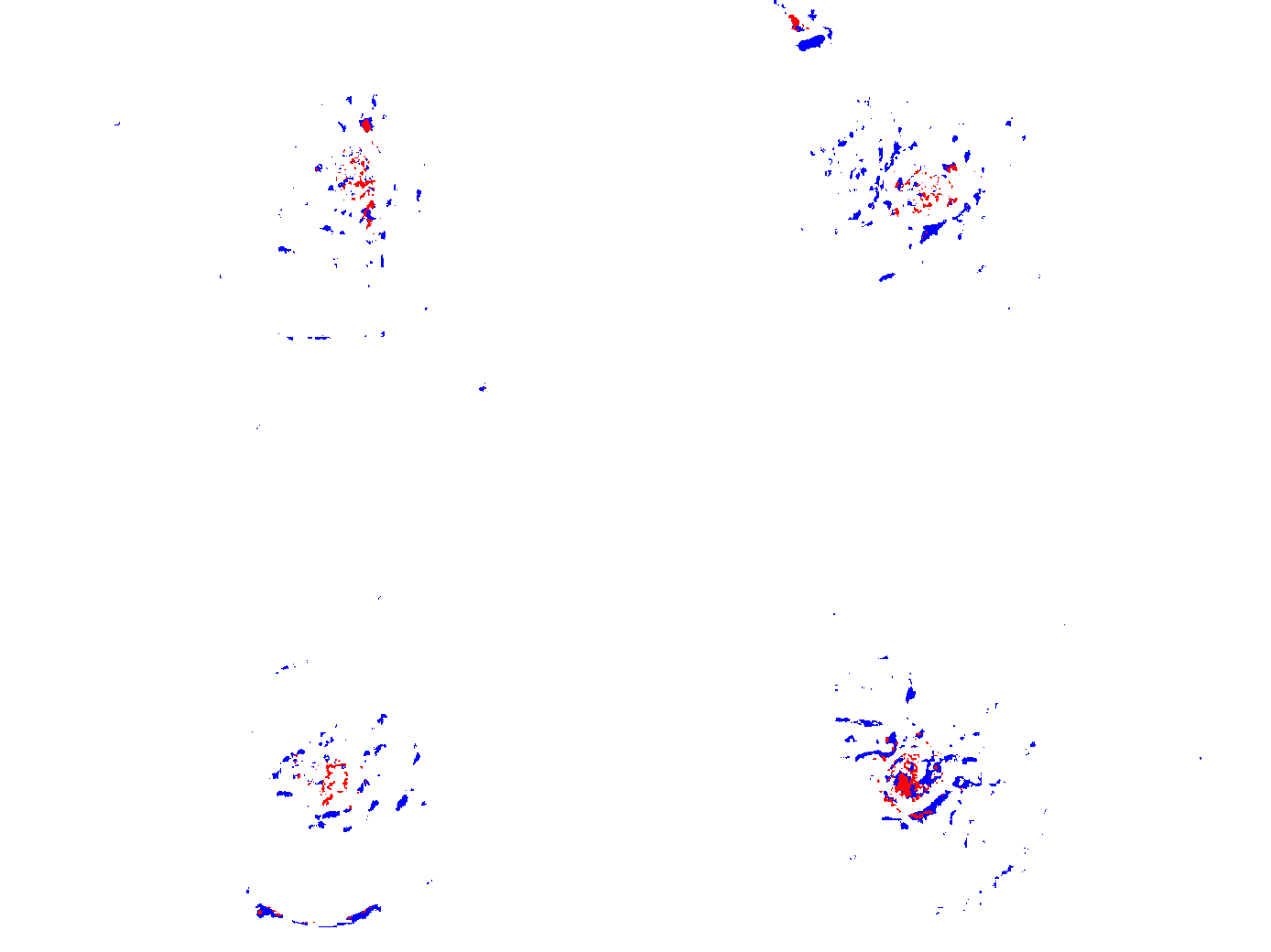

Supplement: S1 File — (ZIP) [file pone.0173647.s002.zip › S1_File/20160830_225416/G4_1.jpg.tiff]

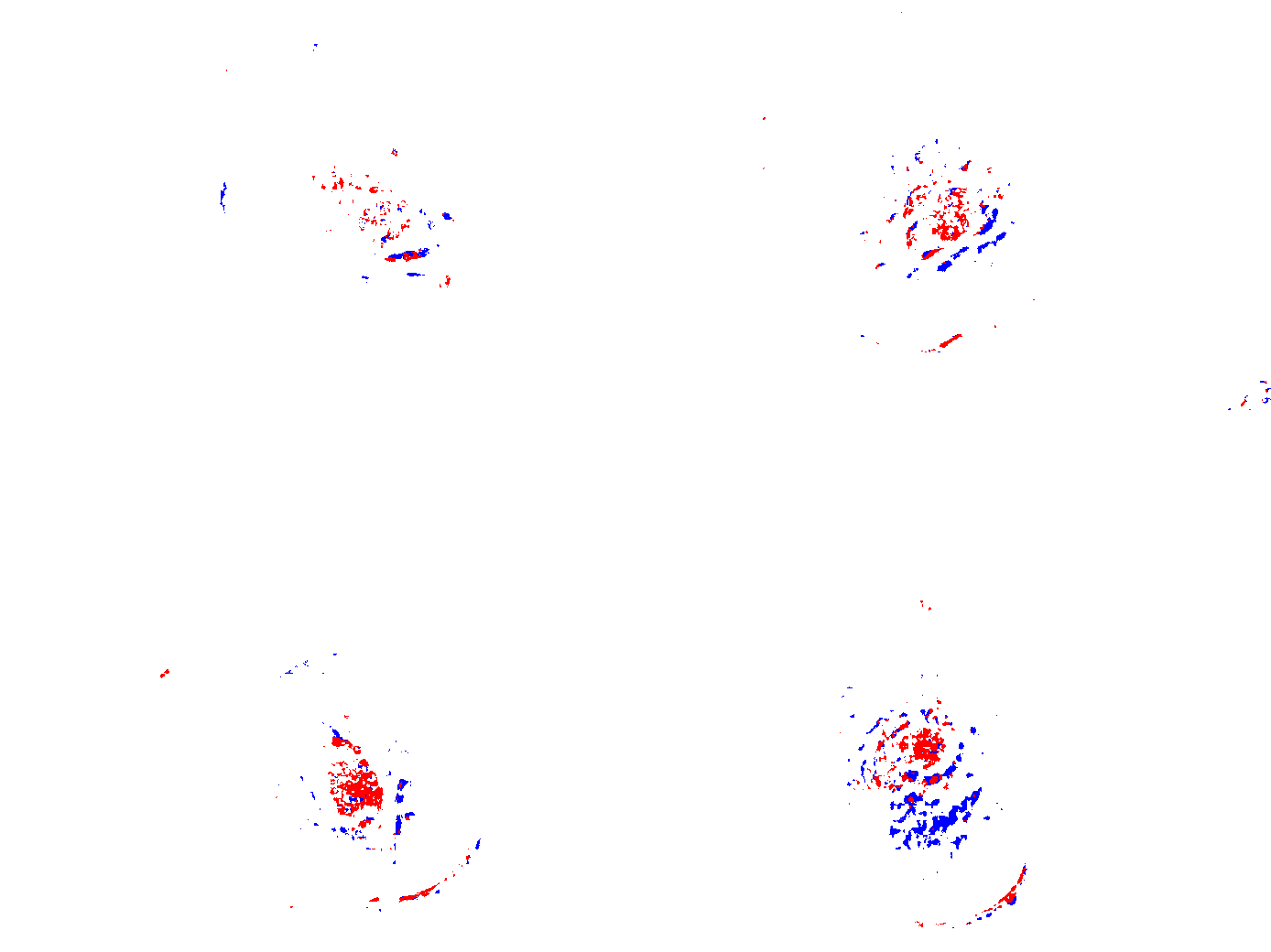

Supplement: S1 File — (ZIP) [file pone.0173647.s002.zip › S1_File/20160830_225416/G4_2.jpg.tiff]

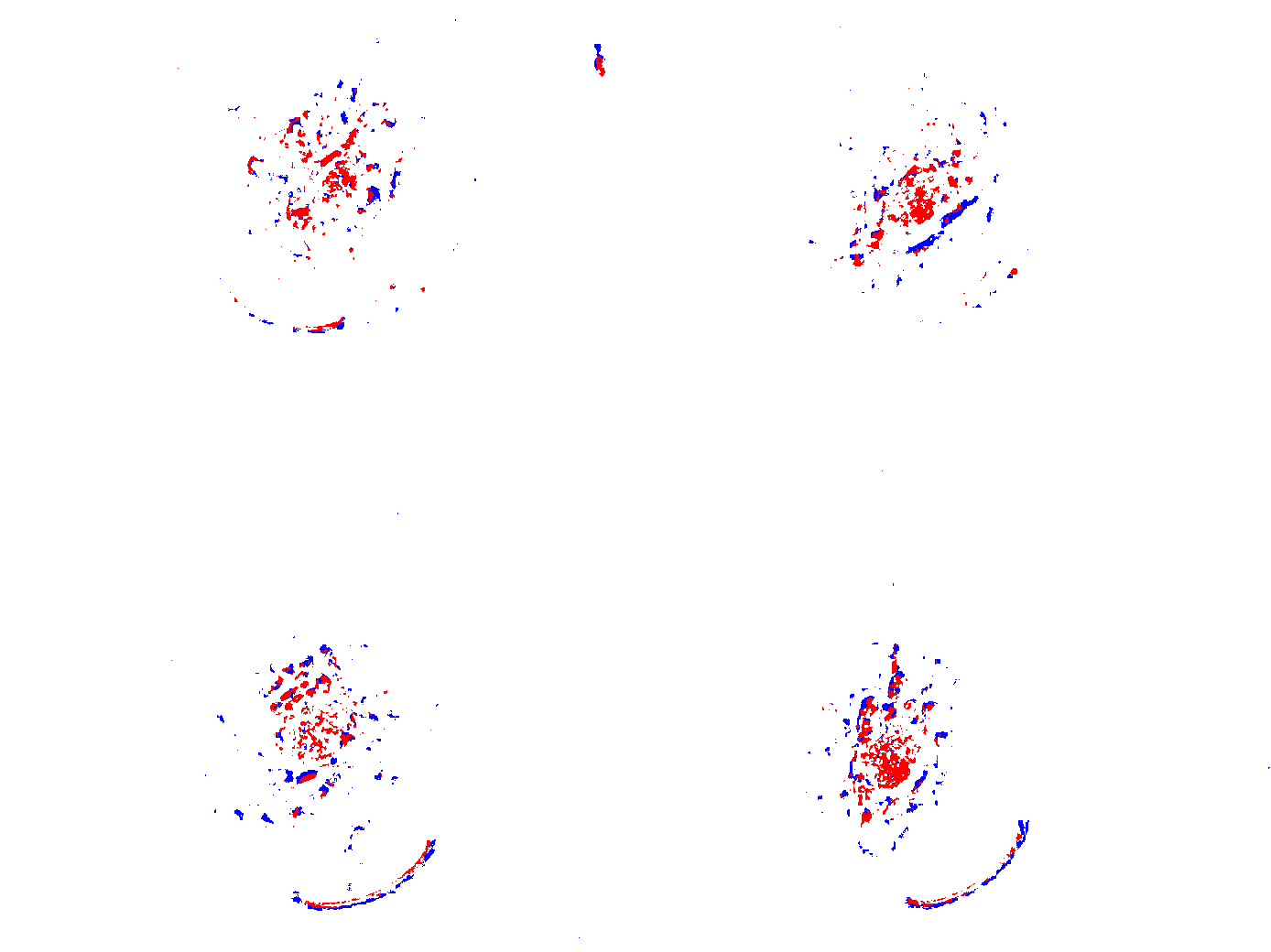

Supplement: S1 File — (ZIP) [file pone.0173647.s002.zip › S1_File/20160830_225416/G4_3.jpg.tiff]

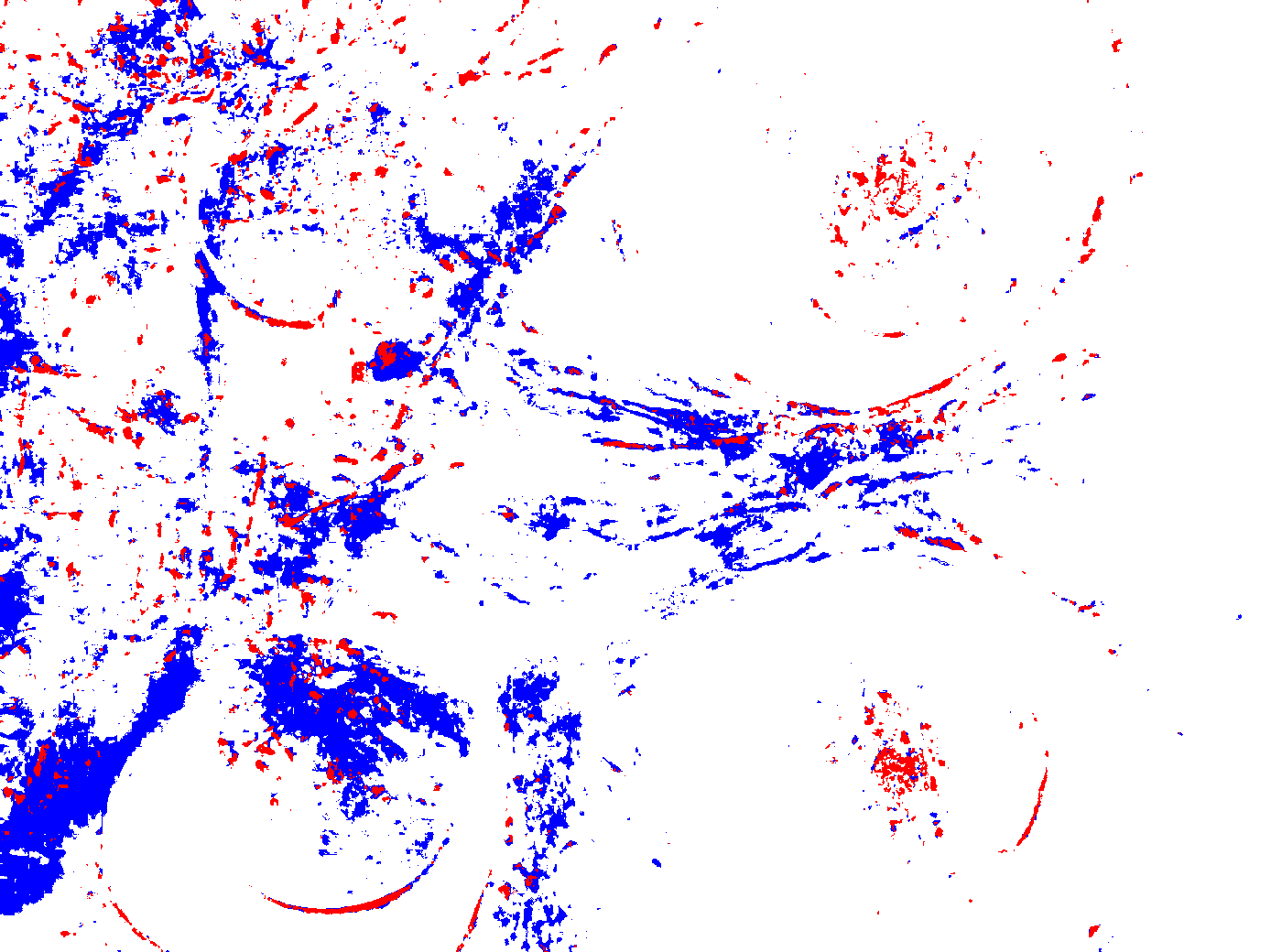

Supplement: S1 File — (ZIP) [file pone.0173647.s002.zip › S1_File/20160830_225416/G4_4.jpg.tiff]

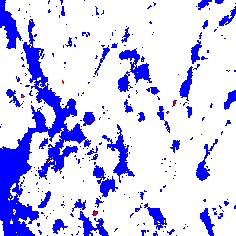

Supplement: S1 File — (ZIP) [file pone.0173647.s002.zip › S1_File/20160830_225416/GC_1.jpg.tiff]

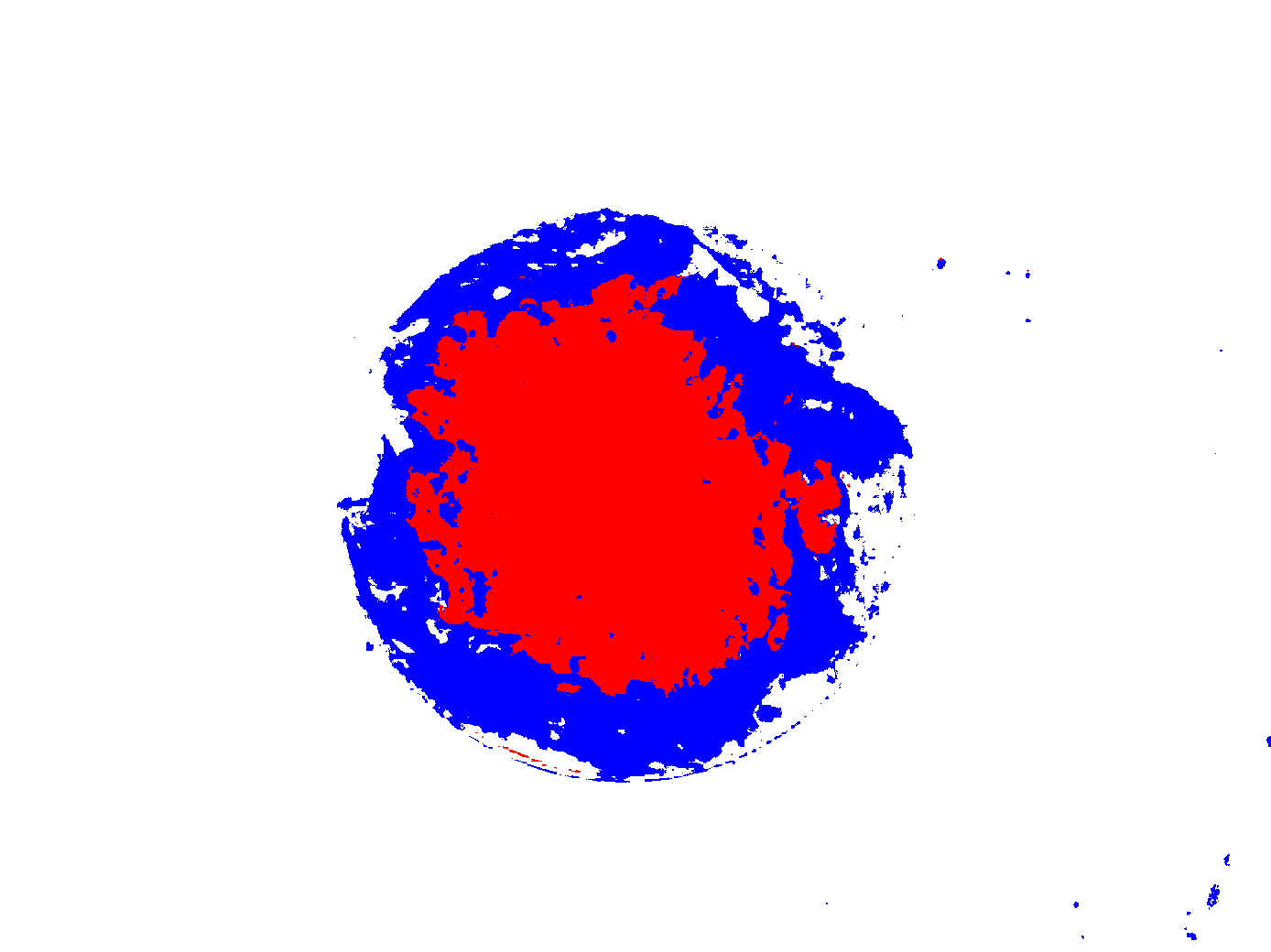

Supplement: S1 File — (ZIP) [file pone.0173647.s002.zip › S1_File/20160830_225416/M2_1.jpg.tiff]

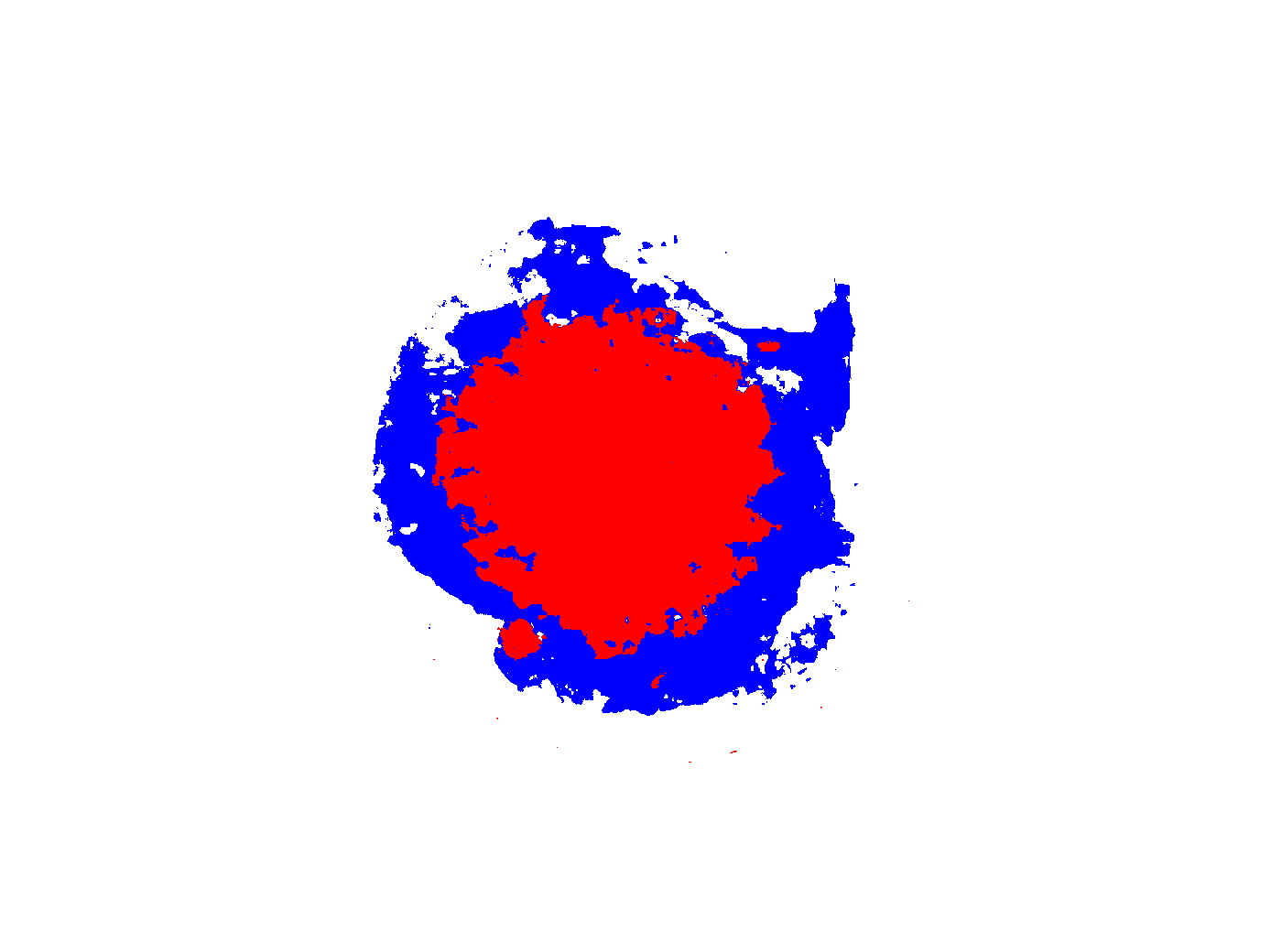

Supplement: S1 File — (ZIP) [file pone.0173647.s002.zip › S1_File/20160830_225416/M2_2.jpg.tiff]

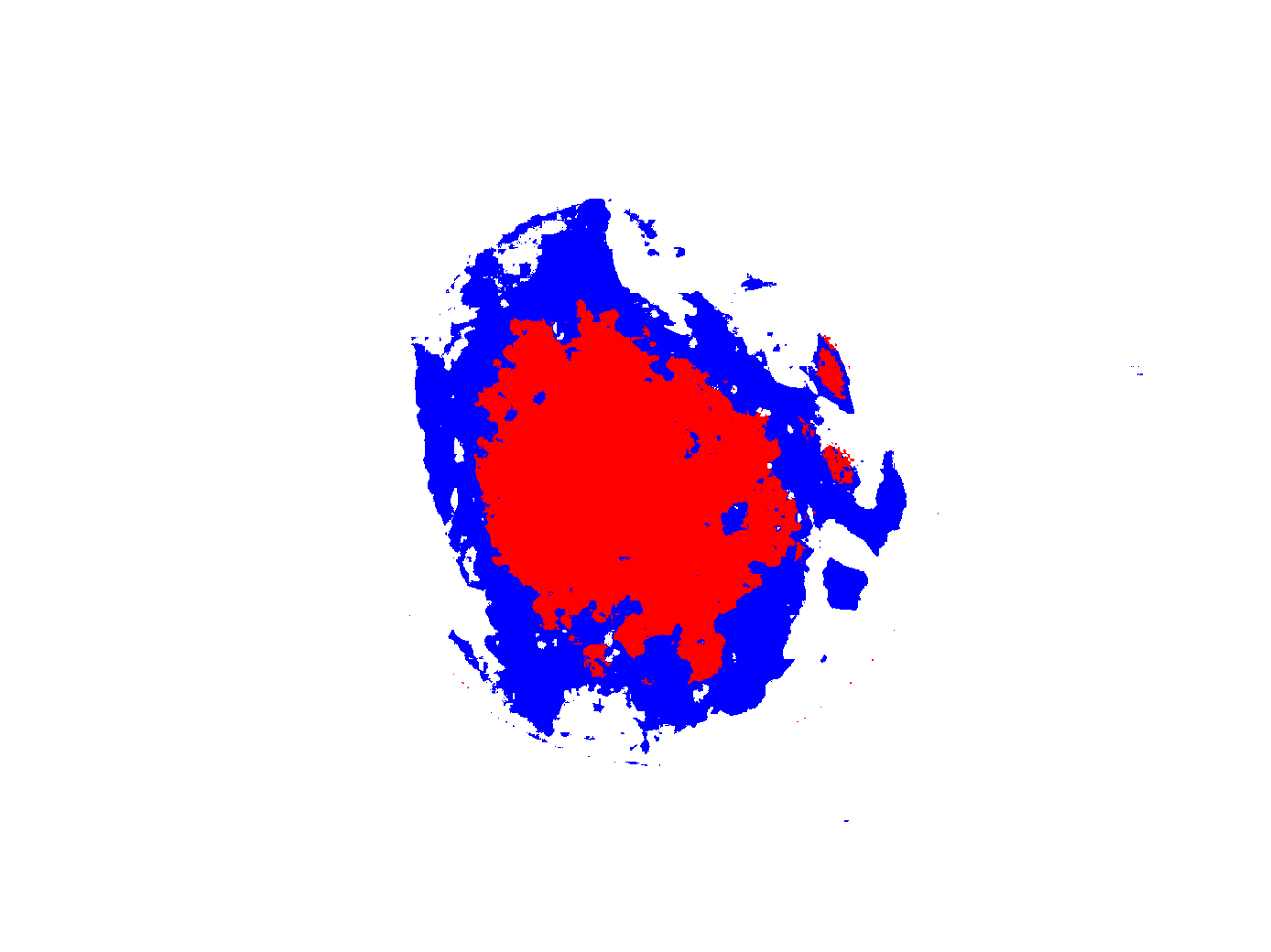

Supplement: S1 File — (ZIP) [file pone.0173647.s002.zip › S1_File/20160830_225416/M2_3.jpg.tiff]

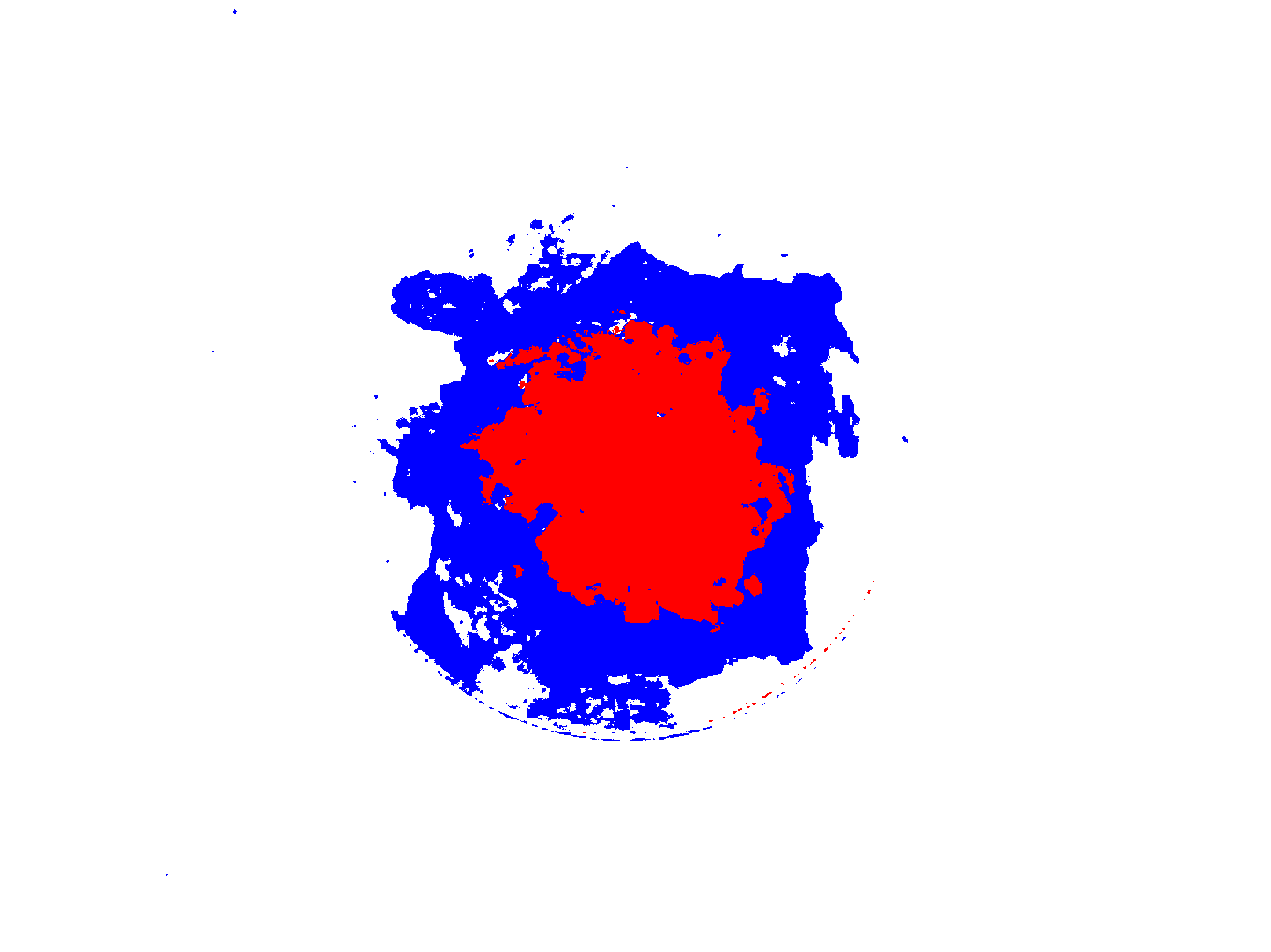

Supplement: S1 File — (ZIP) [file pone.0173647.s002.zip › S1_File/20160830_225416/M2_4.jpg.tiff]

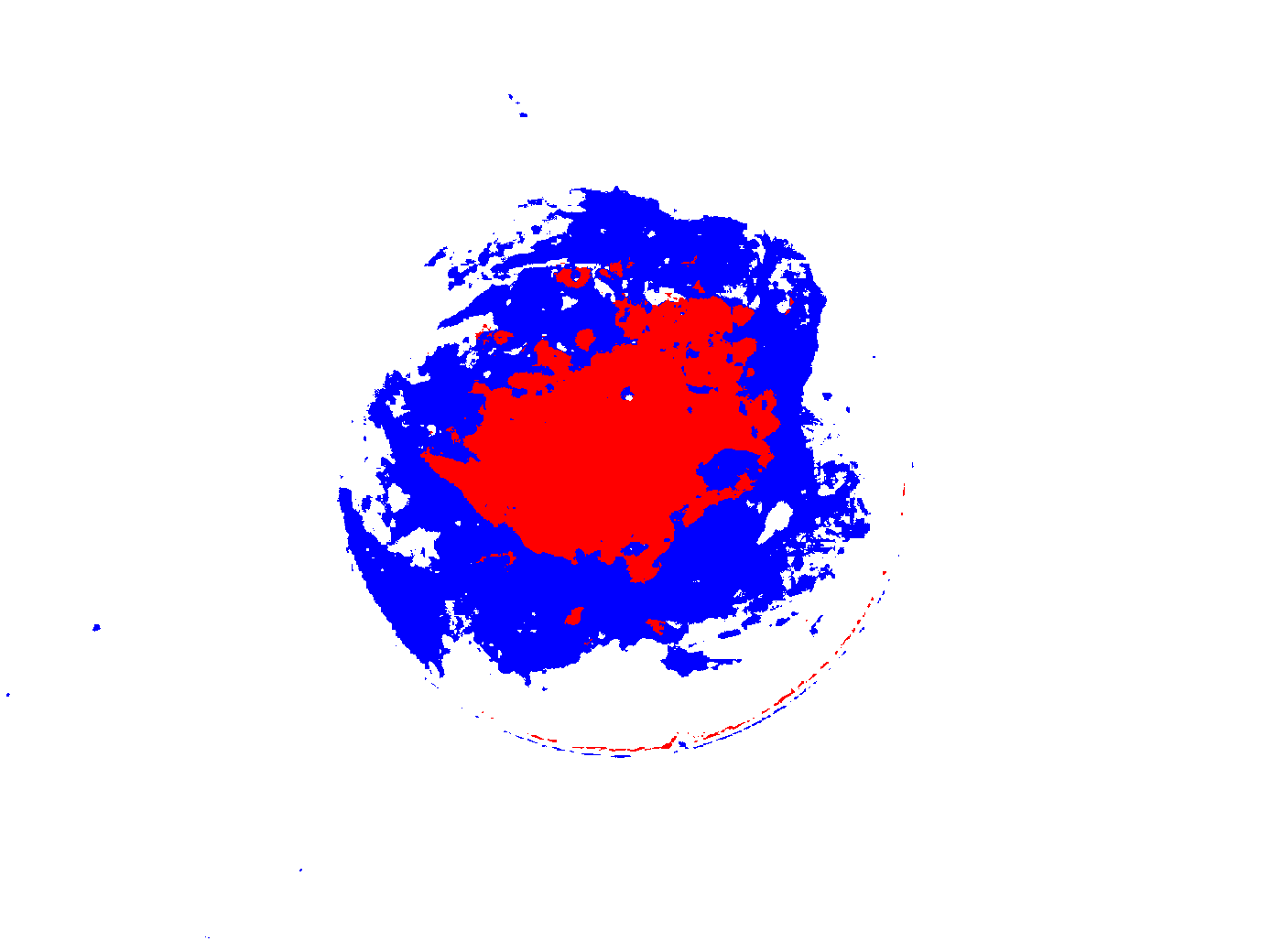

Supplement: S1 File — (ZIP) [file pone.0173647.s002.zip › S1_File/20160830_225416/M2_5.jpg.tiff]

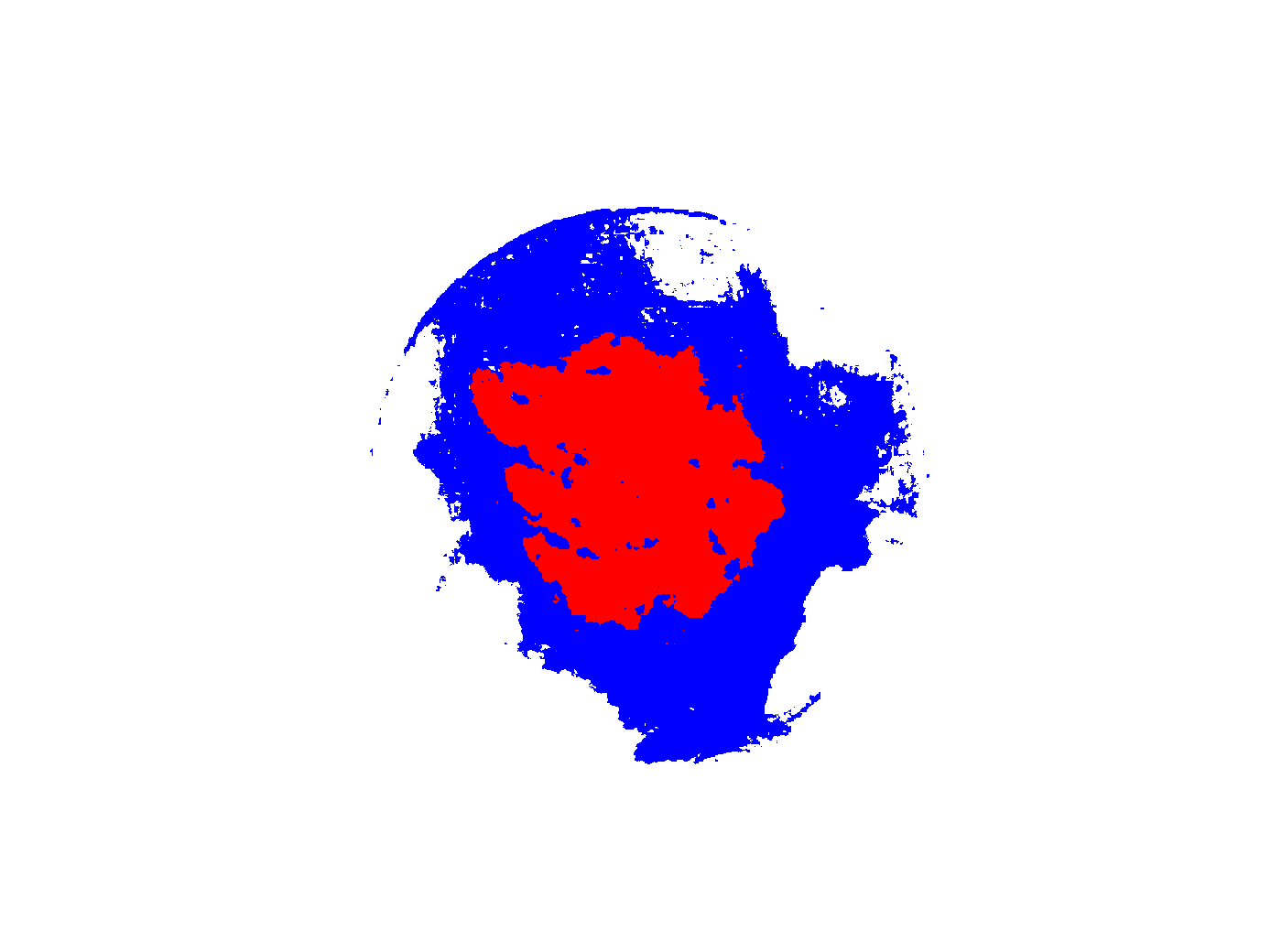

Supplement: S1 File — (ZIP) [file pone.0173647.s002.zip › S1_File/20160830_225416/M3_1.jpg.tiff]

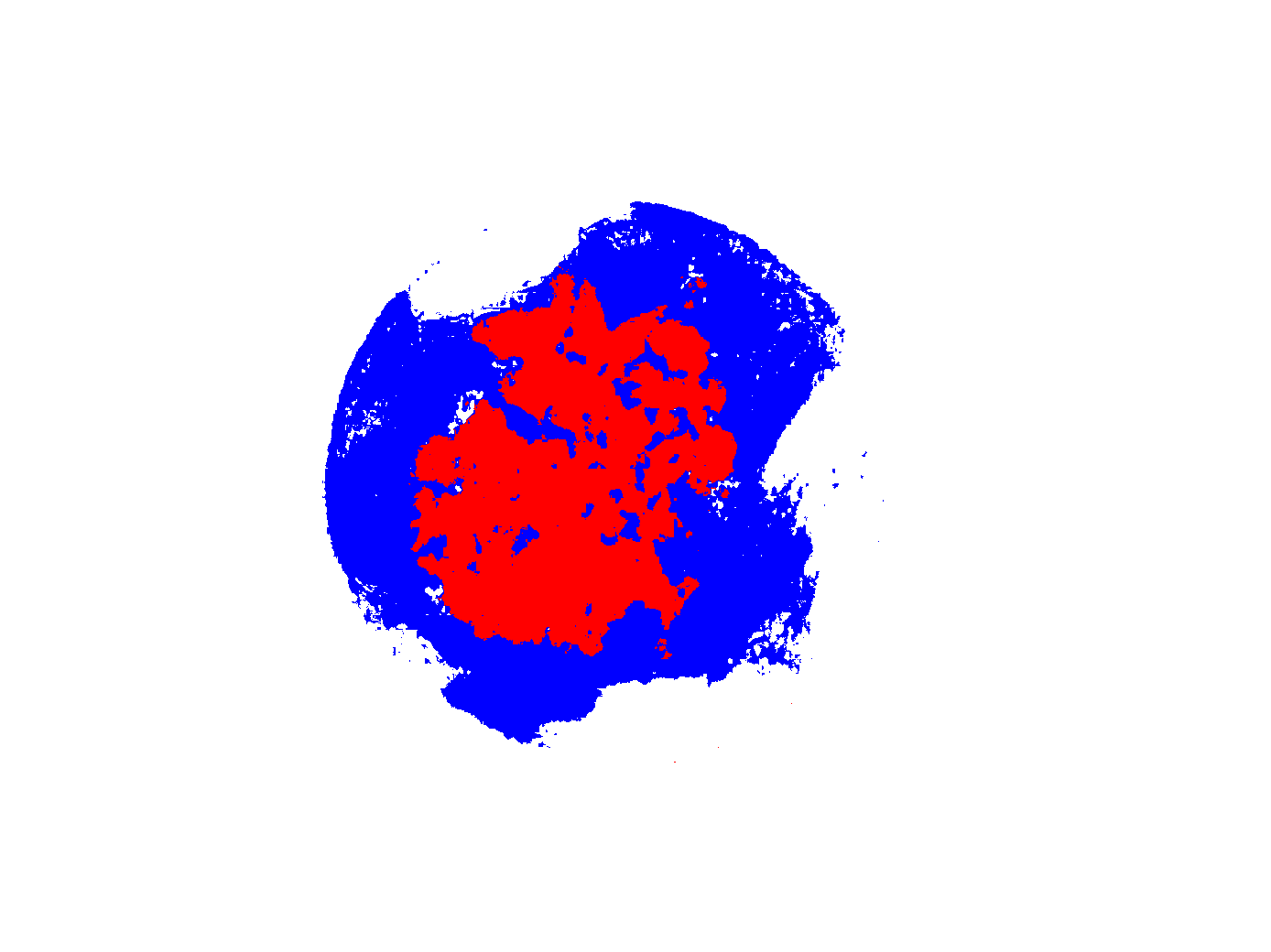

Supplement: S1 File — (ZIP) [file pone.0173647.s002.zip › S1_File/20160830_225416/M3_2.jpg.tiff]

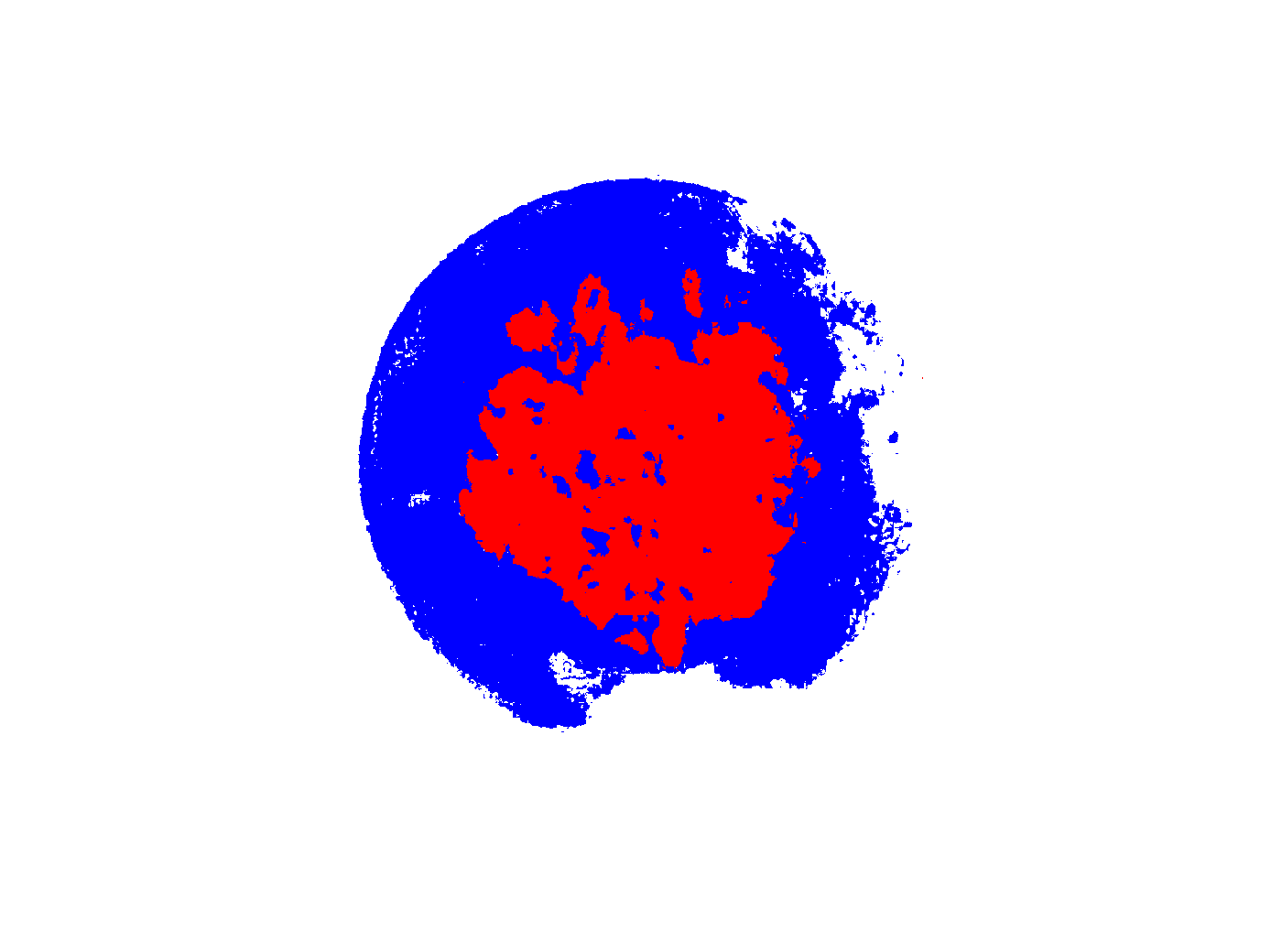

Supplement: S1 File — (ZIP) [file pone.0173647.s002.zip › S1_File/20160830_225416/M3_3.jpg.tiff]

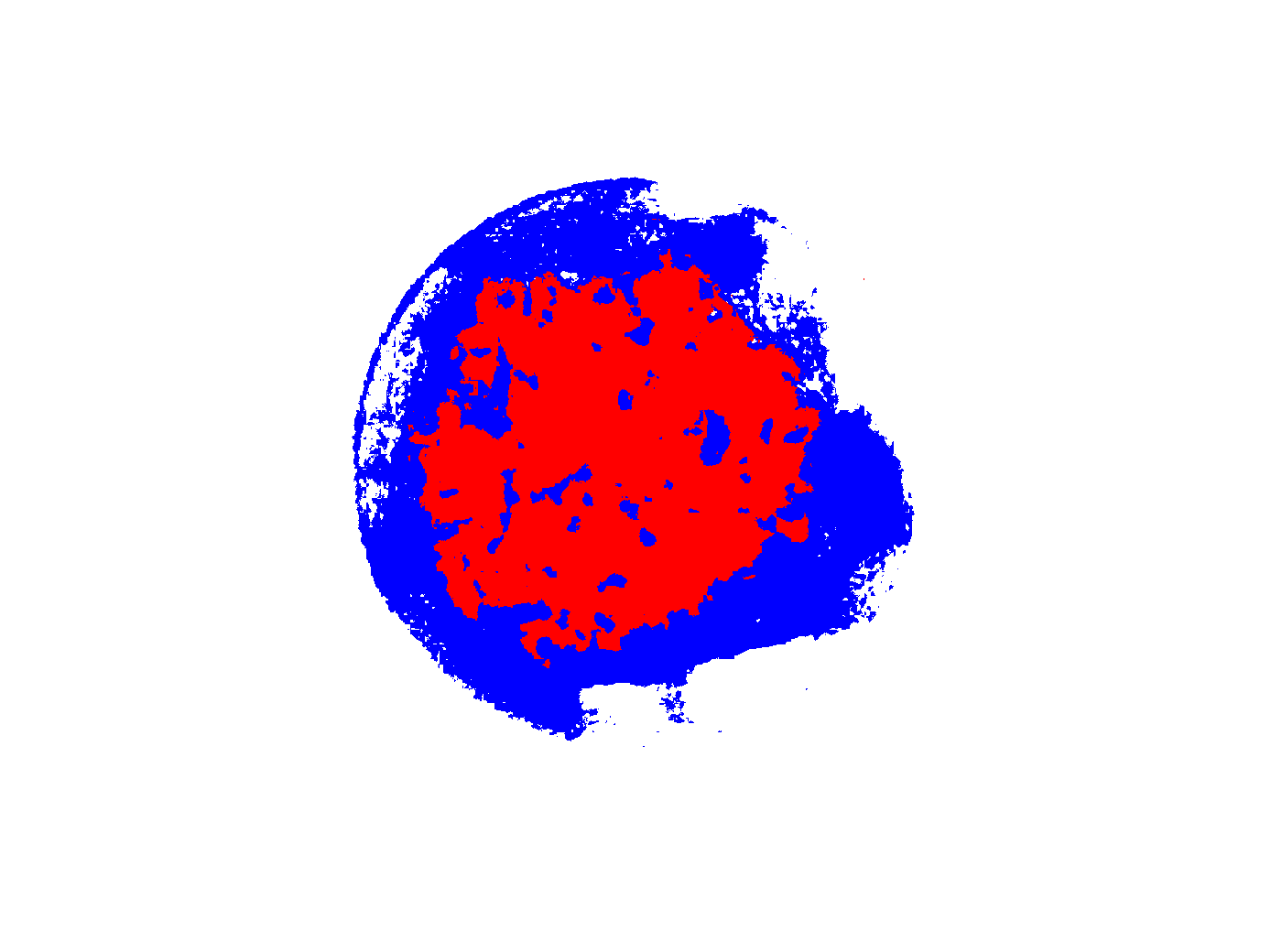

Supplement: S1 File — (ZIP) [file pone.0173647.s002.zip › S1_File/20160830_225416/M3_4.jpg.tiff]

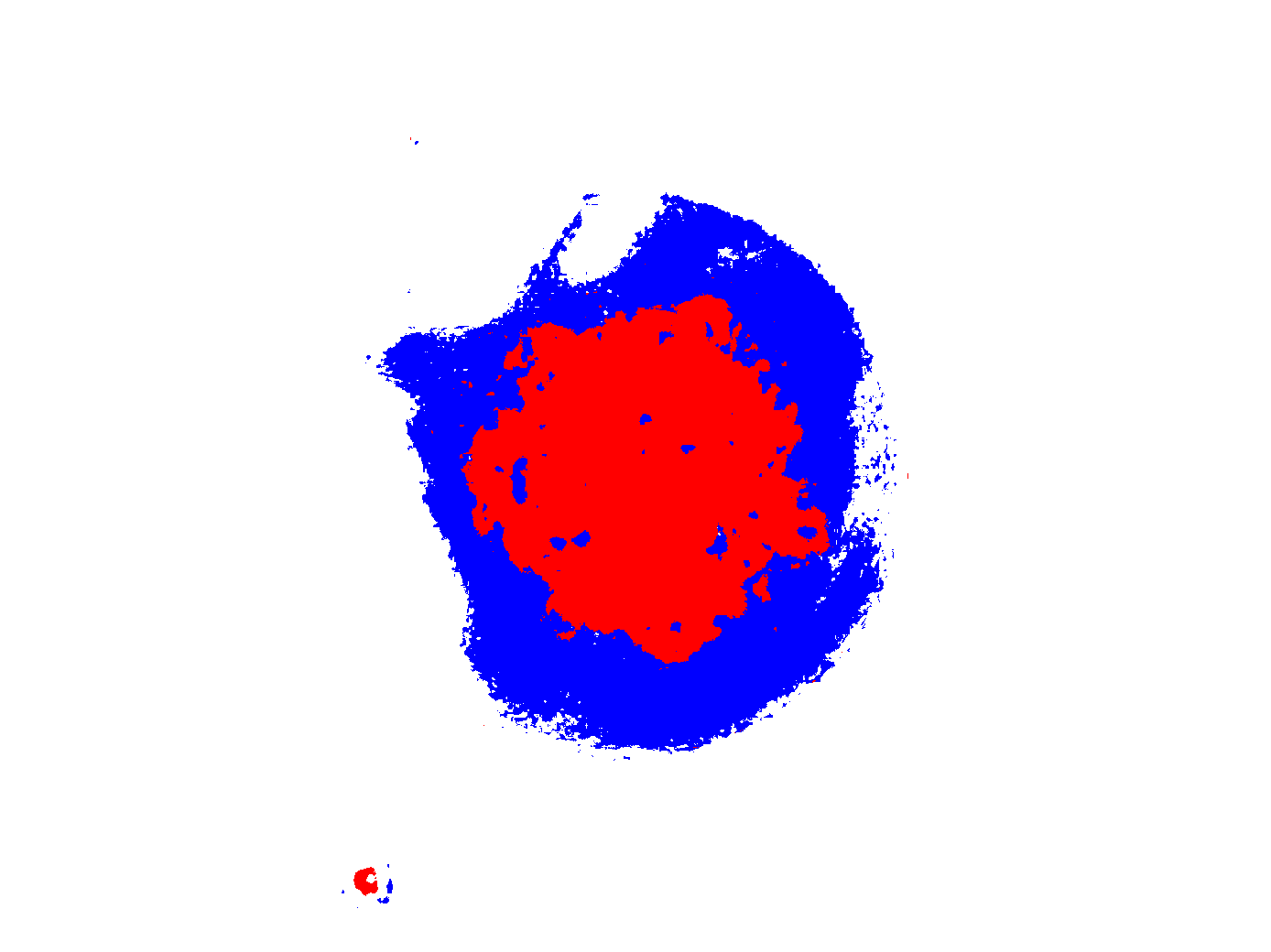

Supplement: S1 File — (ZIP) [file pone.0173647.s002.zip › S1_File/20160830_225416/M3_5.jpg.tiff]

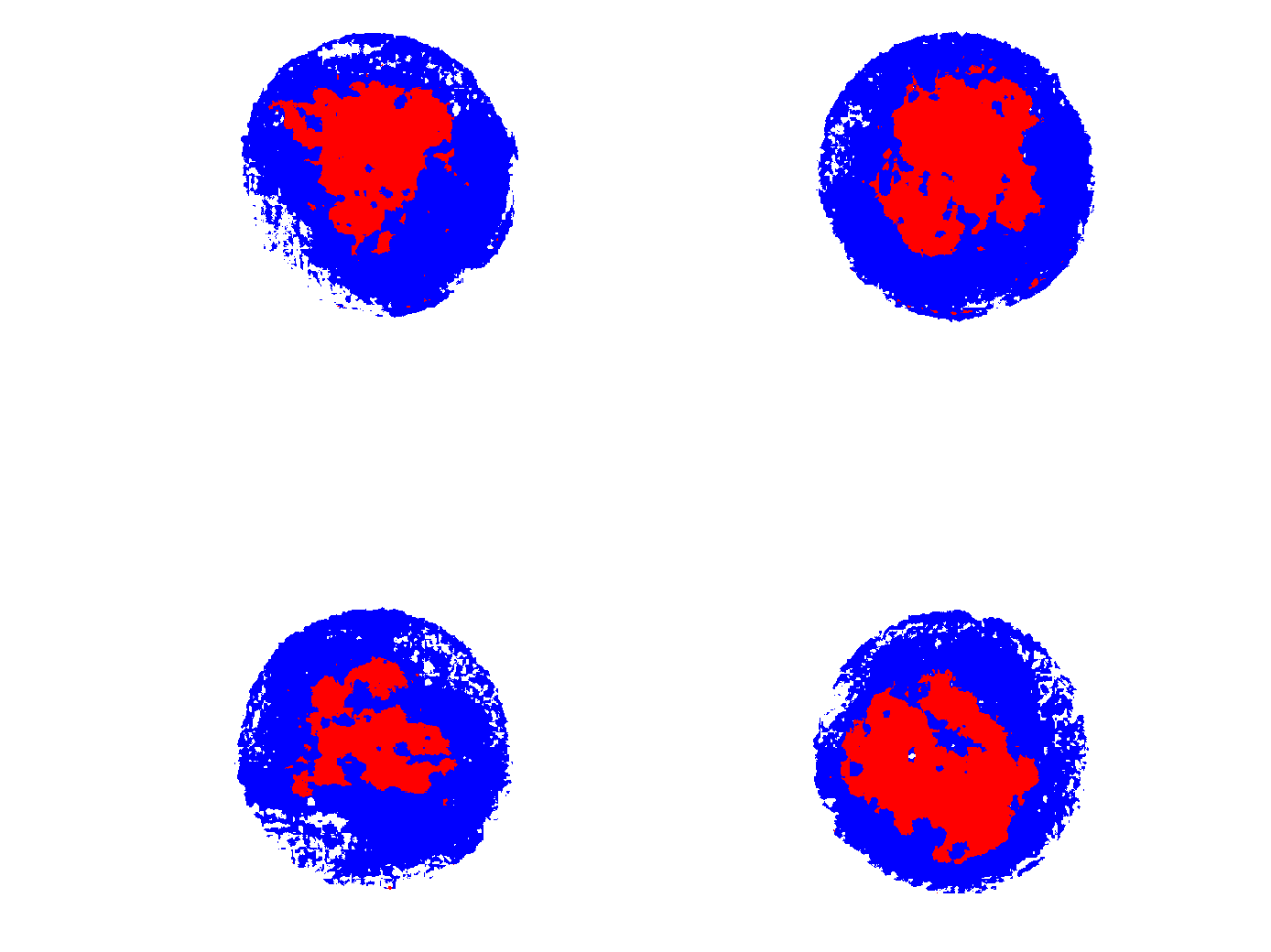

Supplement: S1 File — (ZIP) [file pone.0173647.s002.zip › S1_File/20160830_225416/M4_1.jpg.tiff]

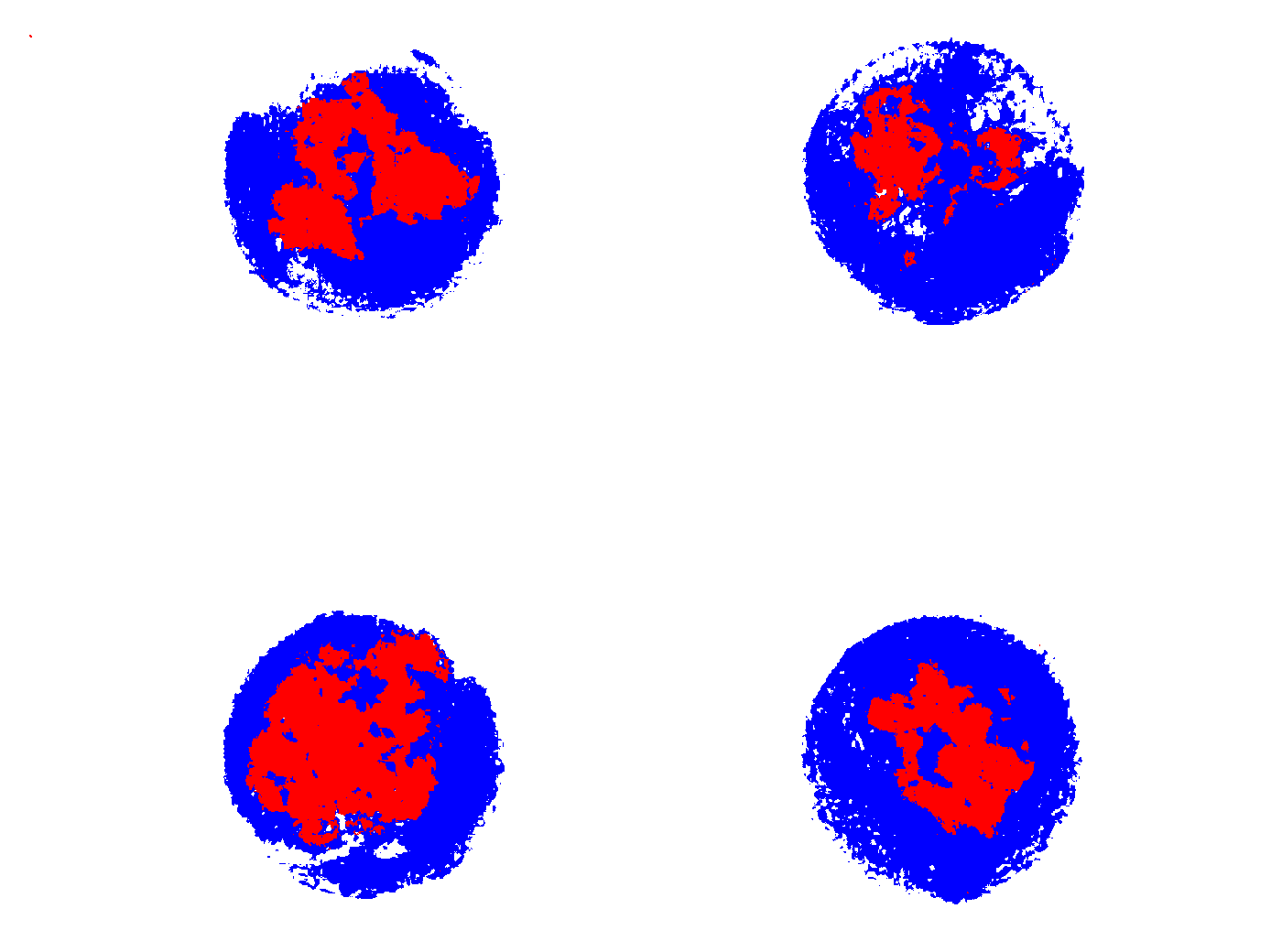

Supplement: S1 File — (ZIP) [file pone.0173647.s002.zip › S1_File/20160830_225416/M4_2.jpg.tiff]

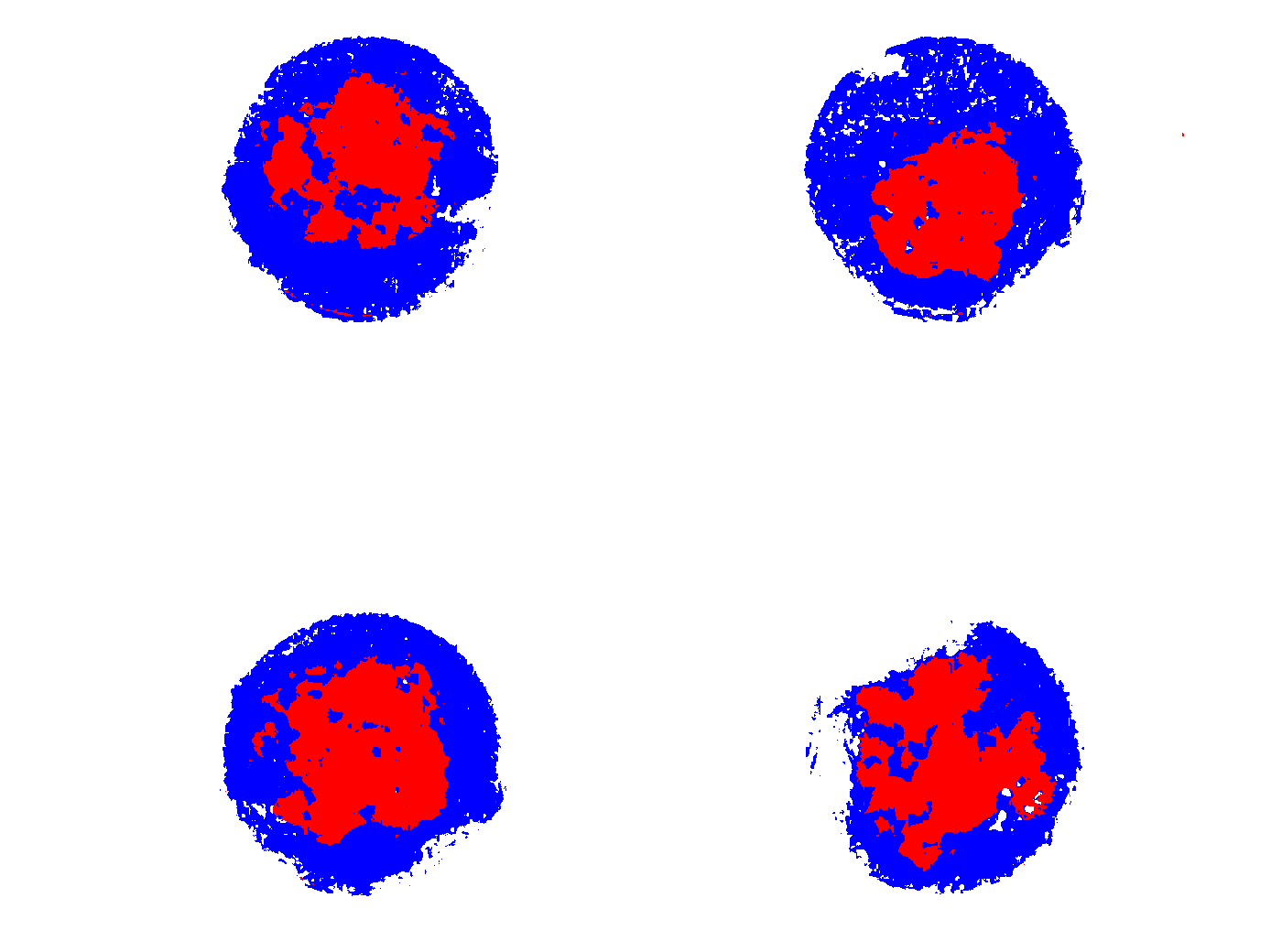

Supplement: S1 File — (ZIP) [file pone.0173647.s002.zip › S1_File/20160830_225416/M4_3.jpg.tiff]

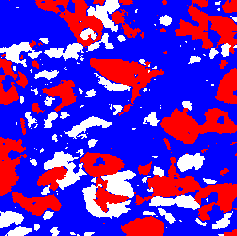

Supplement: S1 File — (ZIP) [file pone.0173647.s002.zip › S1_File/20160830_225416/MC_1.jpg.tiff]

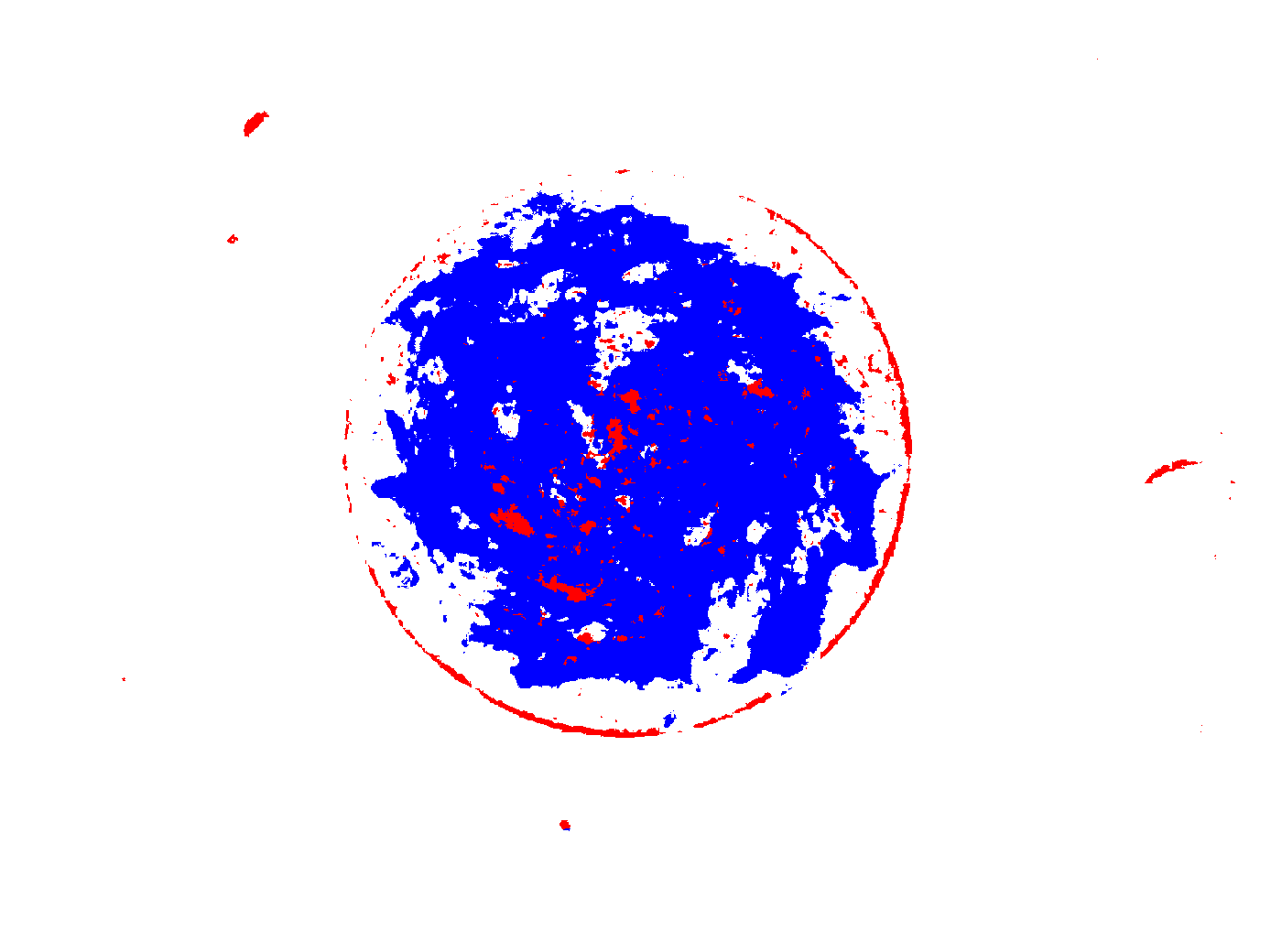

Supplement: S1 File — (ZIP) [file pone.0173647.s002.zip › S1_File/20160830_225416/O2_1.jpg.tiff]

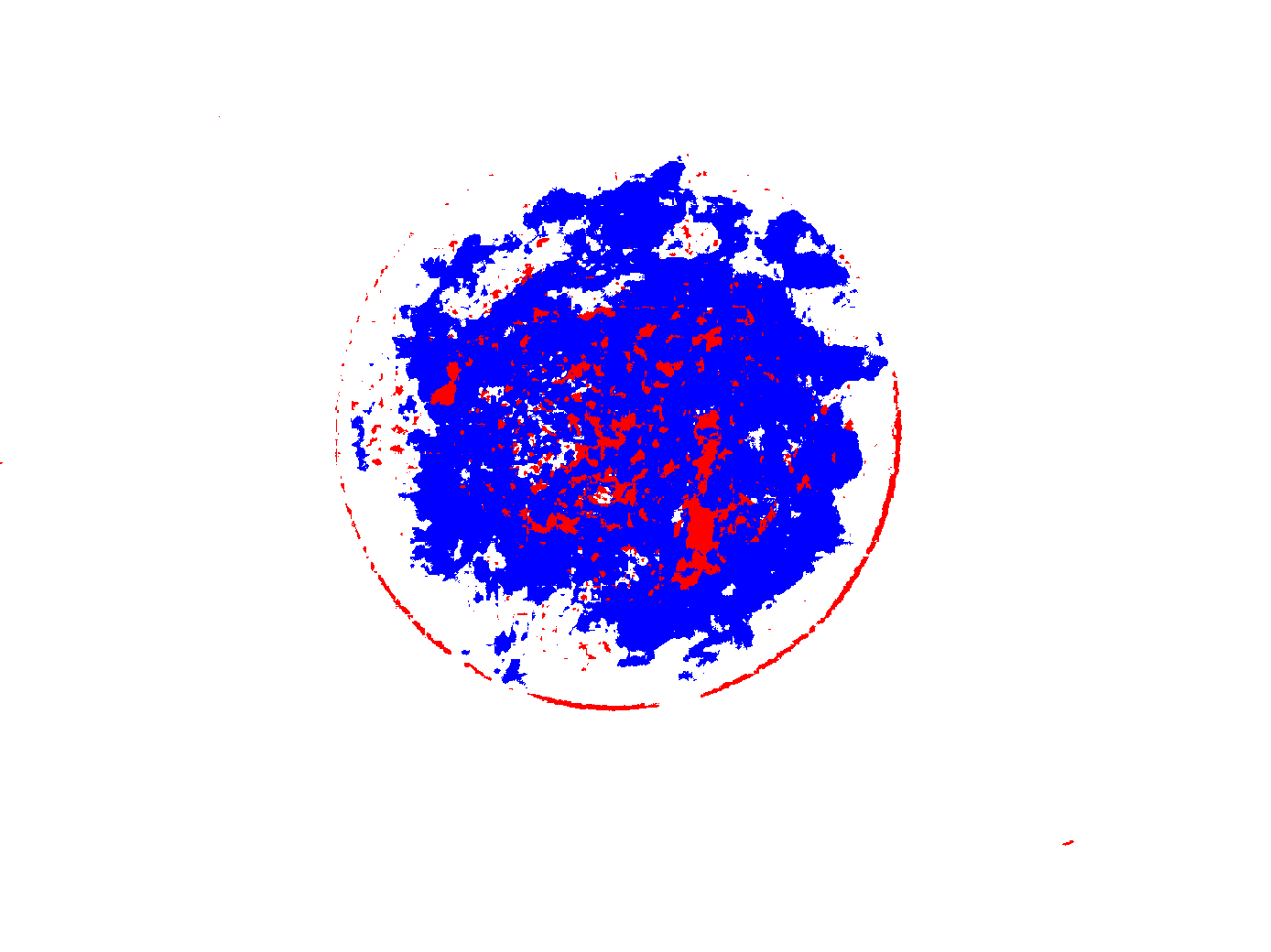

Supplement: S1 File — (ZIP) [file pone.0173647.s002.zip › S1_File/20160830_225416/O2_2.jpg.tiff]

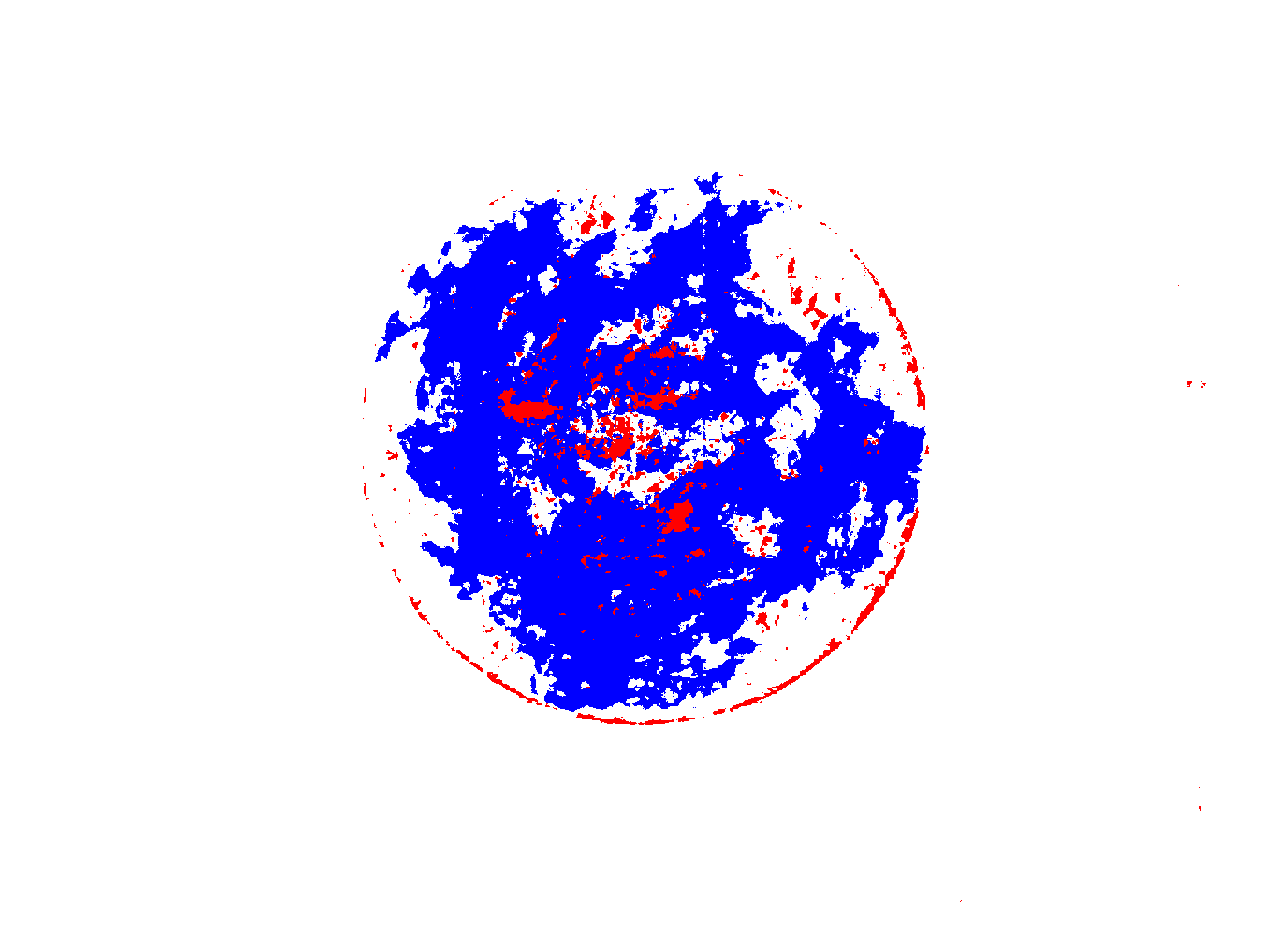

Supplement: S1 File — (ZIP) [file pone.0173647.s002.zip › S1_File/20160830_225416/O2_3.jpg.tiff]

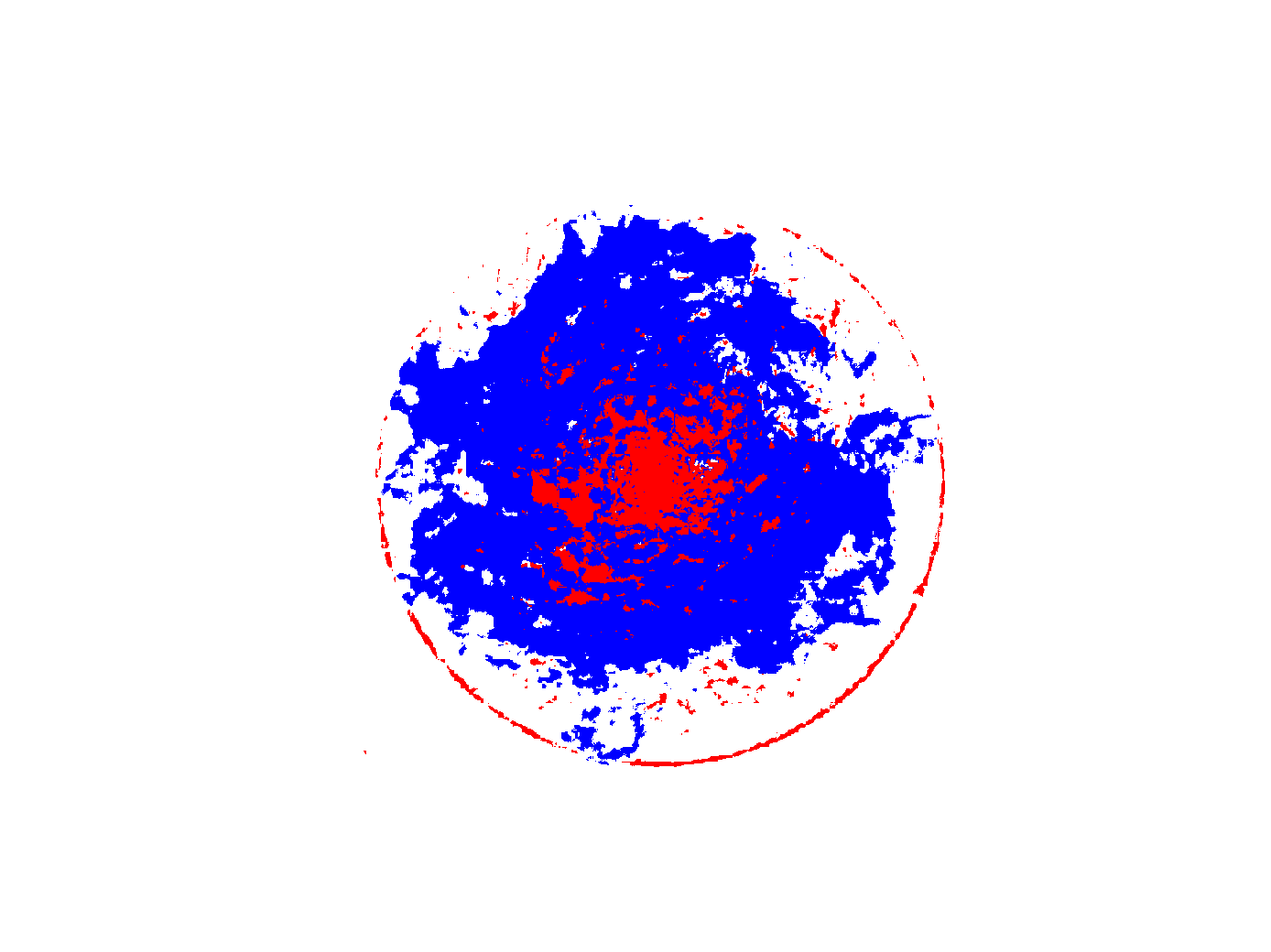

Supplement: S1 File — (ZIP) [file pone.0173647.s002.zip › S1_File/20160830_225416/O2_4.jpg.tiff]

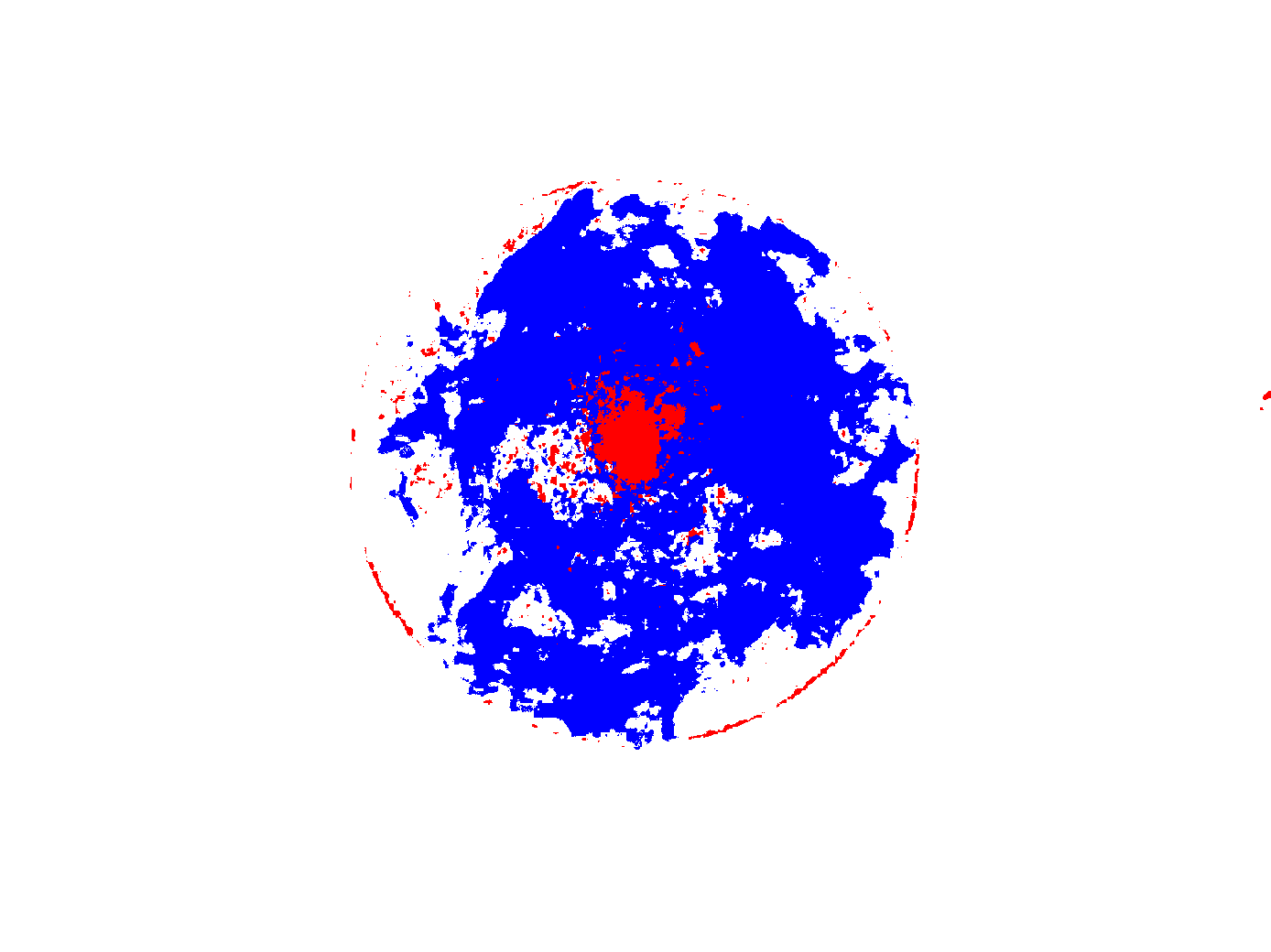

Supplement: S1 File — (ZIP) [file pone.0173647.s002.zip › S1_File/20160830_225416/O2_5.jpg.tiff]

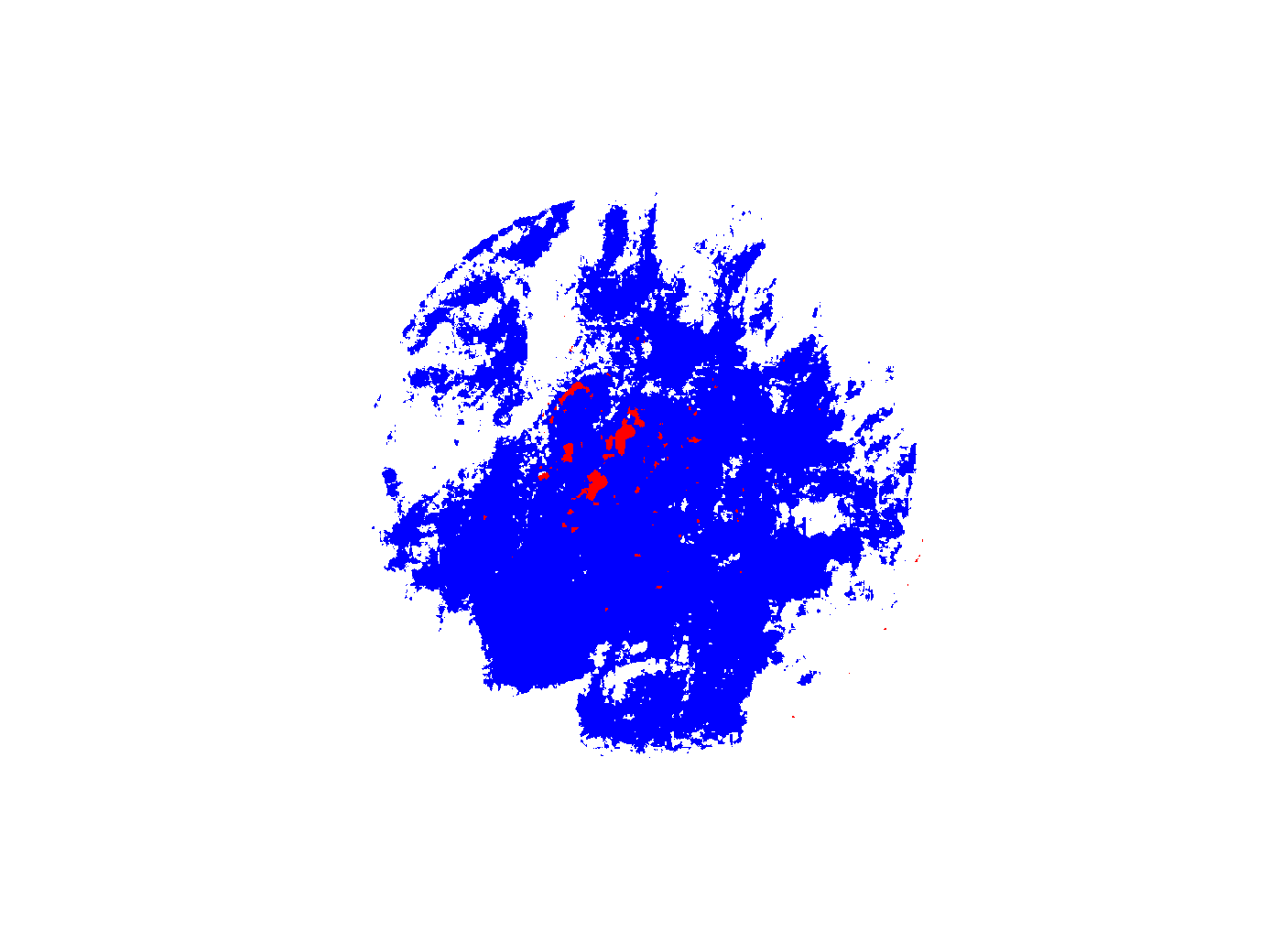

Supplement: S1 File — (ZIP) [file pone.0173647.s002.zip › S1_File/20160830_225416/O3_1.jpg.tiff]

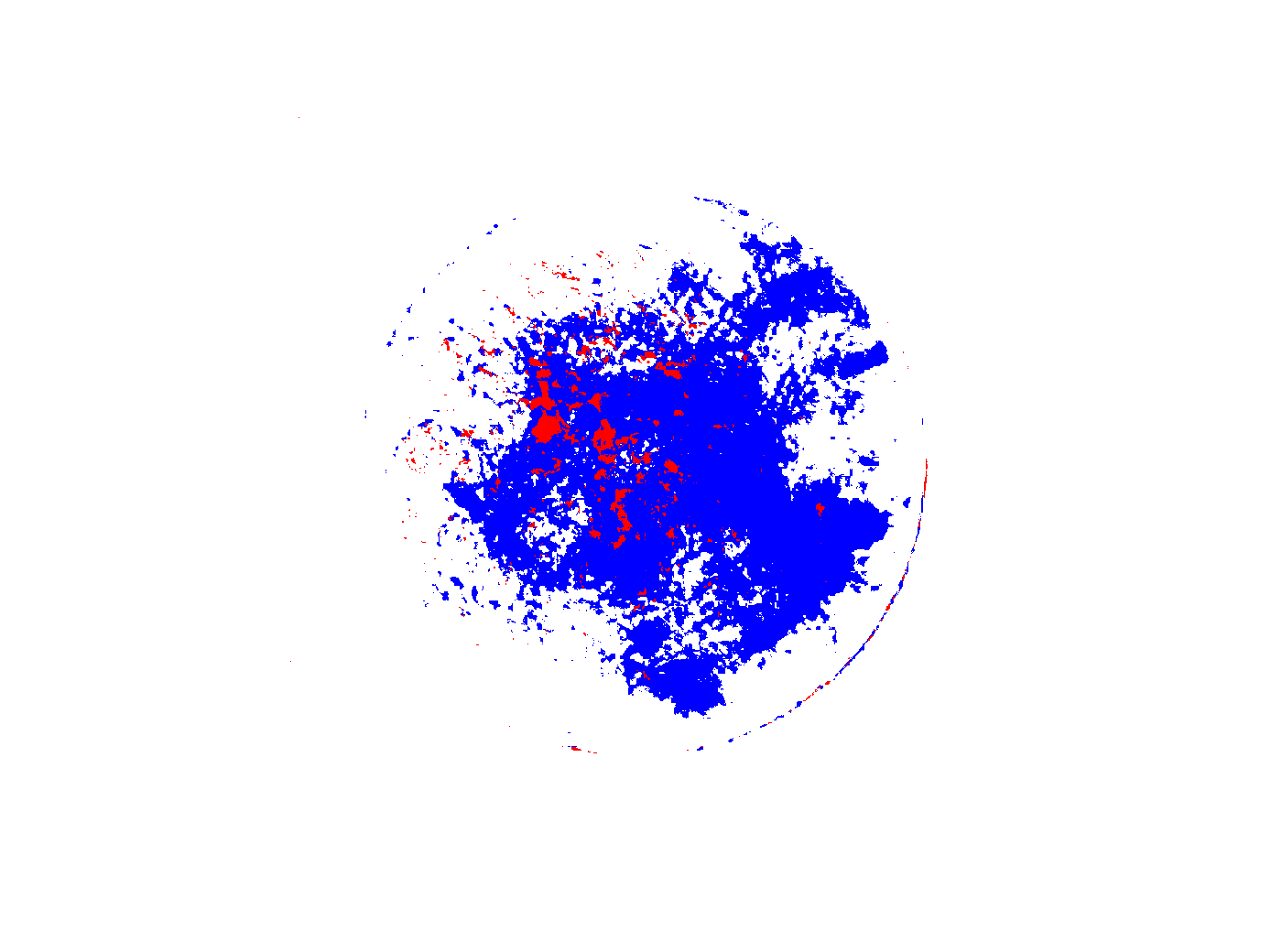

Supplement: S1 File — (ZIP) [file pone.0173647.s002.zip › S1_File/20160830_225416/O3_2.jpg.tiff]

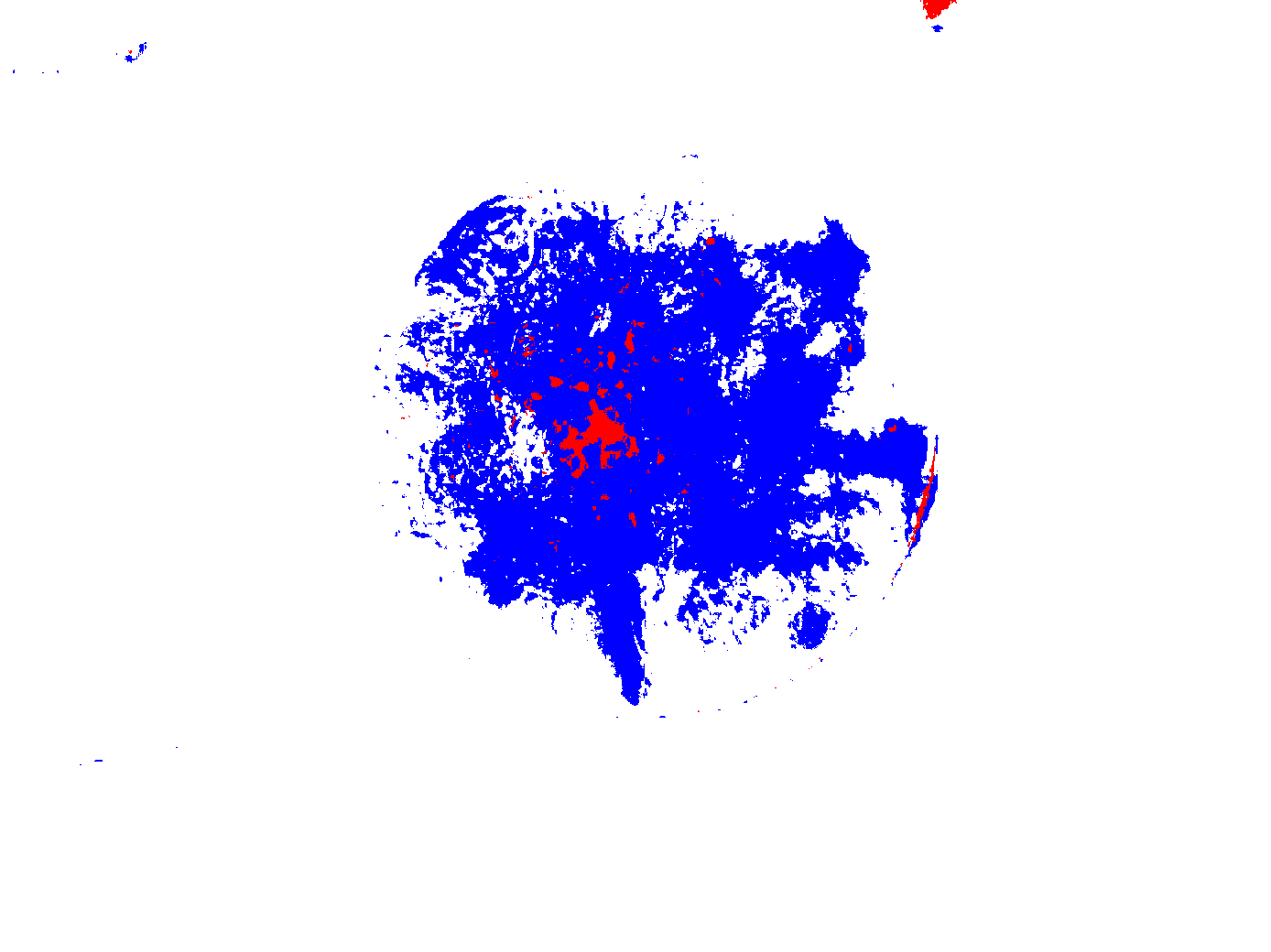

Supplement: S1 File — (ZIP) [file pone.0173647.s002.zip › S1_File/20160830_225416/O3_3.jpg.tiff]

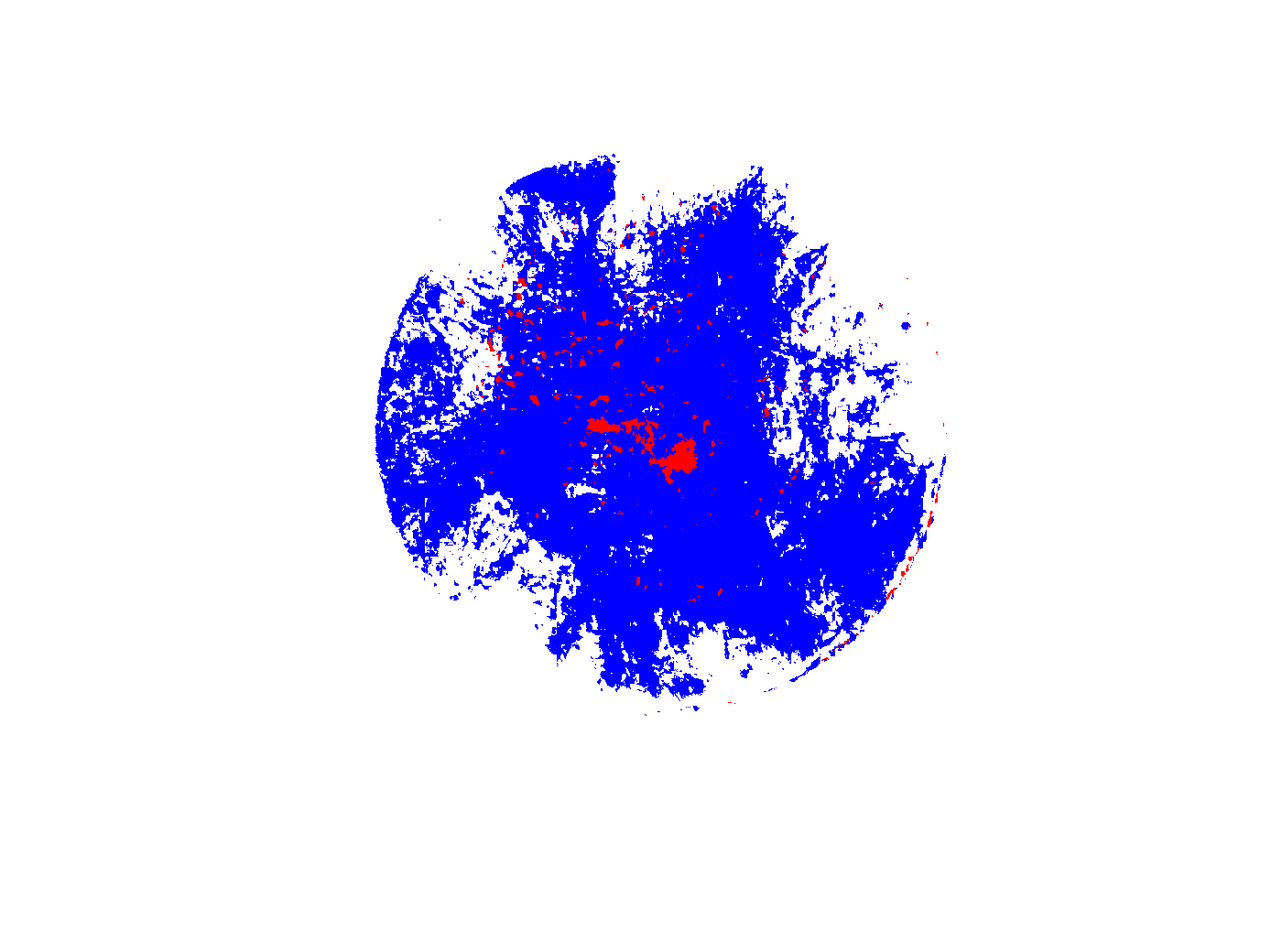

Supplement: S1 File — (ZIP) [file pone.0173647.s002.zip › S1_File/20160830_225416/O3_4.jpg.tiff]

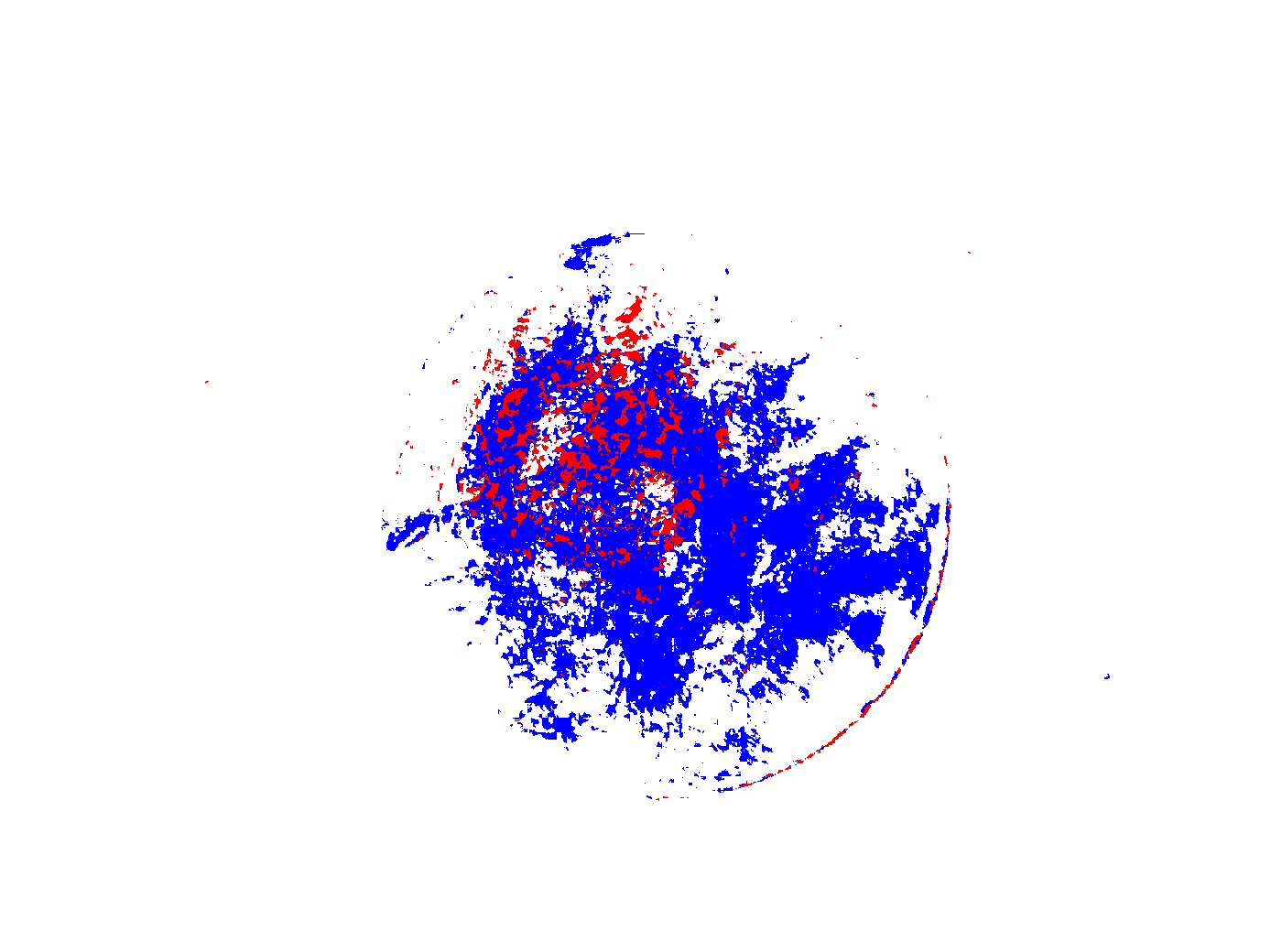

Supplement: S1 File — (ZIP) [file pone.0173647.s002.zip › S1_File/20160830_225416/O3_5.jpg.tiff]

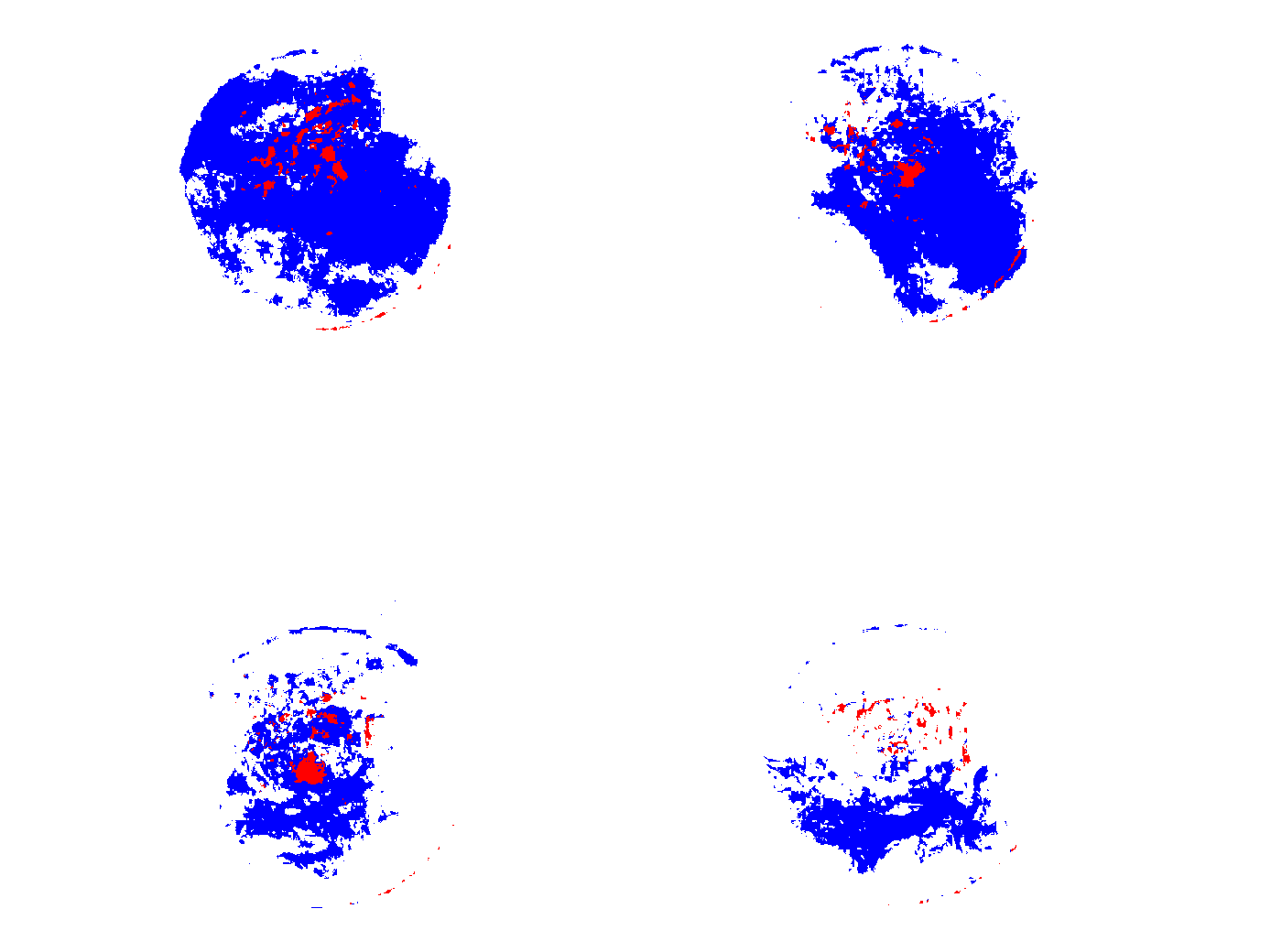

Supplement: S1 File — (ZIP) [file pone.0173647.s002.zip › S1_File/20160830_225416/O4_1.jpg.tiff]

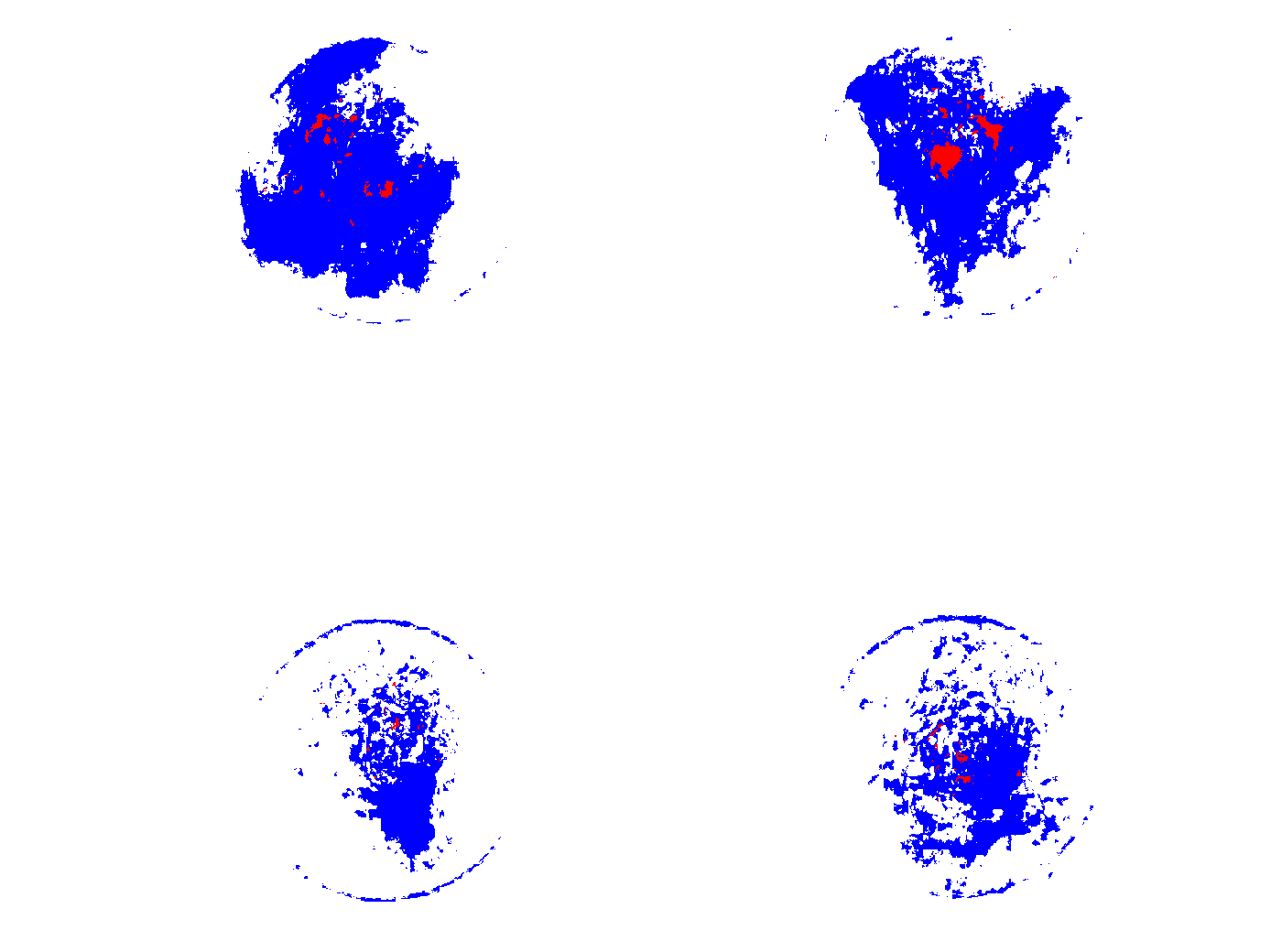

Supplement: S1 File — (ZIP) [file pone.0173647.s002.zip › S1_File/20160830_225416/O4_2.jpg.tiff]

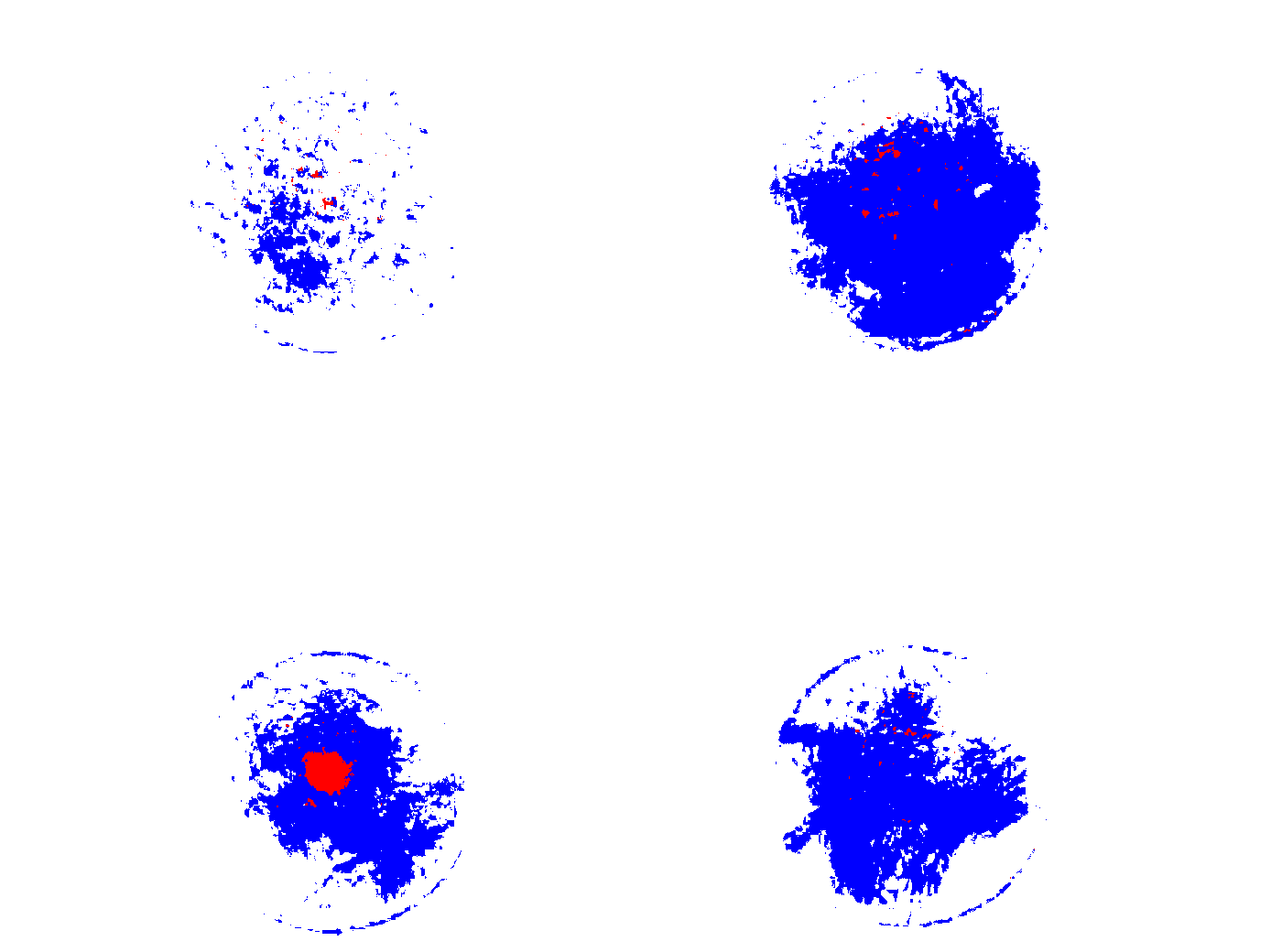

Supplement: S1 File — (ZIP) [file pone.0173647.s002.zip › S1_File/20160830_225416/O4_3.jpg.tiff]

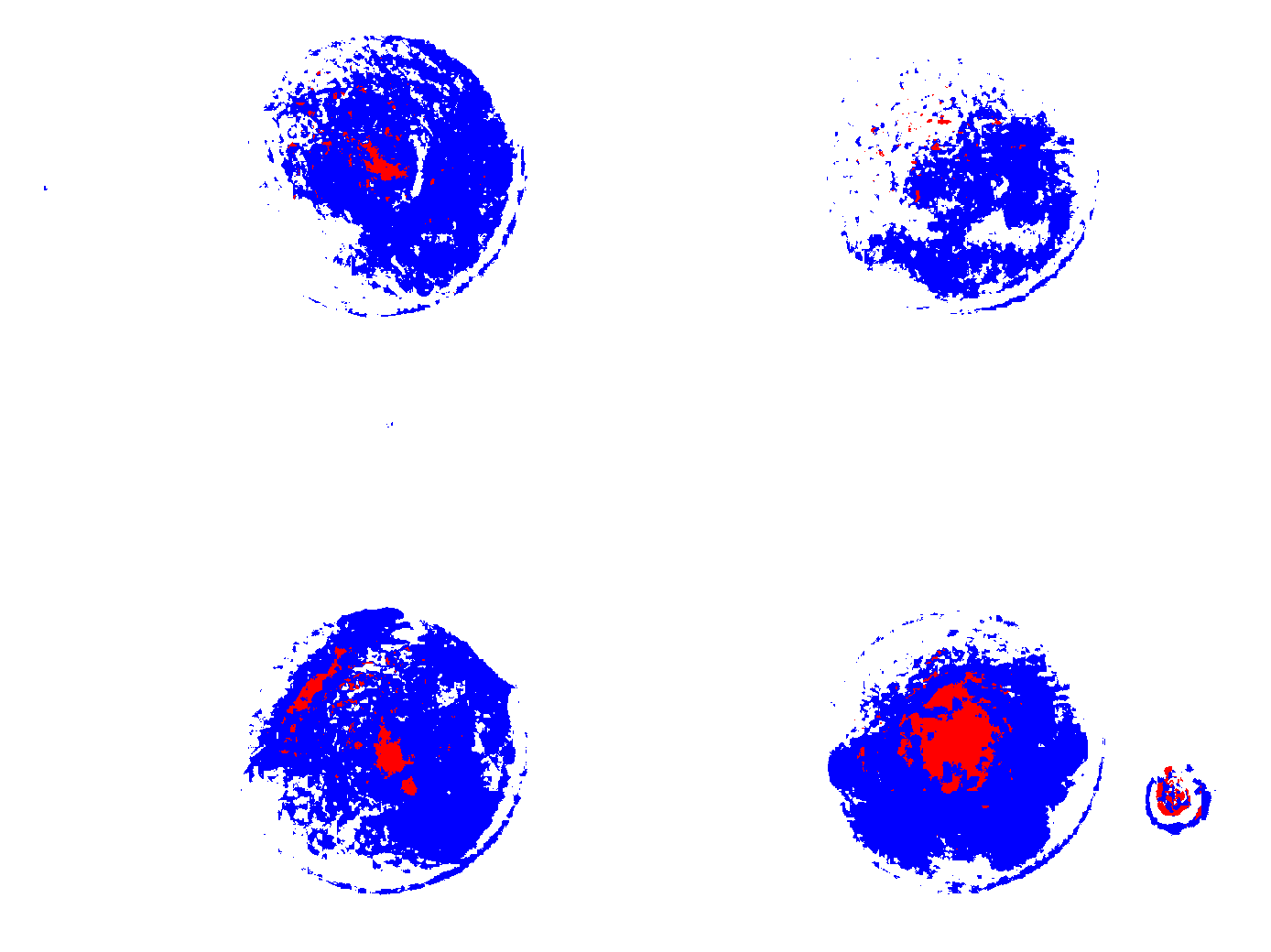

Supplement: S1 File — (ZIP) [file pone.0173647.s002.zip › S1_File/20160830_225416/O4_4.jpg.tiff]

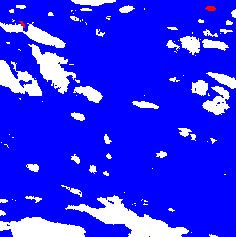

Supplement: S1 File — (ZIP) [file pone.0173647.s002.zip › S1_File/20160830_225416/OC_1.jpg.tiff]

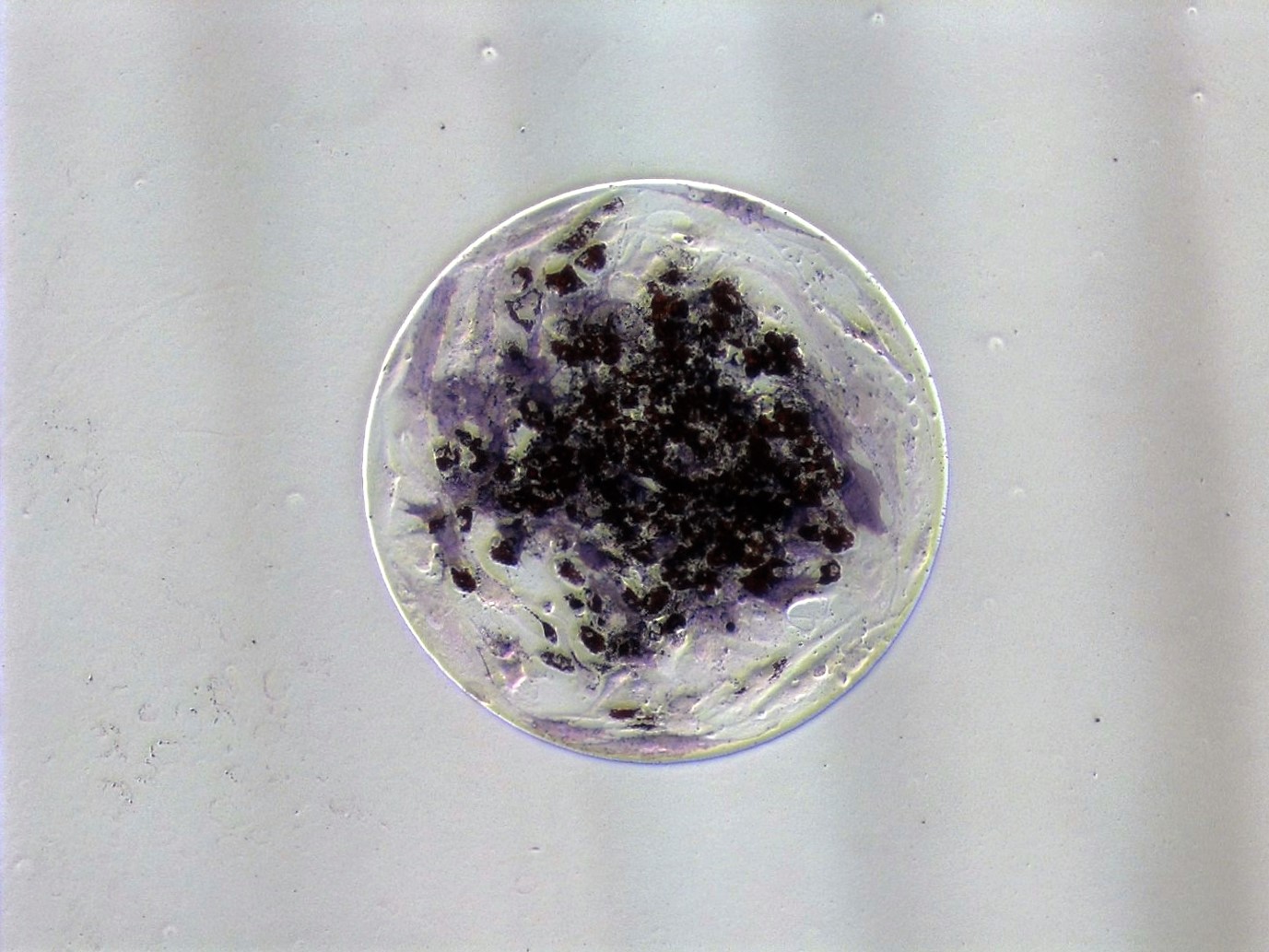

Supplement: S1 File — (ZIP) [file pone.0173647.s002.zip › S1_File/targets/A2_1.jpg]

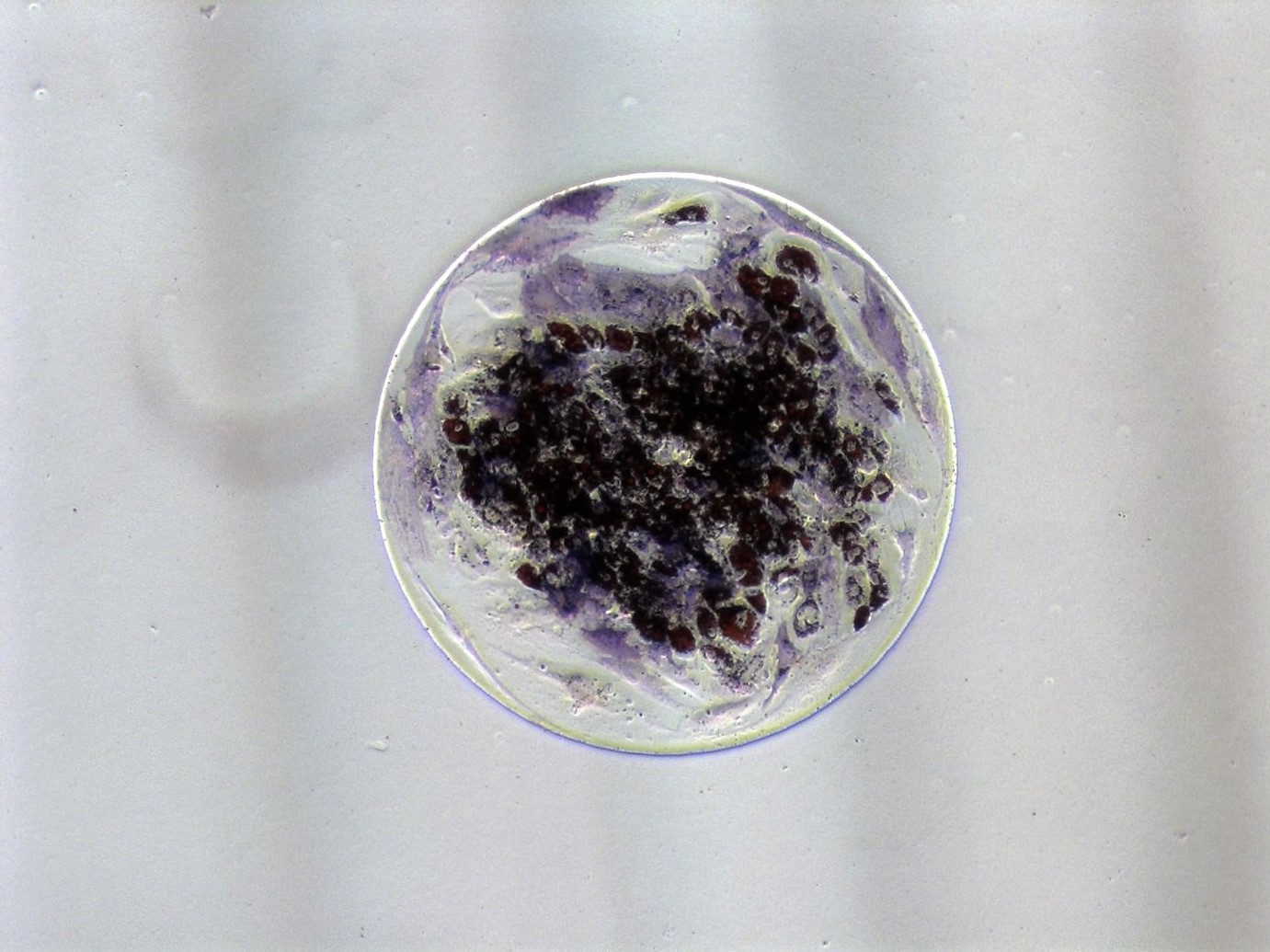

Supplement: S1 File — (ZIP) [file pone.0173647.s002.zip › S1_File/targets/A2_2.jpg]

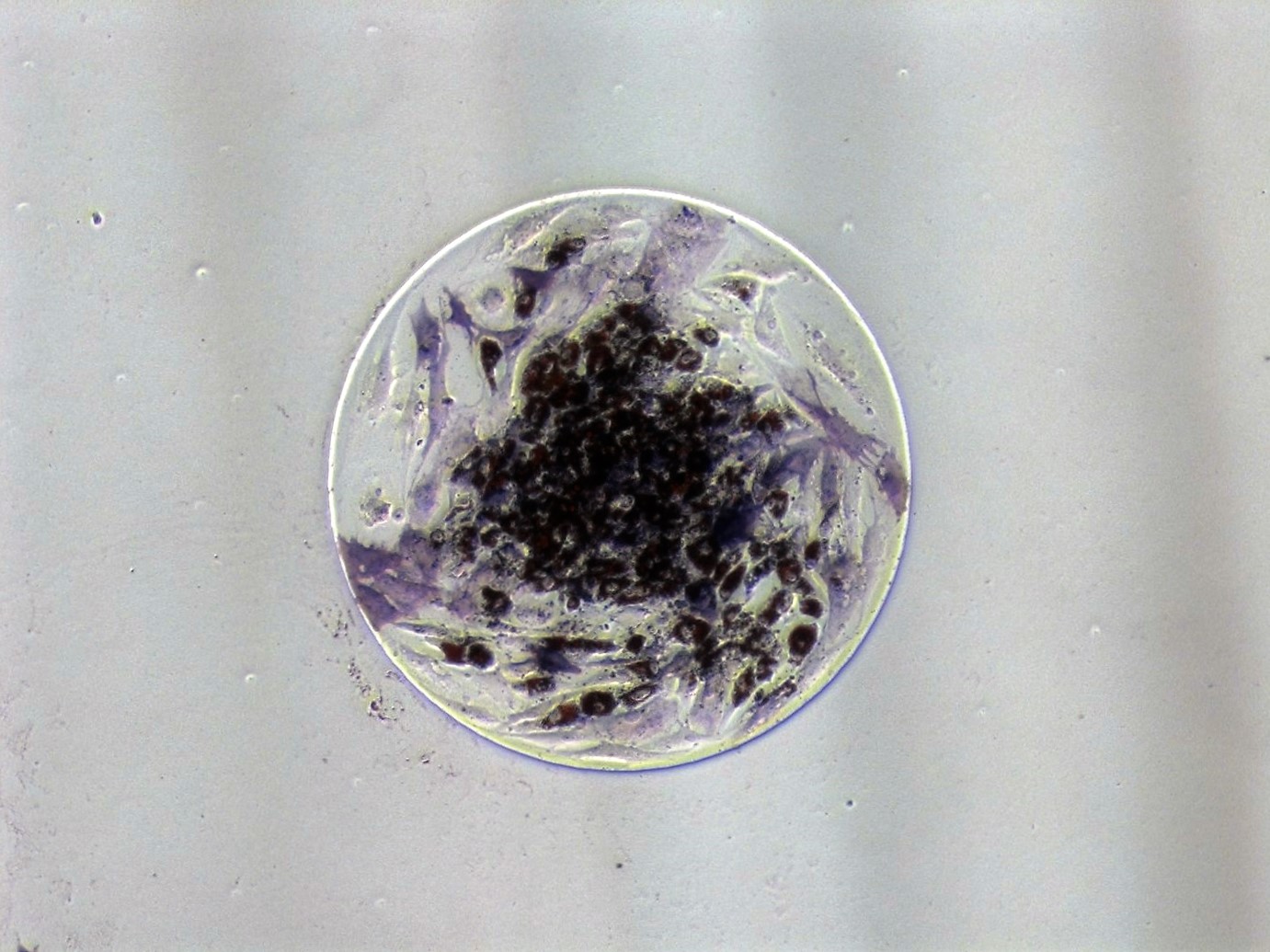

Supplement: S1 File — (ZIP) [file pone.0173647.s002.zip › S1_File/targets/A2_3.jpg]

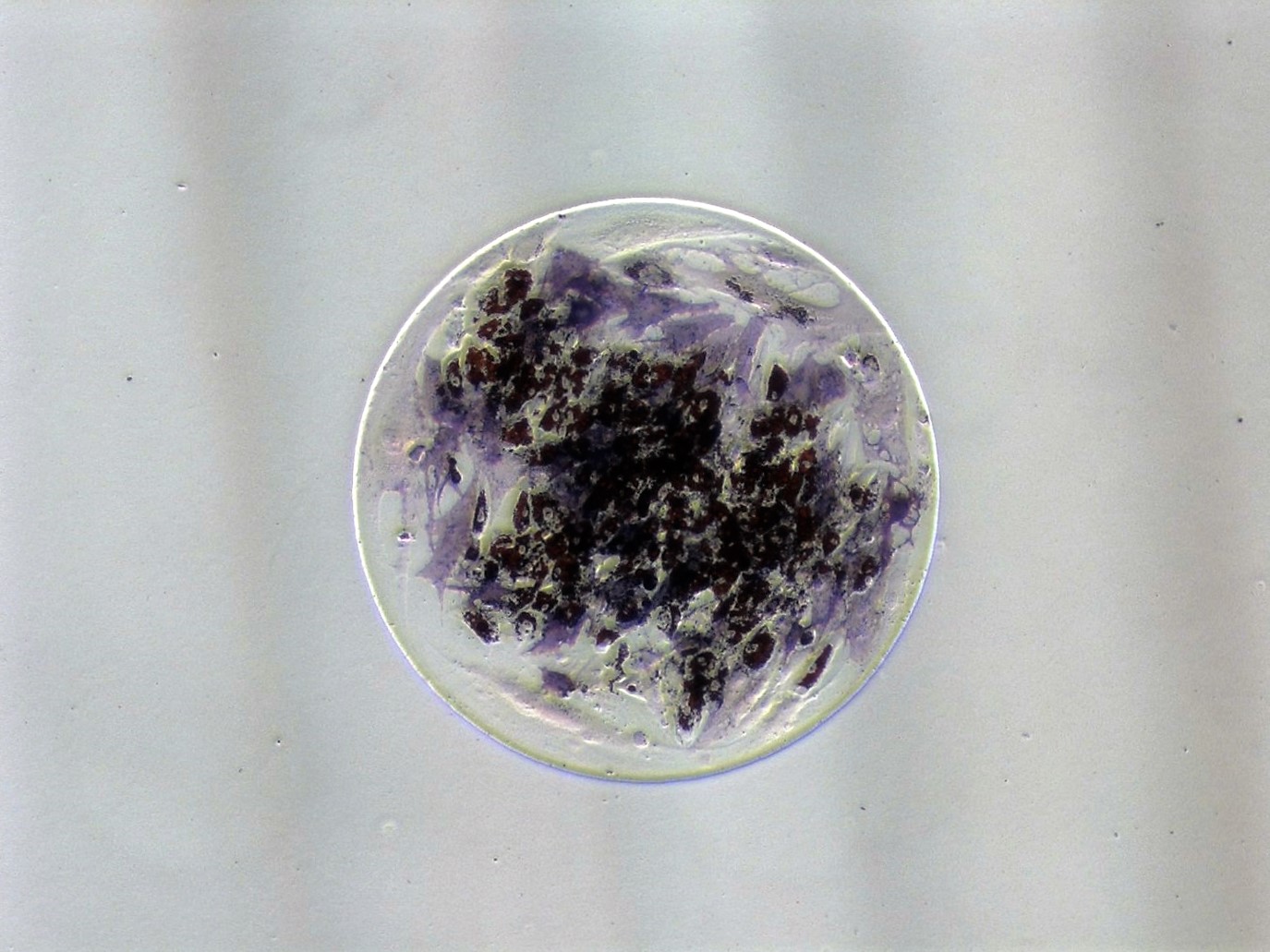

Supplement: S1 File — (ZIP) [file pone.0173647.s002.zip › S1_File/targets/A2_4.jpg]

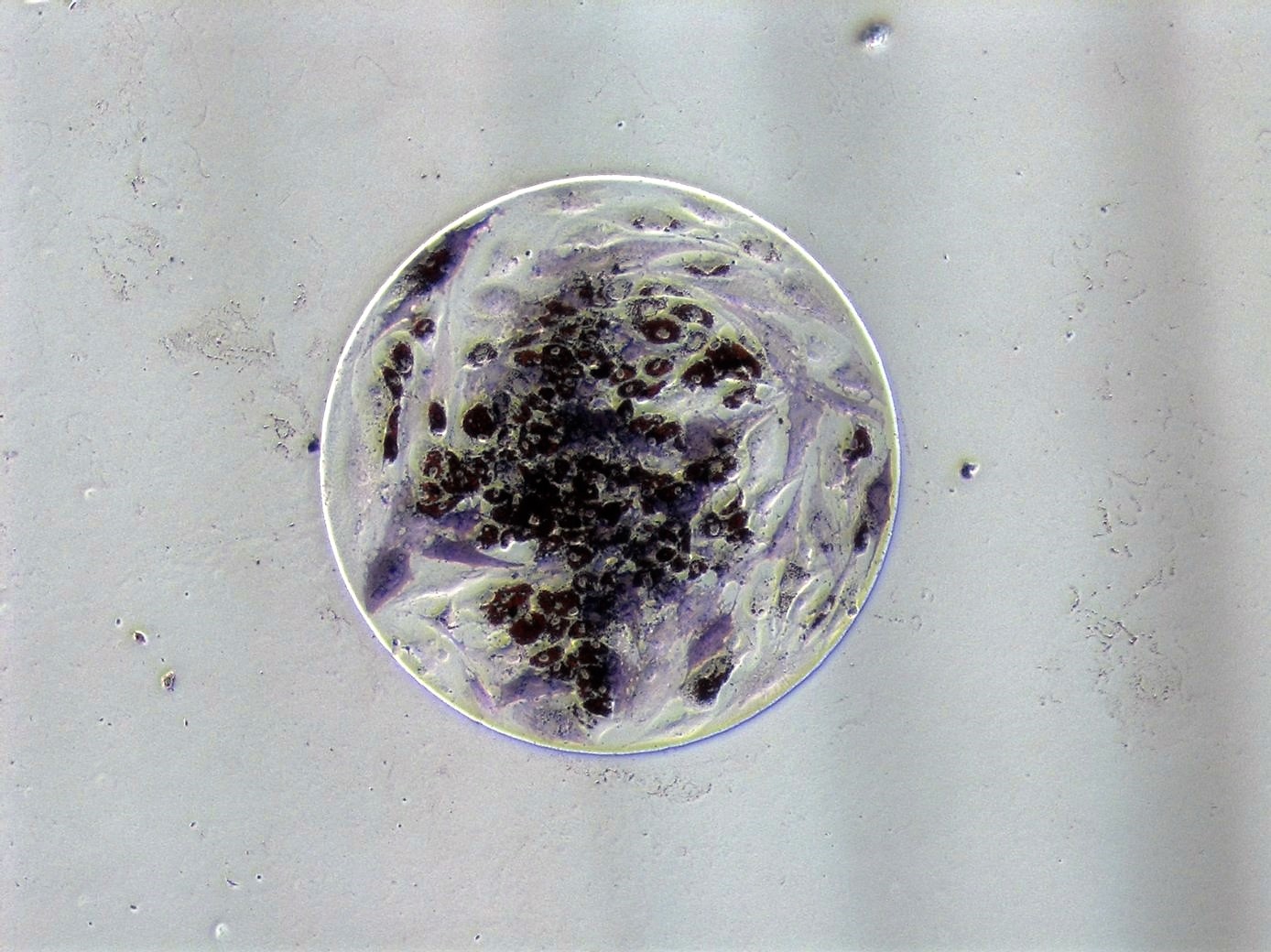

Supplement: S1 File — (ZIP) [file pone.0173647.s002.zip › S1_File/targets/A2_5.jpg]

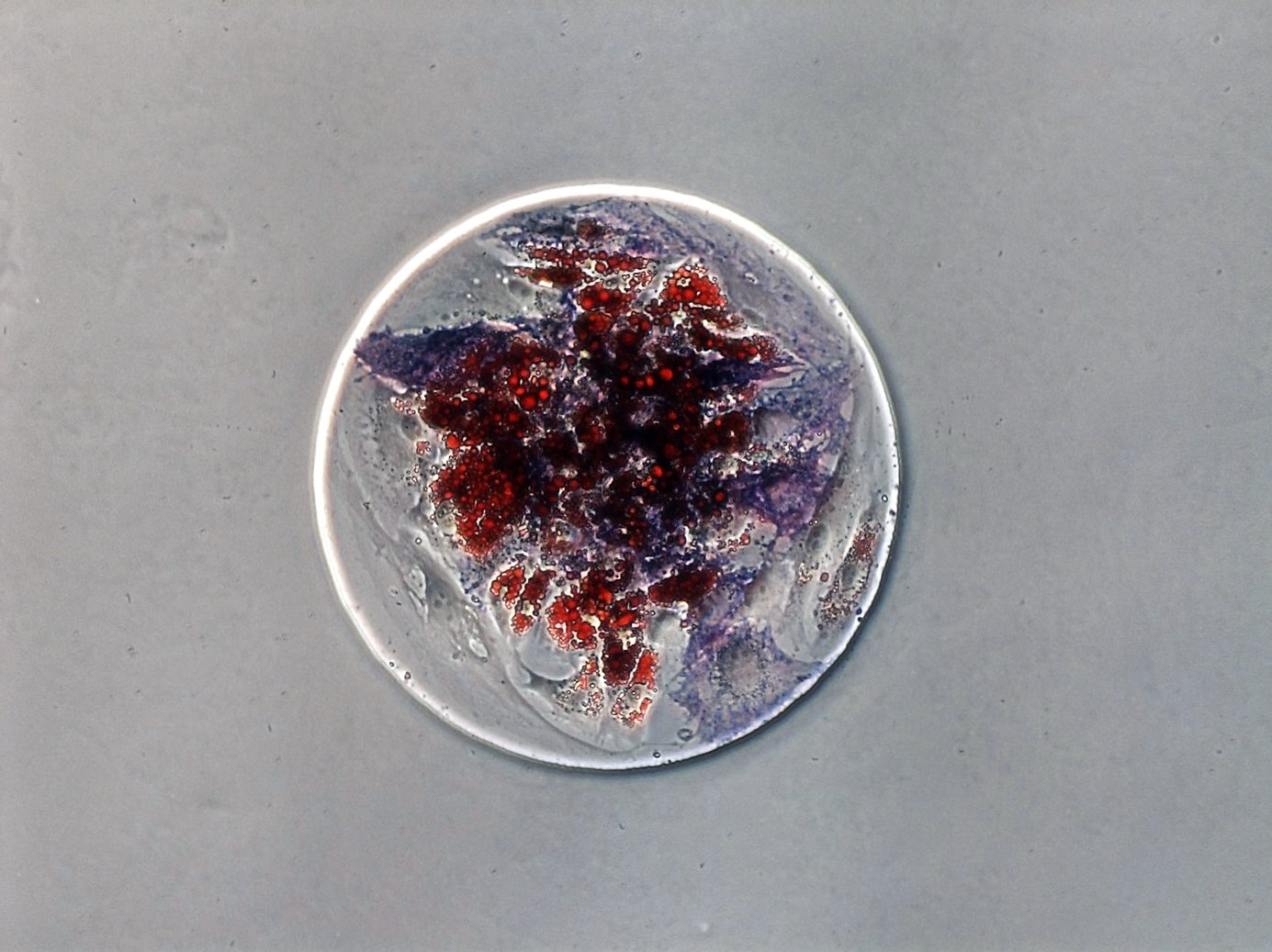

Supplement: S1 File — (ZIP) [file pone.0173647.s002.zip › S1_File/targets/A3_1.jpg]

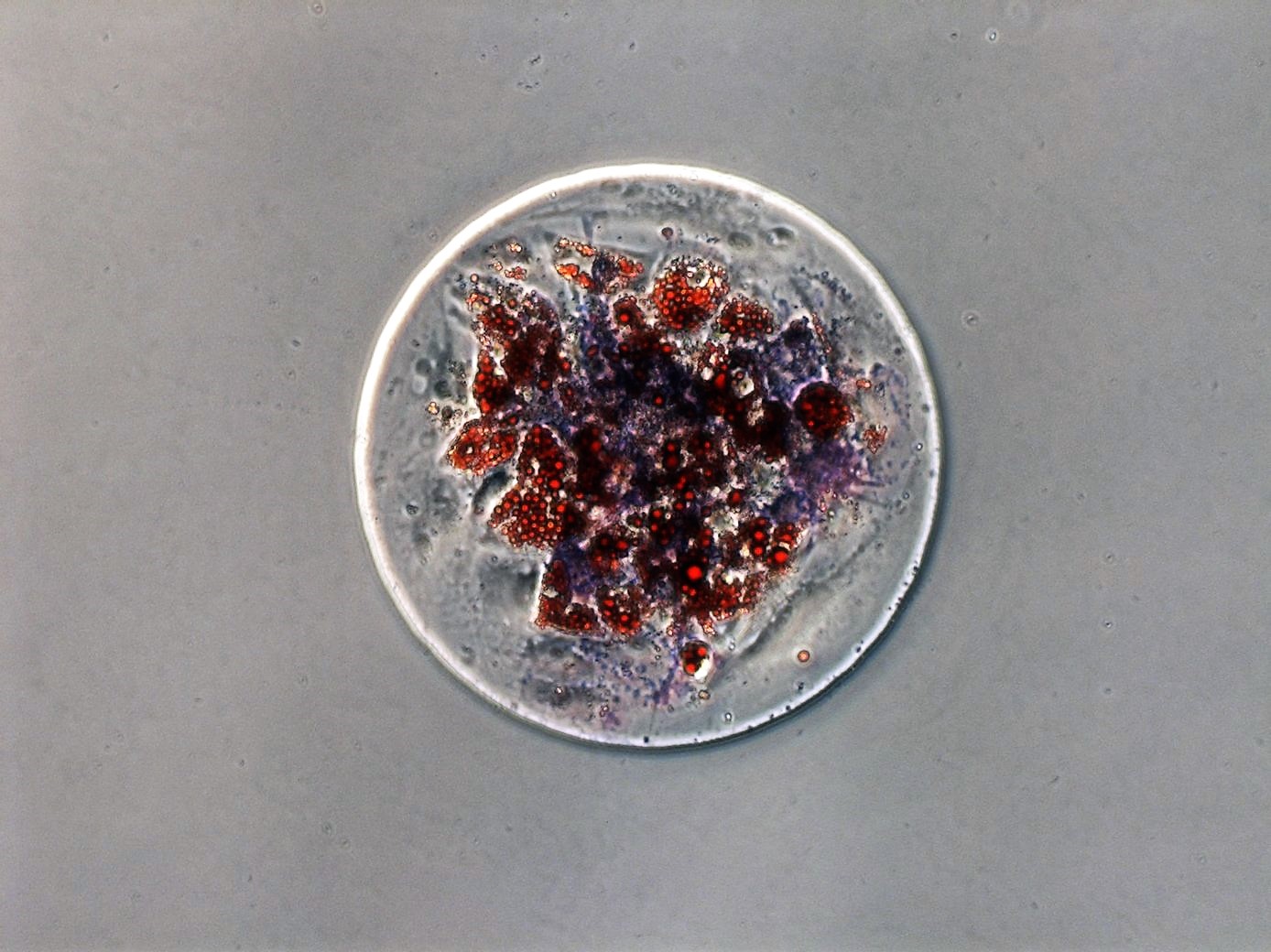

Supplement: S1 File — (ZIP) [file pone.0173647.s002.zip › S1_File/targets/A3_2.jpg]

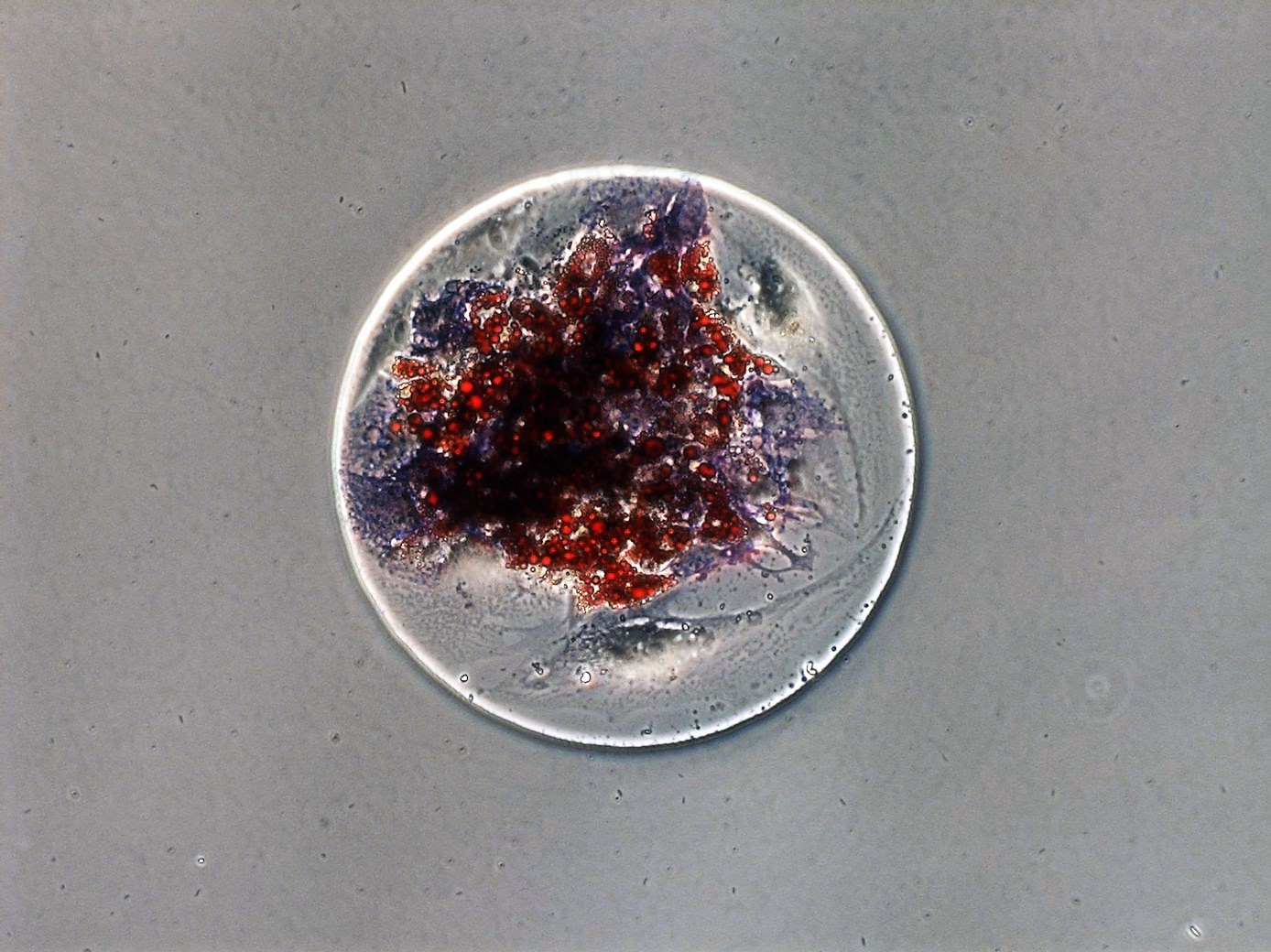

Supplement: S1 File — (ZIP) [file pone.0173647.s002.zip › S1_File/targets/A3_3.jpg]

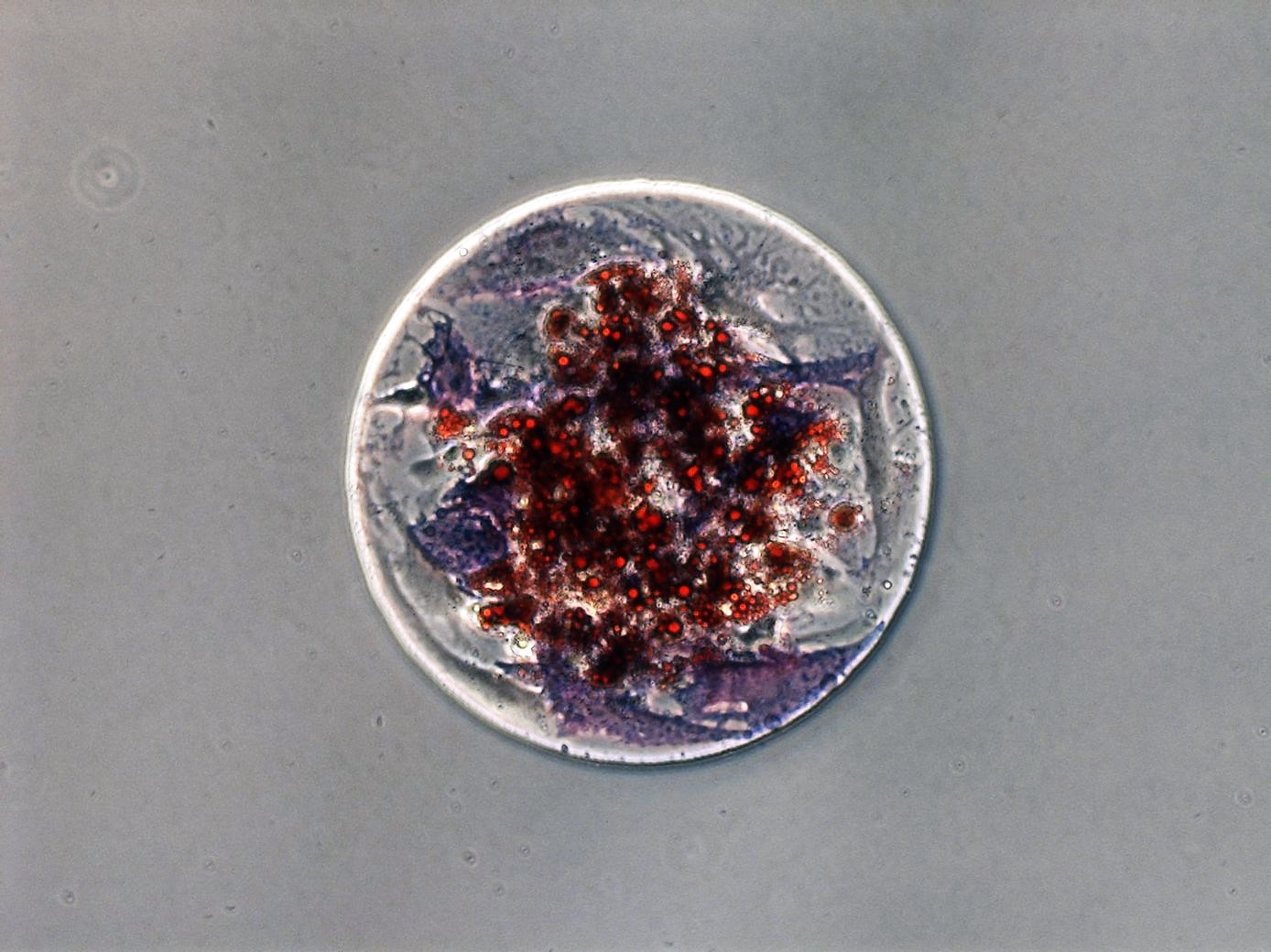

Supplement: S1 File — (ZIP) [file pone.0173647.s002.zip › S1_File/targets/A3_4.jpg]

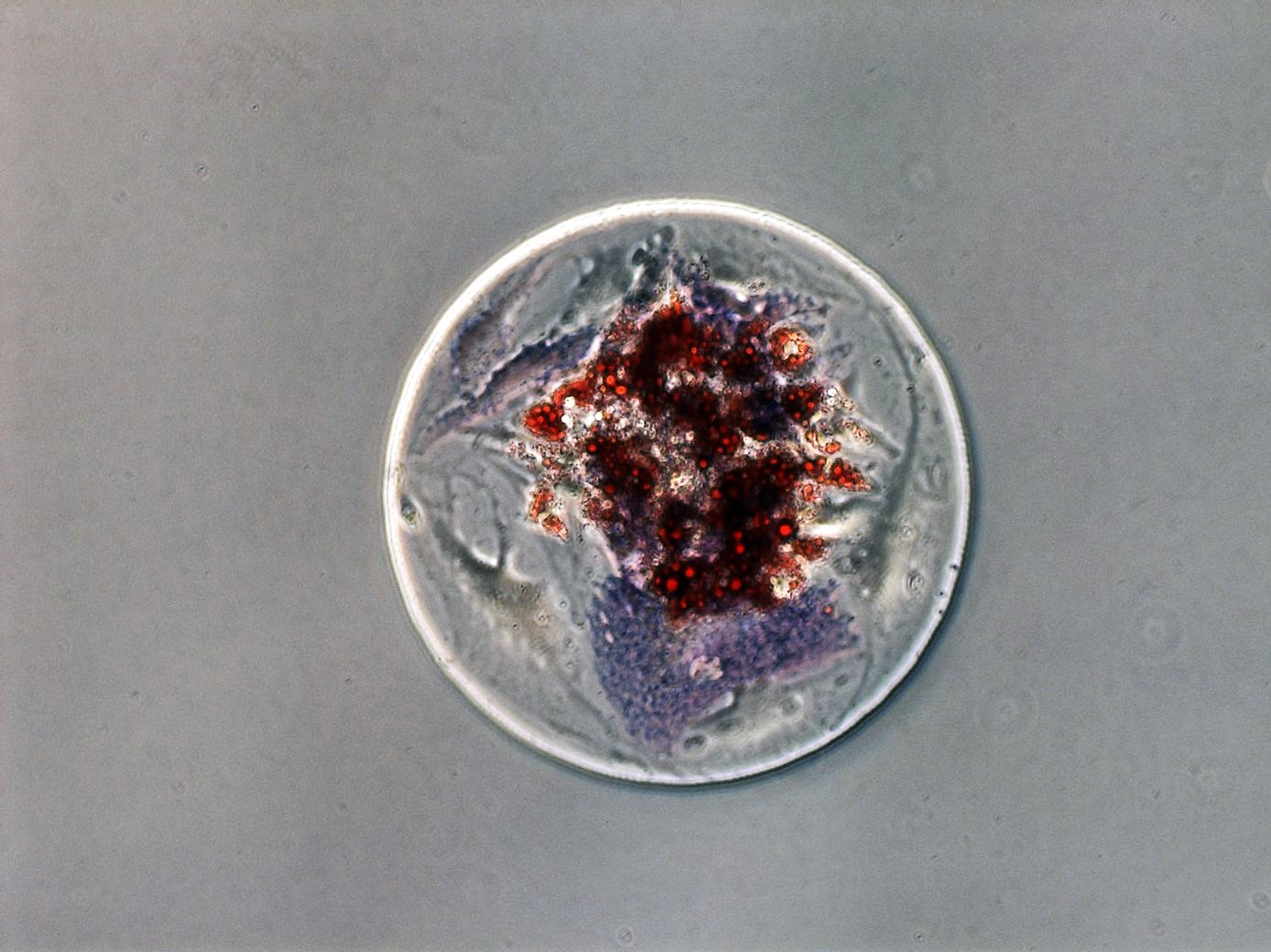

Supplement: S1 File — (ZIP) [file pone.0173647.s002.zip › S1_File/targets/A3_5.jpg]

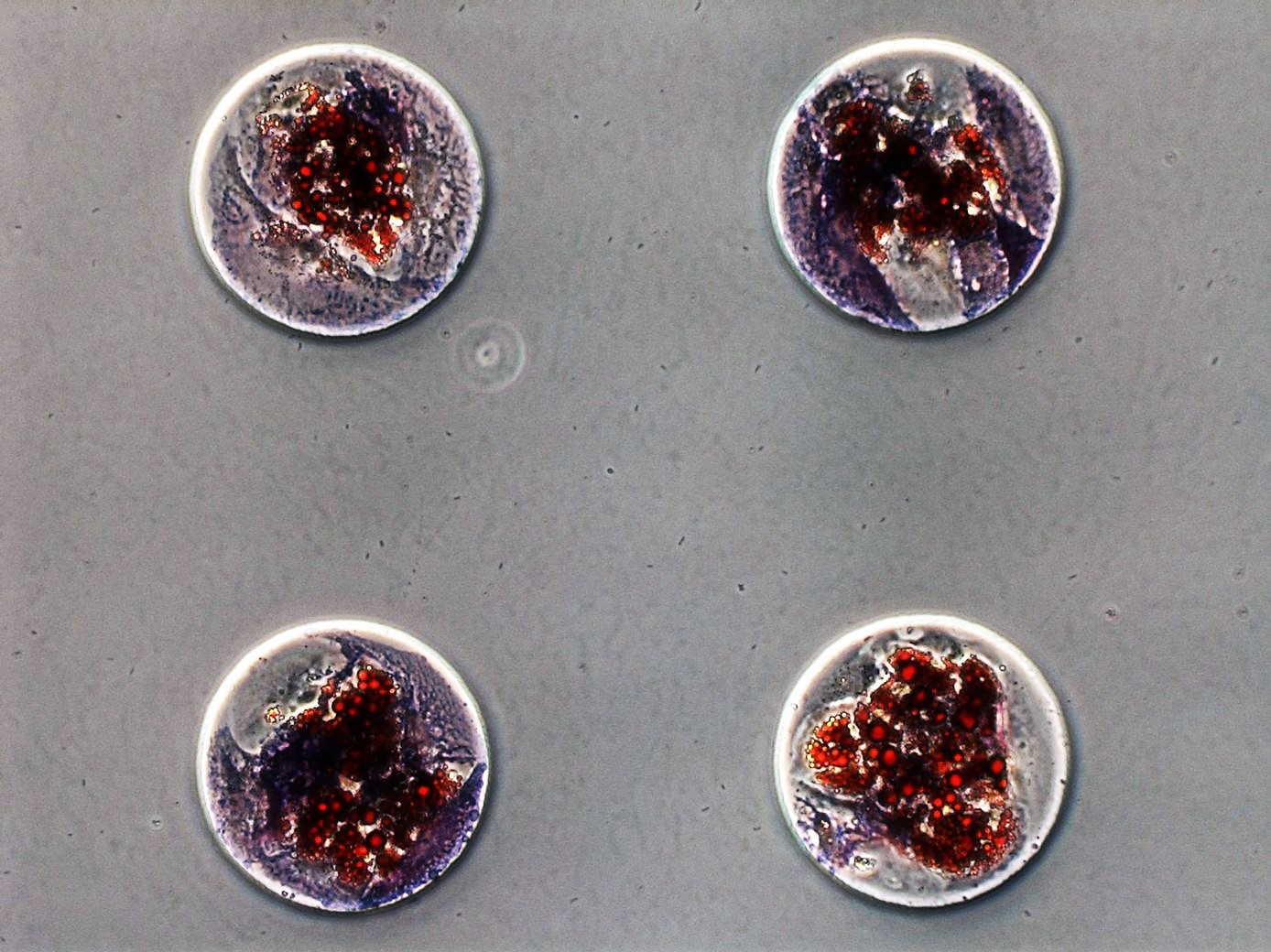

Supplement: S1 File — (ZIP) [file pone.0173647.s002.zip › S1_File/targets/A4_1.jpg]

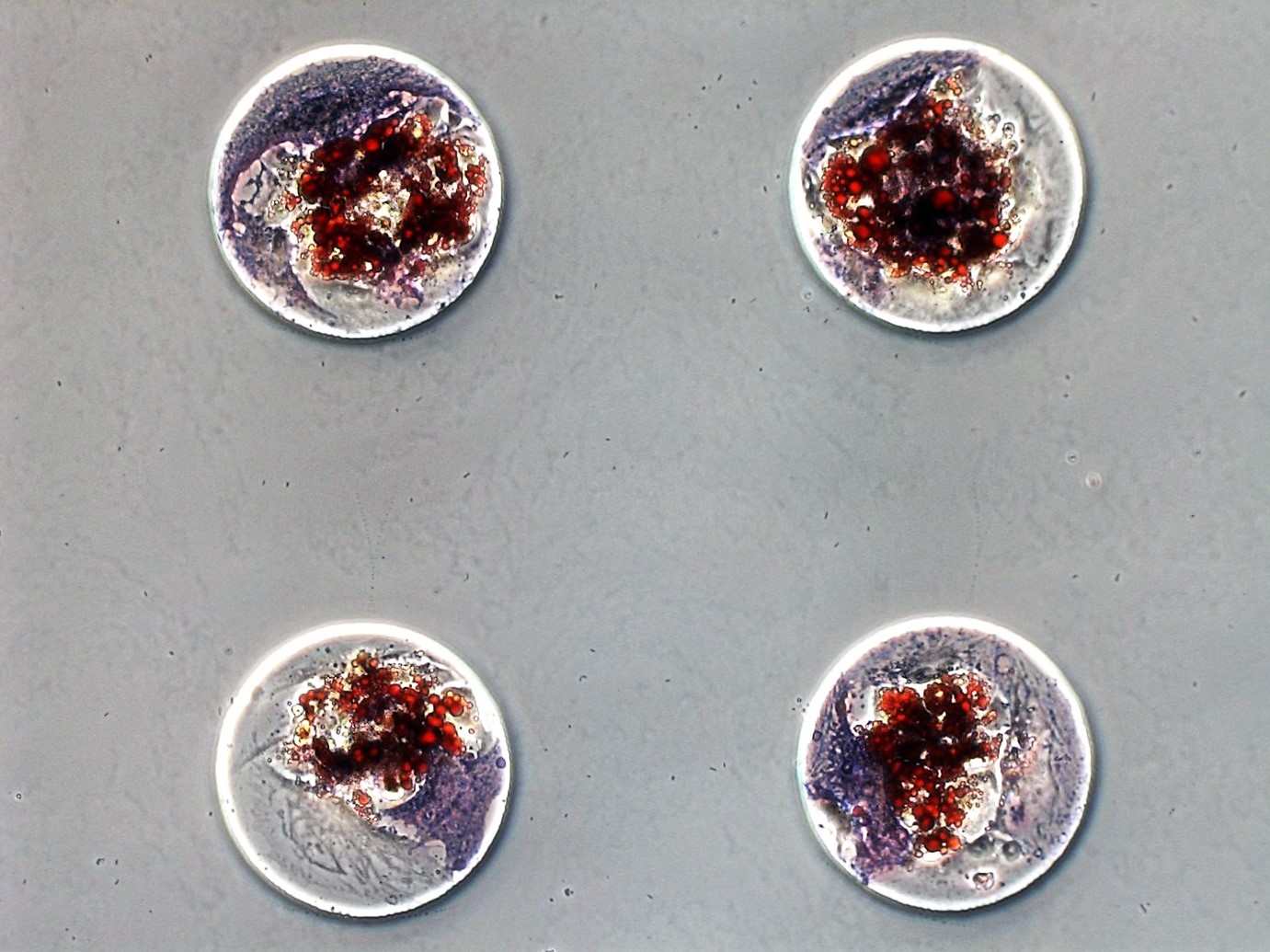

Supplement: S1 File — (ZIP) [file pone.0173647.s002.zip › S1_File/targets/A4_2.jpg]

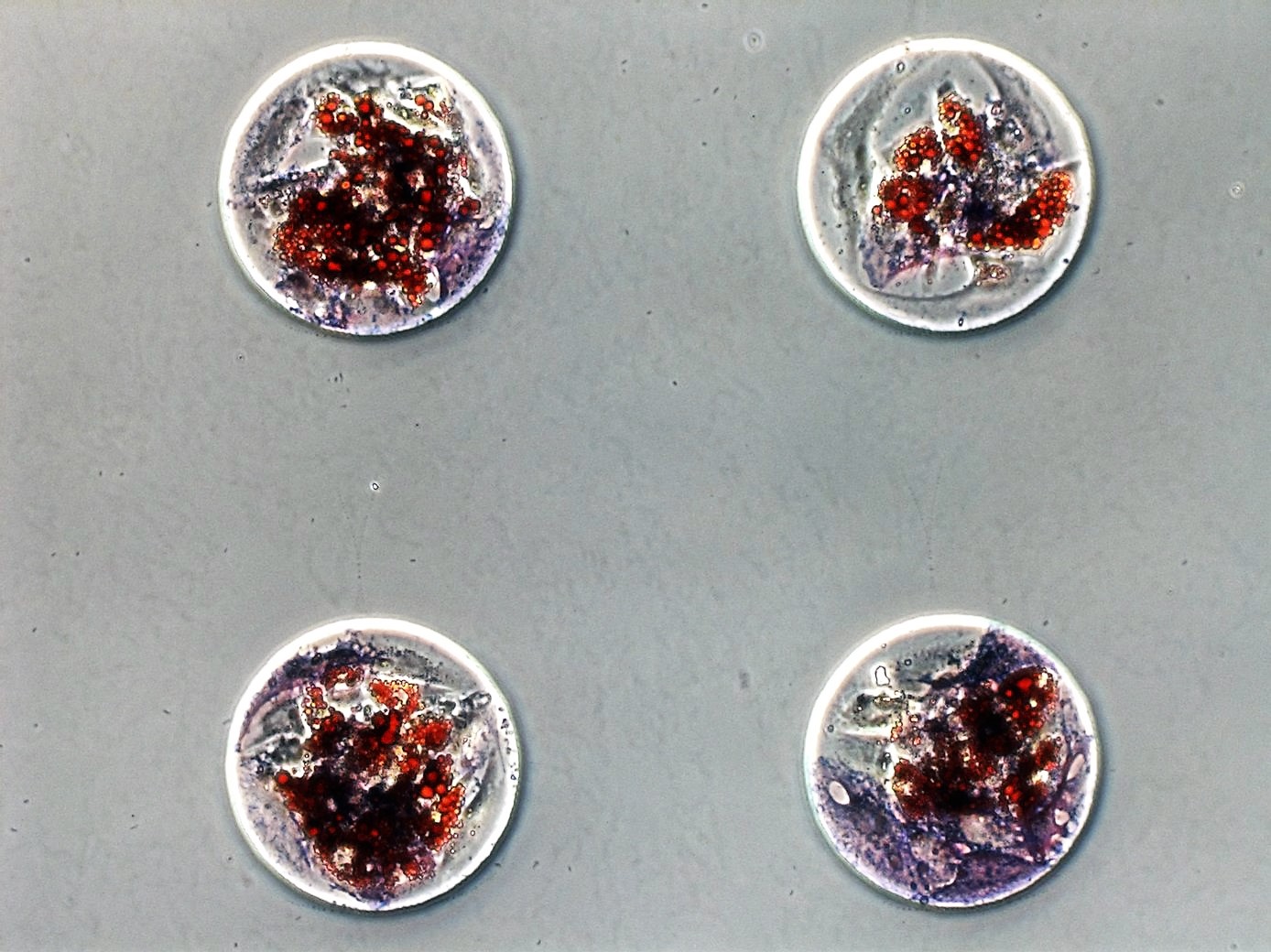

Supplement: S1 File — (ZIP) [file pone.0173647.s002.zip › S1_File/targets/A4_3.jpg]

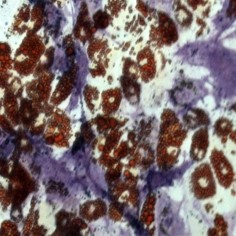

Supplement: S1 File — (ZIP) [file pone.0173647.s002.zip › S1_File/targets/AC_1.jpg]

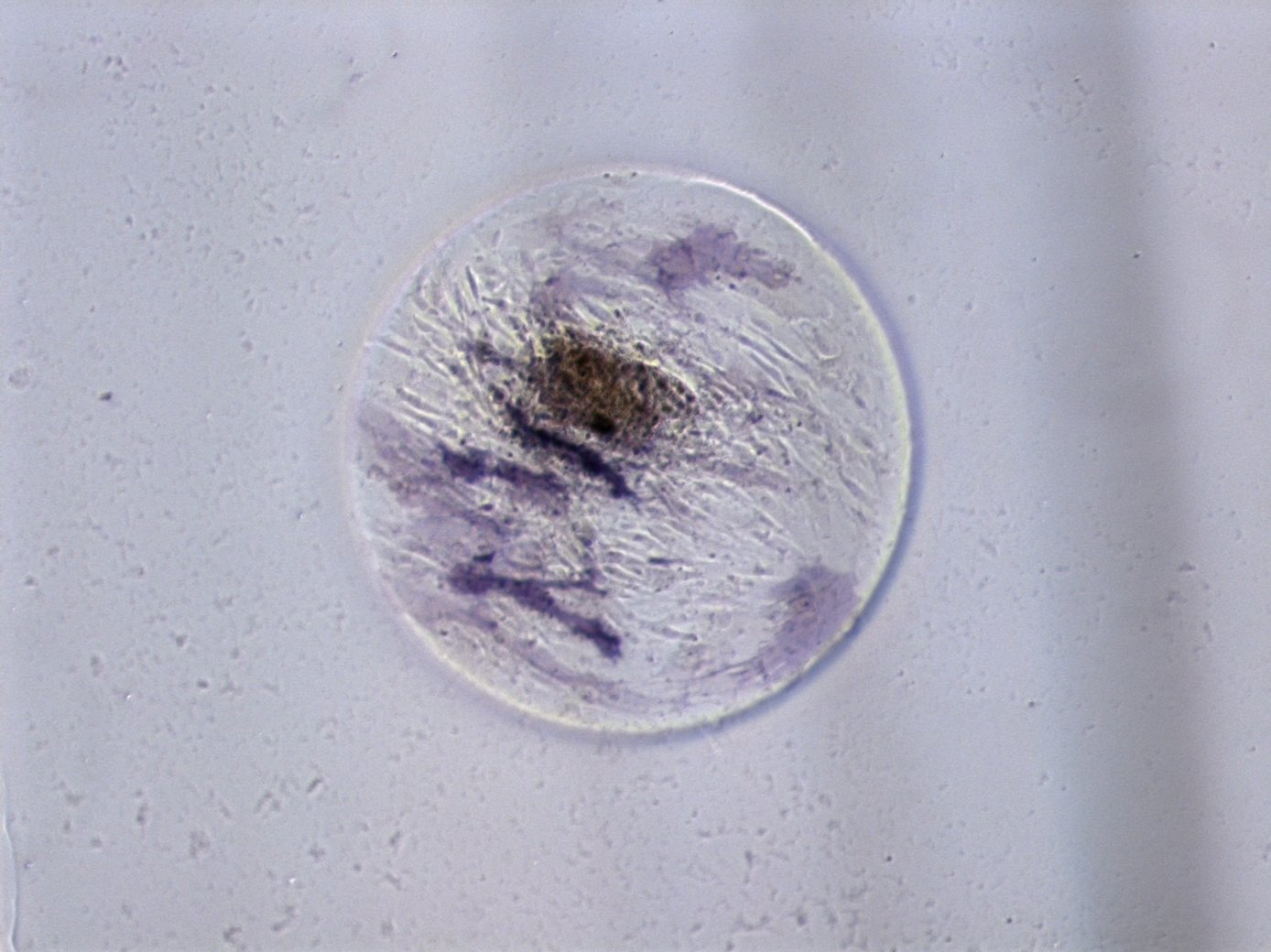

Supplement: S1 File — (ZIP) [file pone.0173647.s002.zip › S1_File/targets/G2_1.jpg]

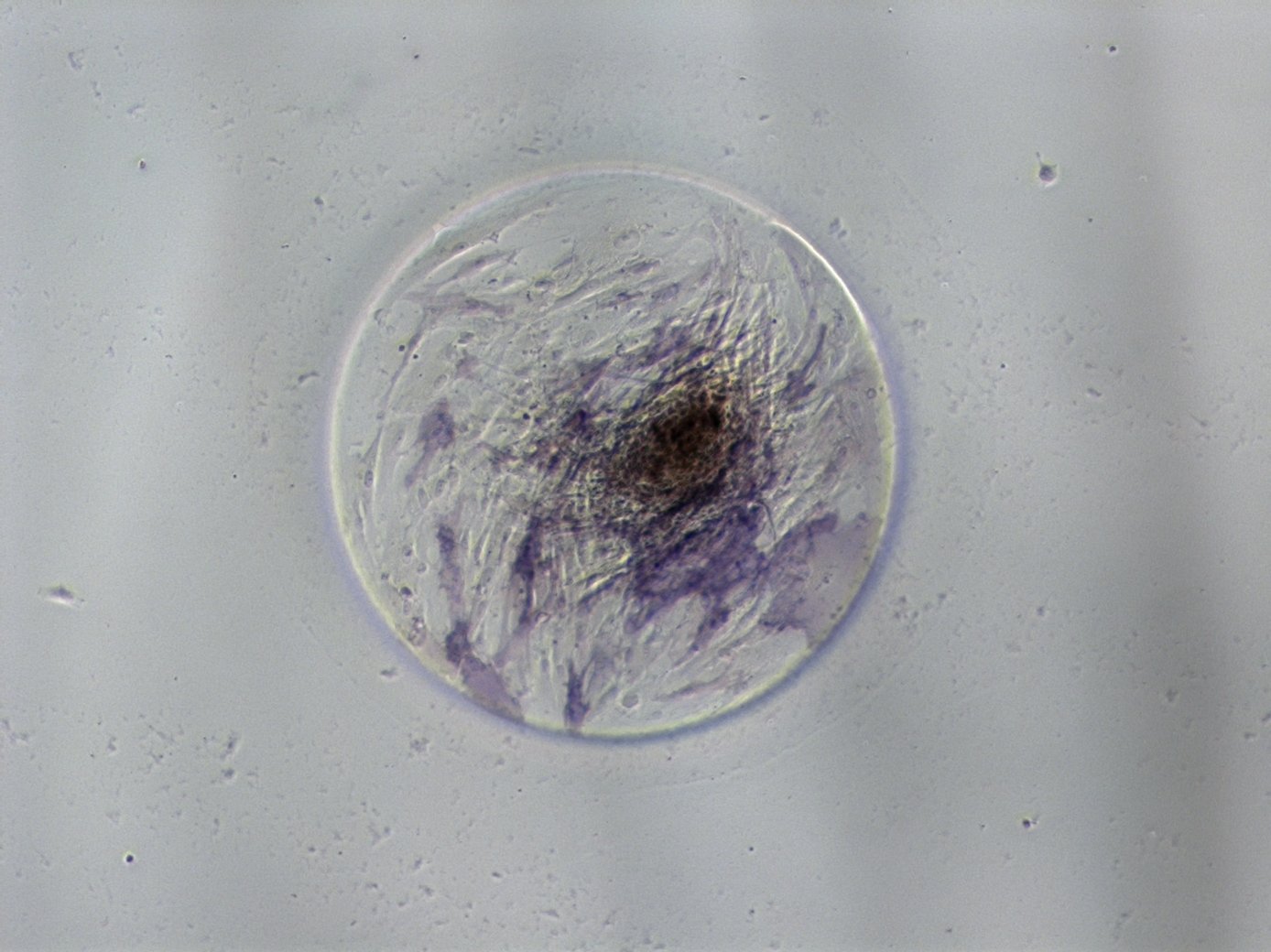

Supplement: S1 File — (ZIP) [file pone.0173647.s002.zip › S1_File/targets/G2_2.jpg]

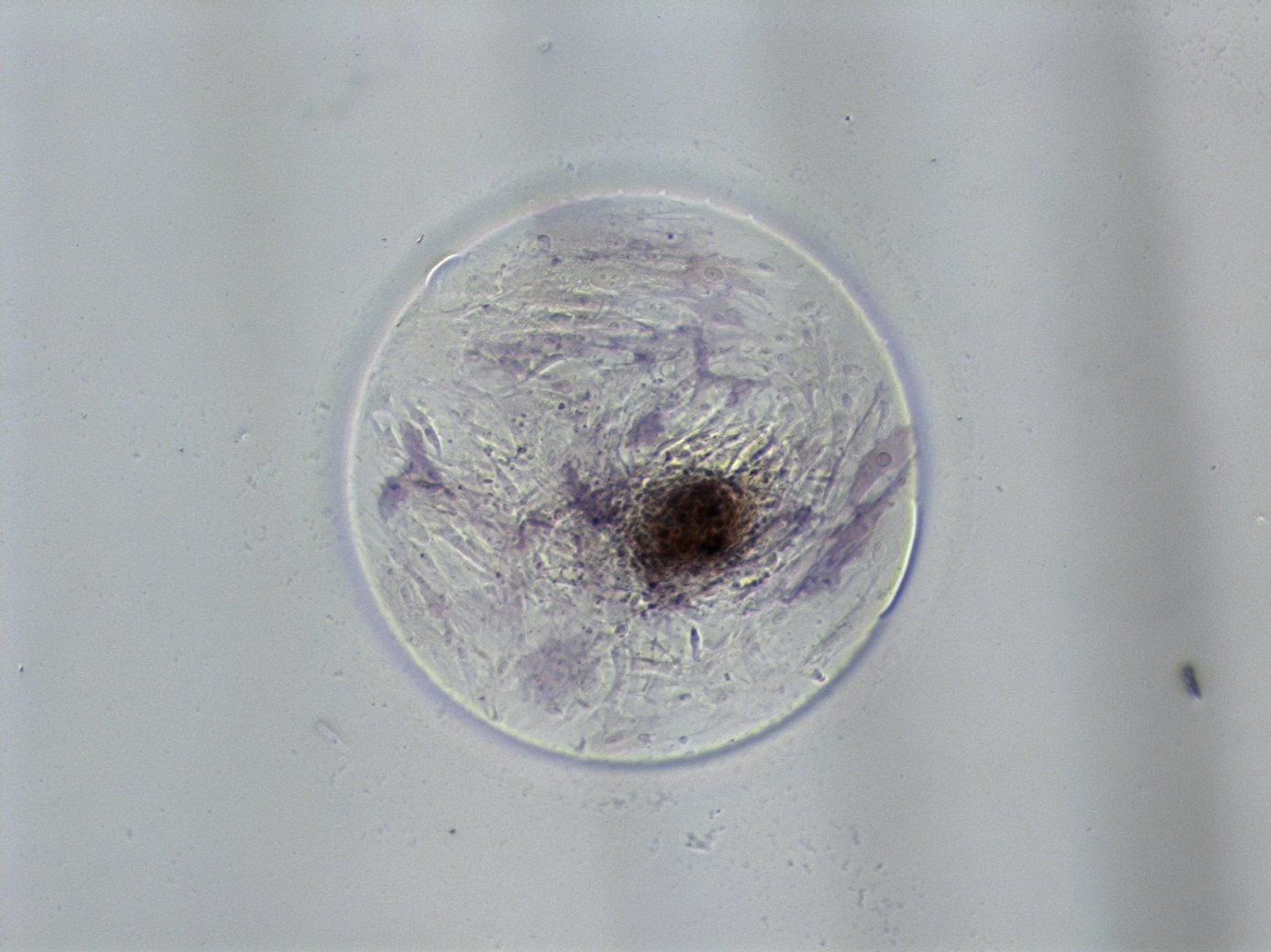

Supplement: S1 File — (ZIP) [file pone.0173647.s002.zip › S1_File/targets/G2_3.jpg]

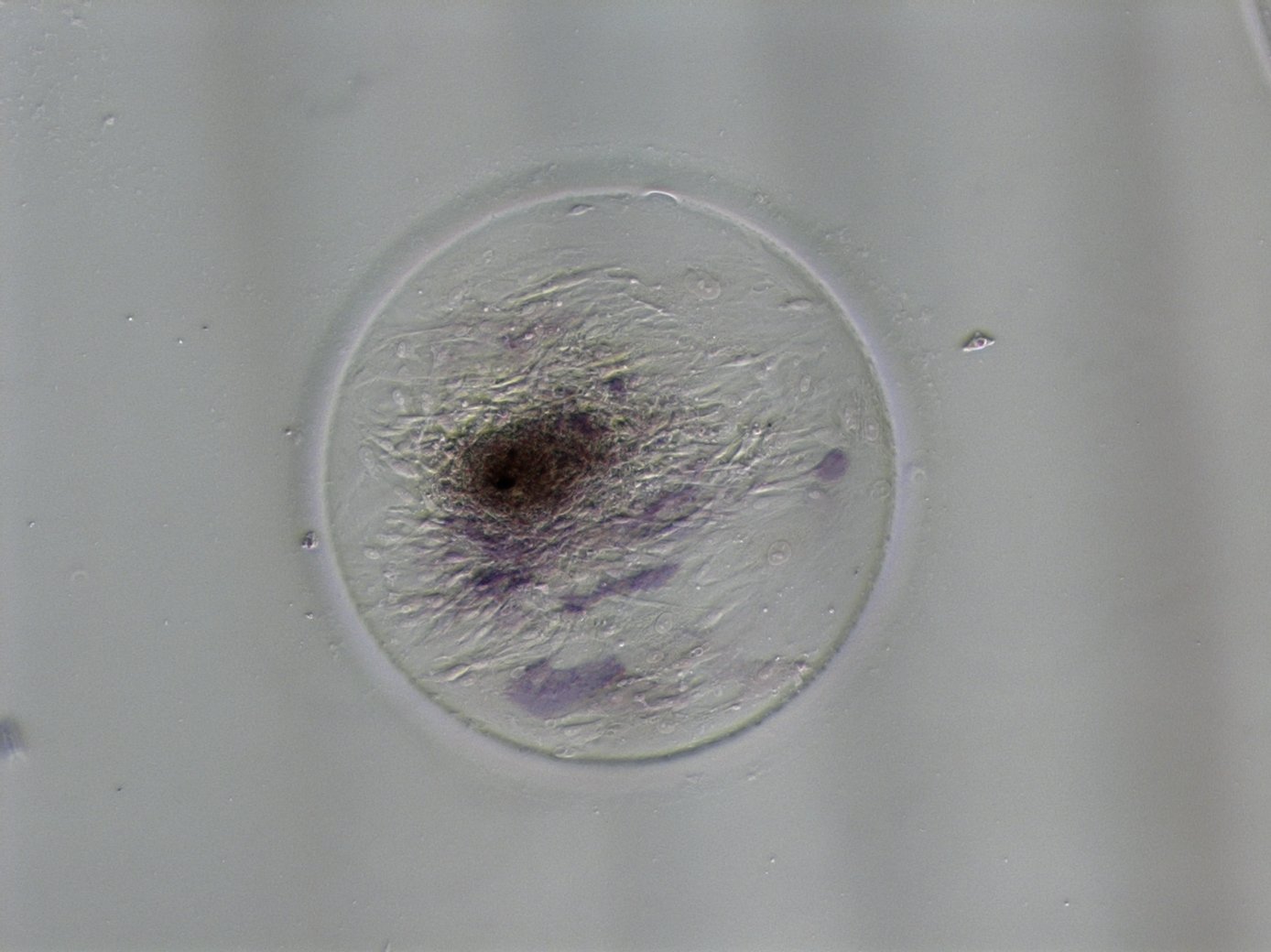

Supplement: S1 File — (ZIP) [file pone.0173647.s002.zip › S1_File/targets/G2_4.jpg]

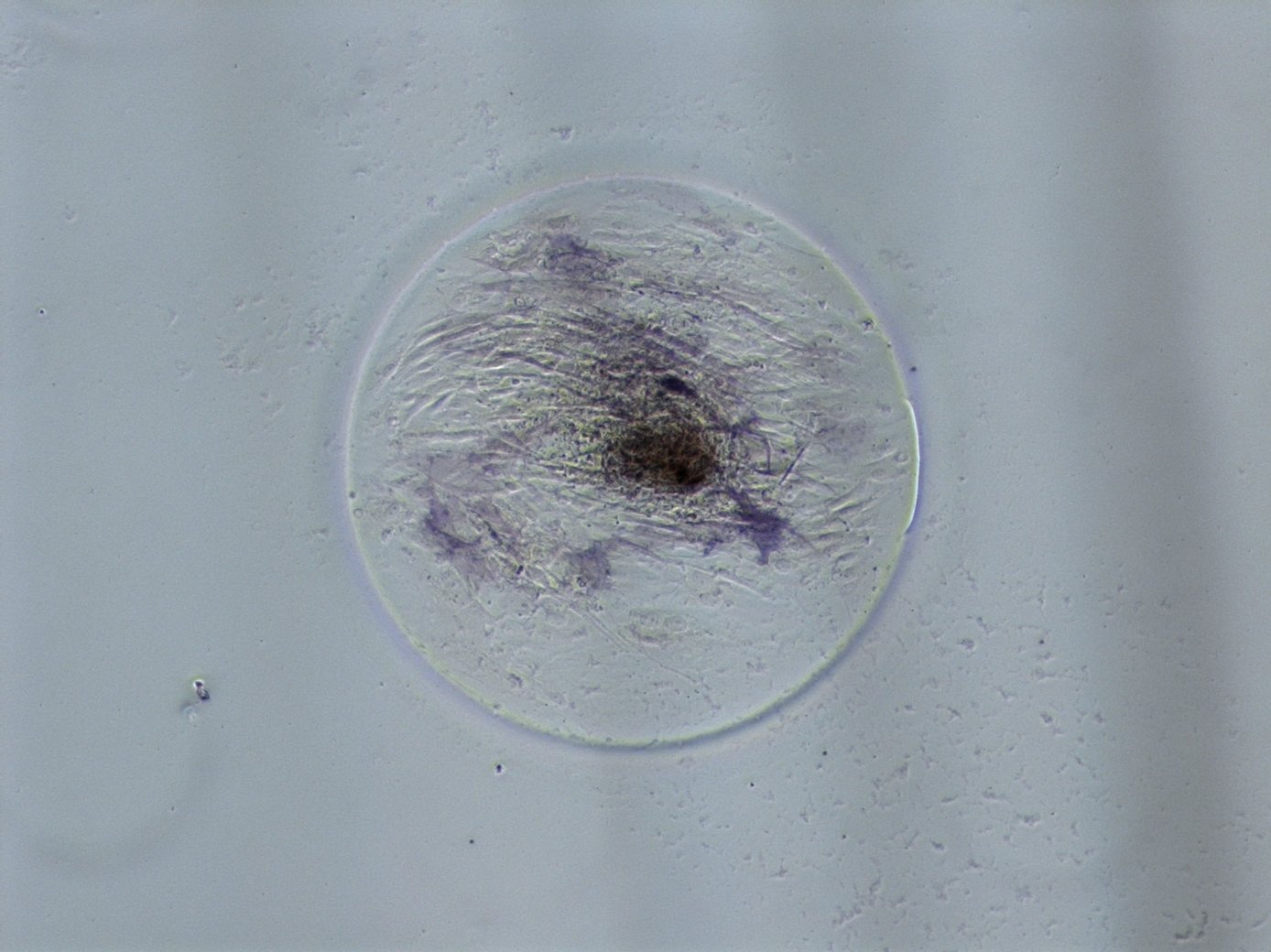

Supplement: S1 File — (ZIP) [file pone.0173647.s002.zip › S1_File/targets/G2_5.jpg]

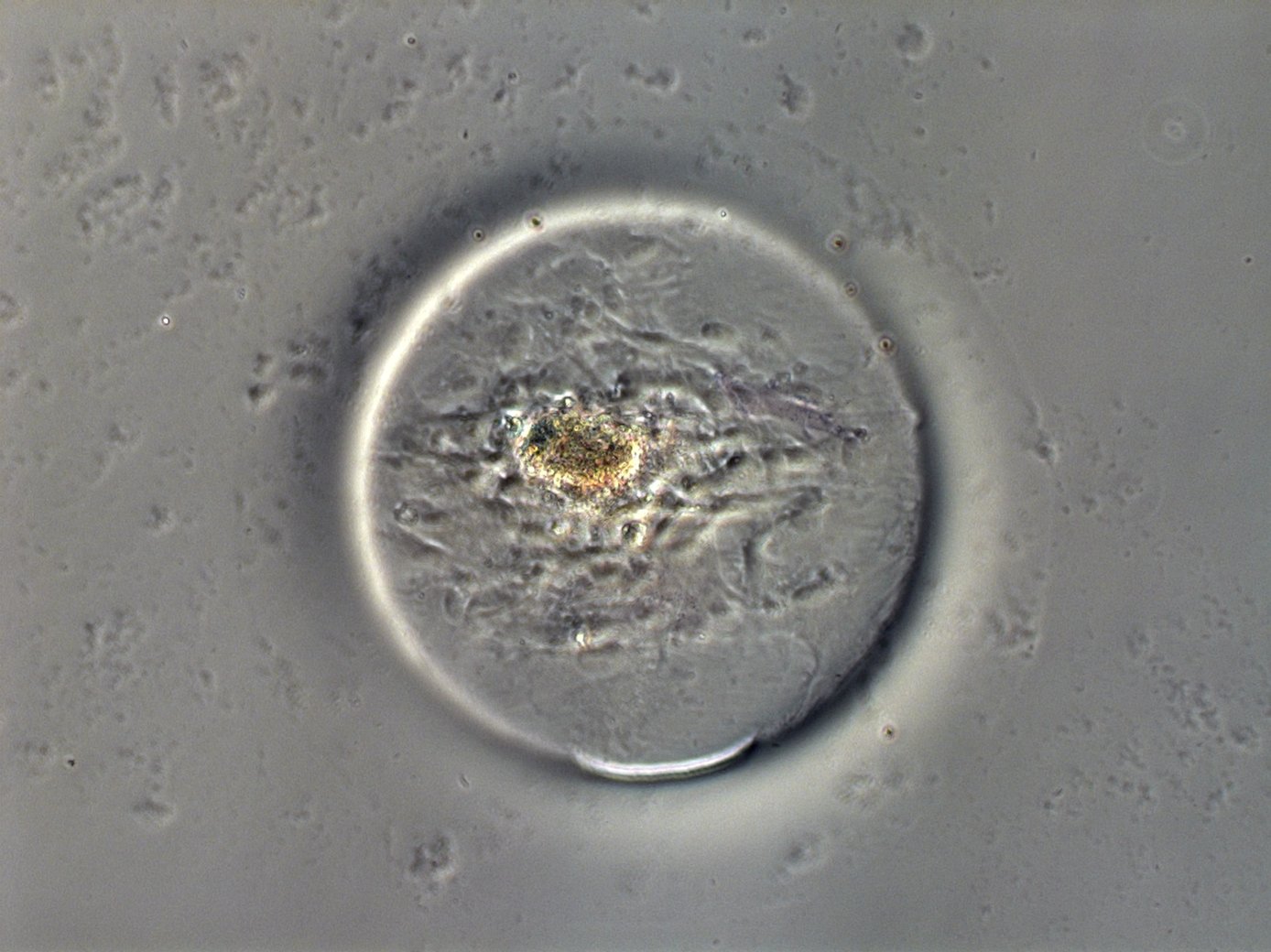

Supplement: S1 File — (ZIP) [file pone.0173647.s002.zip › S1_File/targets/G3_1.jpg]

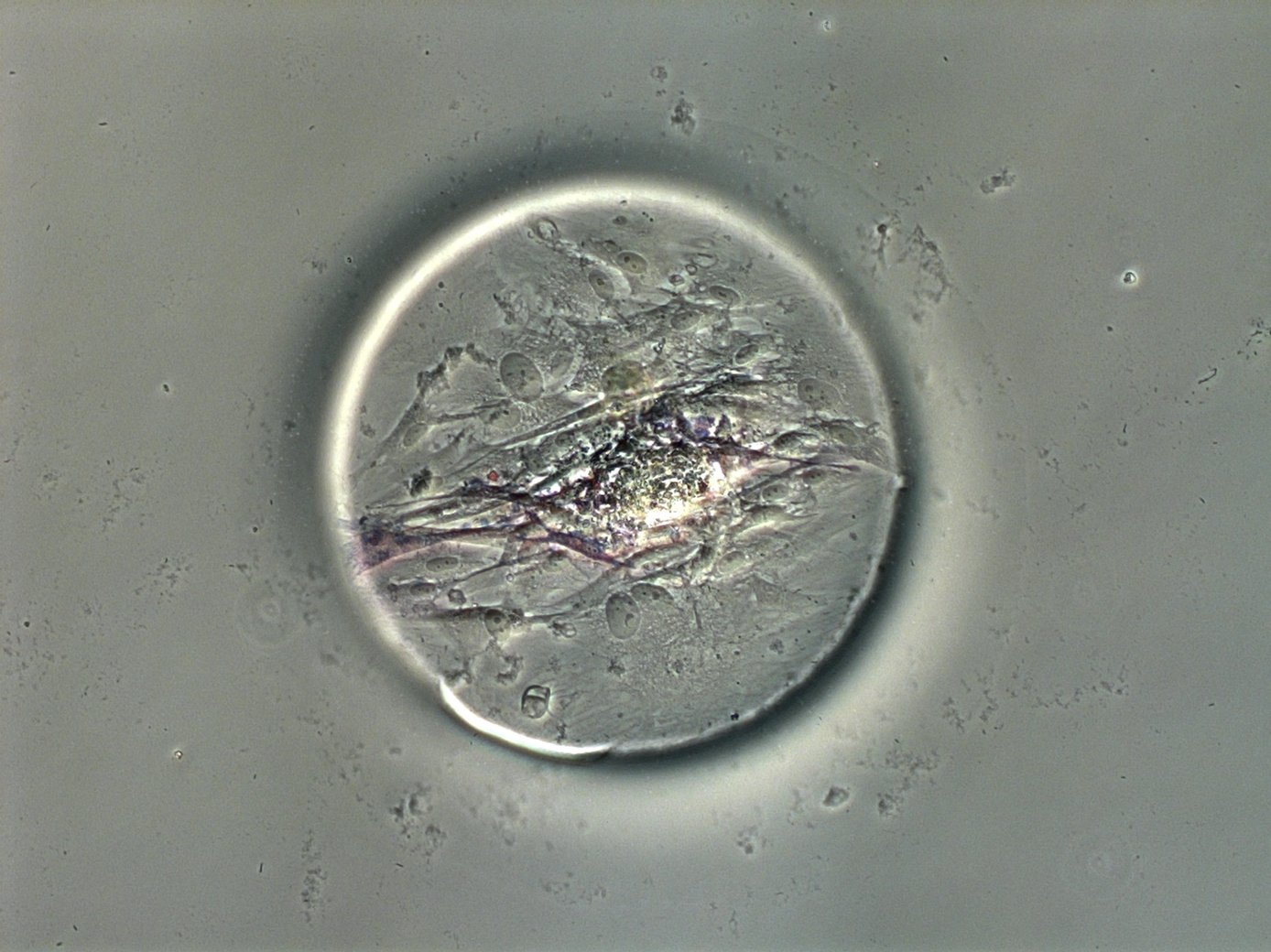

Supplement: S1 File — (ZIP) [file pone.0173647.s002.zip › S1_File/targets/G3_2.jpg]

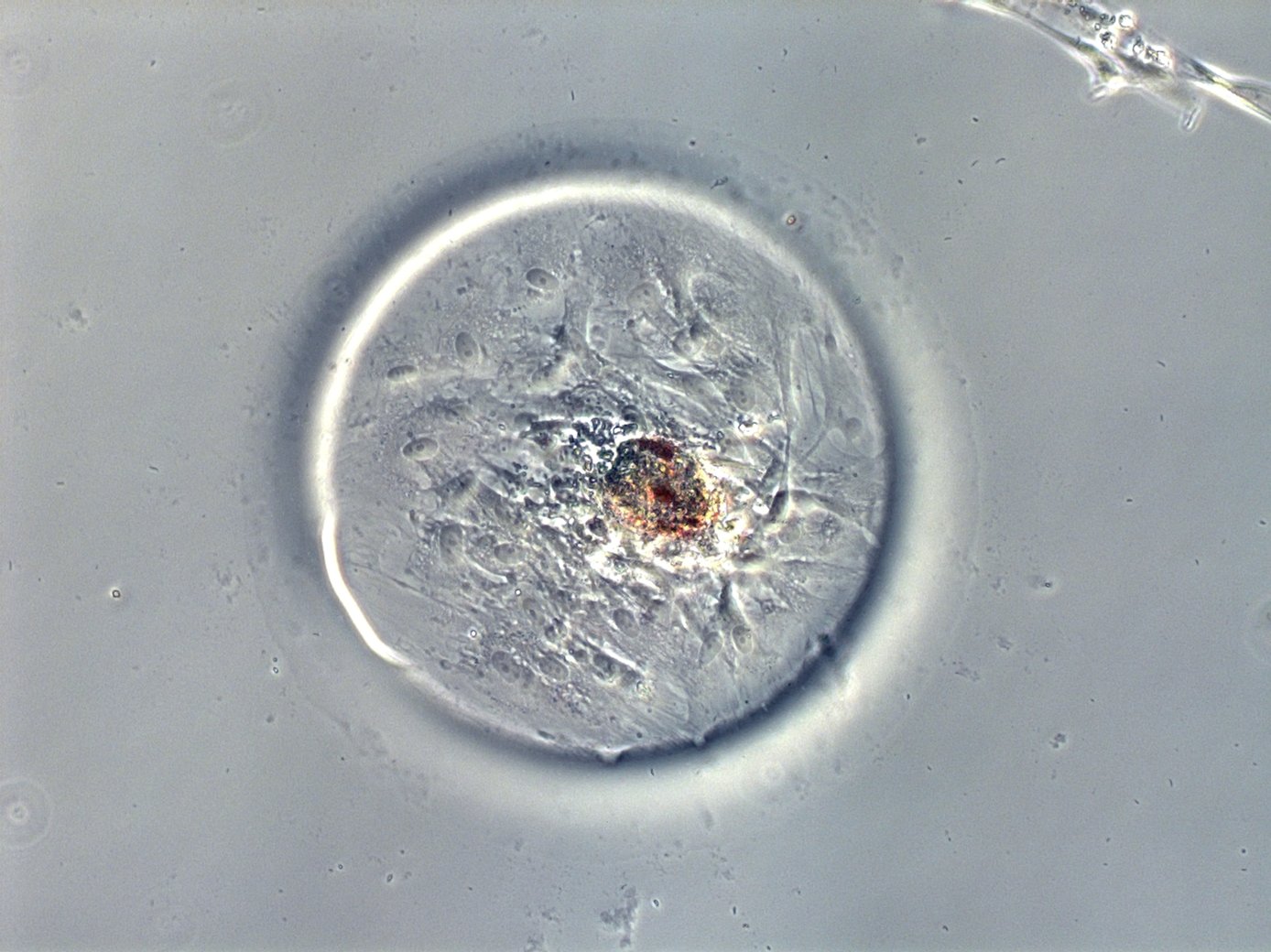

Supplement: S1 File — (ZIP) [file pone.0173647.s002.zip › S1_File/targets/G3_3.jpg]

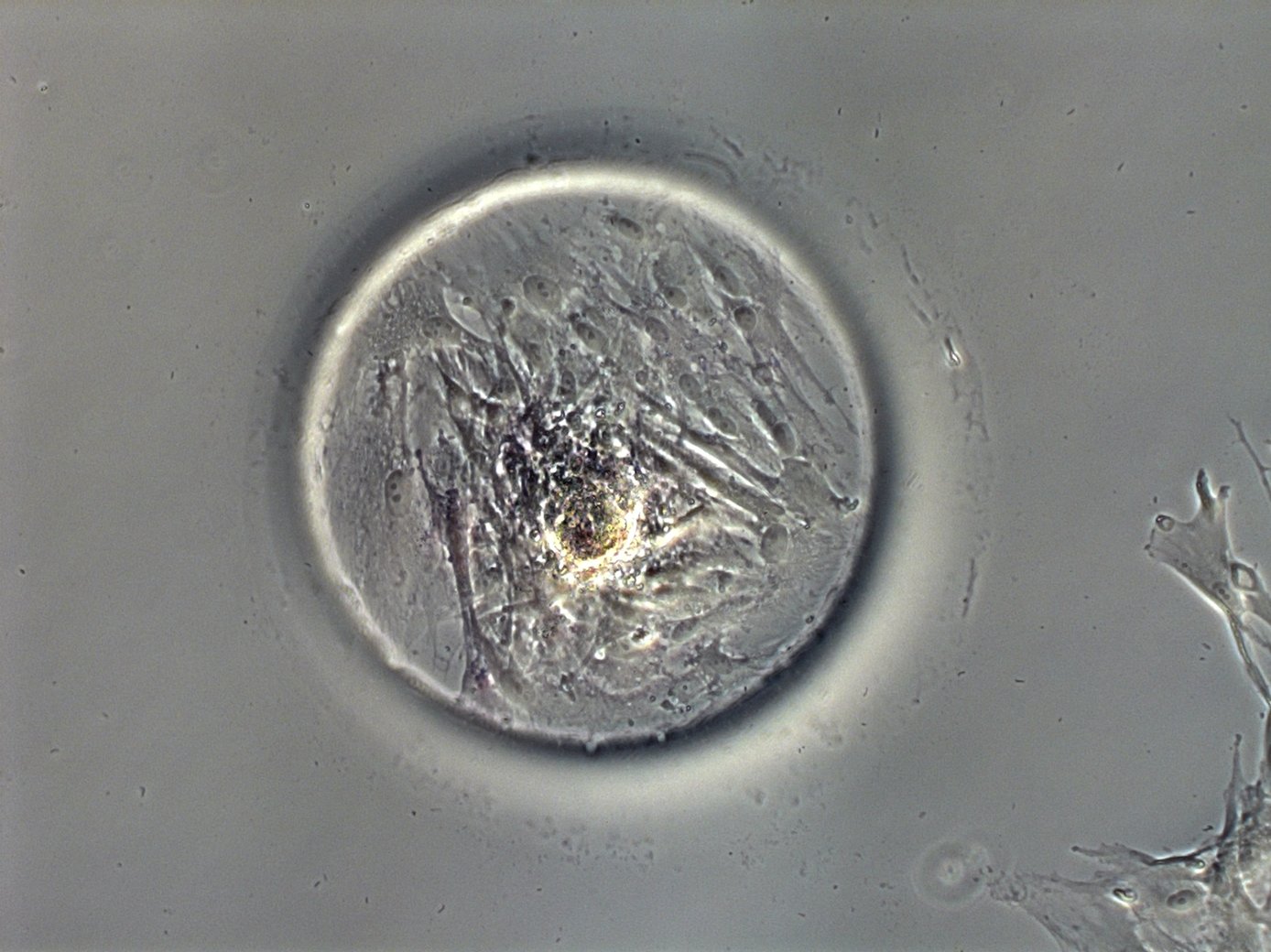

Supplement: S1 File — (ZIP) [file pone.0173647.s002.zip › S1_File/targets/G3_4.jpg]

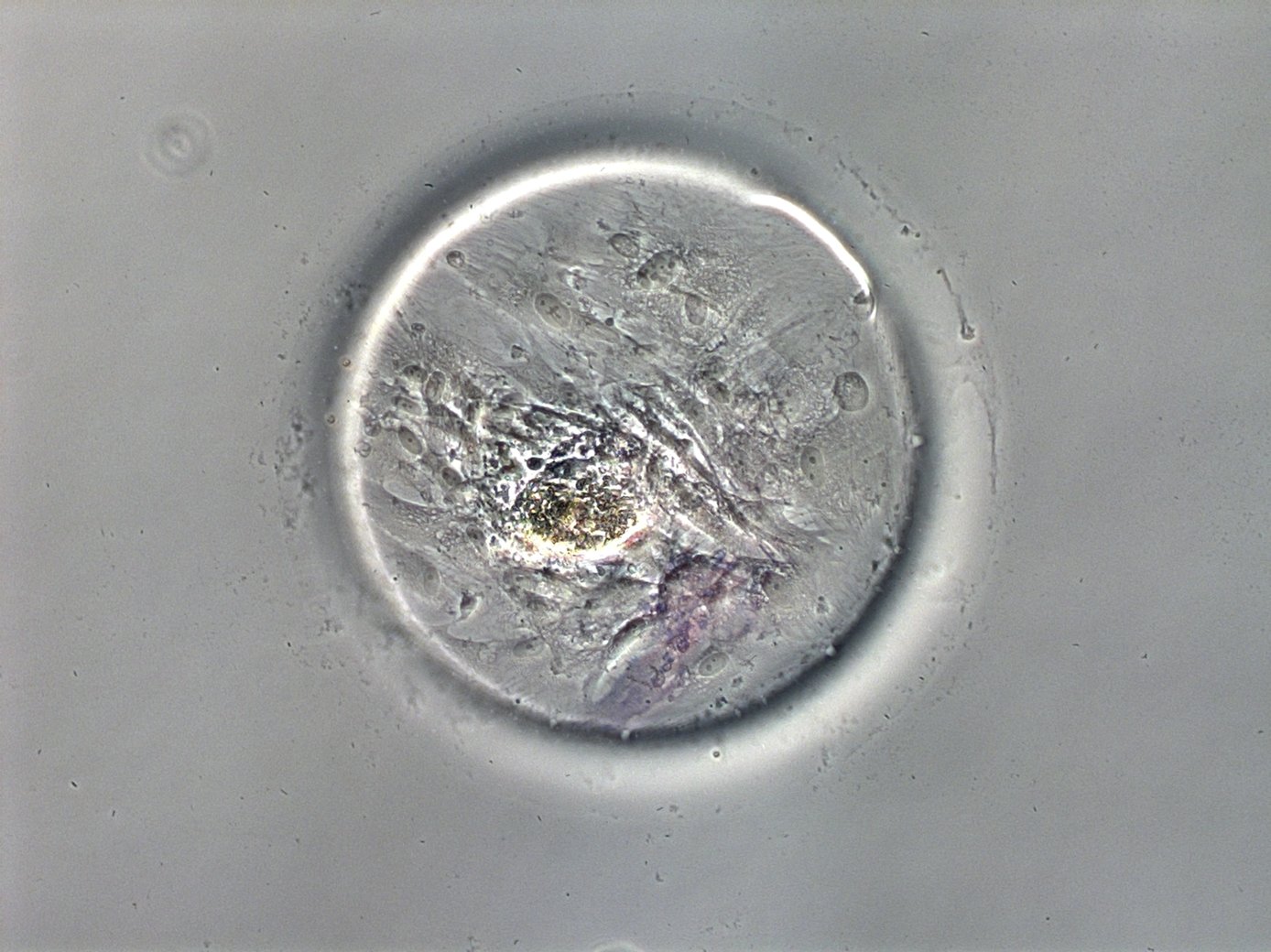

Supplement: S1 File — (ZIP) [file pone.0173647.s002.zip › S1_File/targets/G3_5.jpg]

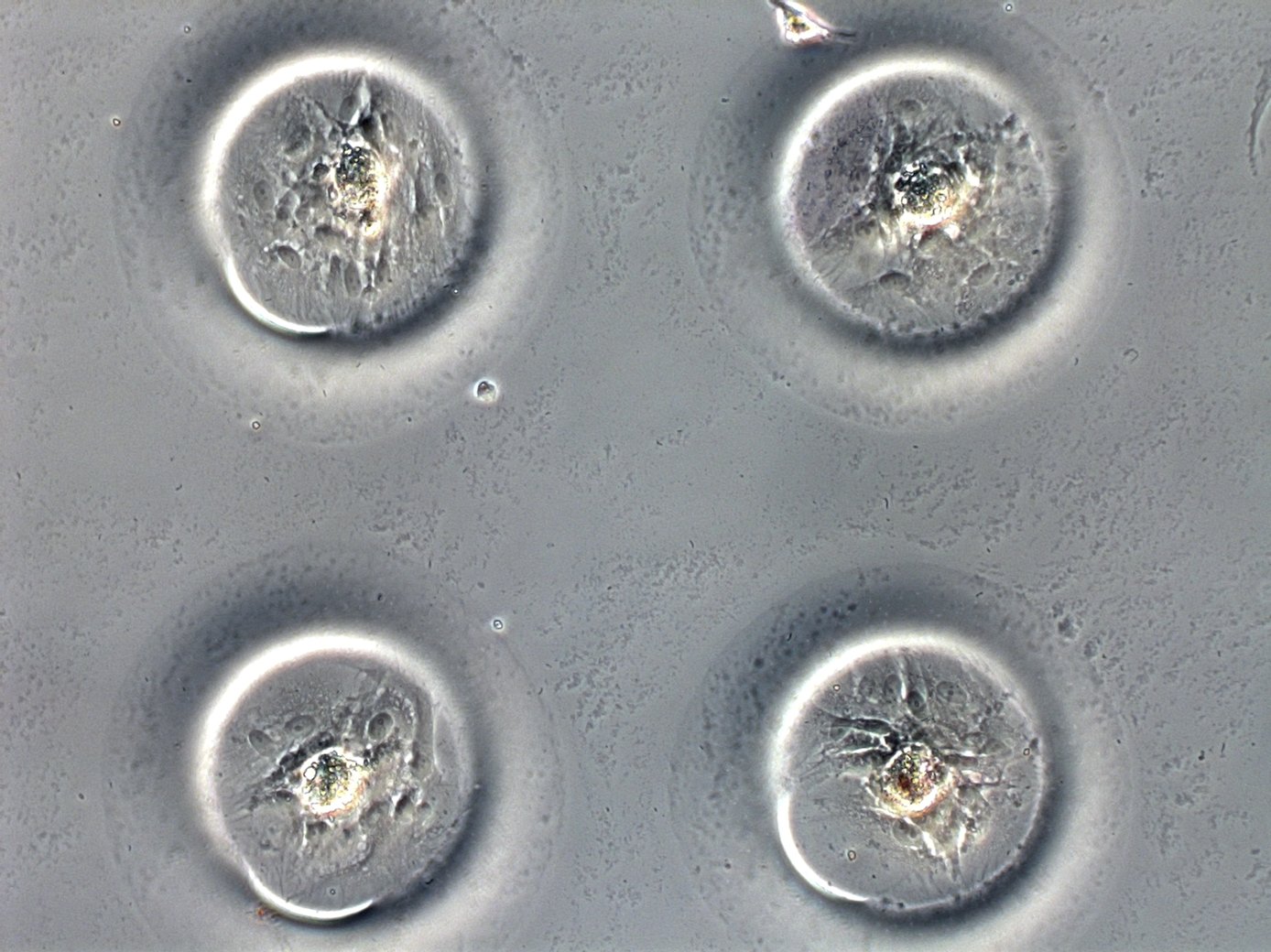

Supplement: S1 File — (ZIP) [file pone.0173647.s002.zip › S1_File/targets/G4_1.jpg]

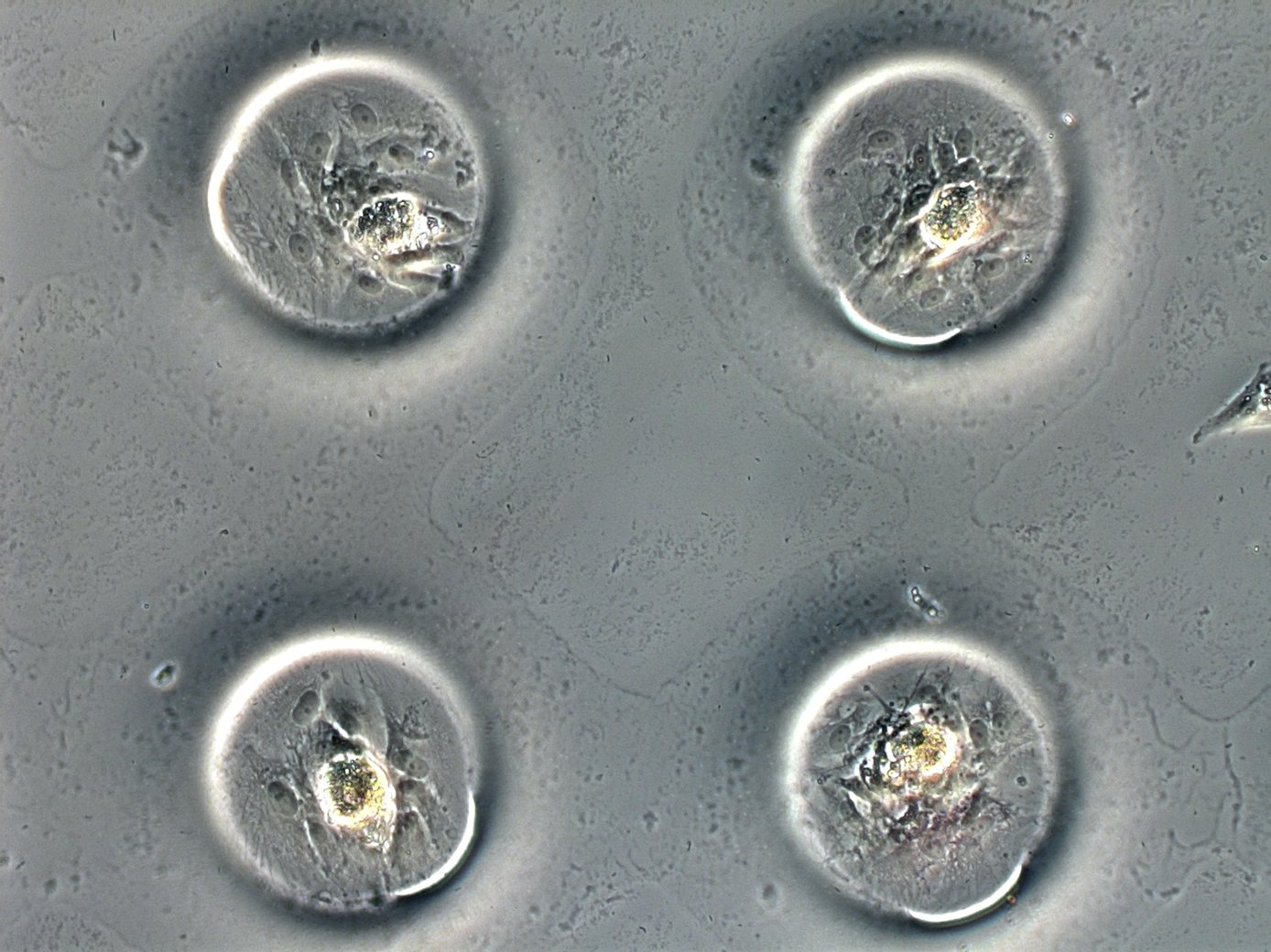

Supplement: S1 File — (ZIP) [file pone.0173647.s002.zip › S1_File/targets/G4_2.jpg]

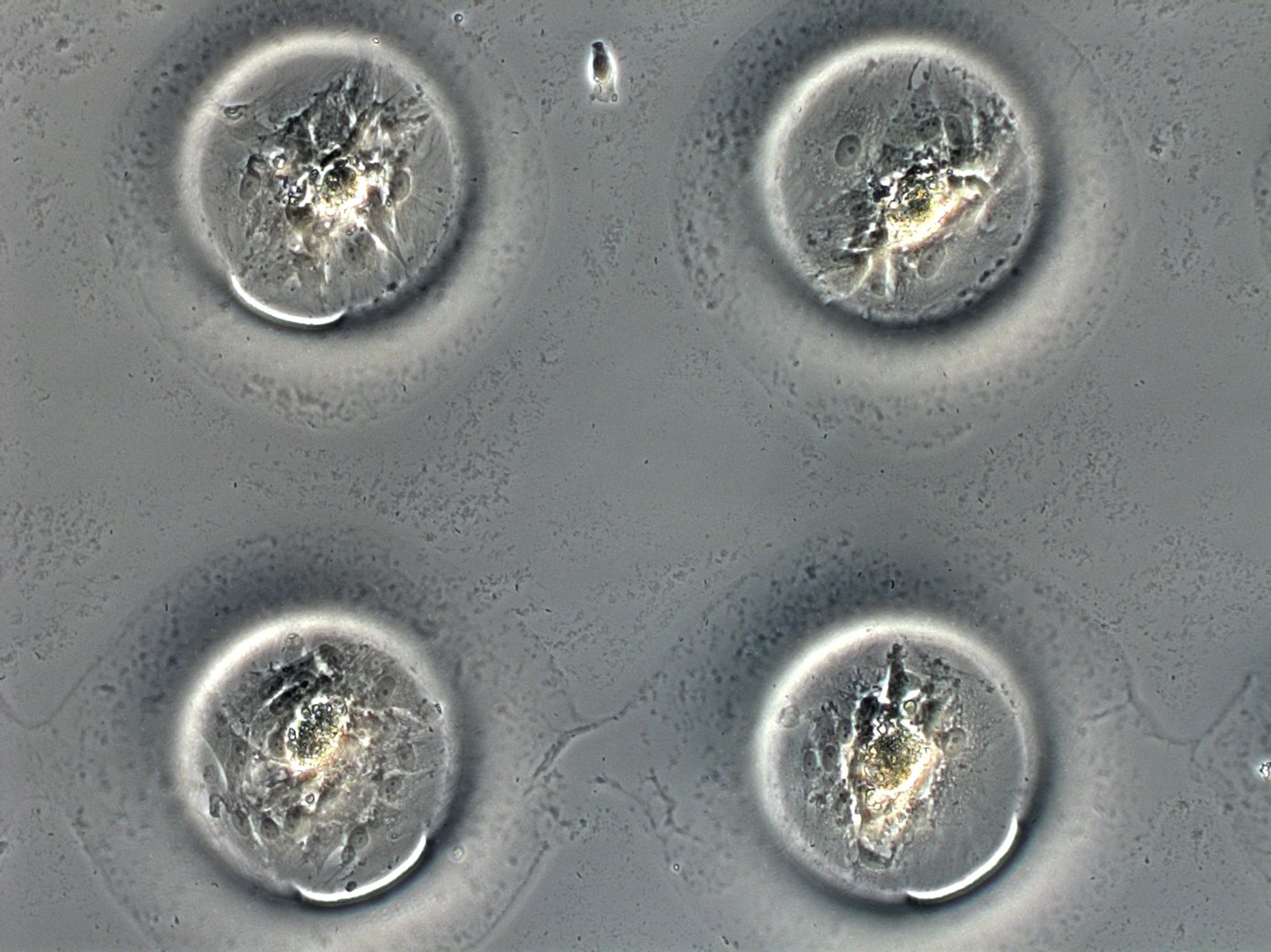

Supplement: S1 File — (ZIP) [file pone.0173647.s002.zip › S1_File/targets/G4_3.jpg]

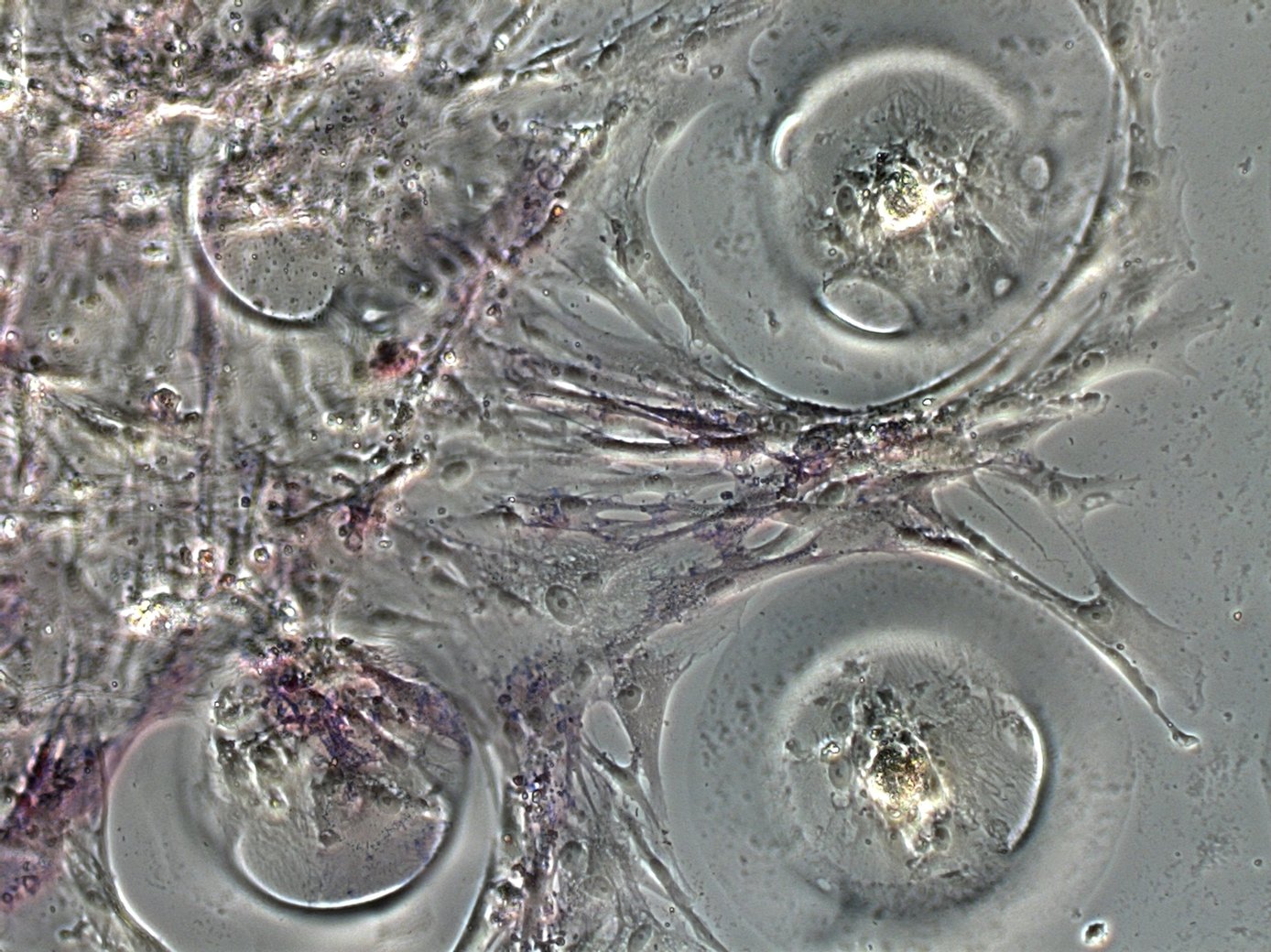

Supplement: S1 File — (ZIP) [file pone.0173647.s002.zip › S1_File/targets/G4_4.jpg]

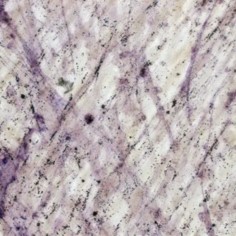

Supplement: S1 File — (ZIP) [file pone.0173647.s002.zip › S1_File/targets/GC_1.jpg]

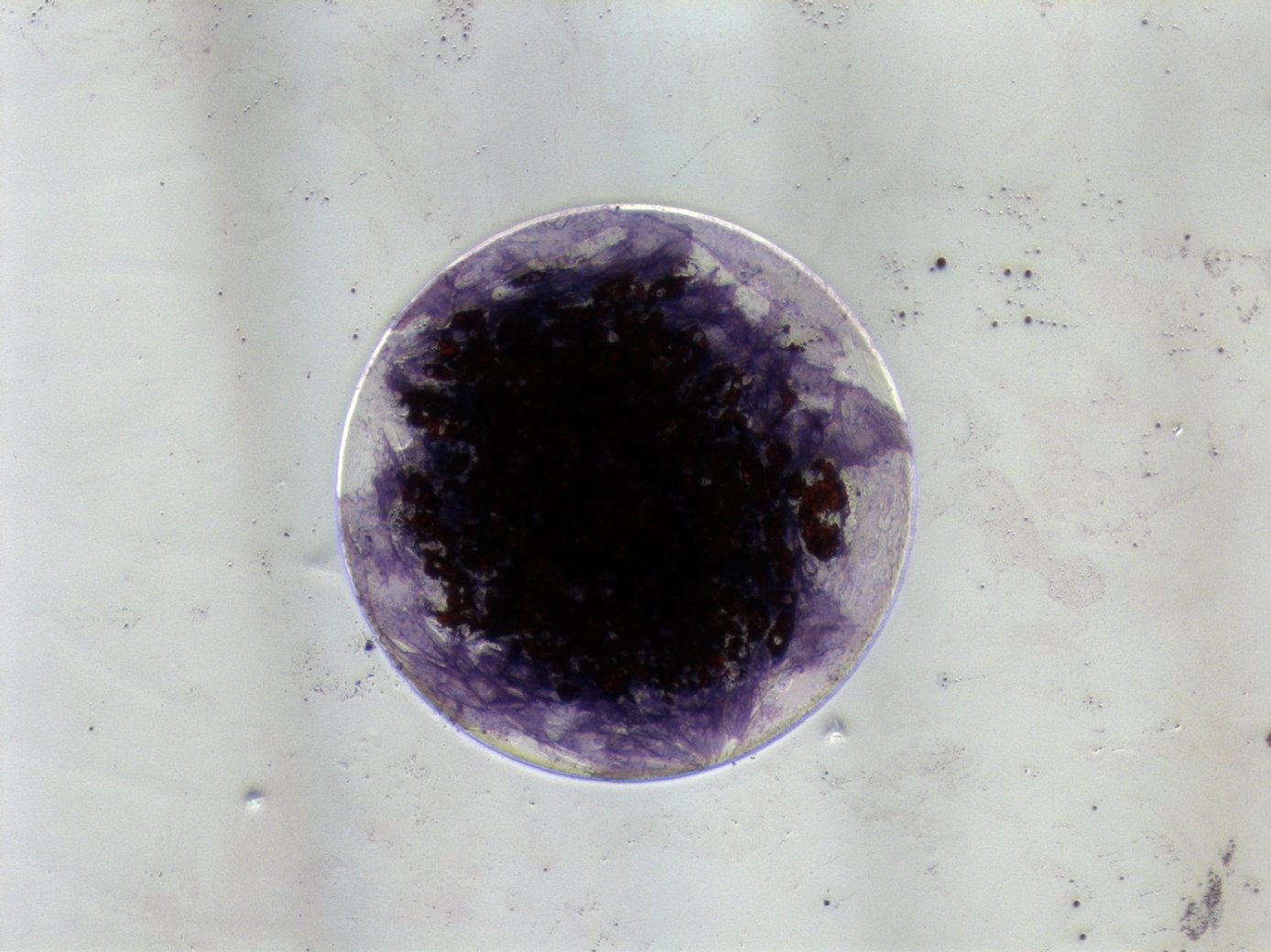

Supplement: S1 File — (ZIP) [file pone.0173647.s002.zip › S1_File/targets/M2_1.jpg]

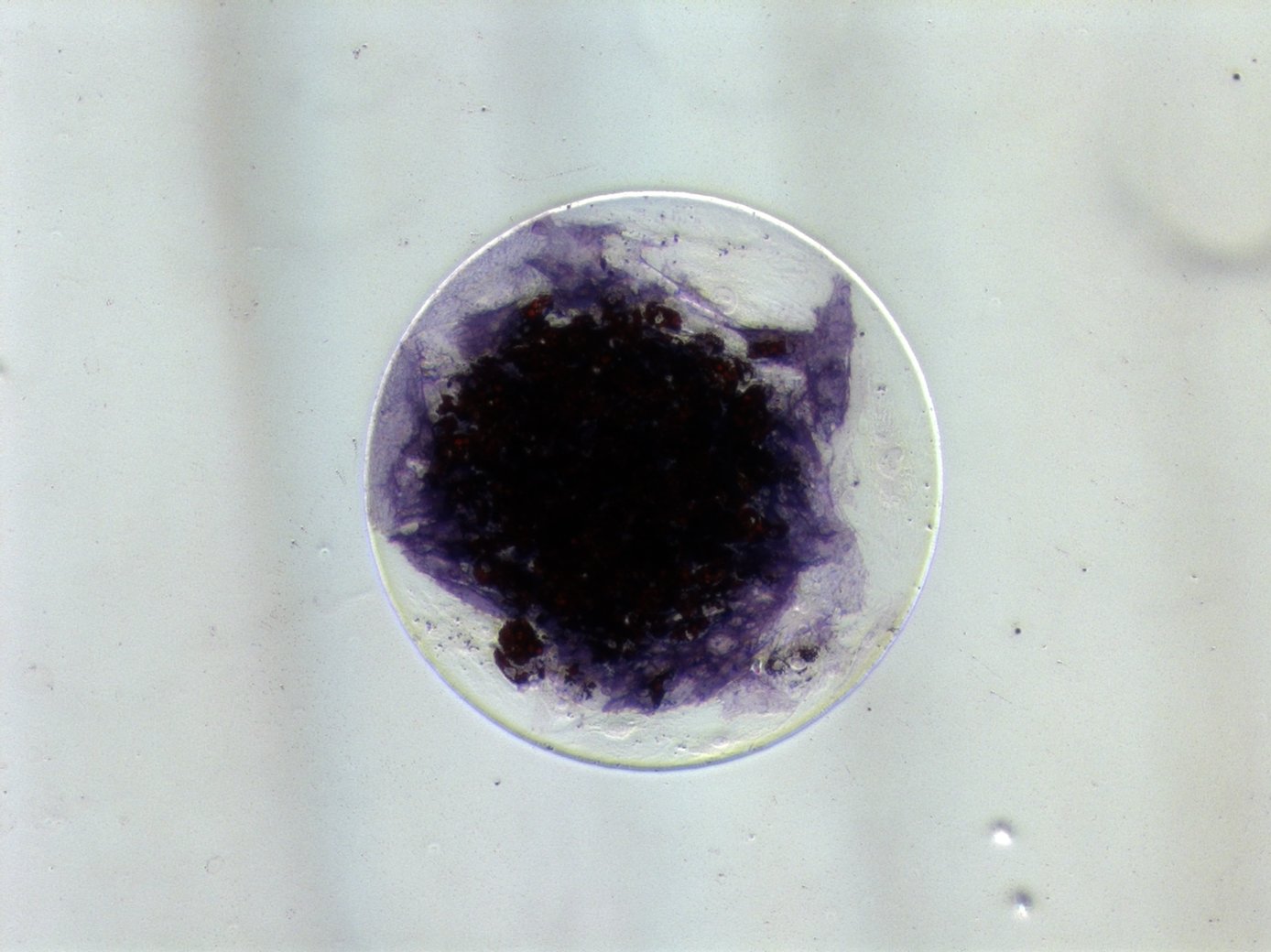

Supplement: S1 File — (ZIP) [file pone.0173647.s002.zip › S1_File/targets/M2_2.jpg]

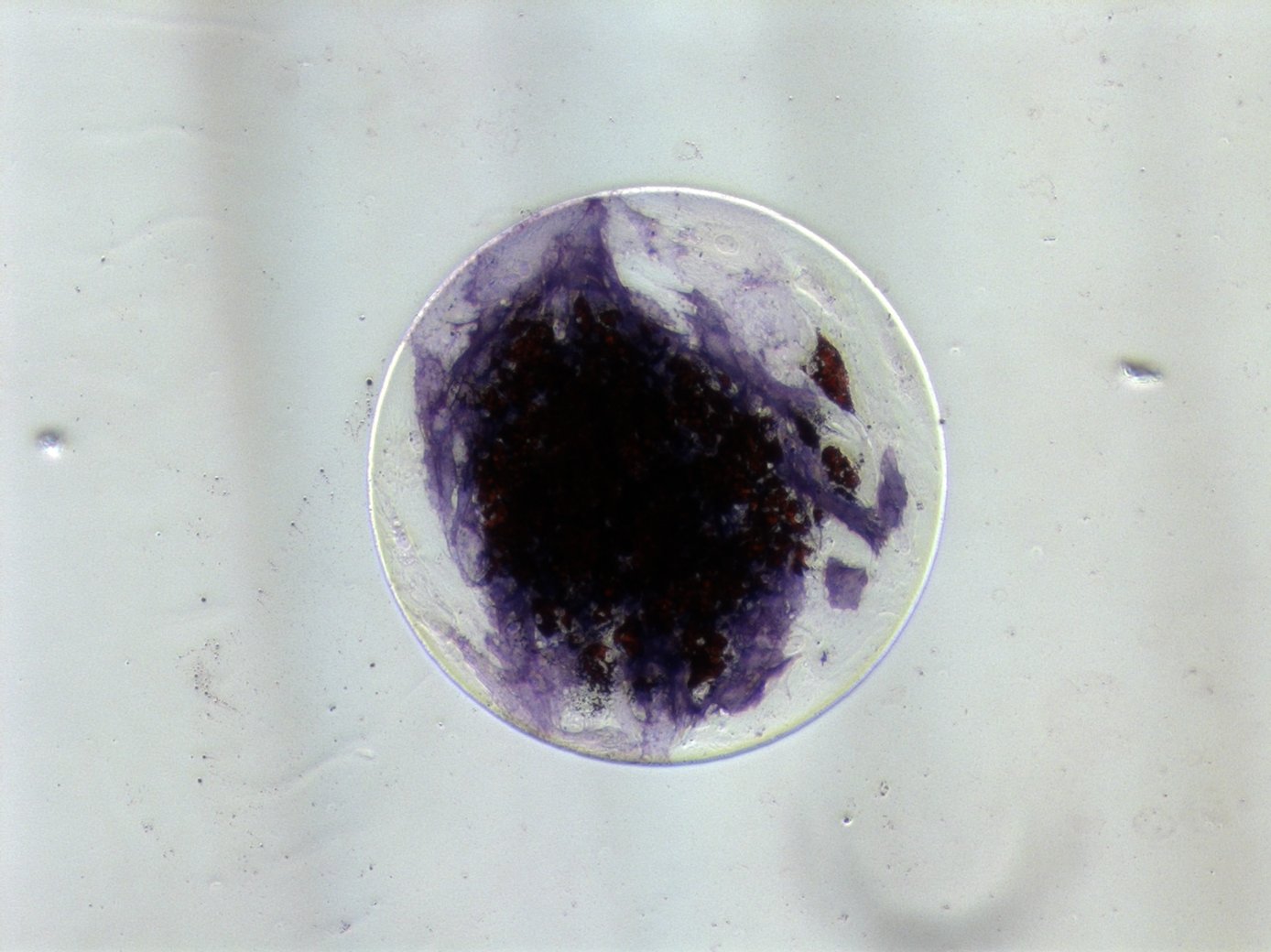

Supplement: S1 File — (ZIP) [file pone.0173647.s002.zip › S1_File/targets/M2_3.jpg]

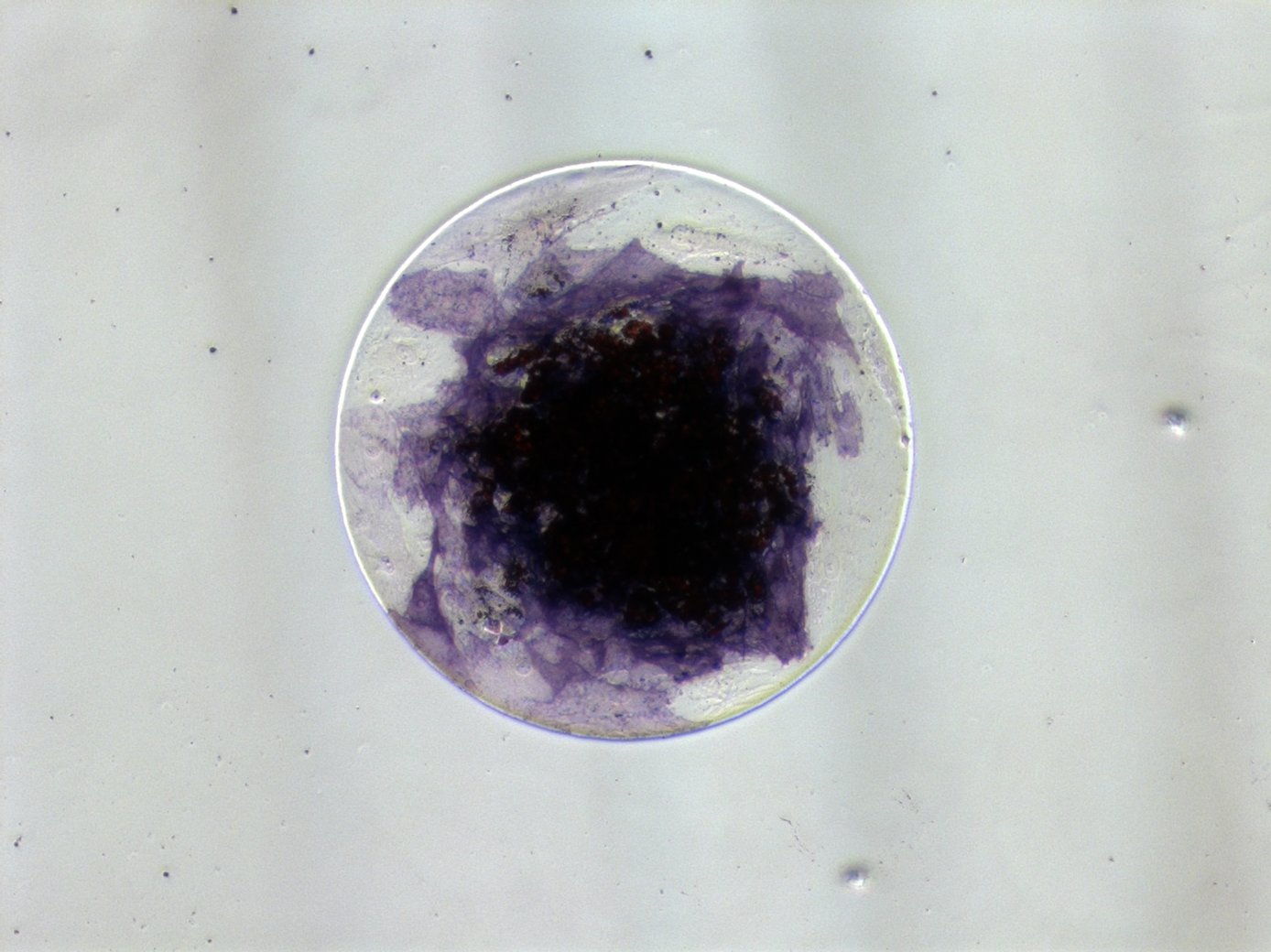

Supplement: S1 File — (ZIP) [file pone.0173647.s002.zip › S1_File/targets/M2_4.jpg]

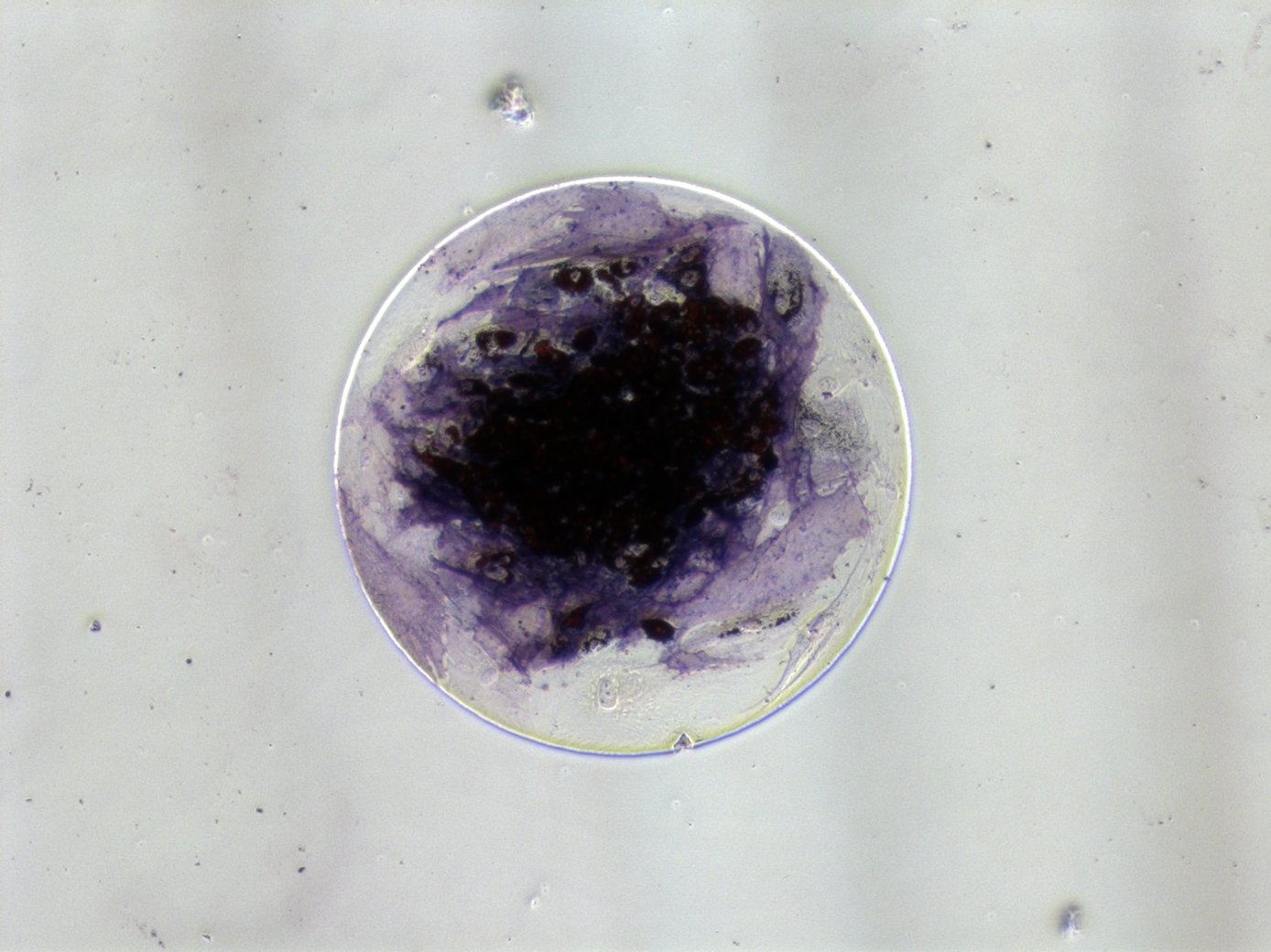

Supplement: S1 File — (ZIP) [file pone.0173647.s002.zip › S1_File/targets/M2_5.jpg]

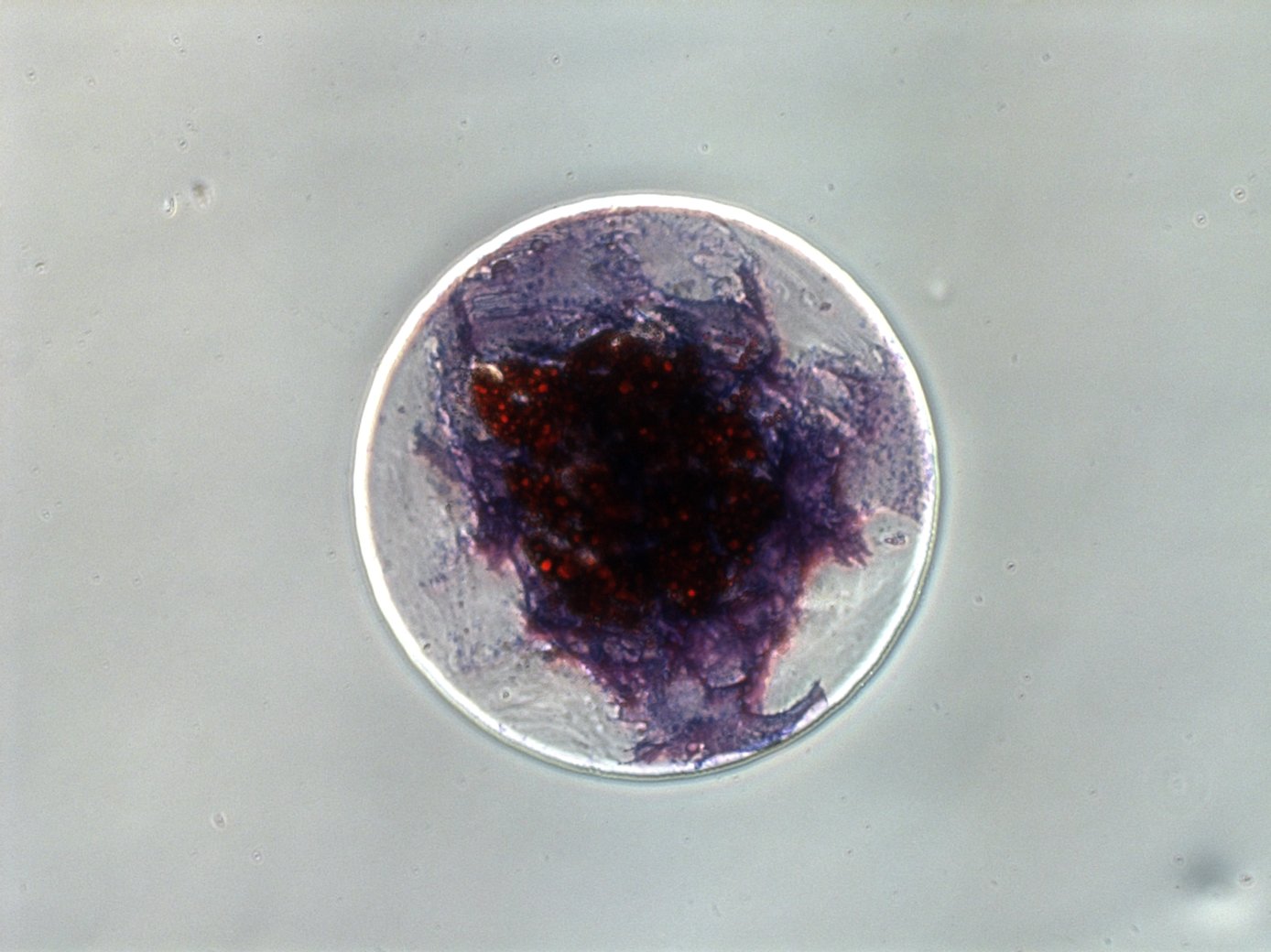

Supplement: S1 File — (ZIP) [file pone.0173647.s002.zip › S1_File/targets/M3_1.jpg]

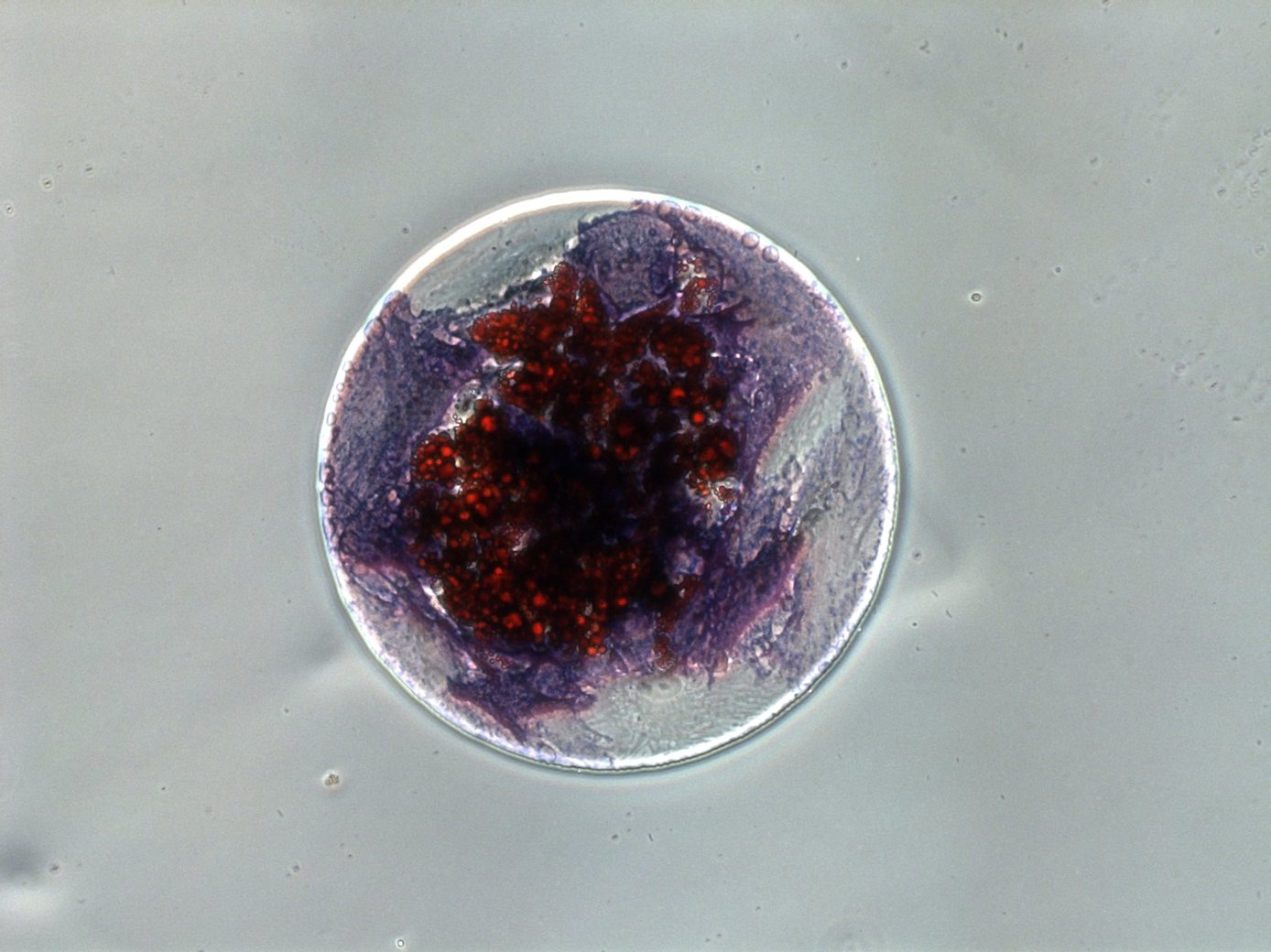

Supplement: S1 File — (ZIP) [file pone.0173647.s002.zip › S1_File/targets/M3_2.jpg]

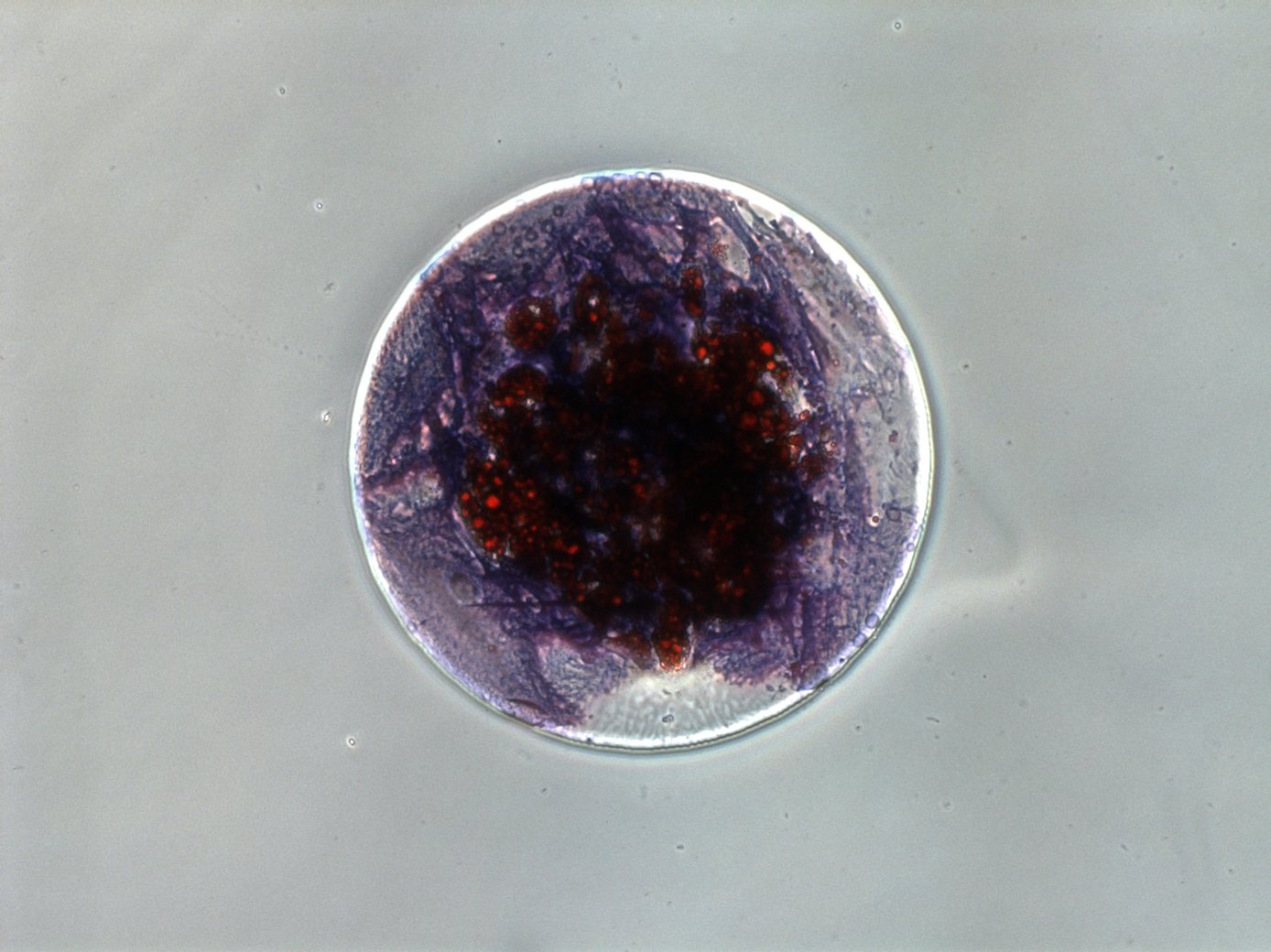

Supplement: S1 File — (ZIP) [file pone.0173647.s002.zip › S1_File/targets/M3_3.jpg]

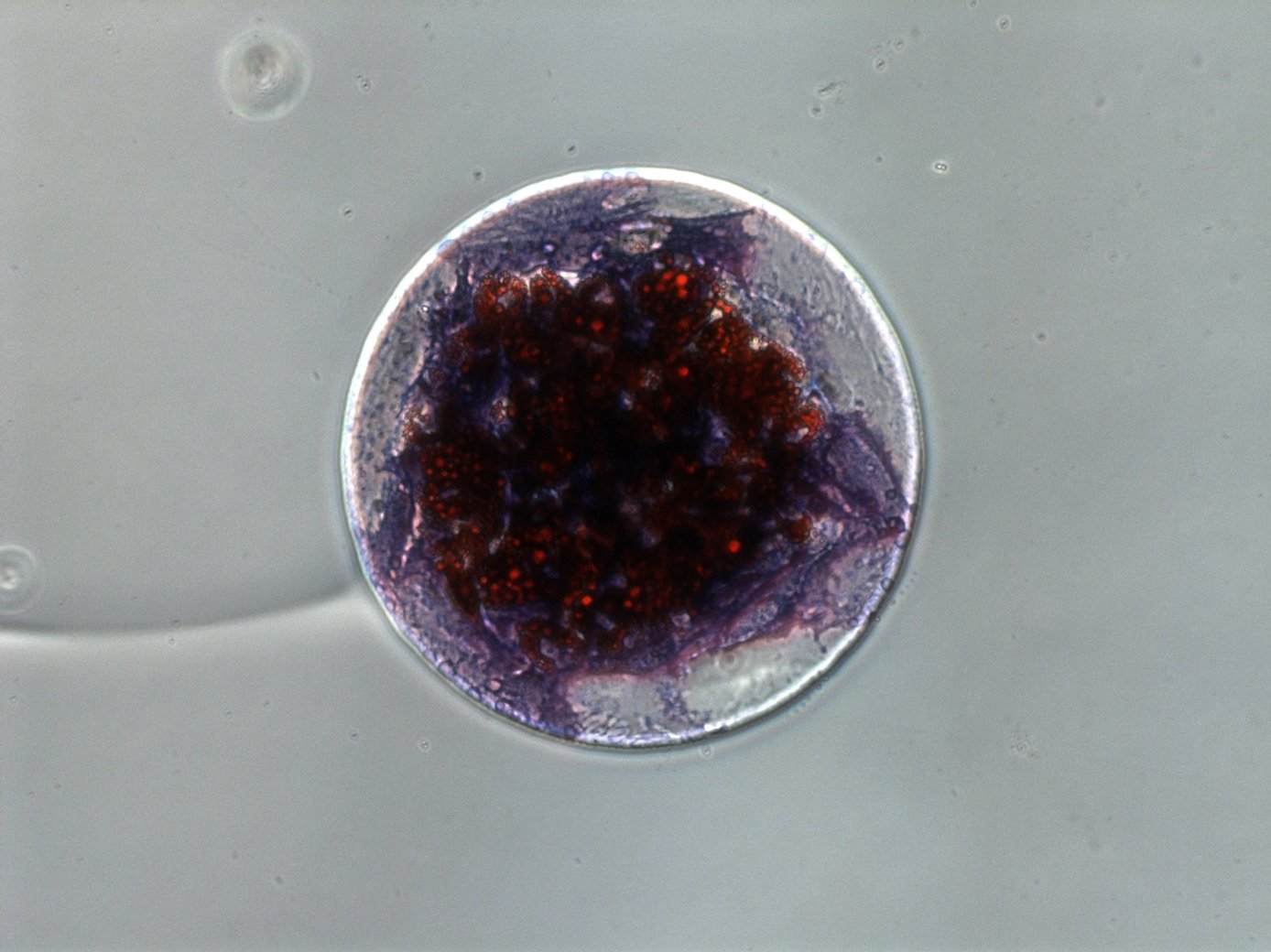

Supplement: S1 File — (ZIP) [file pone.0173647.s002.zip › S1_File/targets/M3_4.jpg]

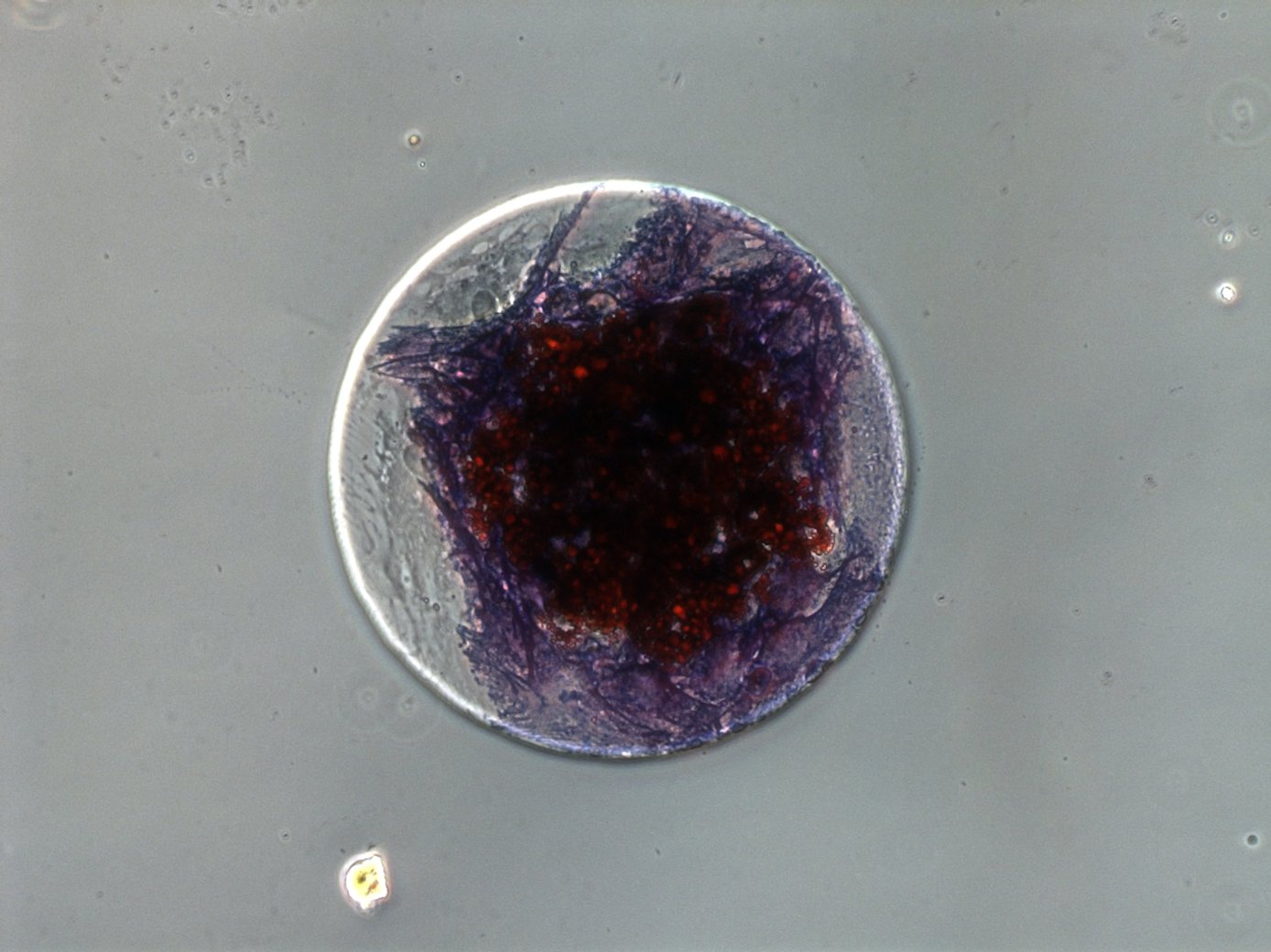

Supplement: S1 File — (ZIP) [file pone.0173647.s002.zip › S1_File/targets/M3_5.jpg]

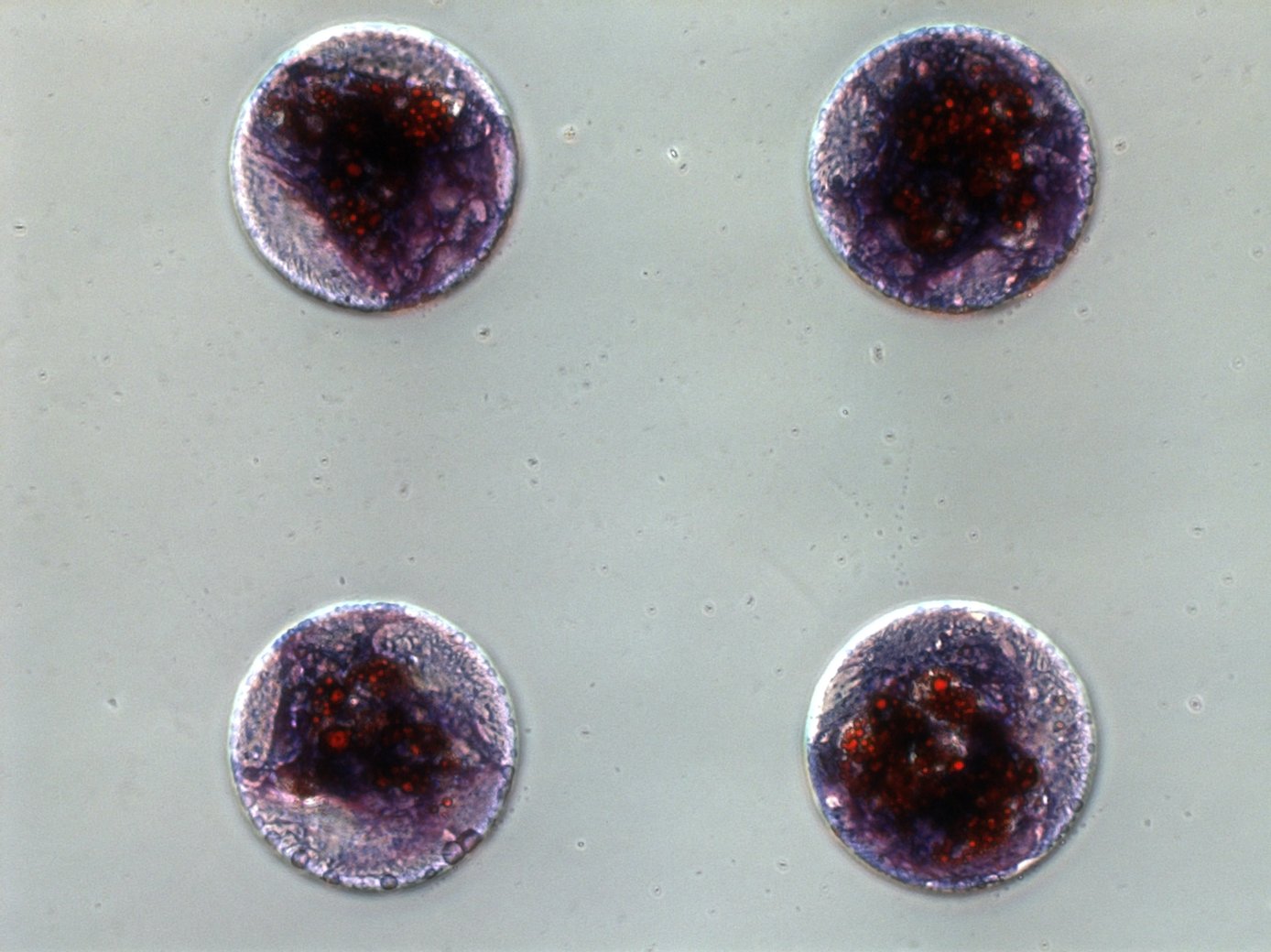

Supplement: S1 File — (ZIP) [file pone.0173647.s002.zip › S1_File/targets/M4_1.jpg]

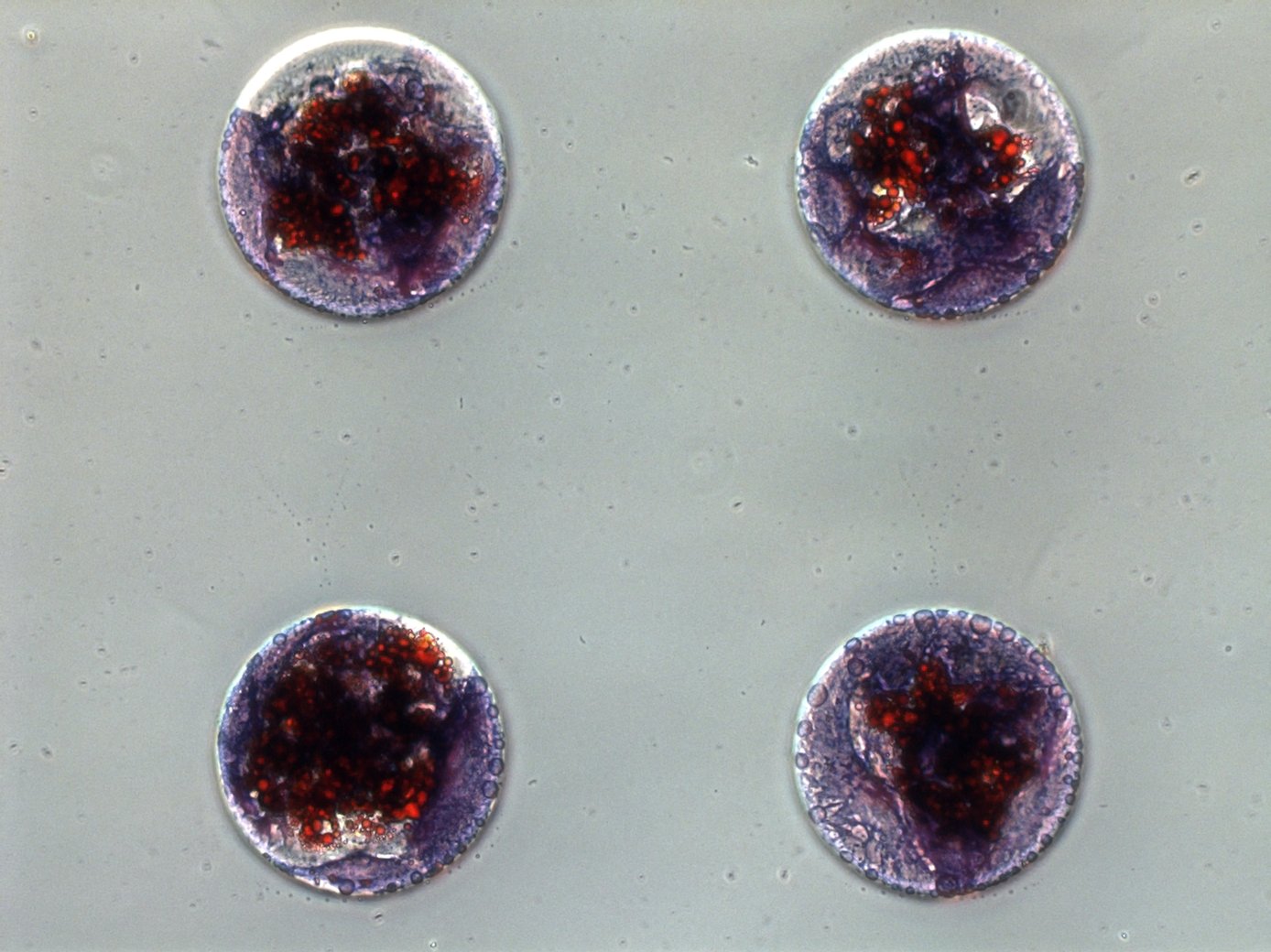

Supplement: S1 File — (ZIP) [file pone.0173647.s002.zip › S1_File/targets/M4_2.jpg]

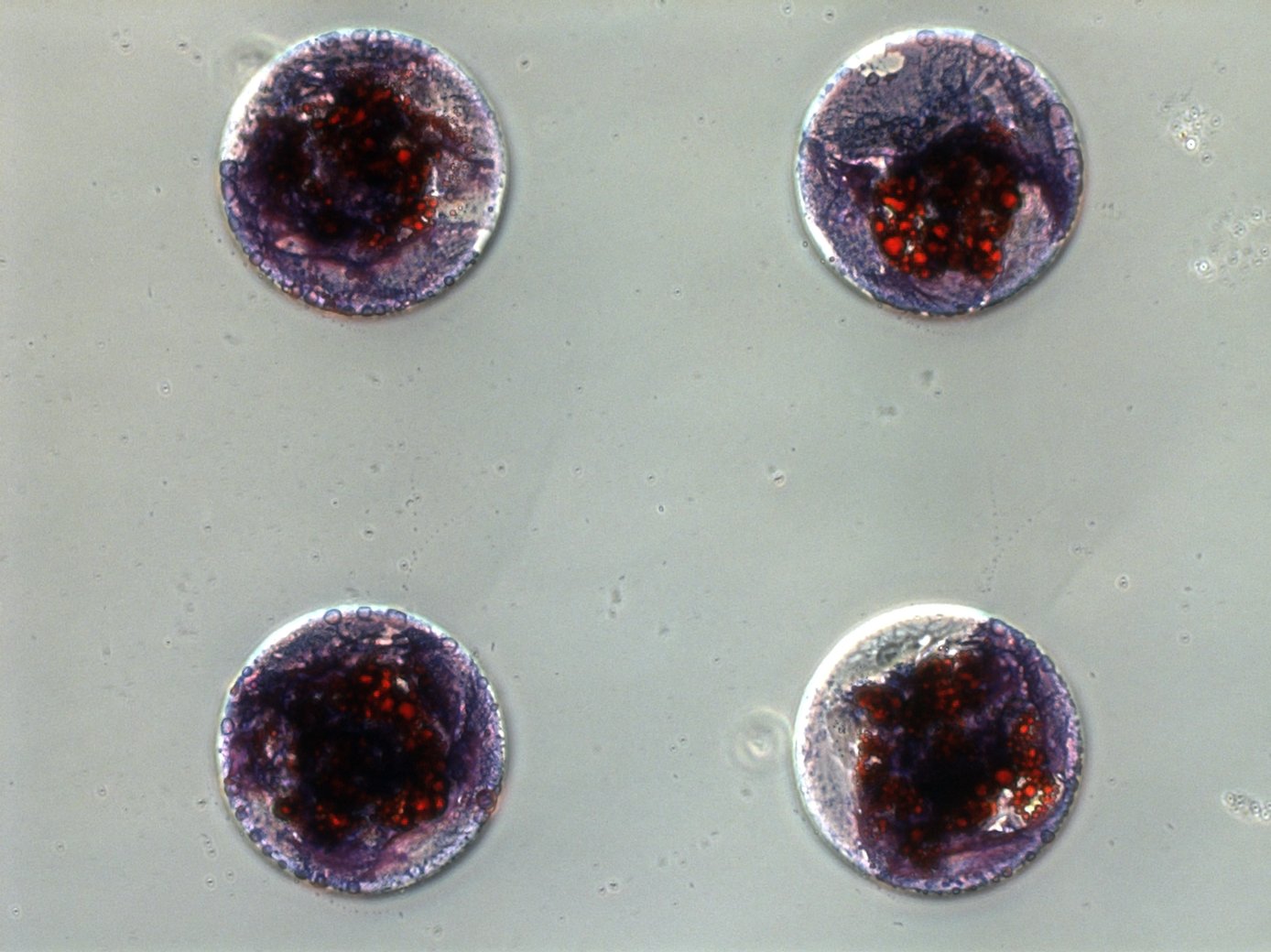

Supplement: S1 File — (ZIP) [file pone.0173647.s002.zip › S1_File/targets/M4_3.jpg]
