# Supplementary material for: Simple agarose micro-confinement array and machine-learning-based classification for analyzing the patterned differentiation of mesenchymal stem cells
Source: PLoS One. 2017 Apr 5;12(4):e0173647. doi: 10.1371/journal.pone.0173647 (PMC5381775; doi:10.1371/journal.pone.0173647)
Supplement: S2 File — (DOCX) [file pone.0173647.s003.docx]

**Supporting information**

**Simple agarose micro-confinement array and machine-learning-based classification for analyzing the patterned differentiation of mesenchymal stem cells**

Nobuyuki Tanaka, Tadahiro Yamashita, Asako Sato, Viola Vogel, and Yo Tanaka

**S3 File. Materials and methods for staining and observation of nuclei.**

To visualize the 3D distribution of cells, the nuclei of hMSCs cultured inside patterned agar gel walls for 15 days were stained with 2 μg/mL DAPI solution (D1306) (Invitrogen, Carlsbad, CA) following fixation and permeabilization by 4% paraformaldehyde (P6148) (Sigma-Aldrich, St. Louis, MO) and 0.1% Triton X-100 (BP151) (Thermo Fisher Scientific, Carlsbad, CA) diluted in PBS, respectively. The nuclei were observed by confocal laser scanning microscope (TCS SP5 SMD) (Leica, Wetzlar, Germany) with 5 μm of Z stepping using a 20x objective lens (N.A. 0.7). The supplementary movie was created from the stack of confocal images to visualize the cross-sectional view at arbitrary Y position.
